# Defining structural and evolutionary modules in proteins: a community detection approach to explore sub-domain architecture

**DOI:** 10.1186/1472-6807-13-20

**Published:** 2013-10-16

**Authors:** Jose Sergio Hleap, Edward Susko, Christian Blouin

**Affiliations:** 1 Department of Biochemistry and Molecular Biology, Dalhousie University, Halifax, NS, B3H 4R2, Canada; 2 Department of Mathematics and Statistics, Dalhousie University, Halifax, NS, B3H 3J5, Canada; 3 Department of Computer Science, Dalhousie University, Halifax, NS, B3H 1W5, Canada

## Abstract

**Background:**

Assessing protein modularity is important to understand protein evolution. Still the question of the existence of a sub-domain modular architecture remains. We propose a graph-theory approach with significance and power testing to identify modules in protein structures. In the first step, clusters are determined by optimizing the partition that maximizes the modularity score. Second, each cluster is tested for significance. Significant clusters are referred to as modules. Evolutionary modules are identified by analyzing homologous structures. Dynamic modules are inferred from sets of snapshots of molecular simulations. We present here a methodology to identify sub-domain architecture robustly, biologically meaningful, and statistically supported.

**Results:**

The robustness of this new method is tested using simulated data with known modularity. Modules are correctly identified even when there is a low correlation between landmarks within a module. We also analyzed the evolutionary modularity of a data set of *α*-amylase catalytic domain homologs, and the dynamic modularity of the Niemann-Pick C1 (NPC1) protein N-terminal domain.

The *α*-amylase contains an (*α*/*β*)_8_ barrel (TIM barrel) with the polysaccharides cleavage site and a calcium-binding domain. In this data set we identified four robust evolutionary modules, one of which forms the minimal functional TIM barrel topology.

The NPC1 protein is involved in the intracellular lipid metabolism coordinating sterol trafficking. NPC1 N-terminus is the first luminal domain which binds to cholesterol and its oxygenated derivatives. Our inferred dynamic modules in the protein NPC1 are also shown to match functional components of the protein related to the NPC1 disease.

**Conclusions:**

A domain compartmentalization can be found and described in correlation space. To our knowledge, there is no other method attempting to identify sub-domain architecture from the correlation among residues. Most attempts made focus on sequence motifs of protein-protein interactions, binding sites, or sequence conservancy. We were able to describe functional/structural sub-domain architecture related to key residues for starch cleavage, calcium, and chloride binding sites in the *α*-amylase, and sterol opening-defining modules and disease-related residues in the NPC1. We also described the evolutionary sub-domain architecture of the *α*-amylase catalytic domain, identifying the already reported minimum functional TIM barrel.

## Background

Jacob [1] stated that "*Nature is a tinkerer and not an inventor*". We still use a similar thought relating protein evolution, since domains are accepted as the protein evolutionary modules, and its modular reuse has been demonstrated in all domains of life [2]. This modularity gives protein structures enhanced flexibility [3] and might influence its ability to respond to selection. This ability is of main concern for evolutionary biology and is related to the robustness of a system [4,5]. Robustness is the ability of a system to maintain its function under perturbations. A robust system neither increases nor decreases heritable phenotypic variation [5,6]. In the protein world, the phenotype is the structure and the phenotypic variance is given by slight variations in protein structure shape.

In organismal biology, the coordination of subunits within a whole (e.g. mammalian limb bones, floral and leaf traits, parts of wings, individual organs, etc.) has long been known as morphological integration [7], which was renamed by evolutionary developmental biologists as modularity ( [8], and references therein). The modularity of a system is a property that is closely related with both evolvability and robustness [6,9]. Such a property allows a system to increase its evolvability by diminishing adaptative constraints as well as giving the system the possibility for plasticity and the emergence of novel functions by rearranging the modules [9]. As stated in [8], integration and modularity concern the degree of covariation between parts of a structure. It is important, from an evolutionary viewpoint, to determine whether a structure is a single unit or consists of several modules. In molecular biology, the modularity of systems has been used to an extent, but more work has been done in systems biology [10-13] including analyses of metabolic networks [14-17], cell signaling networks [18-20], and protein interaction networks [21-25]. In the context of protein architecture, modularity has been used to refer to modules of exon shuffling [26,27], and complexes of enzymatic machineries [11]. Some approaches to protein structure modularity have also been explored [6,9,28] showing modules as domains [29,30] and also as sub-domain components [31-34]. However, the criterion to define protein modules depend on the definition of a proper quantitative treatment, which is not a trivial problem [9].

There have been different attempts to identify modules in protein structures [9] such as highly conserved close loops [35], foldons, and autonomous folding units [36]. Some of the aforementioned modules can only be identified experimentally and/or in single proteins. Another way, particularly robust, to perform modular decomposition is by using community detection algorithms [37] which have been applied extensively in system biology [10-25] as well as to the protein structure modularity identification problem [3,38]. However, most of these attempts only consider the contact matrix [3,38]. This approach bears no evolutionary information and depends exclusively in the definition of contact between residues [9]. We postulate that correlation information across a group of homologous structures (or a group of snapshots from a molecular dynamic simulation) is more relevant than molecular contact alone.

The analysis of graphs has become crucial to understand the features of different systems [39] such as community structure [40]. Several clustering algorithms have been developed (for a review on such algorithms see [39]) and applied successfully to different kinds of networks, such as networks of email messages [41], biological, and social networks [3,37,38,42]. However, all clustering techniques including the graph-based ones, lack a statistical framework to determine the significance of the inferred clusters. This may lead to results that may not be biologically meaningful. In this paper we present a graph theory-based clustering method that includes a test of statistical significance, a power test, and a test for the accuracy the estimates giving the sample size (i.e. bootstrap). To do this, we propose a permutation-based t-test to assess statistical significance, and power test based on [43] to assess the reliability of the estimates. We also propose a bootstrap test and a power analysis to infer cluster robustness. These tests are applied to coordinate data, but can be generalized to other applications. Here a module is defined as any group of residues that has significant correlation within the group (i.e. among residues within group) and such correlation within is significantly higher than the one obtained when correlating this residues with residues of other groups in the dataset. To assess performance and illustrate the method, a simulation with one correlating module against background noise and one with two modules were performed. We also analyzed two different kinds of protein structure data sets: Dynamic modularity from a molecular dynamics simulation of the Niemann-Pick C1 (NPC1) protein, and evolutionary modularity from the *α*-amylase homologs. The former protein is part of a complex of two proteins (NPC 1 and 2) required for the cholesterol to exit the lysosomes [44]. The NPC1 N-terminal domain binds to the cholesterol in an orientation opposite to NPC2. Mutations of NPC1 N-terminal domain are involved in the development of the NPC1 disease, an inherited disorder associated with lipid metabolism [44]. Therefore, it is important to know its dynamics in solution and identify probable sub-domain architecture that can be related with function. The latter dataset (*α*-amylase) is a digestive enzyme that, by acting at random locations along the starch chain, hydrolyses the *α*-1,4 bonds of larger polysaccharides yielding glucose and maltose [45]. It is a phylogenetically widespread type of hydrolases with multiple industrial uses [46]. These enzymes are multidomain proteins, but share a common catalytic domain in the form of a (*β*/*α*)_8_-barrel [47] which might give insight into fold evolution of the TIM-barrel, and the rise of its catalytic activity.

## Methods

Here we develop a method to explore the sub-domain architecture. To do so we proceeded as follows: 

1. Create test datasets: The input data (Section Input data) are cartesian coordinates of simulated data (Section Multivariate normal simulation) or coordinates of residues in the protein datasets (Sections Molecular dynamic simulation of NPC1 and *α*-amylase catalytic domain homologs).

2. Align the structures when needed: In the case of the simulations (both Multivariate normal simulation and molecular dynamics simulation) there is no need to align, since they all share the same plane, and absolute rotation (Section Structural alignment).

3. Extract the information as landmarks (Section Landmark definition) and residues contact matrix (Section Inter-residue contact definition).

4. Create a graph where each landmark is a node and they are connected if significant correlation is found (Section Graph construction).

5. Test if the partition of the data (grouping of residues) is statistically significant (Section Statistical significance test of clusters: controlling the false positives).

6. Test for statistical power of each partition (Section Statistical power test of clusters: acknowledging the false negatives probability).

7. Test for the stability of the partition to sample size: Bootstrapping (Section Bootstrapping: measuring the accuracy of sample estimates).

### Input data

#### Multivariate normal simulation

To test the method in known modular entities, two simulations were performed using Cholesky decomposition. First a multivariate normal random vector was generated as *Ly*, where *y* was a vector of independent *N*(0,1) variates. A matrix of correlated variables *LL*^*T*^ was created by Cholesky decomposition of the correlation matrix of the form:

$$\left(\begin{array}{cccccc} 1 & \cdots & \rho & 0 & \cdots & 0\\ \vdots & \ddots & \vdots & \vdots & \ddots & \vdots \\ \rho & \cdots & 1 & 0 & \cdots & 0\\ 0 & \cdots & 0 & 1 & \cdots & 0\\ \vdots & \ddots & \vdots & \vdots & \ddots & \vdots \\ 0 & \cdots & 0 & 0 & \cdots & 1 \end{array}\right)$$

The result is a matrix with a set of correlated variables (cluster), surrounded by random (uncorrelated) variables. Cluster intracorrelations ranged from 0 to 1 in increments of 0.05. The first 60 entries (accounting for a cluster with 30 elements with X and Y coordinates) had a given correlation, while 140 entries (accounting for 70 landmarks) where uncorrelated.

A simulation was also performed to evaluate the effectiveness in solving the boundaries of two modules. In that case, the correlation matrix was:

$$\left(\begin{array}{cccccc} 1 & \cdots & \rho_{1} & 0 & \cdots & 0\\ \vdots & \ddots & \vdots & \vdots & \ddots & \vdots \\ \rho_{1} & \cdots & 1 & 0 & \cdots & 0\\ 0 & \cdots & 0 & 1 & \cdots & \rho_{2}\\ \vdots & \ddots & \vdots & \vdots & \ddots & \vdots \\ 0 & \cdots & 0 & \rho_{2} & \cdots & 1 \end{array}\right)$$

The resulting matrix contains two cluster which intracorrelations ranged from 0 to 1 in increments of 0.05.

The output of the simulation is a set of coordinates for a given number of samples to which the method (explained in subsequent sections) will be applied.

#### Molecular dynamic simulation of NPC1

The Niemann-Pick, type C1 (NPC1; PDB code: 3GKH) N-terminal domain was simulated in solution with the ligand (cholesterol), using the software GROMACS 4 [48]. The force fields modes used for the simulations were OPLS-AA/L for the protein, and the TIP3P for the water molecules. The data was collected every two picoseconds for 10 nanoseconds discarding the first 2 nanoseconds of simulation. All other parameters where left as default. This process was performed using 100 CPU cores on a computer cluster, in triplicates. Four thousand samples where gathered and analyzed. The data includes the coordinates for each atom of each residue for the NPC1 protein simulated across snapshots of the simulation. This dataset was ensemble to test the method (explained in the subsequent sections) in dynamic data. That is, to find correlating residues for a protein in solution.

#### *α*-amylase catalytic domain homologs

In Homstrad database, the structures are manually curated guaranteeing the homology between them, and avoiding redundant structures [49]. However, the sample size is reduced with this curation (down to 24 structures in this data set). Here we used the structures in the Homstrad data set to fetch other structures with over 80% sequence identity available at the protein data bank ( http://www.rcsb.org/pdb/). With this procedure we increased our sampling, gathering 85 structures which PDB codes and species can be seen in Table 1.

**Table 1:** **PDB codes of the *α *-amylase homologs and the species from it was crystallized**

**PDB code**	**Species**	**PDB code**	**Species**
1A47.A	*T. thermosulfurigenes EM1*	1PIF.A	*S. scrofa*
1AMY.A	*H. vulgare*	1PJ9.A	*B. circulans*
1AQH.A	*P. haloplanctis*	1PPI.A	*S. scrofa*
1AQM.A	*P. haloplanctis*	1QHO.A	*B. stearothermophilus*
1AVA.A	*H. vulgare*	1QHP.A	*stearothermophilus*
1B0I.A	*P. haloplanctis*	1S46.A	*N. polysaccharea*
1BAG.A	*B. subtilis*	1SMD.A	*H. sapiens*
1BF2.A	*P. amyloderamosa*	1TMQ.A	*T. molitor*
1BLI.A	*B. licheniformis*	1U33.A	*H. sapiens*
1BSI.A	*H. sapiens*	1UA3.A	*S. scrofa*
1BVZ.A	*T. vulgaris R47*	1UA7.A	*B. subtilis*
1CDG.A	*B. circulans*	1UOK.A	*B. cereus*
1CGT.A	*B. circulans,s8*	1VB9.A	*T. vulgaris R47*
1CGU.A	*B. circulans,s8*	1VFM.A	*T. vulgaris R47*
1CIU.A	*T. thermosulfurigenes EM1*	1VFO.A	*T. vulgaris R47*
1CLV.A	*T. molitor*	1VIW.A	*T. molitor*
1CXE.A	*B. circulans*	1VJS.A	*B. licheniformis*
1CXF.A	*B. circulans*	1WZK.A	*T. vulgaris R47*
1CXK.A	*B. circulans*	1WZL.A	*T. vulgaris R47*
1CXL.A	*B. circulans*	1WZM.A	*T. vulgaris R47*
1CYG.A	*B. stearothermophilus*	1ZS2.A	*N. polysaccharea*
1D3C.A	*B. circulans*	2AAA.A	*A.niger*
1DHK.A	*S. scrofa*	2QMK.A	*H. sapiens*
1EH9.A	*S. solfataricus KM1*	2QV4.A	*H. sapiens*
1EHA.A	*S. solfataricus KM1*	2TAA.A	*A. oryzae*
1G5A.A	*N. polysaccharea*	3BAI.A	*H. sapiens*
1G94.A	*P. haloplanctis*	3BAJ.A	*H. sapiens*
1GCY.A	*P. stutzeri*	3BAW.A	*H. sapiens*
1GJU.A	*T. maritima*	3BMV.A	*T. thermosulfurigenes EM1*
1GJW.A	*T. maritima*	3BMW.A	*T. thermosulfurigenes EM1*
1HNY.A	*H. sapiens*	3CGT.A	*B. circulans,s8*
1HVX.A	*B. stearothermophilus B*	3L2L.A	*S. scrofa*
1HX0.A	*S. scrofa*	3L2M.A	*S. scrofa*
1JAE.A	*T. molitor*	3UEQ.A	*N. polysaccharea*
1JFH.A	*S. scrofa*	4CGT.A	*B. circulans,s8*
1JG9.A	*N. polysaccharea*	5CGT.A	*B. circulans,s8*
1JGI.A	*N. polysaccharea*	6CGT.A	*B. circulans,s8*
1JIB.A	*T. vulgaris R47*	6TAA.A	*A. oryzae*
1KCL.A	*B. circulans*	7CGT.A	*B. circulans,s8*
1MVY.A	*N. polysaccharea*	7TAA.A	*A. oryzae*
1OB0.A	*B. licheniformis*	8CGT.A	*B. circulans,s8*
1OSE.A	*S. scrofa*	9CGT.A	*B. circulans,s8*
1OT1.A	*B. circulans*		

Only the chain A, corresponding to the catalytic domain, was used.

### Structural alignment

The flexible structure aligner MATT with default parameters was used to align the structures and therefore deals with rotation, translation and natural deformations. This method allows local geometric flexibility for protein structures producing alignments with low root-mean-square deviations (RMSD), and estimating a p-value expressing the likelihood that a given alignment score can be generated by the alignment of unrelated proteins [50]. The multiple structure alignment outputted by MATT is then processed and analyzed as explain in the subsequent sections.

### Landmark definition

A landmark is a point, vertex, site or control point in a shape object (protein or simulation object in our case) that can be found repeatedly (and consistently) in a group of such objects [51]. Here we define a landmark as the centroid of homologous residues in a multiple structure alignment. The residue centroid is used to include both sequence (residue side chain) and geometry, as opposed to only the geometry of the backbone. To do this the coordinates of all heavy atoms (*A*) are taken into account.

### Inter-residue contact definition

Inter-residue contact maps are a widely used approach to analyze protein structures [52]. They are also important to understand protein folding and stability [53], and to identify residues playing structural and/or functional roles [52]. Despite this and the advances in the contact definition ( [52] and references therein), accurate contact map predictions are still mainly unsolved. There are some proposed tests [52] and software [54] but they are mainly using C_*α*_-C_*α*_ or C_*β*_-C_*β*_ distances with a threshold of about 7 to 8 Å [52,54]. However, these types of contacts are a mere approximation to true contacts. Here we defined a contact between any two residues if the distance between them is equal or less than 4.5 Å in an all-atom (all side chain atoms) contact analysis. The all-atom approach is more accurate since it takes into account the distance between each possible pair of atoms in two side chains.

### Graph construction

Assume that we have a dataset made of *n* observed protein structures (either homologous or sampled from a simulation in solution). For each of these structures, the input data matrix is composed of *k* landmarks. Here a landmark is defined as the Cartesian coordinates in three dimensions of the centroid of a residue. This centroid is calculated using the residue’s side-chains (see section Landmark definition). To deal with dimensionality, the original data matrix is split into its components (X, Y, Z) and, for each dimension, a correlation matrix between landmarks is computed. For each entry in each dimension, we test the significance of the correlation coefficient. This coefficient is set to 0 if it meets the following criteria:

(1) $$\frac{1}{2}\log\left(\frac{1+r}{1-r}\right)<\frac{Z_{\alpha }}{\sqrt{\left(n-3\right)}}$$

where the left-hand side of the equation 1 is the Fisher transformation of the estimated correlation *r*. The right-hand side of the equation 1 is the critical value for an alpha-level test of the null hypothesis that the correlation is 0. There, the *Z*_*α*_ is the standard score which allows us to calculate the probability of a value occurring within our normal distribution and compare scores from different distributions.

This step is done to simplify the graph building process such that insignificant correlations are ignored.

The overall magnitude of the correlation vector is calculated as:

(2) $$\Xi =\sqrt{\overset{3}{\underset{i}{\sum }}P_{i}^{2}}$$

where the value for the *i*th dimension, *P*_*i*_, is either *r* or 0 depending on the result in (1). The Ξ are obtained for each pair of landmarks and assign the edges of an undirected graph *S*, using the python-igraph library [55].

The summation in equation 2 is performed to agglomerate the dimensions (dimension reduction minimizing information loss). Since *r* is not additive and *r*^2^ is, the sum of *r*^2^ is the appropriate way to add the correlations without violating non-additivity. Also, Ξ is the correlation vector magnitude that guarantees that if there is any correlation in any of the dimensions, Ξ will include it, regardless of the vector direction. Let us assume that a given residue is highly and significantly correlated in the *X* axis, but poorly and/or not significantly correlated in *Y* or *Z* axes. Ξ will reflect such correlation since the residues must behave completely independent for Ξ to be zero or close to zero.

#### Graph abstraction

Let *S* = (*N*,*f*) be an undirected graph, where *N* is a list of nodes (landmarks), and *f* is a function f:N×N→k that assigns an edge weight to each landmark pair. An edge *E*_*ij*_ is assigned only if Ξ_*ij*_ > 0, *and if* the residues are in contact. The edge weight value is set to Ξ_*ij*_.

#### Community structure or clustering optimization

With the defined graph, the community structure is assessed using a fast-greedy approach, since it is an efficient way to detect clusters [40]. Clusters are defined by finding the partition of landmarks that maximizes the modularity index (*Q*) [56]:

(3) $$Q=\frac{1}{2m}\underset{\text{vw}}{\sum }\left[A_{\text{vw}}-\frac{\sum_{w}A_{\text{vw}}\sum_{v}A_{\text{vw}}}{2m}\right]\delta (C_{v},C_{w})$$

where *m* is the number of edges in the graph, *A*_*vw*_ represents the weight of the edge between vertices *v* and *w*, $\sum_{w}A_{\text{vw}}$ and $\sum_{v}A_{\text{vw}}$ are the weighted degree of a vertex (*v* or *w*), defined to be the sum of the edge weights of the adjacent edges for each vertex. *C*_*v*_ and *C*_*w*_ are communities to which the vectors *v* and *w* belong to, and the *δ* is a binary function where *δ*(*C*_*v*_,*C*_*w*_) is 1 if *C*_*v*_ = *C*_*w*_ and 0 otherwise.

The modularity index (*Q*) is then the proportion of edges shared within groups minus the expected proportion if edges were distributed at random. For a given partition, *Q* indicates the density of nodes within groups when compared against a random distribution of connections regardless the partition. *Q* ranges between -1 and 1. If positive, there are more connections inside the module than expected by chance and therefore a possible community structure [56,57] (i.e. partition or clustering of the data). In our case, a partition made by the optimization of *Q* is a group of residues that correlate in space (i.e. move together) given the sample. If the sample is across homologous proteins, the cluster or partition represents a concerted movement in the evolution of the protein. Sampling across molecular dynamic simulation snapshots represents parts of the protein that are moving together in solution.

The output is a membership vector that corresponds to the community structure (partition or clustering) in the graph of landmarks. It is interpreted as a set of clusters which number is given by the optimization procedure and therefore there is no need for an a priori determination of the number of clusters to be obtained. Each cluster is assumed to be a putative module but this membership vector provides no support or information about its statistical robustness and significance.

### Statistical significance test of clusters: controlling the false positives

Despite the usefulness and ubiquity of tests using similar algorithms, the question of significance of clusters is critical since there is no support for the obtained clusters, and therefore its validity is questionable. To test if each cluster is significant, a permutation t-test [58] (as implemented in R [59,60]) is applied.

The rationale for the test is based on the definition of cluster as an entity where the distribution of correlation of the elements inside the cluster (*intracorrelation*) is significantly distinct from the distribution of correlation with elements from other clusters (*intercorrelation*). This test is applied for each possible pair of clusters defined by a membership vector. For a given pair of clusters, we compare the distribution of the intracorrelation for that cluster with the distribution of intercorrelations for this pair. If one cluster is artificially broken down by the clustering algorithm, there should be no significant differences between the distribution of intra and inter-correlations.

Because the test is performed for a number of pairs, multiple comparisons are made. Let *M*(*A*) and *M*(*B*) be the mean intracorrelations for two clusters *A* and *B*, found by the community detection algorithm. Let *M*(*AB*) be the mean intercorrelation. The null hypothesis we test is *H*_0_ : *M*(*A*) = *M*(*AB*). With more than two clusters the number of comparisons (*K*_*C*_) will be *K*(*K* - 1), *K* being the number of clusters. If a single-inference procedure is used, this can result in an false increased significance which we correct for using the Benjamini-Hochberg False Discovery Rate correction (FDRc) procedure [61].

For example, a given set of homologous proteins is analyzed with our method and a possible partition is obtained. This will give us different pieces of the protein that correspond to groups of residues that are correlating (moving together) more within each cluster than among clusters. We use the correlations inside a given group and test against the correlation that exist between that group and other groups. If there is no significant difference, both entities are moving together and therefore should be merged.

#### Refinement of the membership vector

The results of the significance testing are summarized into a new graph. Let graph *S* = (*C*,*E*) be a directed graph, where *C* is a list of inferred clusters, and *E* a list of assigned edges. There will be a directed edge from cluster *C*_*u*_ to cluster *C*_*v*_ if the hypothesis that *M*(*u*) is distinct from *M*(*uv*) cannot be rejected. If *C*_*u*_ and *C*_*v*_ are connected by a bi-directional edge, they are merged into a single cluster. The process is iterated until no clusters can be merged.

Following the example in the previous section, let’s assume that the protein dataset analyzed was partitioned into 4 groups of residues (*A*, *B*, *C*, and *D*). Each of those groups will be the vertices (nodes) in a new graph. We will draw an arrow if there is no significant differences between a given group and other (e.g. correlations within *A* are not significantly different than the correlations between residues in *A* and residues in *B*). If this is reciprocal (e.g. correlations within *B* are not significantly different than the correlations between residues in *B* and residues in *A*), both groups of residues are merged.

### Statistical power test of clusters: acknowledging the false negatives probability

The above statistical test assesses False positives (Type I error). It is important as well to assess the strength of association between members of a cluster. To determine the minimum resolvable correlation for a given sample size, and for a given significance and power, let *ρ*_*res*_ be the correlation that can be resolved with a power of 1-*β*, and a significance level of *α* given the number of observations *n*, as suggested by [43] and implemented in the R package "PWR" [59,62]. Let *γ* be a function of *i* and *j*:

(4) $$\gamma (i,j)=\left\{\begin{array}{ll} 1 & \text{if}\;r_{\text{ij}}\geq \rho_{\text{res}}\\ 0 & \text{Otherwise} \end{array}\right)$$

where *r*_*i*,*j*_ is the correlation coefficient between landmarks *i* and *j*. To assess the power of a candidate cluster *C* with *c* elements, we estimate the proportion of correlation values between landmarks of *C* that are larger than *ρ*_*res*_. For each *C* the proportion of variables with enough power (PVP) is thus:

(5) $$\text{PV}\;P_{C}=2\left(\frac{\sum_{1}^{p}\gamma (i,j)}{\left(c^{2}-c\right)}\right)$$

where *p* is the number of pairs *i*,*j* in cluster *C*.

**Figure 1:**
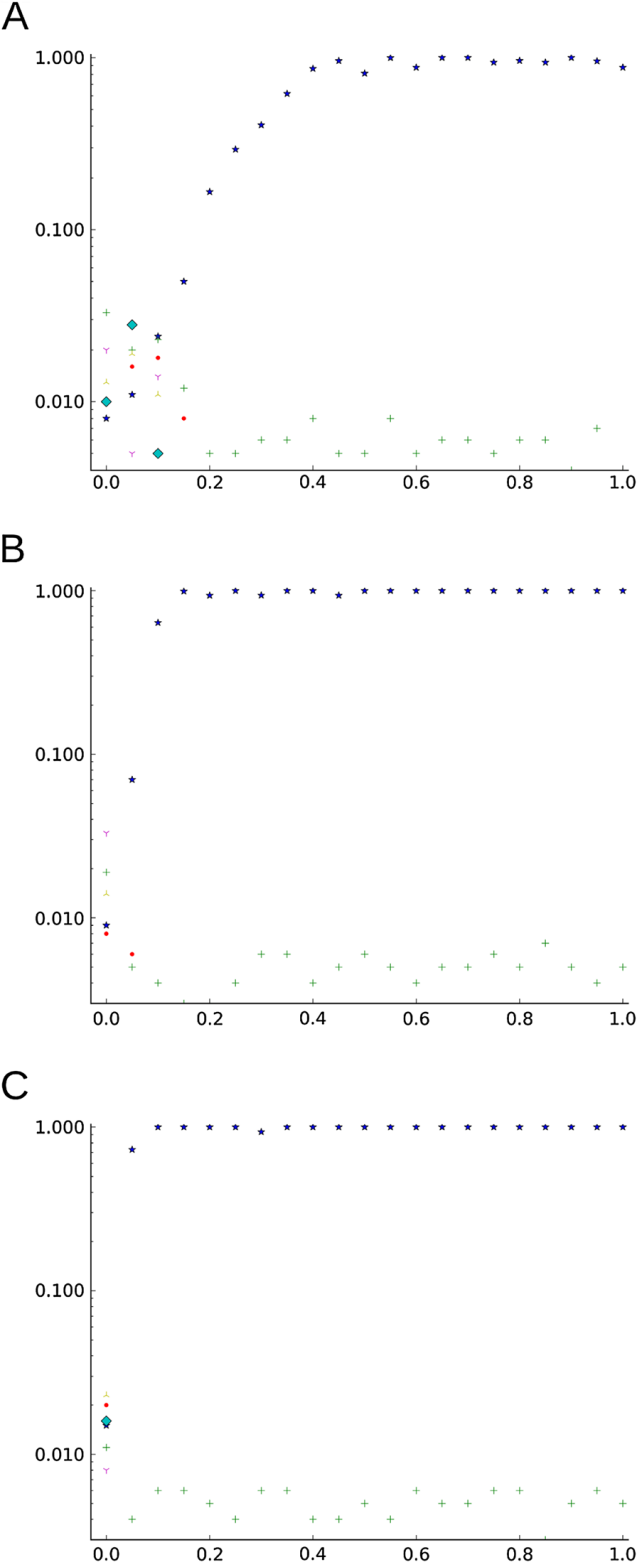
**Behavior of the estimated PVP in different sample sizes.** PVP values (y axis) against intracorrelations (x axis) for the simulated data. Simulations were run with 85 observations **(A)**, 1000 observations **(B)**, and 5000 observations **(C)**. The star represents the true cluster, the cross represents the grouped singletons, and the rest of the markers represent other singleton clusters recovered as false positives (and therefore low PVP values).

Here *PV* *P*_*C*_ is the estimated PVP which should be distinguished from the true PVP, that arises when the estimated *r*_*ij*_ in equation 4 are replaced by the actual *ρ*_*ij*_. *PV* *P*_*C*_ provides a qualitative information to help interpret the results given the used sample size. Figure 1 shows the behavior of the PVP in the intracorrelations evaluated for 85 (Figure 1A), 1000 (Figure 1B), and 5000 (Figure 1C) observations. Even in simulated data, PVP deviates from the possible values of 0.0 and 1.0 when the number of observations is small.

For instance, take a cluster (group of residues from the previous example) *A* that contains 10 elements, and 45 entries in the upper triangle of its correlation matrix. Assume that *A* was inferred with 100 observations (protein structures from the example). With that sample size, *ρ*_*res*_ will be approximately 0.28 with a power of 0.8 and a significance level of 0.05. If two thirds of the entries in the upper triangle of the correlation matrix of *A* are below *ρ*_*res*_, *PVP*_*A*_ will be equal to 0.66. In other words, for 30 entries of the correlation matrix we estimate that there was a power of 0.8 or greater. If there are clusters created by optimizing the modularity score using weakly correlated landmarks, this cluster’s PVPs will tend to be close to 0. This test is post-hoc, and is only to inform about the robustness of the partition created.

### Bootstrapping: measuring the accuracy of sample estimates

**Figure 2:**
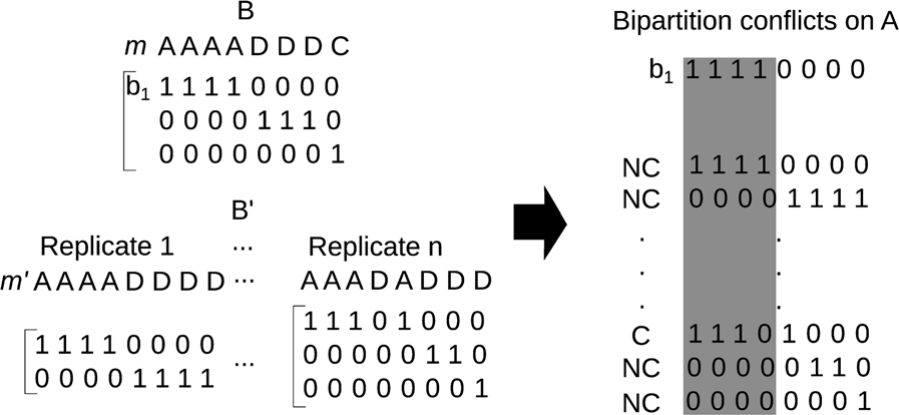
**Bootstrapping: Defining conflicts.** After the clustering method is applied to a given data set, a membership vector *m* is inferred (3 clusters, *A*, *D*, *C*, in this example) from which a set of bipartitions *B* can be deduced. By re-sampling with replacement (bootstrapping) the data set, we obtain *n* replicates which give us a set of bipartitions *B*^′^ (left-hand side). When a single bipartition from *B* is compared against *B*^′^, a conflict (C) or non-conflict (NC) is inferred (right-hand side). This procedure is repeated for all clusters in the original *m*, giving support for to each cluster as the percentage of non-conflicts for that cluster.

The previous tests evaluate the probability of false positives (Type I errors) and false negatives (Type II errors). However, the sensitivity to sampling error in each estimated cluster can be tested using bootstrapping techniques. The clusters for any set of *n* samples can be represented as a set of *K* bipartitions, $b_{1},\;\ldots ,\;b_{K}$, where *b*_*ji*_ = 1 or 0 according to whether the *i*th landmark was in the *j* cluster or not. The bootstrap approach repeatedly generates sets of *n* samples with replacement from the original data. For each of these sets of *n* samples, we obtain a membership vector as with the original data. All of the bipartitions from all bootstrap sets are then aggregated. The bootstrap percentage for an inferred cluster in the original dataset is calculated as the proportion of bipartitions in the aggregate set showing no conflicts with that cluster. This proportion is reported as the bootstrap value which evaluates the cluster’s robustness (Figure 2). From the example, 100 protein structures correspond to the original data from which we have the bipartitions (as shown in Figure 2). We create N new replicates by sampling the original data. In some occasions, the same protein structure will be picked. The bootstrap resampling evaluates the effect of possible missing data.

All methods described here were performed with original python scripts (otherwise stated), available upon request to the authors and licensed under a GPL agreement.

## Results and discussion

In this section we present the outcome of two simulations to validate the method. The parameters for the simulations can be found in the Methods section. We also present two real datasets. First a set of snapshots from a molecular dynamics simulation (MDS) of the NPC1 N-terminal domain are analyzed to provide insights into the modular architecture of dynamic data. That is, group of residues that move together in solution.

Then a set of homologous structures of the *alpha*-amylase catalytic domain are use to test the sub-domain architecture at the evolutionary level. A module here (diferent than the MDS modules) refers to a group of residues that are moving together in the evolution of the structure.

Most biological data are typically highly multivariate and multidimensional in nature. Many tools have been developed to deal with such dimensionality ( [63] and references therein). However, the variable selection and dimensionality reduction used in such methods (aiming to reduce matrix complexity) may compromise information conservation [64], or require a larger sample size than is possible for protein data. To overcome these drawbacks, we introduce a community detection-based clustering method. Community detection-based approaches do not need a priori knowledge of the number of clusters [65], are not heavily parametrized, and can handle multivariate and multidimensional data without dimensionality reduction. Here we propose a graph based method to explore protein structure modularity, where: 

1. A graph is built where the vertices are the centroids of residues. The correlation between coordinates is set as edge weight if it is significant (see equations 1 and 2), and if the two residues are in contact (See Contact definition in Methods).

2. The community structure in the graph is inferred by fast-greedy (evaluating and selecting the best result at each step, as opposed to maximizing at the end of the scoring process) optimization of a modularity score (Q; see equation 3).

3. The statistical support for each cluster is obtained.

4. The solution is refined based on this statistical support.

5. The statistical power to resolve each partition with respect to the size of the dataset is estimated (equations 4 and 5).

6. The stability of the estimates with respect to the sampling error is measured using bootstrapping (Figure 2).

Finally, we present the results of the analysis of two protein data sets: a molecular dynamics simulation of the NPC1 protein, and a multiple structure alignment of the *α*-amylase catalytic domain homologs.

### Simulations

Correlated landmark data was simulated using a multivariate normal simulation. Intracorrelations ranged from 0 to 1 in 0.05 increments. The parameters to make the simulations are explained in the Methods section.

#### Estimated correlations: precision of the simulations

Accurate estimates of correlations were gathered for the simulation performed. However, precision varied substantially with sample size. Despite this, even with low sample sizes the median correlations were close to the true values (Tables 2 and 3). As can be seen, some variance was allowed to make the simulation more realistic.

**Table 2:** Precision of the simulations with one module in background noise

**Sample**	**Intra-cluster**	**Intracorrelation quantiles**					**Background correlation quantiles**				
**Size**	**correlation**	**0%**	**25%**	**50%**	**75%**	**100%**	**0%**	**25%**	**50%**	**75%**	**100%**
100	0.2	-0.087	0.145	0.206	0.274	0.545	-0.348	-0.058	0.009	0.077	0.304
	0.4	0.061	0.288	0.341	0.395	0.589	-0.314	-0.075	-0.0064	0.059	0.331
	0.6	0.334	0.542	0.578	0.619	0.765	-0.361	-0.072	-0.002	0.066	0.296
	0.8	0.651	0.771	0.791	0.811	0.875	-0.365	-0.073	0.002	0.067	0.291
	1.0	1.0	1.0	1.0	1.0	1.0	-0.209	-0.069	-0.019	0.051	0.232
	0.2	0.061	0.168	0.193	0.220	0.354	-0.169	-0.029	0.001	0.029	0.139
	0.4	0.261	0.365	0.389	0.411	0.494	-0.172	-0.029	0.002	0.0332	0.204
500	0.6	0.485	0.562	0.580	0.597	0.656	-0.152	-0.031	2 × 10^-5^	0.031	0.149
	0.8	0.743	0.776	0.786	0.795	0.824	-0.161	-0.041	-0.011	0.019	0.144
	1.0	1.0	1.0	1.0	1.0	1.0	-0.142	-0.0350	1.7 × 10^-4^	0.029	0.087
	0.2	0.087	0.170	0.190	0.209	0.308	-0.131	-0.021	7.2 × 10^-4^	0.023	0.116
	0.4	0.338	0.391	0.407	0.422	0.4745	-0.108	-0.016	0.005	0.0267	0.126
1000	0.6	0.535	0.580	0.591	0.603	0.647	-0.103	-0.018	0.002	0.023	0.129
	0.8	0.759	0.791	0.798	0.804	0.827	-0.131	-0.024	-0.005	0.012	0.083
	1.0	1.0	1.0	1.0	1.0	1.0	-0.038	-0.014	0.009	0.024	0.068

Precision of the simulation of one module on background noise with 100, 500, and 1000 samples. The quantiles describe the distribution of values in the lower triangle of the correlation matrix for the full simulated data set. The background quantiles represent the distribution of values in the rest of the matrix as background noise.

**Table 3:** Precision of the simulations with two modules

**Sample**	**Intra-cluster**	**Intracorrelation quantiles**					**Intercorrelation quantiles**				
**Size**	**correlation**	**0%**	**25%**	**50%**	**75%**	**100%**	**0%**	**25%**	**50%**	**75%**	**100%**
	0.2	-0.12	0.112	0.178	0.247	0.532	-0.349	-0.061	0.009	0.081	0.357
	0.4	0.135	0.347	0.401	0.451	0.627	-0.372	-0.075	-0.014	0.049	0.314
100	0.6	0.405	0.581	0.617	0.653	0.758	-0.468	-0.225	-0.165	-0.104	0.116
	0.8	0.716	0.776	0.795	0.813	0.879	-0.256	-0.087	-0.049	-0.011	0.147
	1.0	1.0	1.0	1.0	1.0	1.0	-0.018	-0.018	-0.018	-0.018	-0.018
	0.2	0.059	0.172	0.2	0.229	0.337	-0.177	-0.036	-0.008	0.023	0.151
	0.4	0.286	0.371	0.393	0.414	0.495	-0.099	0.013	0.04	0.066	0.183
500	0.6	0.516	0.587	0.602	0.618	0.67	-0.099	0.005	0.029	0.051	0.153
	0.8	0.748	0.793	0.801	0.808	0.838	-0.069	0.014	0.031	0.05	0.131
	1.0	1.0	1.0	1.0	1.0	1.0	-0.012	-0.012	-0.012	-0.012	-0.012
	0.2	0.109	0.182	0.201	0.221	0.301	-0.116	-0.023	-0.001	0.021	0.128
	0.4	0.321	0.388	0.405	0.421	0.488	-0.133	-0.03	-0.011	0.008	0.089
1000	0.6	0.531	0.582	0.593	0.605	0.647	-0.077	-0.008	0.008	0.023	0.091
	0.8	0.764	0.793	0.799	0.805	0.825	-0.052	0.007	0.021	0.034	0.082
	1.0	1.0	1.0	1.0	1.0	1.0	0.026	0.026	0.026	0.026	0.026

Precision of the simulation of two modules with 100, 500 and 1000 samples. The quantiles describe the distribution of values in the lower triangle of the correlation matrix for the full simulated data set. The intercorrelation quantiles represent the distribution of the sub-matrix corresponding to the correlation between the two modules.

#### Performance of the the method

**Figure 3:**
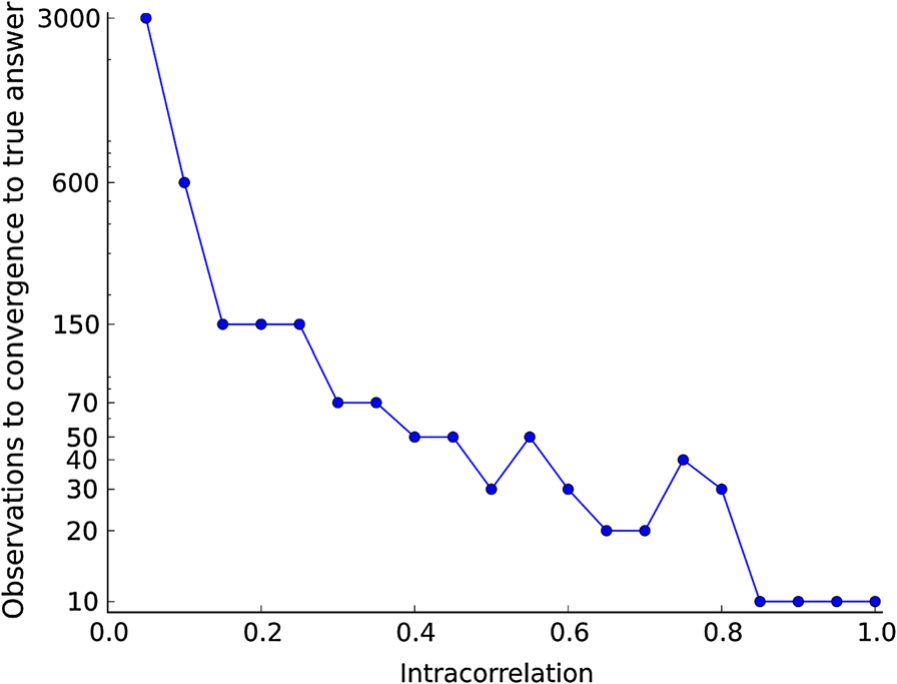
**Performance of the method in simulated data.** Performance of the clustering method by number of observations and intracorrelation in one module over background noise. The Y-axis represents the number of observations when there is convergence to the true answer (1 cluster) for each intracorrelation evaluated.

In noisy data, our method is able to correctly identify and assign the membership vector at very low modular intracorrelations (Figure 3) when the sample size is sufficient. Even for intracorrelations as low as 0.05, if provided with more than 3000 observations the method identifies the true cluster.

Table 4 shows the results of the significance tests, power analysis, and bootstrapping. The significance test controls the Type I error and therefore the false positives. Here it is reported for an *α* (False positives or Type I error probability) of 0.05. However, the permutation test is not able to deal with the false negatives or Type II error (Table 4).

**Table 4:** Clusters, significance, PVP and Bootstrap support for the simulated data

**Clusters**	**Significance**	**PVP**	**Bootstrap**
**One module on background noise**			
A	< 0.0375	0.617	93%
Singletons	0.134	0.006	9%
**Two modules**			
A	< 0.0125	0.631	100%
B	< 0.0125	0.640	100%

The proportion of pairs that were judged not to be in the same cluster after the permutation test (Significance), the estimated PVP for the simulated data set and the bootstrap value in the simulated datasets with 0.35 intracorrelation and 85 observations. PVP is the proportion of variables in the cluster with enough power (with a *β* of 0.2 and an *α* of 0.05) to be resolved. The significance test critical value was corrected using the False Discovery Rate correction.

In simulations with correlations of 0.35, the method was able to identify the "true" cluster (Table 4) with a relatively low number of observations (85 in this case), and the power analysis gives an estimation of robustness. In the simulation, only cluster A has enough power (0.617 estimated PVP) to resolve almost two thirds of the components of the cluster. The other "module" is a collection of singletons which has an estimated PVP close to zero. Similarly, the bootstrap value highly supports the "true" cluster, while the group of singletons is ruled out. Here we show that the significance test efficiently deals with false positives, the PVP gives information about the strength of clustering, and the bootstrap gives information about the repeatability of the clusters when re-sampling.

### Protein data sets

The coordinate data was collected using two different strategies. The structure of a protein was simulated using molecular dynamics (MD) to produce molecular motion over time. From these simulations, snapshots of atomic coordinates were captured. Sites that move together over time are expected to have correlating centroid coordinates. Therefore any module inferred from this kind of data indicates the mechanistic component of the protein structure in solution. The Niemann-Pick, type C1 (NPC1; PDB code: 3GKH) N-terminal domain was simulated in solution, using the software GROMACS 4 [48]. All the parameters for the MD simulation are explained in the Methods section.

The second type of data is based on homology. In this case, homologous structures are aligned. The centroid coordinates of sites that are packed together and interacting across the evolutionary samples are expected to correlate. A module inferred from this type of data indicates that a defined subset of the protein structure is evolving as a unit. The *α*-amylase catalytic domain dataset from the Homstrad database [49] was used to assess clustering in an evolutionary perspective. The structures used, the sampling strategy, and the alignment method used are explained further in the Methods section.

#### Dynamic modules of the Niemann Pick C1 protein N-terminal domain

**Figure 4:**
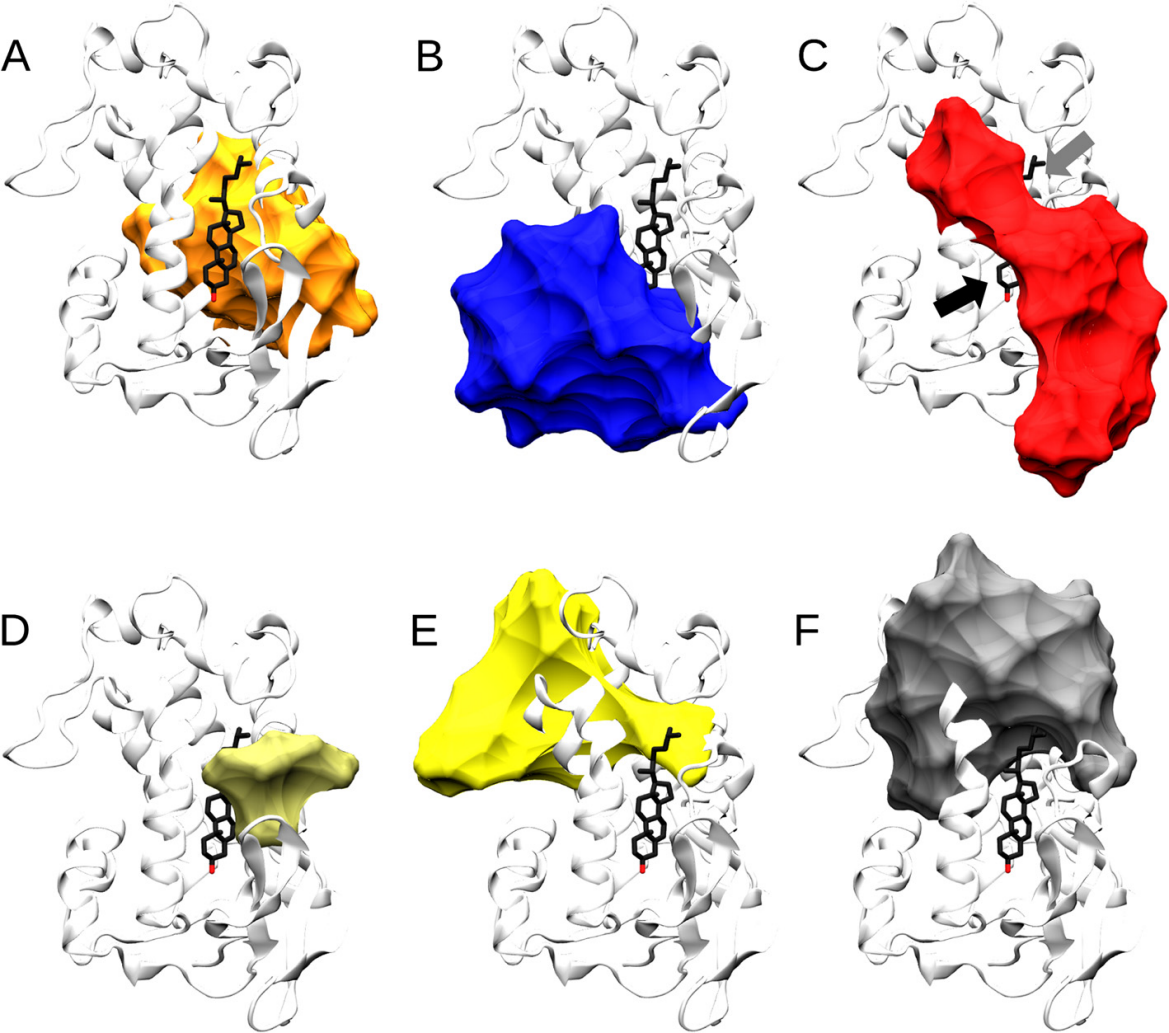
**Dynamic modules in the Niemann-Pick disease, type C1 protein.** Modules recovered by the method in a molecular dynamics simulation of the The Niemann-Pick disease, type C1 (NPC1; PDB code: 3GKH) protein with cholesterol (Licorice-type structure) bound. The arrows in sub-figure C point to the water (black arrow) and sterol (gray arrow) openings, described in [44]. The list of the equivalences of residues in each module can be seen in Additional file 1 (S86, p. 579). The images were rendered using VMD [71] and POVray ( http://www.povray.org). Panels **A**-**F** show the individual modules inferred.

The Niemann-Pick disease type C (NPC) is an autosomal recessive disease, expressed when there is an error in the exogenous cholesterol trafficking and as result a lysosomal accumulation of it [66]. This disease is caused by a mutation in either of the two NPC proteins (NPC1 and NPC2) [44]. The Niemann-Pick C1 (NPC1) protein regulates the lysosomal cholesterol transport to other intracellular compartments [67]. NPC1 contains 13 (13-16 according to [66]) membrane domains and 3 other domains that are in the lumen of the lysosomes [68]. One of these luminal domains is the N-terminal domain which bears the cholesterol binding site [69], and has eight *α*-helices flanked by three *β*-sheets (Figure 4) and its sequence is highly conserved [70]. NPC1 N-terminal domain (unlike the NPC2 protein) can bind with the oxygenated derivatives of the cholesterol [44] making it an interesting domain to study dynamic properties.

Figure 4 shows the modules gathered when the module identification is applied to the molecular dynamics simulation of the NPC1 N-terminal domain snapshots. All these modules showed a bootstrap above 66.7% and a PVP over 0.96. Interestingly, all modules are related with the binding pocket, surrounding the cholesterol molecule.

The first module (Figure 4A) encloses three cholesterol binding residues, and another binding residue to the N-Acetyl-D-Glucosamine (NAG). It also spans a residue associated with the development of the NPC1 disease in adulthood [72]. All other residues correlating with these seem to give structural support to the back of the cholesterol binding pocket, as well as serving as receptacles for both ligands. This region also encompasses four residues containing single nucleotide polymorphisms (SNPs) for the human gene [73].

In Figure 4B, a module that comprises more than half of the residues that make the sterol pocket is shown. From these residues, this module is the only one that includes the non-hydrophobic ones, being of importance in the direct protein - 3 *β*-hydroxyl interactions, as well as the water-mediated interaction with such groups. This helps in the stabilization of the bounded sterols and giving a particular stereo-specificity [44]. This module is located in the bottom part of the binding pocket and can be seen as a "glue" for the secondary structures in contact with such pockets, and therefore maintaining the shape of the structure in its less movable part. This also supports the model in [44], where the sterol opening needs to move in order to uptake the cholesterol from NPC2. This module also contains three SNPs found in the human gene [73].

The module shown in Figure 4C, shows the residues responsible for the water (black arrow) and sterol (gray arrow) openings described in [44] as being of functional importance to the cholesterol uptake and the retention of it in the binding site. If some residues within this module are mutated, the cholesterol might not be taken by the protein and the Niemann-Pick disease is expressed [44]. This module also includes two cholesterol binding sites, a residue shown to be related to the development of the disease in infantile stages [74], and a SNP [73].

The module in Figure 4D shows a small module that coincides with functionally important residues involved in the affinity for cholesterol binding [44]. These thus may be related to the expression of the NPC disease. Giving that these modules are analyzed in the light of dynamics, the module in Figure 4D shows that the affinity for the cholesterol mediated by these residues is given by geometric constraints induced by cholesterol binding.

Figure 4E shows a module that encloses two binding residues to NAG. It has also been shown that two residues are important in the development of a late infantile NPC1 disease [72,74], and one SNP is also enclosed. It seems to be also of structural support for the cholesterol binding pocket in the top(E), creating a pocket that receives the ligand.

The module shown in Figure 4F encloses the *α*-helices 3, 7 and 8, that have been shown to play an important role in the access and release of cholesterol, since its movement controls the enlargement of the sterol opening [44]. This module also contains some of the residues that decrease the cholesterol transfer to the liposomes if mutated [44], as well as four SNPs [73]. The module shown in Figure 4F is therefore of functional importance for the intake and outtake of cholesterol.

Since there are disease-related mutations in all of the modules, it would be important to further study the relationship between modules and protein function. The correlation within modules is large enough to think of them as units, and therefore it is probable that the residues exposed in [44,73,74] are not the only major contributors to the disease. Further confirmation of the effects of mutations within these modules is needed.

#### Evolutionary modules in the *α*-amylase catalytic domain

Starch is the main storage of carbohydrates in plants. Processing it and discovering novel poly and oligosaccharides is important for biotechnological and chemo industrial applications [75]. Most starch-related enzymes are classified within the *α*-amylase family. This family catalyzes the hydrolysis of *α*-(1,4) glycosidic bonds of polysaccharides, and therefore is classified as glycoside hydrolases [76]. This a multi-reaction catalytic family, since its members can catalyze different reactions (hydrolysis, transglycosylation, condensation and cyclization) [77]. Industrially, some *α*-amylases are used in the production of ethanol [78], high-fructose corn syrup [79], and other oligosaccharides. It is therefore of industrial and biological importance. It has a highly symmetrical TIM-barrel ((*β*/*α*)_8_) catalytic domain [75]. This fold is highly versatile and widespread among the structurally characterized enzymes, being present in almost 10% of them [80-83]. There has been a debate about the type of evolution that this fold has been through: convergent, divergent, or both [80]. However, there is evidence supporting the divergent evolution hypothesis [81]. The catalytic activity and substrate binding residues occur at the C-termini of *β*-strands and in loops that extend from these strands [75].

**Figure 5:**
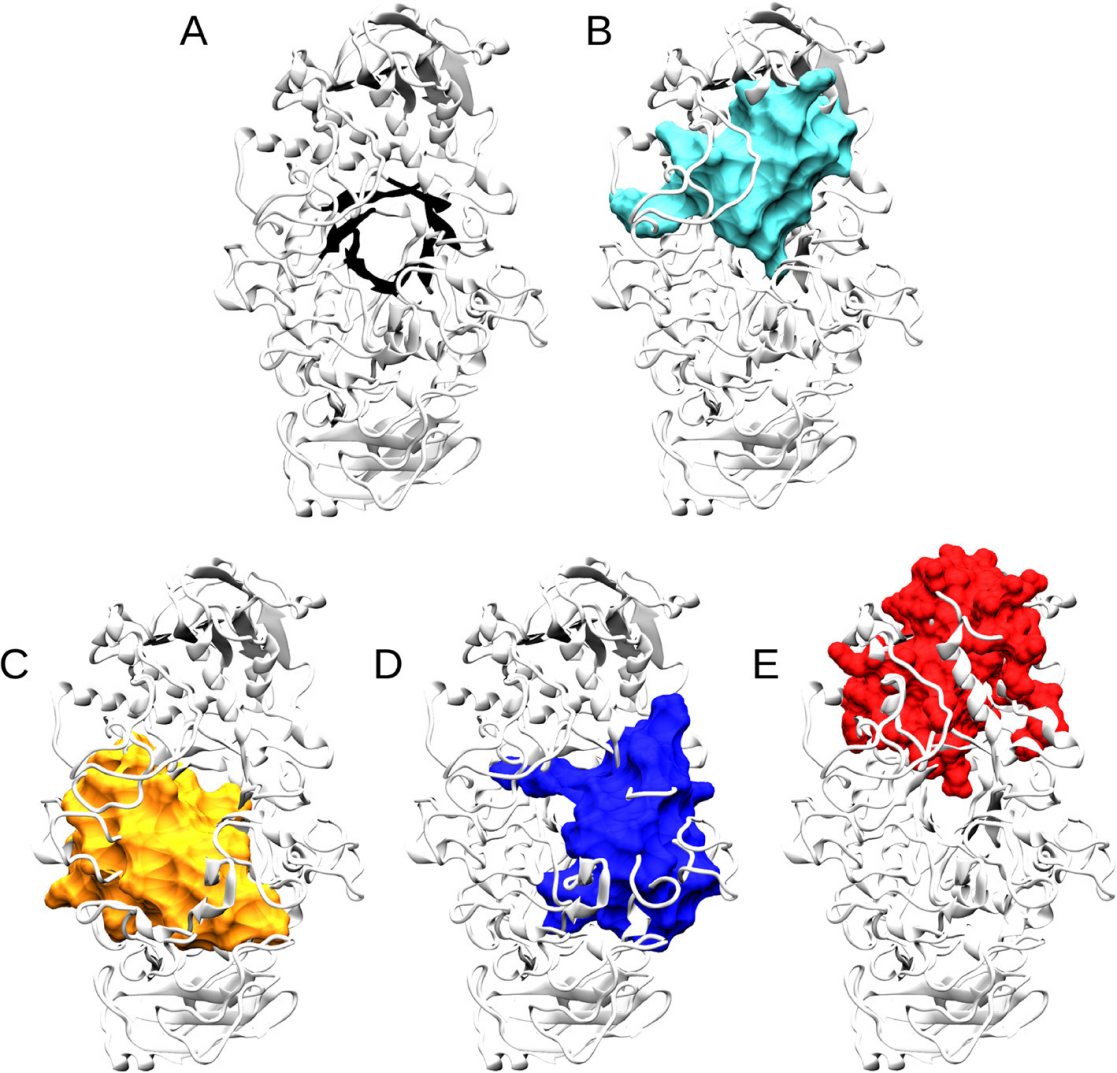
**Evolutionary modules in** ***α*-amylase homologs.** Modules recovered by the method in the homologous structures of the *α*-amylase, its catalytic domain. This analysis was performed using the 85 redundant structures available at the protein data bank ( http://www.rcsb.org/pdb/). In the sub-figure **A** the TIM barrel is highlighted, and sub-figures **B**-**E** show the different modules obtained. The full list of PDB codes is available in the Table 1. The list of the equivalences of residues in each module in each structure can be seen in Additional file 1 (S36, p.241).The images were rendered using VMD [71] and POVray ( http://www.povray.org). The structure used to visualize the modules is the PDB 1BF2.A from *P. amyloderamosa*.

**Figure 6:**
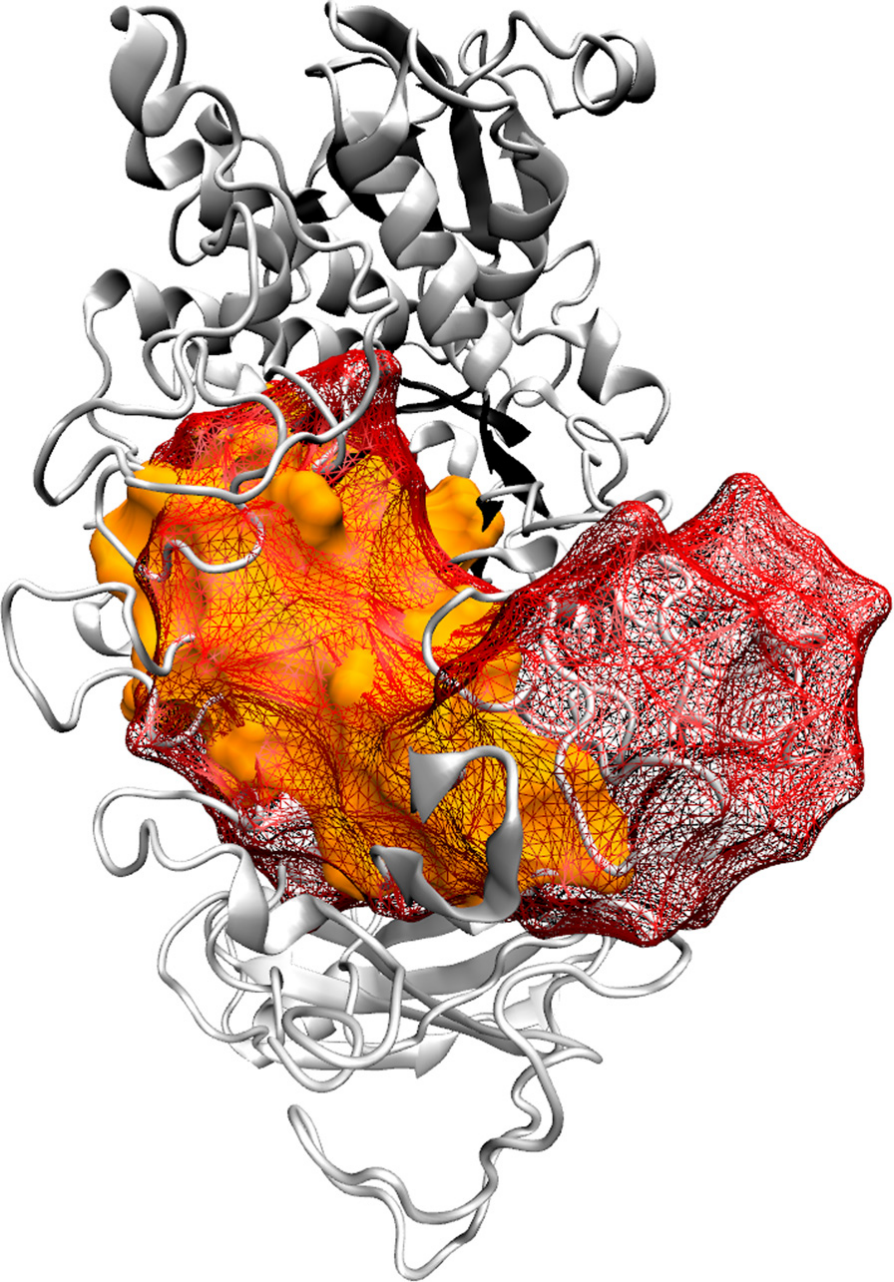
**Evolutionary module in *α *-amylase spanning the smallest active sub-domain of a TIM-barrel fold.** Expansion of the module shown in Figure 5C with a superimposition of the AmyTM structure reported in [85] (wire-frame structure). The images were rendered using VMD [71] and POVray ( http://www.povray.org).

Four modules are identified in the *α*-amylase sub-domain architecture (Table 5 and Figure 5). In Figure 5, most of the modules span the surface to the TIM-barrel (*β*-sheets of the TIM-barrel are highlighted in Figure 5A). This behavior is due to the interaction of the protein and its catalytic pocket, with the ions calcium and sodium received by this structure mainly on its surface. Modules shown in Figures 5B, D and E span regions where these ions are frequently found among the homologs, and the residues in charge of the ligation of the three metal ions [84] as co-factors for the hydrolysis. The module in Figure 5B also comprises two residues that mutational studies have shown as important for the cleavage site [47] and have been reported as substrate binding and catalytic residues. The module shown in Figure 5D also spans important catalytic residues. This includes a proton donor, a catalytic nucleophile [47], and five substrate binding residues [75]. The module in Figure 5C comprises a substrate binding residue [75]. Furthermore, module 5C seem to span most of the smallest active sub-domain of a TIM-barrel fold, as shown by [85] in *Bacillus stearothermophilus*, comprising almost all of the *β*_2_*α*_2_ domain (Figure 6). No known catalytic residue was found in this module, however [85] showed that this module retains its catalytic activity. The second domain showed by [85] (Figure 6), was not homologous throughout our sampling (i.e. was not present in all the sampled structures), and therefore, no information was available about this domain.

**Table 5:** **Clusters, significance, PVP and Bootstrap support for the *α*-amylase data set**

**Modules**	**Significance**	**PVP**	**Bootstrap**
B	< 0.001	0.479	31.3%
C	0.005	0.440	42.9%
D	< 0.001	0.503	39.9%
E	< 0.001	0.580	51.7%

The proportion of pairs that were judged not to be in the same cluster after the permutation test (Significance), the estimated PVP and the bootstrap value in the *α*-amylase. The significance test critical value was corrected using the False Discovery Rate correction.

### Putative meaning of the sub-domain architecture

So far we have shown the significant partitions of a domain. But what is the probable meaning of such modules? One might think that this modules can represent autonomous folding units (AFU), however our data show discontinuous amino acid sequences (in one dimension, since they are in contact in 3D space) per module. Also, comparative analysis with the dataset analyzed by [86] showed no relationship with our grouping (Data not shown). Another plausible hypothesis could be assign modules to close loops, but the same continuity argument can be brought upon. Furthermore, the *α*-amylase subdomains identified by our method span several of the TIM-barrel close loops exposed by [87] with no particular pattern. This discrepancies are expected, since the definition of foldons, AUFs and close loops have little or no meaning in an evolutionary perspective. These concepts are derived from the analysis of single structures and their internal interactions (i.e. contact matrix, physical interactions, length, distance) and therefore the non-evolutionary approaches for sub-domain determination will identify a different kind of module than an evolutionary approach.

On a more related framework, [88] developed a method to test co-evolving sites. When tested on the *α*-amylase dataset used in this article, no pattern correlating the two methods were found (Data not shown). Moreover, the largest significant grouping of co-evolving residues with [88] method span only 10 residues of the protein. This discrepancy can be attributed to the fact that [88] are testing co-evolution in a sequence based perspective. That is, giving a phylogenetic tree and its source alignment, which residues have significant mutual information. This method disregard completely the geometry of protein structures, therefore answering a different question than our approach.

So what is the possible meaning of our sub-domains? Despite more work (both bioinformatic and experimental) is needed to clearly address this question, the sub-domain architecture here represented is probably co-evolving geometric units (in the case of homologous sampling) and semi-rigid components (in the dynamic perspective) of proteins.

## Conclusions

Protein structures have a modular architecture. Such modularity can be seen as hierarchical because there are different degrees of integration among its modules. The domain architecture has been shown to harbor evolutionary and structural coherence [9,29,38,89-92]. However, there is also evidence of a sub-domain architecture of protein structures that can drive protein structure evolution. Here we introduced a robust and significant way to identify such sub-domain architecture, giving information about the result’s power with a finite sample size number, providing ways of assessing the significance for clustering and the strength of correlations within clusters. With enough sampling the method correctly and confidently identifies modules with an intracorrelation as low as 0.05 (nearly random) for simulated data. In real datasets our method is able to capture functional, structural, and evolutionary information, returning sensible results.

The NPC1 N-terminal domain depicted a sub-domain architecture when tested in dynamic data, showing a correlation between its modularity and its proposed function. Further analysis of these modules, and experimental tests (e.g. directed mutagenesis) in these modules might provide important insights in the protein function and evolution, as well as important information for possible treatments of the NPC1 related diseases.

Evolutionarily, the *α*-amylase family displayed a clear sub-domain architecture. All its modules were tightly connected with its catalytic capabilities. These results give some insights into the evolution of a common fold, the TIM barrel, that have been of wide interest [77,80-83,85]. It can also provide guidance for new improvements of thermal stability, substrate plasticity, and in bioengineering of the amylase’s function.

A drawback of the sub-domain modularity identification for homologous aligned proteins presented here, is the relative low power and support for them. However, the support values can be improved by using a bigger dataset (if possible; see Methods). We are exploring the use of homology modeling to enrich the available data for a given protein system, and the mixture of MD simulations and evolutionary sampling.

## Competing interests

The authors declare that they have no competing interests.

## Authors’ contributions

JSH and CB conceived of the study. JSH carry out the experiments and test the method. JSH, ES, and CB checked the statistical models, wrote and reviewed the manuscript. All authors read and approved the final manuscript.

## Supplementary Material

Additional file 1 Supporting Tables. Click here for file
