# Supplementary material for: Defining structural and evolutionary modules in proteins: a community detection approach to explore sub-domain architecture
Source: BMC Struct Biol. 2013 Oct 16;13:20. doi: 10.1186/1472-6807-13-20 (PMC4016585; doi:10.1186/1472-6807-13-20)
Supplement: Additional file 1 — Supporting Tables. [file 1472-6807-13-20-S1.pdf]

Supporting information for "Defining Structural and Evolutionary Modules in Proteins: A Community Detection Approach to explore sub-domain architecture"

Jose Sergio Hleap Edward Susko Christian Blouin

Supporting Information Content

List of Tables

S1 Residues membership for the *H. sapiens* α-amylase (PDB code 1U33, chain A) 5
S2 Residues membership for the *N. polysaccharea* α-amylase (PDB code 1MVY, chain A) 12
S3 Residues membership for the *T. vulgaris R47* α-amylase (PDB code 1VFM, chain A) 18
S4 Residues membership for the *S. scrofa* α-amylase (PDB code 1OSE, chain A) 25
S5 Residues membership for the *B. licheniformis* α-amylase (PDB code 1OB0, chain A) 32
S6 Residues membership for the *B. subtilis* α-amylase (PDB code 1UA7, chain A) 39
S7 Residues membership for the *S. scrofa* α-amylase (PDB code 1PIF, chain A) 45
S8 Residues membership for the *S. scrofa* α-amylase (PDB code 1PPI, chain A) 52
S9 Residues membership for the *B. circulans* α-amylase (PDB code 1PJ9, chain A) 59
S10 Residues membership for the *. stearothermophilus maltose* α-amylase (PDB code 1QHP, chain A) 66
S11 Residues membership for the *B. stearothermophilus maltose* α-amylase (PDB code 1QHO, chain A) 72
S12 Residues membership for the *S. scrofa* α-amylase (PDB code 1UA3, chain A) 79
S13 Residues membership for the *P. haloplanctis* α-amylase (PDB code 1G94, chain A) 86

S14 Residues membership for the *N. polysaccharea* α-amylase (PDB code 1JG9, chain A) 93
S15 Residues membership for the *T. maritima* α-amylase (PDB code 1GJU, chain A) 99
S16 Residues membership for the *P. stutzeri* α-amylase (PDB code 1GCY, chain A) 106
S17 Residues membership for the *H. sapiens* α-amylase (PDB code 1HNY, chain A) 113
S18 Residues membership for the *T. maritima* α-amylase (PDB code 1GJW, chain A) 120
S19 Residues membership for the *S. scrofa* α-amylase (PDB code 1HX0, chain A) 126
S20 Residues membership for the *B. stearothermophilus B* α-amylase (PDB code 1HVX, chain A) 133
S21 Residues membership for the *S. scrofa* α-amylase (PDB code 1JFH, chain A) 140
S22 Residues membership for the *T. molitor* α-amylase (PDB code 1JAE, chain A) 147
S23 Residues membership for the *N. polysaccharea* α-amylase (PDB code 1S46, chain A) 153
S24 Residues membership for the *T. molitor* α-amylase (PDB code 1TMQ, chain A) 160
S25 Residues membership for the *T. vulgaris R47* α-amylase (PDB code 1VB9, chain A) 167
S26 Residues membership for the *H. sapiens* α-amylase (PDB code 1U33, chain A) 174
S27 Residues membership for the *T. thermosulfurigenes EM1* α-amylase (PDB code 1A47, chain A) 180
S28 Residues membership for the *B. circulans* α-amylase (PDB code 1KCL, chain A) 187
S29 Residues membership for the *P. haloplanctis* α-amylase (PDB code 1AQH, chain A) 194
S30 Residues membership for the *H. vulgare* α-amylase (PDB code 1AMY, chain A) 201
S31 Residues membership for the *H. vulgare* α-amylase (PDB code 1AVA, chain A) 207
S32 Residues membership for the *P. haloplanctis* α-amylase (PDB code 1AQM, chain A) 214
S33 Residues membership for the *B. subtilis* α-amylase (PDB code 1BAG, chain A) 221
S34 Residues membership for the *P. haloplanctis* α-amylase (PDB code 1B0I, chain A) 228
S35 Residues membership for the *B. licheniformis* α-amylase (PDB code 1BLI, chain A) 234
S36 Residues membership for the *P. amyloderamosa* α-amylase (PDB code 1BF2, chain A) 241

S37 Residues membership for the *T. vulgaris R47* α-amylase (PDB code 1BVZ, chain A) 248
S38 Residues membership for the *H. sapiens* α-amylase (PDB code 1BSI, chain A) 255
S39 Residues membership for the *B. circulans,s8* α-amylase (PDB code 1CGT, chain A) 261
S40 Residues membership for the *B. circulans* α-amylase (PDB code 1CDG, chain A) 268
S41 Residues membership for the *T. thermosulfurigenes EM1* α-amylase (PDB code 1CIU, chain A) 275
S42 Residues membership for the *B. circulans,s8* α-amylase (PDB code 1CGU, chain A) 282
S43 Residues membership for the *B. circulans* α-amylase (PDB code 1CXE, chain A) 288
S44 Residues membership for the *T. molitor* α-amylase (PDB code 1CLV, chain A) 295
S45 Residues membership for the *B. circulans* α-amylase (PDB code 1CXK, chain A) 302
S46 Residues membership for the *B. circulans* α-amylase (PDB code 1CXF, chain A) 309
S47 Residues membership for the *B. stearothermophilus* α-amylase (PDB code 1CYG, chain A) 315
S48 Residues membership for the *B. circulans* α-amylase (PDB code 1CXL, chain A) 322
S49 Residues membership for the *S. scrofa* α-amylase (PDB code 1DHK, chain A) 329
S50 Residues membership for the *B. circulans* α-amylase (PDB code 1D3C, chain A) 336
S51 Residues membership for the *S. solfataricus KM1* α-amylase (PDB code 1EHA, chain A) 342
S52 Residues membership for the *S. solfataricus KM1* α-amylase (PDB code 1EH9, chain A) 349
S53 Residues membership for the *T. vulgaris R47* α-amylase (PDB code 1VFO, chain A) 356
S54 Residues membership for the *N. polysaccharea* α-amylase (PDB code 1G5A, chain A) 363
S55 Residues membership for the *T. molitor* α-amylase (PDB code 1VIW, chain A) 369
S56 Residues membership for the *B. cereus* α-amylase (PDB code 1UOK, chain A) 376
S57 Residues membership for the *H. sapiens* α-amylase (PDB code 1SMD, chain A) 383
S58 Residues membership for the *B. circulans* α-amylase (PDB code 1OT1, chain A) 390
S59 Residues membership for the *T. vulgaris R47* α-amylase (PDB code 1WZL, chain A) 396

S60 Residues membership for the *N. polysaccharea* α-amylase (PDB code 1ZS2, chain A) 403
S61 Residues membership for the *T. vulgaris R47* α-amylase (PDB code 1WZM, chain A) 410
S62 Residues membership for the *H. sapiens* α-amylase (PDB code 2QMK, chain A) 417
S63 Residues membership for the *A.niger* α-amylase (PDB code 2AAA, chain A) 423
S64 Residues membership for the *A. oryzae* α-amylase (PDB code 2TAA, chain A) 430
S65 Residues membership for the *H. sapiens* α-amylase (PDB code 2QV4, chain A) 437
S66 Residues membership for the *H. sapiens* α-amylase (PDB code 3BAJ, chain A) 444
S67 Residues membership for the *H. sapiens* α-amylase (PDB code 3BAI, chain A) 450
S68 Residues membership for the *T. thermosulfurigenes EM1* α-amylase (PDB code 3BMV, chain A) 457
S69 Residues membership for the *H. sapiens* α-amylase (PDB code 3BAW, chain A) 464
S70 Residues membership for the *B. circulans,s8* α-amylase (PDB code 3CGT, chain A) 471
S71 Residues membership for the *T. thermosulfurigenes EM1* α-amylase (PDB code 3BMW, chain A) 477
S72 Residues membership for the *S. scrofa* α-amylase (PDB code 3L2M, chain A) 484
S73 Residues membership for the *S. scrofa* α-amylase (PDB code 3L2L, chain A) 491
S74 Residues membership for the *B. circulans,s8* α-amylase (PDB code 4CGT, chain A) 498
S75 Residues membership for the *N. polysaccharea* α-amylase (PDB code 3UEQ, chain A) 504
S76 Residues membership for the *B. circulans,s8* α-amylase (PDB code 6CGT, chain A) 511
S77 Residues membership for the *B. circulans,s8* α-amylase (PDB code 5CGT, chain A) 518
S78 Residues membership for the *B. circulans,s8* α-amylase (PDB code 7CGT, chain A) 525
S79 Residues membership for the *A. oryzae* α-amylase (PDB code 6TAA, chain A) 531
S80 Residues membership for the *B. circulans,s8* α-amylase (PDB code 8CGT, chain A) 538
S81 Residues membership for the *A. oryzae* α-amylase (PDB code 7TAA, chain A) 545
S82 Residues membership for the *B. circulans,s8* α-amylase (PDB code 9CGT, chain A) 552

S83 Residues membership for the *B. licheniformis* α-amylase (PDB code 1VJS, chain A) 558
S84 Residues membership for the *T. vulgaris R47* α-amylase (PDB code 1WZK, chain A) 565
S85 Residues membership for the *N. polysaccharea* α-amylase (PDB code 1JGI, chain A) 572
S86 Residues membership for the *H. sapiens* NPC1 (PDB code 3GKH, chain A)protein 579

Supporting Tables

Table S1: Residues membership for the *H. sapiens* α-amylase (PDB code 1U33, chain A)

Homology index	Residue Index	Residue abbreviation	Module
0	131	ALA	A
1	132	VAL	A
2	133	ILE	B
3	134	TYR	B
4	135	GLN	B
5	136	ILE	C
6	137	PHE	C
7	138	PRO	C
8	170	GLY	C
9	171	ASP	C
10	172	LEU	C
11	173	LYS	C
12	174	GLY	C
13	175	VAL	C
14	176	ILE	C
15	177	ASP	C
16	178	ARG	C
17	179	LEU	C
18	180	PRO	C
19	181	TYR	C
20	182	LEU	C
21	183	GLU	C
22	184	GLU	C
23	185	LEU	C
24	186	GLY	C
25	187	VAL	C
26	188	THR	C
27	189	ALA	C
28	190	LEU	C
29	191	TYR	C

Continued on next page

Table S1 – *Continued from previous page*

Homology index	Residue Index	Residue abbreviation	Module
30	192	PHE	C
31	193	THR	C
32	194	PRO	C
33	195	ILE	C
34	196	PHE	C
35	197	ALA	C
36	198	SER	C
37	199	PRO	C
38	200	SER	C
39	201	HIS	C
40	202	HIS	C
41	203	LYS	C
42	204	TYR	C
43	205	ASP	C
44	206	THR	C
45	207	ALA	C
46	208	ASP	C
47	210	LEU	C
48	211	ALA	C
49	212	ILE	C
50	213	ASP	C
51	214	PRO	C
52	215	GLN	C
53	216	PHE	C
54	217	GLY	C
55	218	ASP	C
56	219	LEU	C
57	220	PRO	C
58	221	THR	C
59	222	PHE	C
60	223	ARG	C
61	224	ARG	C
62	225	LEU	C
63	226	VAL	C
64	227	ASP	C
65	228	GLU	C
66	229	ALA	C
67	230	HIS	C
68	231	ARG	C
69	232	ARG	C
70	233	GLY	C
71	234	ILE	C
72	235	LYS	C
73	236	ILE	C
74	237	ILE	C
75	238	LEU	C

Continued on next page

Table S1 – *Continued from previous page*

Homology index	Residue Index	Residue abbreviation	Module
76	239	ASP	C
77	240	ALA	C
78	241	VAL	C
79	242	PHE	C
80	243	ASN	C
81	244	HIS	C
82	245	ALA	C
83	246	GLY	C
84	247	ASP	C
85	248	GLN	C
86	249	PHE	C
87	250	PHE	C
88	251	ALA	C
89	252	PHE	C
90	265	LYS	C
91	266	ASP	C
92	267	TRP	C
93	268	PHE	C
94	269	PHE	B
95	284	GLU	B
96	285	THR	B
97	286	PHE	B
98	287	ALA	B
99	293	MET	B
100	294	PRO	B
101	295	LYS	B
102	296	LEU	B
103	297	ARG	B
104	298	THR	B
105	299	GLU	B
106	300	ASN	B
107	301	PRO	B
108	302	GLU	B
109	303	VAL	B
110	304	LYS	B
111	305	GLU	B
112	306	TYR	B
113	307	LEU	B
114	308	PHE	B
115	309	ASP	B
116	310	VAL	B
117	311	ALA	B
118	312	ARG	B
119	313	PHE	B
120	314	TRP	B
121	315	MET	B

Continued on next page

Table S1 – *Continued from previous page*

Homology index	Residue Index	Residue abbreviation	Module
122	316	GLU	B
123	317	GLN	C
124	318	GLY	C
125	319	ILE	C
126	320	ASP	C
127	321	GLY	C
128	322	TRP	B
129	323	ARG	D
130	324	LEU	B
131	325	ASN	B
132	326	VAL	B
133	327	ALA	B
134	328	ASN	B
135	329	GLU	B
136	330	VAL	B
137	331	ASP	B
138	332	HIS	B
139	333	ALA	B
140	334	PHE	B
141	335	TRP	B
142	336	ARG	B
143	337	GLU	B
144	338	PHE	B
145	339	ARG	B
146	340	ARG	B
147	341	LEU	B
148	342	VAL	B
149	343	LYS	B
150	349	ALA	B
151	350	LEU	B
152	351	ILE	B
153	352	VAL	C
154	353	GLY	B
155	354	GLU	B
156	355	ILE	B
157	356	TRP	B
158	357	HIS	B
159	358	ASP	B
160	359	ALA	B
161	360	SER	B
162	361	GLY	B
163	362	TRP	B
164	363	LEU	B
165	364	MET	B
166	368	PHE	B
167	369	ASP	B

Continued on next page

Table S1 – *Continued from previous page*

Homology index	Residue Index	Residue abbreviation	Module
168	370	SER	B
169	371	VAL	B
170	372	MET	D
171	373	ASN	D
172	374	TYR	D
173	375	LEU	D
174	377	ARG	D
175	378	GLU	D
176	379	SER	D
177	380	VAL	D
178	381	ILE	D
179	382	ARG	D
180	383	PHE	D
181	384	PHE	D
182	389	ILE	D
183	390	HIS	A
184	391	ALA	A
185	392	GLU	A
186	397	GLU	A
187	398	LEU	D
188	399	THR	D
189	400	ARG	D
190	401	ALA	D
191	402	ARG	B
192	403	MET	B
193	409	ALA	B
194	410	ALA	B
195	411	GLN	B
196	412	GLY	B
197	413	LEU	B
198	414	TRP	D
199	415	ASN	D
200	416	LEU	D
201	417	LEU	A
202	418	ASP	D
203	419	SER	D
204	420	HIS	D
205	421	ASP	D
206	422	THR	D
207	423	GLU	D
208	424	ARG	D
209	425	PHE	D
210	426	LEU	D
211	427	THR	D
212	433	GLU	A
213	435	LYS	A

Continued on next page

Table S1 – *Continued from previous page*

Homology index	Residue Index	Residue abbreviation	Module
214	436	PHE	A
215	437	ARG	A
216	438	LEU	A
217	439	ALA	A
218	440	VAL	A
219	441	LEU	A
220	442	PHE	A
221	443	GLN	A
222	444	MET	A
223	445	THR	A
224	446	TYR	A
225	447	LEU	D
226	448	GLY	D
227	449	THR	D
228	450	PRO	A
229	451	LEU	D
230	452	ILE	A
231	453	TYR	D
232	454	TYR	C
233	455	GLY	D
234	456	ASP	A
235	470	ARG	C
236	471	PRO	C
237	472	MET	D
238	473	ILE	D
239	474	TRP	D
240	483	LEU	A
241	484	PHE	A
242	485	GLU	A
243	486	PHE	A
244	487	TYR	A
245	488	LYS	A
246	489	GLU	A
247	490	LEU	A
248	491	ILE	A
249	492	ARG	A
250	493	LEU	A
251	494	ARG	A
252	495	HIS	A
253	496	ARG	A
254	497	LEU	A
255	498	ALA	A
256	502	ARG	A
257	503	GLY	A
258	504	ASN	A
259	505	VAL	A

Continued on next page

Table S1 – *Continued from previous page*

Homology index	Residue Index	Residue abbreviation	Module
260	507	SER	A
261	508	TRP	A
262	509	HIS	A
263	510	ALA	A
264	513	GLN	A
265	514	ALA	A
266	515	ASN	A
267	516	LEU	A
268	517	TYR	A
269	518	ALA	A
270	519	PHE	A
271	520	VAL	A
272	521	ARG	A
273	526	GLN	A
274	527	HIS	A
275	528	VAL	A
276	529	GLY	A
277	530	VAL	A
278	531	VAL	A
279	532	LEU	A
280	533	ASN	A
281	536	GLY	A
282	537	GLU	A
283	538	LYS	A
284	539	GLN	A
285	540	THR	A
286	541	VAL	A
287	542	LEU	A
288	552	THR	A
289	553	TRP	A
290	554	LEU	A
291	555	ASP	A
292	556	CYS	A
293	557	LEU	A
294	574	ARG	A
295	575	PRO	A
296	576	TYR	A
297	577	GLN	A
298	578	GLY	A
299	579	MET	A
300	580	ILE	A
301	581	LEU	A
302	582	TRP	A

Table S2: Residues membership for the *N. polysaccharea* α-amylase (PDB code 1MVY, chain A)

**Homology index**	**Residue Index**	**Residue abbreviation**	**Module**
0	98	GLN	A
1	99	VAL	A
2	100	GLY	B
3	101	GLY	B
4	102	VAL	B
5	103	CYS	C
6	104	TYR	C
7	105	VAL	C
8	110	GLY	C
9	111	ASP	C
10	112	LEU	C
11	113	LYS	C
12	114	GLY	C
13	115	LEU	C
14	116	LYS	C
15	117	ASP	C
16	118	LYS	C
17	119	ILE	C
18	120	PRO	C
19	121	TYR	C
20	122	PHE	C
21	123	GLN	C
22	124	GLU	C
23	125	LEU	C
24	126	GLY	C
25	127	LEU	C
26	128	THR	C
27	129	TYR	C
28	130	LEU	C
29	131	TYR	C
30	132	LEU	C
31	133	MET	C
32	134	PRO	C
33	135	LEU	C
34	136	PHE	C
35	137	LYS	C
36	138	CYS	C
37	139	PRO	C
38	141	GLY	C
39	144	ASP	C
40	145	GLY	C
41	146	GLY	C
42	147	TYR	C
43	148	ALA	C

Continued on next page

Table S2 – *Continued from previous page*

Homology index	Residue Index	Residue abbreviation	Module
44	149	VAL	C
45	150	SER	C
46	151	SER	C
47	153	ARG	C
48	154	ASP	C
49	155	VAL	C
50	156	ASN	C
51	157	PRO	C
52	158	ALA	C
53	159	LEU	C
54	160	GLY	C
55	161	THR	C
56	162	ILE	C
57	163	GLY	C
58	164	ASP	C
59	165	LEU	C
60	166	ARG	C
61	167	GLU	C
62	168	VAL	C
63	169	ILE	C
64	170	ALA	C
65	171	ALA	C
66	172	LEU	C
67	173	HIS	C
68	174	GLU	C
69	175	ALA	C
70	176	GLY	C
71	177	ILE	C
72	178	SER	C
73	179	ALA	C
74	180	VAL	C
75	181	VAL	C
76	182	ASP	C
77	183	PHE	C
78	184	ILE	C
79	185	PHE	C
80	186	ASN	C
81	187	HIS	C
82	188	THR	C
83	189	SER	C
84	190	ASN	C
85	191	GLU	C
86	192	HIS	C
87	193	GLU	C
88	194	TRP	C
89	195	ALA	C

Continued on next page

Table S2 – *Continued from previous page*

Homology index	Residue Index	Residue abbreviation	Module
90	206	ASP	C
91	207	ASN	C
92	208	PHE	C
93	209	TYR	C
94	210	TYR	B
95	247	TRP	B
96	248	THR	B
97	249	THR	B
98	250	PHE	B
99	254	GLN	B
100	255	TRP	B
101	256	ASP	B
102	257	LEU	B
103	258	ASN	B
104	259	TYR	B
105	260	SER	B
106	261	ASN	B
107	262	PRO	B
108	263	TRP	B
109	264	VAL	B
110	265	PHE	B
111	266	ARG	B
112	267	ALA	B
113	268	MET	B
114	269	ALA	B
115	270	GLY	B
116	271	GLU	B
117	272	MET	B
118	273	LEU	B
119	274	PHE	B
120	275	LEU	B
121	276	ALA	B
122	277	ASN	B
123	278	LEU	C
124	279	GLY	C
125	280	VAL	C
126	281	ASP	C
127	282	ILE	C
128	283	LEU	B
129	284	ARG	D
130	285	MET	B
131	286	ASP	B
132	287	ALA	B
133	288	VAL	B
134	289	ALA	B
135	290	PHE	B

Continued on next page

Table S2 – *Continued from previous page*

Homology index	Residue Index	Residue abbreviation	Module
136	291	ILE	B
137	292	TRP	B
138	306	HIS	B
139	307	ALA	B
140	308	LEU	B
141	309	ILE	B
142	310	ARG	B
143	311	ALA	B
144	312	PHE	B
145	313	ASN	B
146	314	ALA	B
147	315	VAL	B
148	316	MET	B
149	317	ARG	B
150	323	VAL	B
151	324	PHE	B
152	325	PHE	B
153	326	LYS	C
154	327	SER	B
155	328	GLN	B
156	329	ALA	B
157	330	ILE	B
158	331	VAL	B
159	332	HIS	B
160	336	VAL	B
161	337	VAL	B
162	338	GLN	B
163	339	TYR	B
164	340	ILE	B
165	341	GLY	B
166	345	CYS	B
167	346	GLN	B
168	347	ILE	B
169	348	GLY	B
170	349	TYR	D
171	350	ASN	D
172	351	PRO	D
173	352	LEU	D
174	354	MET	D
175	355	ALA	D
176	356	LEU	D
177	357	LEU	D
178	358	TRP	D
179	359	ASN	D
180	360	THR	D
181	361	LEU	D

Continued on next page

Table S2 – *Continued from previous page*

Homology index	Residue Index	Residue abbreviation	Module
182	364	ARG	D
183	365	GLU	A
184	366	VAL	A
185	367	ASN	A
186	372	ALA	A
187	373	LEU	D
188	374	THR	D
189	375	TYR	D
190	376	ARG	D
191	377	HIS	B
192	378	ASN	B
193	381	GLU	B
194	382	HIS	B
195	383	THR	B
196	384	ALA	B
197	385	TRP	B
198	386	VAL	D
199	387	ASN	D
200	388	TYR	D
201	389	VAL	A
202	390	ARG	D
203	391	SER	D
204	392	HIS	D
205	393	ASP	D
206	394	ASP	D
207	448	SER	D
208	450	THR	D
209	451	ALA	D
210	452	ALA	D
211	453	ALA	D
212	464	ALA	A
213	467	ARG	A
214	468	ILE	A
215	469	LYS	A
216	470	LEU	A
217	471	LEU	A
218	472	TYR	A
219	473	SER	A
220	474	ILE	A
221	475	ALA	A
222	476	LEU	A
223	477	SER	A
224	478	THR	A
225	479	GLY	D
226	480	GLY	D
227	481	LEU	D

Continued on next page

Table S2 – *Continued from previous page*

**Homology index**	**Residue Index**	**Residue abbreviation**	**Module**
228	482	PRO	A
229	483	LEU	D
230	484	ILE	A
231	485	TYR	D
232	486	LEU	C
233	487	GLY	D
234	488	ASP	A
235	514	PRO	C
236	515	ARG	C
237	516	TYR	D
238	517	ASN	D
239	521	TYR	D
240	534	ILE	A
241	535	TYR	A
242	536	GLN	A
243	537	GLY	A
244	538	LEU	A
245	539	ARG	A
246	540	HIS	A
247	541	MET	A
248	542	ILE	A
249	543	ALA	A
250	544	VAL	A
251	545	ARG	A
252	546	GLN	A
253	547	SER	A
254	548	ASN	A
255	549	PRO	A
256	553	GLY	A
257	554	GLY	A
258	555	ARG	A
259	556	LEU	A
260	558	THR	A
261	559	PHE	A
262	560	ASN	A
263	561	THR	A
264	563	ASN	A
265	564	LYS	A
266	565	HIS	A
267	566	ILE	A
268	567	ILE	A
269	568	GLY	A
270	569	TYR	A
271	570	ILE	A
272	571	ARG	A
273	573	ASN	A

Continued on next page

Table S2 – *Continued from previous page*

**Homology index**	**Residue Index**	**Residue abbreviation**	**Module**
274	574	ALA	A
275	575	LEU	A
276	576	LEU	A
277	577	ALA	A
278	578	PHE	A
279	579	GLY	A
280	580	ASN	A
281	584	TYR	A
282	585	PRO	A
283	586	GLN	A
284	587	THR	A
285	588	VAL	A
286	589	THR	A
287	590	ALA	A
288	599	LYS	A
289	600	ALA	A
290	601	HIS	A
291	602	ASP	A
292	603	LEU	A
293	604	ILE	A
294	617	LEU	A
295	618	GLN	A
296	619	PRO	A
297	621	GLN	A
298	622	VAL	A
299	623	MET	A
300	624	TRP	A
301	625	LEU	A
302	626	GLU	A

Table S3: Residues membership for the *T. vulgaris R47* α-amylase (PDB code 1VFM, chain A)

**Homology index**	**Residue Index**	**Residue abbreviation**	**Module**
0	131	ALA	A
1	132	VAL	A
2	133	ILE	B
3	134	TYR	B
4	135	GLN	B
5	136	ILE	C
6	137	PHE	C
7	138	PRO	C
8	170	GLY	C

Continued on next page

Table S3 – *Continued from previous page*

Homology index	Residue Index	Residue abbreviation	Module
9	171	ASP	C
10	172	LEU	C
11	173	LYS	C
12	174	GLY	C
13	175	VAL	C
14	176	ILE	C
15	177	ASP	C
16	178	ARG	C
17	179	LEU	C
18	180	PRO	C
19	181	TYR	C
20	182	LEU	C
21	183	GLU	C
22	184	GLU	C
23	185	LEU	C
24	186	GLY	C
25	187	VAL	C
26	188	THR	C
27	189	ALA	C
28	190	LEU	C
29	191	TYR	C
30	192	PHE	C
31	193	THR	C
32	194	PRO	C
33	195	ILE	C
34	196	PHE	C
35	197	ALA	C
36	198	SER	C
37	199	PRO	C
38	200	SER	C
39	201	HIS	C
40	202	HIS	C
41	203	LYS	C
42	204	TYR	C
43	205	ASP	C
44	206	THR	C
45	207	ALA	C
46	208	ASP	C
47	210	LEU	C
48	211	ALA	C
49	212	ILE	C
50	213	ASP	C
51	214	PRO	C
52	215	GLN	C
53	216	PHE	C
54	217	GLY	C

Continued on next page

Table S3 – *Continued from previous page*

Homology index	Residue Index	Residue abbreviation	Module
55	218	ASP	C
56	219	LEU	C
57	220	PRO	C
58	221	THR	C
59	222	PHE	C
60	223	ARG	C
61	224	ARG	C
62	225	LEU	C
63	226	VAL	C
64	227	ASP	C
65	228	GLU	C
66	229	ALA	C
67	230	HIS	C
68	231	ARG	C
69	232	ARG	C
70	233	GLY	C
71	234	ILE	C
72	235	LYS	C
73	236	ILE	C
74	237	ILE	C
75	238	LEU	C
76	239	ASP	C
77	240	ALA	C
78	241	VAL	C
79	242	PHE	C
80	243	ASN	C
81	244	HIS	C
82	245	ALA	C
83	246	GLY	C
84	247	ASP	C
85	248	GLN	C
86	249	PHE	C
87	250	PHE	C
88	251	ALA	C
89	252	PHE	C
90	265	LYS	C
91	266	ASP	C
92	267	TRP	C
93	268	PHE	C
94	269	PHE	B
95	284	GLU	B
96	285	THR	B
97	286	PHE	B
98	287	ALA	B
99	293	MET	B
100	294	PRO	B

Continued on next page

Table S3 – *Continued from previous page*

Homology index	Residue Index	Residue abbreviation	Module
101	295	LYS	B
102	296	LEU	B
103	297	ARG	B
104	298	THR	B
105	299	GLU	B
106	300	ASN	B
107	301	PRO	B
108	302	GLU	B
109	303	VAL	B
110	304	LYS	B
111	305	GLU	B
112	306	TYR	B
113	307	LEU	B
114	308	PHE	B
115	309	ASP	B
116	310	VAL	B
117	311	ALA	B
118	312	ARG	B
119	313	PHE	B
120	314	TRP	B
121	315	MET	B
122	316	GLU	B
123	317	GLN	C
124	318	GLY	C
125	319	ILE	C
126	320	ASP	C
127	321	GLY	C
128	322	TRP	B
129	323	ARG	D
130	324	LEU	B
131	325	ASN	B
132	326	VAL	B
133	327	ALA	B
134	328	ASN	B
135	329	GLU	B
136	330	VAL	B
137	331	ASP	B
138	332	HIS	B
139	333	ALA	B
140	334	PHE	B
141	335	TRP	B
142	336	ARG	B
143	337	GLU	B
144	338	PHE	B
145	339	ARG	B
146	340	ARG	B

Continued on next page

Table S3 – *Continued from previous page*

Homology index	Residue Index	Residue abbreviation	Module
147	341	LEU	B
148	342	VAL	B
149	343	LYS	B
150	349	ALA	B
151	350	LEU	B
152	351	ILE	B
153	352	VAL	C
154	353	GLY	B
155	354	GLU	B
156	355	ILE	B
157	356	TRP	B
158	357	HIS	B
159	358	ASP	B
160	359	ALA	B
161	360	SER	B
162	361	GLY	B
163	362	TRP	B
164	363	LEU	B
165	364	MET	B
166	368	PHE	B
167	369	ASP	B
168	370	SER	B
169	371	VAL	B
170	372	MET	D
171	373	ASN	D
172	374	TYR	D
173	375	LEU	D
174	377	ARG	D
175	378	GLU	D
176	379	SER	D
177	380	VAL	D
178	381	ILE	D
179	382	ARG	D
180	383	PHE	D
181	384	PHE	D
182	389	ILE	D
183	390	HIS	A
184	391	ALA	A
185	392	GLU	A
186	397	GLU	A
187	398	LEU	D
188	399	THR	D
189	400	ARG	D
190	401	ALA	D
191	402	ARG	B
192	403	MET	B

Continued on next page

Table S3 – *Continued from previous page*

Homology index	Residue Index	Residue abbreviation	Module
193	409	ALA	B
194	410	ALA	B
195	411	GLN	B
196	412	GLY	B
197	413	LEU	B
198	414	TRP	D
199	415	ASN	D
200	416	LEU	D
201	417	LEU	A
202	418	ASP	D
203	419	SER	D
204	420	HIS	D
205	421	ASN	D
206	422	THR	D
207	423	GLU	D
208	424	ARG	D
209	425	PHE	D
210	426	LEU	D
211	427	THR	D
212	433	GLU	A
213	435	LYS	A
214	436	PHE	A
215	437	ARG	A
216	438	LEU	A
217	439	ALA	A
218	440	VAL	A
219	441	LEU	A
220	442	PHE	A
221	443	GLN	A
222	444	MET	A
223	445	THR	A
224	446	TYR	A
225	447	LEU	D
226	448	GLY	D
227	449	THR	D
228	450	PRO	A
229	451	LEU	D
230	452	ILE	A
231	453	TYR	D
232	454	TYR	C
233	455	GLY	D
234	456	ASP	A
235	470	ARG	C
236	471	PRO	C
237	472	MET	D
238	473	ILE	D

Continued on next page

Table S3 – *Continued from previous page*

**Homology index**	**Residue Index**	**Residue abbreviation**	**Module**
239	474	TRP	D
240	483	LEU	A
241	484	PHE	A
242	485	GLU	A
243	486	PHE	A
244	487	TYR	A
245	488	LYS	A
246	489	GLU	A
247	490	LEU	A
248	491	ILE	A
249	492	ARG	A
250	493	LEU	A
251	494	ARG	A
252	495	HIS	A
253	496	ARG	A
254	497	LEU	A
255	498	ALA	A
256	502	ARG	A
257	503	GLY	A
258	504	ASN	A
259	505	VAL	A
260	507	SER	A
261	508	TRP	A
262	509	HIS	A
263	510	ALA	A
264	513	GLN	A
265	514	ALA	A
266	515	ASN	A
267	516	LEU	A
268	517	TYR	A
269	518	ALA	A
270	519	PHE	A
271	520	VAL	A
272	521	ARG	A
273	526	GLN	A
274	527	HIS	A
275	528	VAL	A
276	529	GLY	A
277	530	VAL	A
278	531	VAL	A
279	532	LEU	A
280	533	ASN	A
281	536	GLY	A
282	537	GLU	A
283	538	LYS	A
284	539	GLN	A

Continued on next page

Table S3 – *Continued from previous page*

Homology index	Residue Index	Residue abbreviation	Module
285	540	THR	A
286	541	VAL	A
287	542	LEU	A
288	552	THR	A
289	553	TRP	A
290	554	LEU	A
291	555	ASP	A
292	556	CYS	A
293	557	LEU	A
294	574	ARG	A
295	575	PRO	A
296	576	TYR	A
297	577	GLN	A
298	578	GLY	A
299	579	MET	A
300	580	ILE	A
301	581	LEU	A
302	582	TRP	A

Table S4: Residues membership for the *S. scrofa* α-amylase (PDB code 1OSE, chain A)

Homology index	Residue Index	Residue abbreviation	Module
0	11	THR	A
1	12	SER	A
2	13	ILE	B
3	14	VAL	B
4	15	HIS	B
5	16	LEU	C
6	17	PHE	C
7	18	GLU	C
8	19	TRP	C
9	20	ARG	C
10	21	TRP	C
11	22	VAL	C
12	23	ASP	C
13	24	ILE	C
14	25	ALA	C
15	26	LEU	C
16	27	GLU	C
17	28	CYS	C
18	29	GLU	C
19	30	ARG	C

Continued on next page

Table S4 – *Continued from previous page*

Homology index	Residue Index	Residue abbreviation	Module
20	32	LEU	C
21	33	GLY	C
22	34	PRO	C
23	35	LYS	C
24	36	GLY	C
25	37	PHE	C
26	38	GLY	C
27	39	GLY	C
28	40	VAL	C
29	41	GLN	C
30	42	VAL	C
31	43	SER	C
32	44	PRO	C
33	45	PRO	C
34	46	ASN	C
35	47	GLU	C
36	48	ASN	C
37	49	ILE	C
38	51	VAL	C
39	59	TRP	C
40	60	GLU	C
41	61	ARG	C
42	62	TYR	C
43	63	GLN	C
44	64	PRO	C
45	65	VAL	C
46	66	SER	C
47	67	TYR	C
48	68	LYS	C
49	69	LEU	C
50	70	CYS	C
51	71	THR	C
52	72	ARG	C
53	73	SER	C
54	74	GLY	C
55	75	ASN	C
56	76	GLU	C
57	77	ASN	C
58	78	GLU	C
59	79	PHE	C
60	80	ARG	C
61	81	ASP	C
62	82	MET	C
63	83	VAL	C
64	84	THR	C
65	85	ARG	C

Continued on next page

Table S4 – *Continued from previous page*

**Homology index**	**Residue Index**	**Residue abbreviation**	**Module**
66	86	CYS	C
67	87	ASN	C
68	88	ASN	C
69	89	VAL	C
70	90	GLY	C
71	91	VAL	C
72	92	ARG	C
73	93	ILE	C
74	94	TYR	C
75	95	VAL	C
76	96	ASP	C
77	97	ALA	C
78	98	VAL	C
79	99	ILE	C
80	100	ASN	C
81	101	HIS	C
82	102	MET	C
83	103	CYS	C
84	104	GLY	C
85	105	SER	C
86	126	PHE	C
87	130	PRO	C
88	131	TYR	C
89	132	SER	C
90	133	ALA	C
91	134	TRP	C
92	135	ASP	C
93	136	PHE	C
94	137	ASN	B
95	160	CYS	B
96	161	GLN	B
97	162	LEU	B
98	163	VAL	B
99	165	LEU	B
100	166	LEU	B
101	167	ASP	B
102	168	LEU	B
103	169	ALA	B
104	170	LEU	B
105	171	GLU	B
106	172	LYS	B
107	173	ASP	B
108	174	TYR	B
109	175	VAL	B
110	176	ARG	B
111	177	SER	B

Continued on next page

Table S4 – *Continued from previous page*

**Homology index**	**Residue Index**	**Residue abbreviation**	**Module**
112	178	MET	B
113	179	ILE	B
114	180	ALA	B
115	181	ASP	B
116	182	TYR	B
117	183	LEU	B
118	184	ASN	B
119	185	LYS	B
120	186	LEU	B
121	187	ILE	B
122	188	ASP	B
123	189	ILE	C
124	190	GLY	C
125	191	VAL	C
126	192	ALA	C
127	193	GLY	C
128	194	PHE	B
129	195	ARG	D
130	196	ILE	B
131	197	ASP	B
132	198	ALA	B
133	199	SER	B
134	200	LYS	B
135	201	HIS	B
136	202	MET	B
137	203	TRP	B
138	204	PRO	B
139	205	GLY	B
140	206	ASP	B
141	207	ILE	B
142	208	LYS	B
143	209	ALA	B
144	210	VAL	B
145	211	LEU	B
146	212	ASP	B
147	213	LYS	B
148	214	LEU	B
149	215	HIS	B
150	228	PRO	B
151	229	PHE	B
152	230	ILE	B
153	231	PHE	C
154	232	GLN	B
155	233	GLU	B
156	234	VAL	B
157	235	ILE	B

Continued on next page

Table S4 – *Continued from previous page*

**Homology index**	**Residue Index**	**Residue abbreviation**	**Module**
158	236	ASP	B
159	237	LEU	B
160	244	SER	B
161	245	SER	B
162	246	GLU	B
163	247	TYR	B
164	248	PHE	B
165	249	GLY	B
166	250	ASN	B
167	251	GLY	B
168	252	ARG	B
169	253	VAL	B
170	254	THR	D
171	255	GLU	D
172	256	PHE	D
173	257	LYS	D
174	259	GLY	D
175	260	ALA	D
176	261	LYS	D
177	262	LEU	D
178	263	GLY	D
179	264	THR	D
180	265	VAL	D
181	266	VAL	D
182	272	GLU	D
183	273	LYS	A
184	274	MET	A
185	275	SER	A
186	280	TRP	A
187	281	GLY	D
188	282	GLU	D
189	283	GLY	D
190	285	GLY	D
191	286	PHE	B
192	287	MET	B
193	288	PRO	B
194	289	SER	B
195	290	ASP	B
196	291	ARG	B
197	292	ALA	B
198	293	LEU	D
199	294	VAL	D
200	295	PHE	D
201	296	VAL	A
202	297	ASP	D
203	298	ASN	D

Continued on next page

Table S4 – *Continued from previous page*

Homology index	Residue Index	Residue abbreviation	Module
204	299	HIS	D
205	300	ASP	D
206	301	ASN	D
207	302	GLN	D
208	314	THR	D
209	315	PHE	D
210	316	TRP	D
211	317	ASP	D
212	319	ARG	A
213	320	LEU	A
214	321	TYR	A
215	322	LYS	A
216	323	VAL	A
217	324	ALA	A
218	325	VAL	A
219	326	GLY	A
220	327	PHE	A
221	328	MET	A
222	329	LEU	A
223	330	ALA	A
224	331	HIS	A
225	333	TYR	D
226	334	GLY	D
227	335	PHE	D
228	336	THR	A
229	337	ARG	D
230	338	VAL	A
231	339	MET	D
232	340	SER	C
233	341	SER	D
234	342	TYR	A
235	343	ARG	C
236	381	ASP	C
237	382	TRP	D
238	383	VAL	D
239	384	CYS	D
240	387	ARG	A
241	388	TRP	A
242	389	ARG	A
243	390	GLU	A
244	391	ILE	A
245	392	ARG	A
246	393	ASN	A
247	394	MET	A
248	395	VAL	A
249	396	TRP	A

Continued on next page

Table S4 – *Continued from previous page*

Homology index	Residue Index	Residue abbreviation	Module
250	397	PHE	A
251	398	ARG	A
252	399	ASN	A
253	400	VAL	A
254	401	VAL	A
255	402	ASP	A
256	403	GLY	A
257	404	GLU	A
258	405	PRO	A
259	406	PHE	A
260	408	ASN	A
261	409	TRP	A
262	410	TRP	A
263	411	ASP	A
264	413	GLY	A
265	414	SER	A
266	415	ASN	A
267	416	GLN	A
268	417	VAL	A
269	418	ALA	A
270	419	PHE	A
271	420	GLY	A
272	421	ARG	A
273	422	GLY	A
274	423	ASN	A
275	425	GLY	A
276	426	PHE	A
277	427	ILE	A
278	428	VAL	A
279	429	PHE	A
280	430	ASN	A
281	433	ASP	A
282	434	TRP	A
283	435	GLN	A
284	436	LEU	A
285	437	SER	A
286	438	SER	A
287	439	THR	A
288	445	PRO	A
289	446	GLY	A
290	449	TYR	A
291	450	CYS	A
292	451	ASP	A
293	452	VAL	A
294	479	ILE	A
295	480	SER	A

Continued on next page

Table S4 – *Continued from previous page*

**Homology index**	**Residue Index**	**Residue abbreviation**	**Module**
296	481	ASN	A
297	486	PRO	A
298	487	PHE	A
299	488	ILE	A
300	489	ALA	A
301	490	ILE	A
302	491	HIS	A

Table S5: Residues membership for the *B. licheniformis* α-amylase (PDB code 1OB0, chain A)

**Homology index**	**Residue Index**	**Residue abbreviation**	**Module**
0	5	GLY	A
1	6	THR	A
2	7	LEU	B
3	8	MET	B
4	9	GLN	B
5	10	TYR	C
6	11	PHE	C
7	15	MET	C
8	20	GLN	C
9	21	HIS	C
10	22	TRP	C
11	23	LYS	C
12	24	ARG	C
13	25	LEU	C
14	26	GLN	C
15	27	ASN	C
16	28	ASP	C
17	29	SER	C
18	30	ALA	C
19	31	TYR	C
20	32	LEU	C
21	33	ALA	C
22	34	GLU	C
23	35	HIS	C
24	36	GLY	C
25	37	ILE	C
26	38	THR	C
27	39	ALA	C
28	40	VAL	C
29	41	TRP	C
30	42	ILE	C

Continued on next page

Table S5 – *Continued from previous page*

Homology index	Residue Index	Residue abbreviation	Module
31	43	PRO	C
32	44	PRO	C
33	45	ALA	C
34	46	TYR	C
35	47	LYS	C
36	48	GLY	C
37	49	THR	C
38	52	ALA	C
39	53	ASP	C
40	54	VAL	C
41	55	GLY	C
42	56	TYR	C
43	57	GLY	C
44	58	ALA	C
45	59	TYR	C
46	60	ASP	C
47	62	TYR	C
48	63	ASP	C
49	64	LEU	C
50	65	GLY	C
51	75	THR	C
52	76	LYS	C
53	77	TYR	C
54	78	GLY	C
55	79	THR	C
56	80	LYS	C
57	81	GLY	C
58	82	GLU	C
59	83	LEU	C
60	84	GLN	C
61	85	SER	C
62	86	ALA	C
63	87	ILE	C
64	88	LYS	C
65	89	SER	C
66	90	LEU	C
67	91	HIS	C
68	92	SER	C
69	93	ARG	C
70	94	ASP	C
71	95	ILE	C
72	96	ASN	C
73	97	VAL	C
74	98	TYR	C
75	99	GLY	C
76	100	ASP	C

Continued on next page

Table S5 – *Continued from previous page*

Homology index	Residue Index	Residue abbreviation	Module
77	101	VAL	C
78	102	VAL	C
79	103	ILE	C
80	104	ASN	C
81	105	HIS	C
82	106	LYS	C
83	107	GLY	C
84	108	GLY	C
85	109	ALA	C
86	141	PHE	C
87	154	LYS	C
88	155	TRP	C
89	156	HIS	C
90	157	TRP	C
91	158	TYR	C
92	159	HIS	C
93	160	PHE	C
94	161	ASP	B
95	194	ASP	B
96	195	TYR	B
97	196	LEU	B
98	197	MET	B
99	198	TYR	B
100	199	ALA	B
101	200	ASP	B
102	201	ILE	B
103	202	ASP	B
104	203	TYR	B
105	204	ASP	B
106	205	HIS	B
107	206	PRO	B
108	207	ASP	B
109	208	VAL	B
110	209	VAL	B
111	210	ALA	B
112	211	GLU	B
113	212	ILE	B
114	213	LYS	B
115	214	ARG	B
116	215	TRP	B
117	216	GLY	B
118	217	THR	B
119	218	TRP	B
120	219	TYR	B
121	220	ALA	B
122	221	ASN	B

Continued on next page

Table S5 – *Continued from previous page*

Homology index	Residue Index	Residue abbreviation	Module
123	223	LEU	C
124	224	GLN	C
125	225	LEU	C
126	226	ASP	C
127	227	GLY	C
128	228	PHE	B
129	229	ARG	D
130	230	LEU	B
131	231	ASP	B
132	232	ALA	B
133	233	VAL	B
134	234	LYS	B
135	235	HIS	B
136	236	ILE	B
137	237	LYS	B
138	238	PHE	B
139	239	SER	B
140	240	PHE	B
141	241	LEU	B
142	242	ARG	B
143	243	ASP	B
144	244	TRP	B
145	245	VAL	B
146	246	ASN	B
147	247	HIS	B
148	248	VAL	B
149	249	ARG	B
150	256	MET	B
151	257	PHE	B
152	258	THR	B
153	259	VAL	C
154	260	ALA	B
155	261	GLU	B
156	262	TYR	B
157	263	TRP	B
158	264	SER	B
159	265	TYR	B
160	270	LEU	B
161	271	GLU	B
162	272	ASN	B
163	273	TYR	B
164	274	LEU	B
165	275	ASN	B
166	280	ASN	B
167	281	HIS	B
168	282	SER	B

Continued on next page

Table S5 – *Continued from previous page*

Homology index	Residue Index	Residue abbreviation	Module
169	283	VAL	B
170	284	PHE	D
171	285	ASP	D
172	286	VAL	D
173	287	PRO	D
174	289	HIS	D
175	290	TYR	D
176	291	GLN	D
177	292	PHE	D
178	293	HIS	D
179	294	ALA	D
180	295	ALA	D
181	296	SER	D
182	302	TYR	D
183	303	ASP	A
184	304	MET	A
185	305	ARG	A
186	307	LEU	A
187	308	LEU	D
188	309	ASN	D
189	310	GLY	D
190	313	VAL	D
191	314	SER	B
192	315	LYS	B
193	316	HIS	B
194	317	PRO	B
195	318	LEU	B
196	319	LYS	B
197	320	SER	B
198	321	VAL	D
199	322	THR	D
200	323	PHE	D
201	324	VAL	A
202	325	ASP	D
203	326	ASN	D
204	327	HIS	D
205	328	ASP	D
206	329	THR	D
207	330	GLN	D
208	338	THR	D
209	339	VAL	D
210	340	GLN	D
211	341	THR	D
212	342	TRP	A
213	343	PHE	A
214	344	LYS	A

Continued on next page

Table S5 – *Continued from previous page*

Homology index	Residue Index	Residue abbreviation	Module
215	345	PRO	A
216	346	LEU	A
217	347	ALA	A
218	348	TYR	A
219	349	ALA	A
220	350	PHE	A
221	351	ILE	A
222	352	LEU	A
223	353	THR	A
224	354	ARG	A
225	356	SER	D
226	357	GLY	D
227	358	TYR	D
228	359	PRO	A
229	360	GLN	D
230	361	VAL	A
231	362	PHE	D
232	363	TYR	C
233	364	GLY	D
234	365	ASP	A
235	366	MET	C
236	367	TYR	C
237	368	GLY	D
238	369	THR	D
239	379	ALA	D
240	380	LEU	A
241	381	LYS	A
242	382	HIS	A
243	383	LYS	A
244	384	ILE	A
245	385	GLU	A
246	386	PRO	A
247	387	ILE	A
248	388	LEU	A
249	389	LYS	A
250	390	ALA	A
251	391	ARG	A
252	392	LYS	A
253	393	GLN	A
254	394	TYR	A
255	395	ALA	A
256	397	GLY	A
257	398	ALA	A
258	399	GLN	A
259	400	HIS	A
260	401	ASP	A

Continued on next page

Table S5 – *Continued from previous page*

Homology index	Residue Index	Residue abbreviation	Module
261	402	TYR	A
262	403	PHE	A
263	404	ASP	A
264	405	HIS	A
265	406	HIS	A
266	407	ASP	A
267	408	ILE	A
268	409	VAL	A
269	410	GLY	A
270	411	TRP	A
271	412	THR	A
272	413	ARG	A
273	414	GLU	A
274	415	GLY	A
275	424	LEU	A
276	425	ALA	A
277	426	ALA	A
278	427	LEU	A
279	428	ILE	A
280	429	THR	A
281	431	GLY	A
282	432	PRO	A
283	433	GLY	A
284	434	GLY	A
285	435	ALA	A
286	436	LYS	A
287	437	ARG	A
288	446	GLY	A
289	447	GLU	A
290	449	TRP	A
291	450	HIS	A
292	451	ASP	A
293	452	ILE	A
294	472	VAL	A
295	473	ASN	A
296	474	GLY	A
297	476	SER	A
298	477	VAL	A
299	478	SER	A
300	479	ILE	A
301	480	TYR	A
302	481	VAL	A

Table S6: Residues membership for the *B. subtilis* α-amylase (PDB code 1UA7, chain A)

Homology index	Residue Index	Residue abbreviation	Module
0	9	GLY	A
1	10	THR	A
2	11	ILE	B
3	12	LEU	B
4	13	HIS	B
5	14	ALA	C
6	15	TRP	C
7	16	ASN	C
8	17	TRP	C
9	18	SER	C
10	19	PHE	C
11	20	ASN	C
12	21	THR	C
13	22	LEU	C
14	23	LYS	C
15	24	HIS	C
16	25	ASN	C
17	26	MET	C
18	27	LYS	C
19	28	ASP	C
20	29	ILE	C
21	30	HIS	C
22	31	ASP	C
23	32	ALA	C
24	33	GLY	C
25	34	TYR	C
26	35	THR	C
27	36	ALA	C
28	37	ILE	C
29	38	GLN	C
30	39	THR	C
31	40	SER	C
32	41	PRO	C
33	42	ILE	C
34	43	ASN	C
35	44	GLN	C
36	45	VAL	C
37	46	LYS	C
38	48	GLY	C
39	59	TYR	C
40	60	TRP	C
41	61	LEU	C
42	62	TYR	C
43	63	GLN	C

Continued on next page

Table S6 – *Continued from previous page*

Homology index	Residue Index	Residue abbreviation	Module
44	64	PRO	C
45	65	THR	C
46	66	SER	C
47	67	TYR	C
48	68	GLN	C
49	69	ILE	C
50	70	GLY	C
51	72	ARG	C
52	73	TYR	C
53	74	LEU	C
54	75	GLY	C
55	76	THR	C
56	77	GLU	C
57	78	GLN	C
58	79	GLU	C
59	80	PHE	C
60	81	LYS	C
61	82	GLU	C
62	83	MET	C
63	84	CYS	C
64	85	ALA	C
65	86	ALA	C
66	87	ALA	C
67	88	GLU	C
68	89	GLU	C
69	90	TYR	C
70	91	GLY	C
71	92	ILE	C
72	93	LYS	C
73	94	VAL	C
74	95	ILE	C
75	96	VAL	C
76	97	ASP	C
77	98	ALA	C
78	99	VAL	C
79	100	ILE	C
80	101	ASN	C
81	102	HIS	C
82	103	THR	C
83	104	THR	C
84	105	PHE	C
85	106	ASP	C
86	111	SER	C
87	113	GLU	C
88	114	VAL	C
89	115	LYS	C

Continued on next page

Table S6 – *Continued from previous page*

**Homology index**	**Residue Index**	**Residue abbreviation**	**Module**
90	118	PRO	C
91	119	ASN	C
92	120	TRP	C
93	121	THR	C
94	122	HIS	B
95	139	ASN	B
96	140	SER	B
97	141	LEU	B
98	142	LEU	B
99	144	LEU	B
100	145	TYR	B
101	146	ASP	B
102	147	TRP	B
103	148	ASN	B
104	149	THR	B
105	150	GLN	B
106	151	ASN	B
107	152	THR	B
108	153	GLN	B
109	154	VAL	B
110	155	GLN	B
111	156	SER	B
112	157	TYR	B
113	158	LEU	B
114	159	LYS	B
115	160	ARG	B
116	161	PHE	B
117	162	LEU	B
118	163	GLU	B
119	164	ARG	B
120	165	ALA	B
121	166	LEU	B
122	167	ASN	B
123	168	ASP	C
124	169	GLY	C
125	170	ALA	C
126	171	ASP	C
127	172	GLY	C
128	173	PHE	B
129	174	ARG	D
130	175	PHE	B
131	176	ASP	B
132	177	ALA	B
133	178	ALA	B
134	179	LYS	B
135	180	HIS	B

Continued on next page

Table S6 – *Continued from previous page*

Homology index	Residue Index	Residue abbreviation	Module
136	181	ILE	B
137	182	GLU	B
138	191	SER	B
139	192	GLN	B
140	193	PHE	B
141	194	TRP	B
142	195	PRO	B
143	196	ASN	B
144	197	ILE	B
145	198	THR	B
146	199	ASN	B
147	200	THR	B
148	201	SER	B
149	202	ALA	B
150	203	GLU	B
151	204	PHE	B
152	205	GLN	B
153	206	TYR	C
154	207	GLY	B
155	208	GLU	B
156	209	ILE	B
157	210	LEU	B
158	211	GLN	B
159	212	ASP	B
160	217	ASP	B
161	218	ALA	B
162	219	ALA	B
163	220	TYR	B
164	221	ALA	B
165	222	ASN	B
166	223	TYR	B
167	224	MET	B
168	225	ASP	B
169	226	VAL	B
170	227	THR	D
171	228	ALA	D
172	229	SER	D
173	230	ASN	D
174	232	GLY	D
175	233	HIS	D
176	234	SER	D
177	235	ILE	D
178	236	ARG	D
179	237	SER	D
180	238	ALA	D
181	239	LEU	D

Continued on next page

Table S6 – *Continued from previous page*

Homology index	Residue Index	Residue abbreviation	Module
182	243	ASN	D
183	244	LEU	A
184	245	GLY	A
185	246	VAL	A
186	249	ILE	A
187	250	SER	D
188	251	HIS	D
189	252	TYR	D
190	253	ALA	D
191	254	SER	B
192	255	ASP	B
193	257	SER	B
194	258	ALA	B
195	259	ASP	B
196	260	LYS	B
197	261	LEU	B
198	262	VAL	D
199	263	THR	D
200	264	TRP	D
201	265	VAL	A
202	266	GLU	D
203	267	SER	D
204	268	HIS	D
205	269	ASP	D
206	270	THR	D
207	271	TYR	D
208	279	THR	D
209	280	TRP	D
210	281	MET	D
211	282	SER	D
212	284	ASP	A
213	285	ASP	A
214	286	ILE	A
215	287	ARG	A
216	288	LEU	A
217	289	GLY	A
218	290	TRP	A
219	291	ALA	A
220	292	VAL	A
221	293	ILE	A
222	294	ALA	A
223	295	SER	A
224	296	ARG	A
225	297	SER	D
226	299	SER	D
227	300	THR	D

Continued on next page

Table S6 – *Continued from previous page*

Homology index	Residue Index	Residue abbreviation	Module
228	301	PRO	A
229	302	LEU	D
230	303	PHE	A
231	304	PHE	D
232	305	SER	C
233	306	ARG	D
234	307	PRO	A
235	316	PHE	C
236	325	ARG	C
237	326	GLY	D
238	327	SER	D
239	328	ALA	D
240	331	GLU	A
241	332	ASP	A
242	333	GLN	A
243	334	ALA	A
244	335	ILE	A
245	336	THR	A
246	337	ALA	A
247	338	VAL	A
248	339	ASN	A
249	340	ARG	A
250	341	PHE	A
251	342	HIS	A
252	343	ASN	A
253	344	VAL	A
254	345	MET	A
255	346	ALA	A
256	347	GLY	A
257	348	GLN	A
258	349	PRO	A
259	350	GLU	A
260	351	GLU	A
261	352	LEU	A
262	353	SER	A
263	354	ASN	A
264	357	GLY	A
265	358	ASN	A
266	359	ASN	A
267	360	GLN	A
268	362	PHE	A
269	363	MET	A
270	364	ASN	A
271	365	GLN	A
272	366	ARG	A
273	367	GLY	A

Continued on next page

Table S6 – *Continued from previous page*

**Homology index**	**Residue Index**	**Residue abbreviation**	**Module**
274	368	SER	A
275	370	GLY	A
276	371	VAL	A
277	372	VAL	A
278	373	LEU	A
279	374	ALA	A
280	375	ASN	A
281	378	SER	A
282	379	SER	A
283	380	SER	A
284	381	VAL	A
285	383	ILE	A
286	384	ASN	A
287	385	THR	A
288	391	ASP	A
289	392	GLY	A
290	394	TYR	A
291	395	ASP	A
292	396	ASN	A
293	397	LYS	A
294	414	ILE	A
295	415	ASN	A
296	416	ALA	A
297	418	SER	A
298	419	VAL	A
299	420	ALA	A
300	421	VAL	A
301	422	LEU	A
302	423	TYR	A

Table S7: Residues membership for the *S. scrofa* α-amylase (PDB code 1PIF, chain A)

**Homology index**	**Residue Index**	**Residue abbreviation**	**Module**
0	11	THR	A
1	12	SER	A
2	13	ILE	B
3	14	VAL	B
4	15	HIS	B
5	16	LEU	C
6	17	PHE	C
7	18	GLU	C
8	19	TRP	C

Continued on next page

Table S7 – *Continued from previous page*

Homology index	Residue Index	Residue abbreviation	Module
9	20	ARG	C
10	21	TRP	C
11	22	VAL	C
12	23	ASP	C
13	24	ILE	C
14	25	ALA	C
15	26	LEU	C
16	27	GLU	C
17	28	CYS	C
18	29	GLU	C
19	30	ARG	C
20	32	LEU	C
21	33	GLY	C
22	34	PRO	C
23	35	LYS	C
24	36	GLY	C
25	37	PHE	C
26	38	GLY	C
27	39	GLY	C
28	40	VAL	C
29	41	GLN	C
30	42	VAL	C
31	43	SER	C
32	44	PRO	C
33	45	PRO	C
34	46	ASN	C
35	47	GLU	C
36	48	ASN	C
37	49	VAL	C
38	51	VAL	C
39	59	TRP	C
40	60	GLU	C
41	61	ARG	C
42	62	TYR	C
43	63	GLN	C
44	64	PRO	C
45	65	VAL	C
46	66	SER	C
47	67	TYR	C
48	68	LYS	C
49	69	LEU	C
50	70	CYS	C
51	71	THR	C
52	72	ARG	C
53	73	SER	C
54	74	GLY	C

Continued on next page

Table S7 – *Continued from previous page*

Homology index	Residue Index	Residue abbreviation	Module
55	75	ASN	C
56	76	GLU	C
57	77	ASN	C
58	78	GLU	C
59	79	PHE	C
60	80	ARG	C
61	81	ASP	C
62	82	MET	C
63	83	VAL	C
64	84	THR	C
65	85	ARG	C
66	86	CYS	C
67	87	ASN	C
68	88	ASN	C
69	89	VAL	C
70	90	GLY	C
71	91	VAL	C
72	92	ARG	C
73	93	ILE	C
74	94	TYR	C
75	95	VAL	C
76	96	ASP	C
77	97	ALA	C
78	98	VAL	C
79	99	ILE	C
80	100	ASN	C
81	101	HIS	C
82	102	MET	C
83	103	CYS	C
84	104	GLY	C
85	105	SER	C
86	126	PHE	C
87	130	PRO	C
88	131	TYR	C
89	132	SER	C
90	133	ALA	C
91	134	TRP	C
92	135	ASP	C
93	136	PHE	C
94	137	ASN	B
95	160	CYS	B
96	161	GLN	B
97	162	LEU	B
98	163	VAL	B
99	165	LEU	B
100	166	LEU	B

Continued on next page

Table S7 – *Continued from previous page*

Homology index	Residue Index	Residue abbreviation	Module
101	167	ASP	B
102	168	LEU	B
103	169	ALA	B
104	170	LEU	B
105	171	GLU	B
106	172	LYS	B
107	173	ASP	B
108	174	TYR	B
109	175	VAL	B
110	176	ARG	B
111	177	SER	B
112	178	MET	B
113	179	ILE	B
114	180	ALA	B
115	181	ASP	B
116	182	TYR	B
117	183	LEU	B
118	184	ASN	B
119	185	LYS	B
120	186	LEU	B
121	187	ILE	B
122	188	ASP	B
123	189	ILE	C
124	190	GLY	C
125	191	VAL	C
126	192	ALA	C
127	193	GLY	C
128	194	PHE	B
129	195	ARG	D
130	196	ILE	B
131	197	ASP	B
132	198	ALA	B
133	199	SER	B
134	200	LYS	B
135	201	HIS	B
136	202	MET	B
137	203	TRP	B
138	204	PRO	B
139	205	GLY	B
140	206	ASP	B
141	207	ILE	B
142	208	LYS	B
143	209	ALA	B
144	210	VAL	B
145	211	LEU	B
146	212	ASP	B

Continued on next page

Table S7 – *Continued from previous page*

Homology index	Residue Index	Residue abbreviation	Module
147	213	LYS	B
148	214	LEU	B
149	215	HIS	B
150	228	PRO	B
151	229	PHE	B
152	230	ILE	B
153	231	PHE	C
154	232	GLN	B
155	233	GLU	B
156	234	VAL	B
157	235	ILE	B
158	236	ASP	B
159	237	LEU	B
160	244	SER	B
161	245	SER	B
162	246	GLU	B
163	247	TYR	B
164	248	PHE	B
165	249	GLY	B
166	250	ASN	B
167	251	GLY	B
168	252	ARG	B
169	253	VAL	B
170	254	THR	D
171	255	GLU	D
172	256	PHE	D
173	257	LYS	D
174	259	GLY	D
175	260	ALA	D
176	261	LYS	D
177	262	LEU	D
178	263	GLY	D
179	264	THR	D
180	265	VAL	D
181	266	VAL	D
182	272	GLU	D
183	273	LYS	A
184	274	MET	A
185	275	SER	A
186	280	TRP	A
187	281	GLY	D
188	282	GLU	D
189	283	GLY	D
190	285	GLY	D
191	286	PHE	B
192	287	MET	B

Continued on next page

Table S7 – *Continued from previous page*

Homology index	Residue Index	Residue abbreviation	Module
193	288	PRO	B
194	289	SER	B
195	290	ASP	B
196	291	ARG	B
197	292	ALA	B
198	293	LEU	D
199	294	VAL	D
200	295	PHE	D
201	296	VAL	A
202	297	ASP	D
203	298	ASN	D
204	299	HIS	D
205	300	ASP	D
206	301	ASN	D
207	302	GLN	D
208	314	THR	D
209	315	PHE	D
210	316	TRP	D
211	317	ASP	D
212	319	ARG	A
213	320	LEU	A
214	321	TYR	A
215	322	LYS	A
216	323	VAL	A
217	324	ALA	A
218	325	VAL	A
219	326	GLY	A
220	327	PHE	A
221	328	MET	A
222	329	LEU	A
223	330	ALA	A
224	331	HIS	A
225	333	TYR	D
226	334	GLY	D
227	335	PHE	D
228	336	THR	A
229	337	ARG	D
230	338	VAL	A
231	339	MET	D
232	340	SER	C
233	341	SER	D
234	342	TYR	A
235	343	ARG	C
236	381	ASP	C
237	382	TRP	D
238	383	VAL	D

Continued on next page

Table S7 – *Continued from previous page*

**Homology index**	**Residue Index**	**Residue abbreviation**	**Module**
239	384	CYS	D
240	387	ARG	A
241	388	TRP	A
242	389	ARG	A
243	390	GLU	A
244	391	ILE	A
245	392	ARG	A
246	393	ASN	A
247	394	MET	A
248	395	VAL	A
249	396	TRP	A
250	397	PHE	A
251	398	ARG	A
252	399	ASN	A
253	400	VAL	A
254	401	VAL	A
255	402	ASP	A
256	403	GLY	A
257	404	GLN	A
258	405	PRO	A
259	406	PHE	A
260	408	ASN	A
261	409	TRP	A
262	410	TRP	A
263	411	ASP	A
264	413	GLY	A
265	414	SER	A
266	415	ASN	A
267	416	GLN	A
268	417	VAL	A
269	418	ALA	A
270	419	PHE	A
271	420	GLY	A
272	421	ARG	A
273	422	GLY	A
274	423	ASN	A
275	425	GLY	A
276	426	PHE	A
277	427	ILE	A
278	428	VAL	A
279	429	PHE	A
280	430	ASN	A
281	433	ASP	A
282	434	TRP	A
283	435	GLN	A
284	436	LEU	A

Continued on next page

Table S7 – *Continued from previous page*

Homology index	Residue Index	Residue abbreviation	Module
285	437	SER	A
286	438	SER	A
287	439	THR	A
288	445	PRO	A
289	446	GLY	A
290	449	TYR	A
291	450	CYS	A
292	451	ASN	A
293	452	VAL	A
294	479	ILE	A
295	480	SER	A
296	481	ASN	A
297	486	PRO	A
298	487	PHE	A
299	488	ILE	A
300	489	ALA	A
301	490	ILE	A
302	491	HIS	A

Table S8: Residues membership for the *S. scrofa* α-amylase (PDB code 1PPI, chain A)

Homology index	Residue Index	Residue abbreviation	Module
0	11	THR	A
1	12	SER	A
2	13	ILE	B
3	14	VAL	B
4	15	HIS	B
5	16	LEU	C
6	17	PHE	C
7	18	GLU	C
8	19	TRP	C
9	20	ARG	C
10	21	TRP	C
11	22	VAL	C
12	23	ASP	C
13	24	ILE	C
14	25	ALA	C
15	26	LEU	C
16	27	GLU	C
17	28	CYS	C
18	29	GLU	C
19	30	ARG	C

Continued on next page

Table S8 – *Continued from previous page*

Homology index	Residue Index	Residue abbreviation	Module
20	32	LEU	C
21	33	GLY	C
22	34	PRO	C
23	35	LYS	C
24	36	GLY	C
25	37	PHE	C
26	38	GLY	C
27	39	GLY	C
28	40	VAL	C
29	41	GLN	C
30	42	VAL	C
31	43	SER	C
32	44	PRO	C
33	45	PRO	C
34	46	ASN	C
35	47	GLU	C
36	48	ASN	C
37	49	VAL	C
38	51	VAL	C
39	59	TRP	C
40	60	GLU	C
41	61	ARG	C
42	62	TYR	C
43	63	GLN	C
44	64	PRO	C
45	65	VAL	C
46	66	SER	C
47	67	TYR	C
48	68	LYS	C
49	69	LEU	C
50	70	CYS	C
51	71	THR	C
52	72	ARG	C
53	73	SER	C
54	74	GLY	C
55	75	ASN	C
56	76	GLU	C
57	77	ASN	C
58	78	GLU	C
59	79	PHE	C
60	80	ARG	C
61	81	ASP	C
62	82	MET	C
63	83	VAL	C
64	84	THR	C
65	85	ARG	C

Continued on next page

Table S8 – *Continued from previous page*

Homology index	Residue Index	Residue abbreviation	Module
66	86	CYS	C
67	87	ASN	C
68	88	ASN	C
69	89	VAL	C
70	90	GLY	C
71	91	VAL	C
72	92	ARG	C
73	93	ILE	C
74	94	TYR	C
75	95	VAL	C
76	96	ASP	C
77	97	ALA	C
78	98	VAL	C
79	99	ILE	C
80	100	ASN	C
81	101	HIS	C
82	102	MET	C
83	103	CYS	C
84	104	GLY	C
85	105	SER	C
86	126	PHE	C
87	130	PRO	C
88	131	TYR	C
89	132	SER	C
90	133	ALA	C
91	134	TRP	C
92	135	ASP	C
93	136	PHE	C
94	137	ASN	B
95	160	CYS	B
96	161	GLN	B
97	162	LEU	B
98	163	VAL	B
99	165	LEU	B
100	166	LEU	B
101	167	ASP	B
102	168	LEU	B
103	169	ALA	B
104	170	LEU	B
105	171	GLU	B
106	172	LYS	B
107	173	ASP	B
108	174	TYR	B
109	175	VAL	B
110	176	ARG	B
111	177	SER	B

Continued on next page

Table S8 – *Continued from previous page*

Homology index	Residue Index	Residue abbreviation	Module
112	178	MET	B
113	179	ILE	B
114	180	ALA	B
115	181	ASP	B
116	182	TYR	B
117	183	LEU	B
118	184	ASN	B
119	185	LYS	B
120	186	LEU	B
121	187	ILE	B
122	188	ASP	B
123	189	ILE	C
124	190	GLY	C
125	191	VAL	C
126	192	ALA	C
127	193	GLY	C
128	194	PHE	B
129	195	ARG	D
130	196	ILE	B
131	197	ASP	B
132	198	ALA	B
133	199	SER	B
134	200	LYS	B
135	201	HIS	B
136	202	MET	B
137	203	TRP	B
138	204	PRO	B
139	205	GLY	B
140	206	ASP	B
141	207	ILE	B
142	208	LYS	B
143	209	ALA	B
144	210	VAL	B
145	211	LEU	B
146	212	ASP	B
147	213	LYS	B
148	214	LEU	B
149	215	HIS	B
150	228	PRO	B
151	229	PHE	B
152	230	ILE	B
153	231	PHE	C
154	232	GLN	B
155	233	GLU	B
156	234	VAL	B
157	235	ILE	B

Continued on next page

Table S8 – *Continued from previous page*

Homology index	Residue Index	Residue abbreviation	Module
158	236	ASP	B
159	237	LEU	B
160	244	SER	B
161	245	SER	B
162	246	GLU	B
163	247	TYR	B
164	248	PHE	B
165	249	GLY	B
166	250	ASN	B
167	251	GLY	B
168	252	ARG	B
169	253	VAL	B
170	254	THR	D
171	255	GLU	D
172	256	PHE	D
173	257	LYS	D
174	259	GLY	D
175	260	ALA	D
176	261	LYS	D
177	262	LEU	D
178	263	GLY	D
179	264	THR	D
180	265	VAL	D
181	266	VAL	D
182	272	GLU	D
183	273	LYS	A
184	274	MET	A
185	275	SER	A
186	280	TRP	A
187	281	GLY	D
188	282	GLU	D
189	283	GLY	D
190	285	GLY	D
191	286	PHE	B
192	287	MET	B
193	288	PRO	B
194	289	SER	B
195	290	ASP	B
196	291	ARG	B
197	292	ALA	B
198	293	LEU	D
199	294	VAL	D
200	295	PHE	D
201	296	VAL	A
202	297	ASP	D
203	298	ASN	D

Continued on next page

Table S8 – *Continued from previous page*

**Homology index**	**Residue Index**	**Residue abbreviation**	**Module**
204	299	HIS	D
205	300	ASP	D
206	301	ASN	D
207	302	GLN	D
208	314	THR	D
209	315	PHE	D
210	316	TRP	D
211	317	ASP	D
212	319	ARG	A
213	320	LEU	A
214	321	TYR	A
215	322	LYS	A
216	323	VAL	A
217	324	ALA	A
218	325	VAL	A
219	326	GLY	A
220	327	PHE	A
221	328	MET	A
222	329	LEU	A
223	330	ALA	A
224	331	HIS	A
225	333	TYR	D
226	334	GLY	D
227	335	PHE	D
228	336	THR	A
229	337	ARG	D
230	338	VAL	A
231	339	MET	D
232	340	SER	C
233	341	SER	D
234	342	TYR	A
235	343	ARG	C
236	381	ASP	C
237	382	TRP	D
238	383	VAL	D
239	384	CYS	D
240	387	ARG	A
241	388	TRP	A
242	389	ARG	A
243	390	GLU	A
244	391	ILE	A
245	392	ARG	A
246	393	ASN	A
247	394	MET	A
248	395	VAL	A
249	396	TRP	A

Continued on next page

Table S8 – *Continued from previous page*

Homology index	Residue Index	Residue abbreviation	Module
250	397	PHE	A
251	398	ARG	A
252	399	ASN	A
253	400	VAL	A
254	401	VAL	A
255	402	ASP	A
256	403	GLY	A
257	404	GLU	A
258	405	PRO	A
259	406	PHE	A
260	408	ASN	A
261	409	TRP	A
262	410	TRP	A
263	411	ASP	A
264	413	GLY	A
265	414	SER	A
266	415	ASN	A
267	416	GLN	A
268	417	VAL	A
269	418	ALA	A
270	419	PHE	A
271	420	GLY	A
272	421	ARG	A
273	422	GLY	A
274	423	ASN	A
275	425	GLY	A
276	426	PHE	A
277	427	ILE	A
278	428	VAL	A
279	429	PHE	A
280	430	ASN	A
281	433	ASP	A
282	434	TRP	A
283	435	GLN	A
284	436	LEU	A
285	437	SER	A
286	438	SER	A
287	439	THR	A
288	445	PRO	A
289	446	GLY	A
290	449	TYR	A
291	450	CYS	A
292	451	ASP	A
293	452	VAL	A
294	479	ILE	A
295	480	SER	A

Continued on next page

Table S8 – *Continued from previous page*

**Homology index**	**Residue Index**	**Residue abbreviation**	**Module**
296	481	ASN	A
297	486	PRO	A
298	487	PHE	A
299	488	ILE	A
300	489	ALA	A
301	490	ILE	A
302	491	HIS	A

Table S9: Residues membership for the *B. circulans* α-amylase (PDB code 1PJ9, chain A)

**Homology index**	**Residue Index**	**Residue abbreviation**	**Module**
0	15	ASP	A
1	16	VAL	A
2	17	ILE	B
3	18	TYR	B
4	19	GLN	B
5	20	ILE	C
6	21	PHE	C
7	22	ASN	C
8	52	GLY	C
9	53	ASP	C
10	54	TRP	C
11	55	GLN	C
12	56	GLY	C
13	57	ILE	C
14	58	ILE	C
15	59	ASN	C
16	60	LYS	C
17	61	ILE	C
18	62	ASN	C
19	63	ASP	C
20	66	LEU	C
21	67	THR	C
22	68	GLY	C
23	69	MET	C
24	70	GLY	C
25	71	VAL	C
26	72	THR	C
27	73	ALA	C
28	74	ILE	C
29	75	TRP	C
30	76	ILE	C

Continued on next page

Table S9 – *Continued from previous page*

Homology index	Residue Index	Residue abbreviation	Module
31	77	SER	C
32	78	GLN	C
33	79	PRO	C
34	80	VAL	C
35	81	GLU	C
36	82	ASN	C
37	83	ILE	C
38	95	THR	C
39	97	TYR	C
40	98	HIS	C
41	99	GLY	C
42	100	TYR	C
43	101	TRP	C
44	102	ALA	C
45	103	ARG	C
46	104	ASP	C
47	106	LYS	C
48	107	LYS	C
49	108	THR	C
50	109	ASN	C
51	110	PRO	C
52	111	ALA	C
53	112	TYR	C
54	113	GLY	C
55	114	THR	C
56	115	ILE	C
57	116	ALA	C
58	117	ASP	C
59	118	PHE	C
60	119	GLN	C
61	120	ASN	C
62	121	LEU	C
63	122	ILE	C
64	123	ALA	C
65	124	ALA	C
66	125	ALA	C
67	126	HIS	C
68	127	ALA	C
69	128	LYS	C
70	129	ASN	C
71	130	ILE	C
72	131	LYS	C
73	132	VAL	C
74	133	ILE	C
75	134	ILE	C
76	135	ASP	C

Continued on next page

Table S9 – *Continued from previous page*

Homology index	Residue Index	Residue abbreviation	Module
77	136	PHE	C
78	137	ALA	C
79	138	PRO	C
80	139	ASN	C
81	140	HIS	C
82	141	THR	C
83	142	SER	C
84	143	PRO	C
85	144	ALA	C
86	156	ARG	C
87	163	LEU	C
88	164	LEU	C
89	165	GLY	C
90	172	GLN	C
91	173	ASN	C
92	174	LEU	C
93	175	PHE	C
94	176	HIS	B
95	192	ARG	B
96	193	ASN	B
97	194	LEU	B
98	195	TYR	B
99	197	LEU	B
100	198	ALA	B
101	199	ASP	B
102	200	LEU	B
103	201	ASN	B
104	202	HIS	B
105	203	ASN	B
106	204	ASN	B
107	205	SER	B
108	206	THR	B
109	207	VAL	B
110	208	ASP	B
111	209	VAL	B
112	210	TYR	B
113	211	LEU	B
114	212	LYS	B
115	213	ASP	B
116	214	ALA	B
117	215	ILE	B
118	216	LYS	B
119	217	MET	B
120	218	TRP	B
121	219	LEU	B
122	220	ASP	B

Continued on next page

Table S9 – *Continued from previous page*

Homology index	Residue Index	Residue abbreviation	Module
123	221	LEU	C
124	222	GLY	C
125	223	ILE	C
126	224	ASP	C
127	225	GLY	C
128	226	ILE	B
129	227	ARG	D
130	228	MET	B
131	229	ASP	B
132	230	ALA	B
133	231	VAL	B
134	232	LYS	B
135	233	HIS	B
136	234	MET	B
137	235	PRO	B
138	236	PHE	B
139	237	GLY	B
140	238	TRP	B
141	239	GLN	B
142	240	LYS	B
143	241	SER	B
144	242	PHE	B
145	243	MET	B
146	244	ALA	B
147	245	ALA	B
148	246	VAL	B
149	247	ASN	B
150	252	VAL	B
151	253	PHE	B
152	254	THR	B
153	255	PHE	C
154	256	GLY	B
155	257	GLU	B
156	258	TRP	B
157	259	PHE	B
158	260	LEU	B
159	261	GLY	B
160	269	ASN	B
161	270	HIS	B
162	271	LYS	B
163	272	PHE	B
164	274	ASN	B
165	275	GLU	B
166	277	GLY	B
167	278	MET	B
168	279	SER	B

Continued on next page

Table S9 – *Continued from previous page*

Homology index	Residue Index	Residue abbreviation	Module
169	280	LEU	B
170	281	LEU	D
171	282	ASP	D
172	283	PHE	D
173	284	ARG	D
174	286	ALA	D
175	287	GLN	D
176	288	LYS	D
177	289	VAL	D
178	290	ARG	D
179	291	GLN	D
180	292	VAL	D
181	293	PHE	D
182	298	ASP	D
183	299	ASN	A
184	300	MET	A
185	301	TYR	A
186	306	MET	A
187	307	LEU	D
188	308	GLU	D
189	309	GLY	D
190	310	SER	D
191	311	ALA	B
192	312	ALA	B
193	316	GLN	B
194	317	VAL	B
195	318	ASP	B
196	319	ASP	B
197	320	GLN	B
198	321	VAL	D
199	322	THR	D
200	323	PHE	D
201	324	ILE	A
202	325	ASP	D
203	326	ASN	D
204	327	HIS	D
205	328	ASP	D
206	329	MET	D
207	330	GLU	D
208	331	ARG	D
209	332	PHE	D
210	333	HIS	D
211	334	ALA	D
212	339	ARG	A
213	341	LYS	A
214	342	LEU	A

Continued on next page

Table S9 – *Continued from previous page*

Homology index	Residue Index	Residue abbreviation	Module
215	343	GLU	A
216	344	GLN	A
217	345	ALA	A
218	346	LEU	A
219	347	ALA	A
220	348	PHE	A
221	349	THR	A
222	350	LEU	A
223	351	THR	A
224	352	SER	A
225	353	ARG	D
226	354	GLY	D
227	355	VAL	D
228	356	PRO	A
229	357	ALA	D
230	358	ILE	A
231	359	TYR	D
232	360	TYR	C
233	361	GLY	D
234	362	THR	A
235	376	ALA	C
236	377	ARG	C
237	378	ILE	D
238	379	PRO	D
239	381	PHE	D
240	387	ALA	A
241	388	TYR	A
242	389	GLN	A
243	390	VAL	A
244	391	ILE	A
245	392	GLN	A
246	393	LYS	A
247	394	LEU	A
248	395	ALA	A
249	396	PRO	A
250	397	LEU	A
251	398	ARG	A
252	399	LYS	A
253	400	SER	A
254	401	ASN	A
255	402	PRO	A
256	406	TYR	A
257	407	GLY	A
258	408	SER	A
259	409	THR	A
260	411	GLU	A

Continued on next page

Table S9 – *Continued from previous page*

**Homology index**	**Residue Index**	**Residue abbreviation**	**Module**
261	412	ARG	A
262	413	TRP	A
263	414	ILE	A
264	415	ASN	A
265	416	ASN	A
266	417	ASP	A
267	418	VAL	A
268	419	LEU	A
269	420	ILE	A
270	421	TYR	A
271	422	GLU	A
272	423	ARG	A
273	428	ASN	A
274	429	VAL	A
275	430	ALA	A
276	431	VAL	A
277	432	VAL	A
278	433	ALA	A
279	434	VAL	A
280	435	ASN	A
281	439	ASN	A
282	440	ALA	A
283	441	PRO	A
284	442	ALA	A
285	443	SER	A
286	444	ILE	A
287	445	SER	A
288	454	GLY	A
289	455	SER	A
290	457	ASN	A
291	458	ASP	A
292	459	VAL	A
293	460	LEU	A
294	483	ALA	A
295	484	ALA	A
296	485	GLY	A
297	486	GLY	A
298	487	THR	A
299	488	ALA	A
300	489	VAL	A
301	490	TRP	A
302	491	GLN	A

Table S10: Residues membership for the . *stearothermophilus maltose* α-amylase (PDB code 1QHP, chain A)

**Homology index**	**Residue Index**	**Residue abbreviation**	**Module**
0	9	ASP	A
1	10	VAL	A
2	11	ILE	B
3	12	TYR	B
4	13	GLN	B
5	14	ILE	C
6	15	ILE	C
7	16	ILE	C
8	49	GLY	C
9	50	ASP	C
10	51	LEU	C
11	52	GLU	C
12	53	GLY	C
13	54	VAL	C
14	55	ARG	C
15	56	GLN	C
16	57	LYS	C
17	58	LEU	C
18	59	PRO	C
19	60	TYR	C
20	61	LEU	C
21	62	LYS	C
22	63	GLN	C
23	64	LEU	C
24	65	GLY	C
25	66	VAL	C
26	67	THR	C
27	68	THR	C
28	69	ILE	C
29	70	TRP	C
30	71	LEU	C
31	72	SER	C
32	73	PRO	C
33	74	VAL	C
34	75	LEU	C
35	76	ASP	C
36	77	ASN	C
37	78	LEU	C
38	87	THR	C
39	89	TYR	C
40	90	HIS	C
41	91	GLY	C
42	92	TYR	C
43	93	TRP	C

Continued on next page

Table S10 – *Continued from previous page*

Homology index	Residue Index	Residue abbreviation	Module
44	94	THR	C
45	95	ARG	C
46	96	ASP	C
47	98	LYS	C
48	99	GLN	C
49	100	ILE	C
50	101	GLU	C
51	102	GLU	C
52	103	HIS	C
53	104	PHE	C
54	105	GLY	C
55	106	ASN	C
56	107	TRP	C
57	108	THR	C
58	109	THR	C
59	110	PHE	C
60	111	ASP	C
61	112	THR	C
62	113	LEU	C
63	114	VAL	C
64	115	ASN	C
65	116	ASP	C
66	117	ALA	C
67	118	HIS	C
68	119	GLN	C
69	120	ASN	C
70	121	GLY	C
71	122	ILE	C
72	123	LYS	C
73	124	VAL	C
74	125	ILE	C
75	126	VAL	C
76	127	ASP	C
77	128	PHE	C
78	129	VAL	C
79	130	PRO	C
80	131	ASN	C
81	132	HIS	C
82	133	SER	C
83	134	THR	C
84	135	PRO	C
85	136	PHE	C
86	148	ALA	C
87	155	TYR	C
88	156	MET	C
89	157	GLY	C

Continued on next page

Table S10 – *Continued from previous page*

Homology index	Residue Index	Residue abbreviation	Module
90	165	LYS	C
91	166	GLY	C
92	167	TYR	C
93	168	PHE	C
94	169	HIS	B
95	186	LYS	B
96	187	ASN	B
97	188	PHE	B
98	189	THR	B
99	196	LEU	B
100	197	ALA	B
101	198	ASP	B
102	199	LEU	B
103	200	SER	B
104	201	GLN	B
105	202	GLU	B
106	203	ASN	B
107	204	GLY	B
108	205	THR	B
109	206	ILE	B
110	207	ALA	B
111	208	GLN	B
112	209	TYR	B
113	210	LEU	B
114	211	THR	B
115	212	ASP	B
116	213	ALA	B
117	214	ALA	B
118	215	VAL	B
119	216	GLN	B
120	217	LEU	B
121	218	VAL	B
122	219	ALA	B
123	220	HIS	C
124	221	GLY	C
125	222	ALA	C
126	223	ASP	C
127	224	GLY	C
128	225	LEU	B
129	226	ARG	D
130	227	ILE	B
131	228	ASP	B
132	229	ALA	B
133	230	VAL	B
134	231	LYS	B
135	232	HIS	B

Continued on next page

Table S10 – *Continued from previous page*

Homology index	Residue Index	Residue abbreviation	Module
136	233	PHE	B
137	234	ASN	B
138	235	SER	B
139	236	GLY	B
140	237	PHE	B
141	238	SER	B
142	239	LYS	B
143	240	SER	B
144	241	LEU	B
145	242	ALA	B
146	243	ASP	B
147	244	LYS	B
148	245	LEU	B
149	246	TYR	B
150	251	ILE	B
151	252	PHE	B
152	253	LEU	B
153	254	VAL	C
154	255	GLY	B
155	256	GLU	B
156	257	TRP	B
157	258	TYR	B
158	259	GLY	B
159	260	ASP	B
160	270	LYS	B
161	271	VAL	B
162	272	ARG	B
163	273	TYR	B
164	275	ASN	B
165	276	ASN	B
166	278	GLY	B
167	279	VAL	B
168	280	ASN	B
169	281	VAL	B
170	282	LEU	D
171	283	ASP	D
172	284	PHE	D
173	285	ASP	D
174	287	ASN	D
175	288	THR	D
176	289	VAL	D
177	290	ILE	D
178	291	ARG	D
179	292	ASN	D
180	293	VAL	D
181	294	PHE	D

Continued on next page

Table S10 – *Continued from previous page*

Homology index	Residue Index	Residue abbreviation	Module
182	299	GLN	D
183	300	THR	A
184	301	MET	A
185	302	TYR	A
186	307	MET	A
187	308	VAL	D
188	309	ASN	D
189	310	GLN	D
190	311	THR	D
191	312	GLY	B
192	313	ASN	B
193	317	TYR	B
194	318	LYS	B
195	319	GLU	B
196	320	ASN	B
197	321	LEU	B
198	322	ILE	D
199	323	THR	D
200	324	PHE	D
201	325	ILE	A
202	326	ASP	D
203	327	ASN	D
204	328	HIS	D
205	329	ASP	D
206	330	MET	D
207	331	SER	D
208	332	ARG	D
209	333	PHE	D
210	334	LEU	D
211	335	SER	D
212	340	LYS	A
213	342	ASN	A
214	343	LEU	A
215	344	HIS	A
216	345	GLN	A
217	346	ALA	A
218	347	LEU	A
219	348	ALA	A
220	349	PHE	A
221	350	ILE	A
222	351	LEU	A
223	352	THR	A
224	353	SER	A
225	354	ARG	D
226	355	GLY	D
227	356	THR	D

Continued on next page

Table S10 – *Continued from previous page*

**Homology index**	**Residue Index**	**Residue abbreviation**	**Module**
228	357	PRO	A
229	358	SER	D
230	359	ILE	A
231	360	TYR	D
232	361	TYR	C
233	362	GLY	D
234	363	THR	A
235	377	GLY	C
236	378	MET	C
237	379	MET	D
238	380	PRO	D
239	382	PHE	D
240	388	ALA	A
241	389	PHE	A
242	390	LYS	A
243	391	GLU	A
244	392	VAL	A
245	393	SER	A
246	394	THR	A
247	395	LEU	A
248	396	ALA	A
249	397	GLY	A
250	398	LEU	A
251	399	ARG	A
252	400	ARG	A
253	401	ASN	A
254	402	ASN	A
255	403	ALA	A
256	407	TYR	A
257	408	GLY	A
258	409	THR	A
259	410	THR	A
260	412	GLN	A
261	413	ARG	A
262	414	TRP	A
263	415	ILE	A
264	416	ASN	A
265	417	ASN	A
266	418	ASP	A
267	419	VAL	A
268	420	TYR	A
269	421	ILE	A
270	422	TYR	A
271	423	GLU	A
272	424	ARG	A
273	429	ASP	A

Continued on next page

Table S10 – *Continued from previous page*

Homology index	Residue Index	Residue abbreviation	Module
274	430	VAL	A
275	431	VAL	A
276	432	LEU	A
277	433	VAL	A
278	434	ALA	A
279	435	ILE	A
280	436	ASN	A
281	440	GLN	A
282	441	SER	A
283	442	SER	A
284	443	TYR	A
285	444	SER	A
286	445	ILE	A
287	446	SER	A
288	455	GLY	A
289	456	SER	A
290	458	ALA	A
291	459	ASP	A
292	460	TYR	A
293	461	LEU	A
294	483	ALA	A
295	484	PRO	A
296	485	GLY	A
297	486	ALA	A
298	487	VAL	A
299	488	SER	A
300	489	VAL	A
301	490	TRP	A
302	491	GLN	A

Table S11: Residues membership for the *B. stearothermophilus maltose* α-amylase (PDB code 1QHO, chain A)

Homology index	Residue Index	Residue abbreviation	Module
0	9	ASP	A
1	10	VAL	A
2	11	ILE	B
3	12	TYR	B
4	13	GLN	B
5	14	ILE	C
6	15	ILE	C
7	16	ILE	C
8	49	GLY	C

Continued on next page

Table S11 – *Continued from previous page*

Homology index	Residue Index	Residue abbreviation	Module
9	50	ASP	C
10	51	LEU	C
11	52	GLU	C
12	53	GLY	C
13	54	VAL	C
14	55	ARG	C
15	56	GLN	C
16	57	LYS	C
17	58	LEU	C
18	59	PRO	C
19	60	TYR	C
20	61	LEU	C
21	62	LYS	C
22	63	GLN	C
23	64	LEU	C
24	65	GLY	C
25	66	VAL	C
26	67	THR	C
27	68	THR	C
28	69	ILE	C
29	70	TRP	C
30	71	LEU	C
31	72	SER	C
32	73	PRO	C
33	74	VAL	C
34	75	LEU	C
35	76	ASP	C
36	77	ASN	C
37	78	LEU	C
38	87	THR	C
39	89	TYR	C
40	90	HIS	C
41	91	GLY	C
42	92	TYR	C
43	93	TRP	C
44	94	THR	C
45	95	ARG	C
46	96	ASP	C
47	98	LYS	C
48	99	GLN	C
49	100	ILE	C
50	101	GLU	C
51	102	GLU	C
52	103	HIS	C
53	104	PHE	C
54	105	GLY	C

Continued on next page

Table S11 – *Continued from previous page*

Homology index	Residue Index	Residue abbreviation	Module
55	106	ASN	C
56	107	TRP	C
57	108	THR	C
58	109	THR	C
59	110	PHE	C
60	111	ASP	C
61	112	THR	C
62	113	LEU	C
63	114	VAL	C
64	115	ASN	C
65	116	ASP	C
66	117	ALA	C
67	118	HIS	C
68	119	GLN	C
69	120	ASN	C
70	121	GLY	C
71	122	ILE	C
72	123	LYS	C
73	124	VAL	C
74	125	ILE	C
75	126	VAL	C
76	127	ASP	C
77	128	PHE	C
78	129	VAL	C
79	130	PRO	C
80	131	ASN	C
81	132	HIS	C
82	133	SER	C
83	134	THR	C
84	135	PRO	C
85	136	PHE	C
86	148	ALA	C
87	155	TYR	C
88	156	MET	C
89	157	GLY	C
90	165	LYS	C
91	166	GLY	C
92	167	TYR	C
93	168	PHE	C
94	169	HIS	B
95	186	LYS	B
96	187	ASN	B
97	188	PHE	B
98	189	THR	B
99	196	LEU	B
100	197	ALA	B

Continued on next page

Table S11 – *Continued from previous page*

**Homology index**	**Residue Index**	**Residue abbreviation**	**Module**
101	198	ASP	B
102	199	LEU	B
103	200	SER	B
104	201	GLN	B
105	202	GLU	B
106	203	ASN	B
107	204	GLY	B
108	205	THR	B
109	206	ILE	B
110	207	ALA	B
111	208	GLN	B
112	209	TYR	B
113	210	LEU	B
114	211	THR	B
115	212	ASP	B
116	213	ALA	B
117	214	ALA	B
118	215	VAL	B
119	216	GLN	B
120	217	LEU	B
121	218	VAL	B
122	219	ALA	B
123	220	HIS	C
124	221	GLY	C
125	222	ALA	C
126	223	ASP	C
127	224	GLY	C
128	225	LEU	B
129	226	ARG	D
130	227	ILE	B
131	228	ASP	B
132	229	ALA	B
133	230	VAL	B
134	231	LYS	B
135	232	HIS	B
136	233	PHE	B
137	234	ASN	B
138	235	SER	B
139	236	GLY	B
140	237	PHE	B
141	238	SER	B
142	239	LYS	B
143	240	SER	B
144	241	LEU	B
145	242	ALA	B
146	243	ASP	B

Continued on next page

Table S11 – *Continued from previous page*

Homology index	Residue Index	Residue abbreviation	Module
147	244	LYS	B
148	245	LEU	B
149	246	TYR	B
150	251	ILE	B
151	252	PHE	B
152	253	LEU	B
153	254	VAL	C
154	255	GLY	B
155	256	GLU	B
156	257	TRP	B
157	258	TYR	B
158	259	GLY	B
159	260	ASP	B
160	270	LYS	B
161	271	VAL	B
162	272	ARG	B
163	273	TYR	B
164	275	ASN	B
165	276	ASN	B
166	278	GLY	B
167	279	VAL	B
168	280	ASN	B
169	281	VAL	B
170	282	LEU	D
171	283	ASP	D
172	284	PHE	D
173	285	ASP	D
174	287	ASN	D
175	288	THR	D
176	289	VAL	D
177	290	ILE	D
178	291	ARG	D
179	292	ASN	D
180	293	VAL	D
181	294	PHE	D
182	299	GLN	D
183	300	THR	A
184	301	MET	A
185	302	TYR	A
186	307	MET	A
187	308	VAL	D
188	309	ASN	D
189	310	GLN	D
190	311	THR	D
191	312	GLY	B
192	313	ASN	B

Continued on next page

Table S11 – *Continued from previous page*

**Homology index**	**Residue Index**	**Residue abbreviation**	**Module**
193	317	TYR	B
194	318	LYS	B
195	319	GLU	B
196	320	ASN	B
197	321	LEU	B
198	322	ILE	D
199	323	THR	D
200	324	PHE	D
201	325	ILE	A
202	326	ASP	D
203	327	ASN	D
204	328	HIS	D
205	329	ASP	D
206	330	MET	D
207	331	SER	D
208	332	ARG	D
209	333	PHE	D
210	334	LEU	D
211	335	SER	D
212	340	LYS	A
213	342	ASN	A
214	343	LEU	A
215	344	HIS	A
216	345	GLN	A
217	346	ALA	A
218	347	LEU	A
219	348	ALA	A
220	349	PHE	A
221	350	ILE	A
222	351	LEU	A
223	352	THR	A
224	353	SER	A
225	354	ARG	D
226	355	GLY	D
227	356	THR	D
228	357	PRO	A
229	358	SER	D
230	359	ILE	A
231	360	TYR	D
232	361	TYR	C
233	362	GLY	D
234	363	THR	A
235	377	GLY	C
236	378	MET	C
237	379	MET	D
238	380	PRO	D

Continued on next page

Table S11 – *Continued from previous page*

Homology index	Residue Index	Residue abbreviation	Module
239	382	PHE	D
240	388	ALA	A
241	389	PHE	A
242	390	LYS	A
243	391	GLU	A
244	392	VAL	A
245	393	SER	A
246	394	THR	A
247	395	LEU	A
248	396	ALA	A
249	397	GLY	A
250	398	LEU	A
251	399	ARG	A
252	400	ARG	A
253	401	ASN	A
254	402	ASN	A
255	403	ALA	A
256	407	TYR	A
257	408	GLY	A
258	409	THR	A
259	410	THR	A
260	412	GLN	A
261	413	ARG	A
262	414	TRP	A
263	415	ILE	A
264	416	ASN	A
265	417	ASN	A
266	418	ASP	A
267	419	VAL	A
268	420	TYR	A
269	421	ILE	A
270	422	TYR	A
271	423	GLU	A
272	424	ARG	A
273	429	ASP	A
274	430	VAL	A
275	431	VAL	A
276	432	LEU	A
277	433	VAL	A
278	434	ALA	A
279	435	ILE	A
280	436	ASN	A
281	440	GLN	A
282	441	SER	A
283	442	SER	A
284	443	TYR	A

Continued on next page

Table S11 – *Continued from previous page*

**Homology index**	**Residue Index**	**Residue abbreviation**	**Module**
285	444	SER	A
286	445	ILE	A
287	446	SER	A
288	455	GLY	A
289	456	SER	A
290	458	ALA	A
291	459	ASP	A
292	460	TYR	A
293	461	LEU	A
294	483	ALA	A
295	484	PRO	A
296	485	GLY	A
297	486	ALA	A
298	487	VAL	A
299	488	SER	A
300	489	VAL	A
301	490	TRP	A
302	491	GLN	A

Table S12: Residues membership for the *S. scrofa* α-amylase (PDB code 1UA3, chain A)

**Homology index**	**Residue Index**	**Residue abbreviation**	**Module**
0	11	THR	A
1	12	SER	A
2	13	ILE	B
3	14	VAL	B
4	15	HIS	B
5	16	LEU	C
6	17	PHE	C
7	18	GLU	C
8	19	TRP	C
9	20	ARG	C
10	21	TRP	C
11	22	VAL	C
12	23	ASP	C
13	24	ILE	C
14	25	ALA	C
15	26	LEU	C
16	27	GLU	C
17	28	CYS	C
18	29	GLU	C
19	30	ARG	C

Continued on next page

Table S12 – *Continued from previous page*

Homology index	Residue Index	Residue abbreviation	Module
20	32	LEU	C
21	33	GLY	C
22	34	PRO	C
23	35	LYS	C
24	36	GLY	C
25	37	PHE	C
26	38	GLY	C
27	39	GLY	C
28	40	VAL	C
29	41	GLN	C
30	42	VAL	C
31	43	SER	C
32	44	PRO	C
33	45	PRO	C
34	46	ASN	C
35	47	GLU	C
36	48	ASN	C
37	49	ILE	C
38	51	VAL	C
39	59	TRP	C
40	60	GLU	C
41	61	ARG	C
42	62	TYR	C
43	63	GLN	C
44	64	PRO	C
45	65	VAL	C
46	66	SER	C
47	67	TYR	C
48	68	LYS	C
49	69	LEU	C
50	70	CYS	C
51	71	THR	C
52	72	ARG	C
53	73	SER	C
54	74	GLY	C
55	75	ASN	C
56	76	GLU	C
57	77	ASN	C
58	78	GLU	C
59	79	PHE	C
60	80	ARG	C
61	81	ASP	C
62	82	MET	C
63	83	VAL	C
64	84	THR	C
65	85	ARG	C

Continued on next page

Table S12 – *Continued from previous page*

**Homology index**	**Residue Index**	**Residue abbreviation**	**Module**
66	86	CYS	C
67	87	ASN	C
68	88	ASN	C
69	89	VAL	C
70	90	GLY	C
71	91	VAL	C
72	92	ARG	C
73	93	ILE	C
74	94	TYR	C
75	95	VAL	C
76	96	ASP	C
77	97	ALA	C
78	98	VAL	C
79	99	ILE	C
80	100	ASN	C
81	101	HIS	C
82	102	MET	C
83	103	CYS	C
84	104	GLY	C
85	105	SER	C
86	126	PHE	C
87	130	PRO	C
88	131	TYR	C
89	132	SER	C
90	133	ALA	C
91	134	TRP	C
92	135	ASP	C
93	136	PHE	C
94	137	ASN	B
95	160	CYS	B
96	161	GLN	B
97	162	LEU	B
98	163	VAL	B
99	165	LEU	B
100	166	LEU	B
101	167	ASP	B
102	168	LEU	B
103	169	ALA	B
104	170	LEU	B
105	171	GLU	B
106	172	LYS	B
107	173	ASP	B
108	174	TYR	B
109	175	VAL	B
110	176	ARG	B
111	177	SER	B

Continued on next page

Table S12 – *Continued from previous page*

Homology index	Residue Index	Residue abbreviation	Module
112	178	MET	B
113	179	ILE	B
114	180	ALA	B
115	181	ASP	B
116	182	TYR	B
117	183	LEU	B
118	184	ASN	B
119	185	LYS	B
120	186	LEU	B
121	187	ILE	B
122	188	ASP	B
123	189	ILE	C
124	190	GLY	C
125	191	VAL	C
126	192	ALA	C
127	193	GLY	C
128	194	PHE	B
129	195	ARG	D
130	196	ILE	B
131	197	ASP	B
132	198	ALA	B
133	199	SER	B
134	200	LYS	B
135	201	HIS	B
136	202	MET	B
137	203	TRP	B
138	204	PRO	B
139	205	GLY	B
140	206	ASP	B
141	207	ILE	B
142	208	LYS	B
143	209	ALA	B
144	210	VAL	B
145	211	LEU	B
146	212	ASP	B
147	213	LYS	B
148	214	LEU	B
149	215	HIS	B
150	228	PRO	B
151	229	PHE	B
152	230	ILE	B
153	231	PHE	C
154	232	GLN	B
155	233	GLU	B
156	234	VAL	B
157	235	ILE	B

Continued on next page

Table S12 – *Continued from previous page*

Homology index	Residue Index	Residue abbreviation	Module
158	236	ASP	B
159	237	LEU	B
160	244	SER	B
161	245	SER	B
162	246	GLU	B
163	247	TYR	B
164	248	PHE	B
165	249	GLY	B
166	250	ASN	B
167	251	GLY	B
168	252	ARG	B
169	253	VAL	B
170	254	THR	D
171	255	GLU	D
172	256	PHE	D
173	257	LYS	D
174	259	GLY	D
175	260	ALA	D
176	261	LYS	D
177	262	LEU	D
178	263	GLY	D
179	264	THR	D
180	265	VAL	D
181	266	VAL	D
182	272	GLU	D
183	273	LYS	A
184	274	MET	A
185	275	SER	A
186	280	TRP	A
187	281	GLY	D
188	282	GLU	D
189	283	GLY	D
190	285	GLY	D
191	286	PHE	B
192	287	MET	B
193	288	PRO	B
194	289	SER	B
195	290	ASP	B
196	291	ARG	B
197	292	ALA	B
198	293	LEU	D
199	294	VAL	D
200	295	PHE	D
201	296	VAL	A
202	297	ASP	D
203	298	ASN	D

Continued on next page

Table S12 – *Continued from previous page*

Homology index	Residue Index	Residue abbreviation	Module
204	299	HIS	D
205	300	ASP	D
206	301	ASN	D
207	302	GLN	D
208	314	THR	D
209	315	PHE	D
210	316	TRP	D
211	317	ASP	D
212	319	ARG	A
213	320	LEU	A
214	321	TYR	A
215	322	LYS	A
216	323	ILE	A
217	324	ALA	A
218	325	VAL	A
219	326	GLY	A
220	327	PHE	A
221	328	MET	A
222	329	LEU	A
223	330	ALA	A
224	331	HIS	A
225	333	TYR	D
226	334	GLY	D
227	335	PHE	D
228	336	THR	A
229	337	ARG	D
230	338	VAL	A
231	339	MET	D
232	340	SER	C
233	341	SER	D
234	342	TYR	A
235	343	ARG	C
236	381	ASP	C
237	382	TRP	D
238	383	VAL	D
239	384	CYS	D
240	387	ARG	A
241	388	TRP	A
242	389	ARG	A
243	390	GLU	A
244	391	ILE	A
245	392	ARG	A
246	393	ASN	A
247	394	MET	A
248	395	VAL	A
249	396	TRP	A

Continued on next page

Table S12 – *Continued from previous page*

Homology index	Residue Index	Residue abbreviation	Module
250	397	PHE	A
251	398	ARG	A
252	399	ASN	A
253	400	VAL	A
254	401	VAL	A
255	402	ASP	A
256	403	GLY	A
257	404	GLN	A
258	405	PRO	A
259	406	PHE	A
260	408	ASN	A
261	409	TRP	A
262	410	TRP	A
263	411	ASP	A
264	413	GLY	A
265	414	SER	A
266	415	ASN	A
267	416	GLN	A
268	417	VAL	A
269	418	ALA	A
270	419	PHE	A
271	420	GLY	A
272	421	ARG	A
273	422	GLY	A
274	423	ASN	A
275	425	GLY	A
276	426	PHE	A
277	427	ILE	A
278	428	VAL	A
279	429	PHE	A
280	430	ASN	A
281	433	ASP	A
282	434	TRP	A
283	435	GLN	A
284	436	LEU	A
285	437	SER	A
286	438	SER	A
287	439	THR	A
288	445	PRO	A
289	446	GLY	A
290	449	TYR	A
291	450	CYS	A
292	451	ASP	A
293	452	VAL	A
294	479	ILE	A
295	480	SER	A

Continued on next page

Table S12 – *Continued from previous page*

**Homology index**	**Residue Index**	**Residue abbreviation**	**Module**
296	481	ASN	A
297	486	PRO	A
298	487	PHE	A
299	488	ILE	A
300	489	ALA	A
301	490	ILE	A
302	491	HIS	A

Table S13: Residues membership for the *P. haloplanctis* α-amylase (PDB code 1G94, chain A)

**Homology index**	**Residue Index**	**Residue abbreviation**	**Module**
0	3	THR	A
1	4	THR	A
2	5	PHE	B
3	6	VAL	B
4	7	HIS	B
5	8	LEU	C
6	9	PHE	C
7	10	GLU	C
8	11	TRP	C
9	12	ASN	C
10	13	TRP	C
11	14	GLN	C
12	15	ASP	C
13	16	VAL	C
14	17	ALA	C
15	18	GLN	C
16	19	GLU	C
17	20	CYS	C
18	21	GLU	C
19	22	GLN	C
20	24	LEU	C
21	25	GLY	C
22	26	PRO	C
23	27	LYS	C
24	28	GLY	C
25	29	TYR	C
26	30	ALA	C
27	31	ALA	C
28	32	VAL	C
29	33	GLN	C
30	34	VAL	C

Continued on next page

Table S13 – *Continued from previous page*

**Homology index**	**Residue Index**	**Residue abbreviation**	**Module**
31	35	SER	C
32	36	PRO	C
33	37	PRO	C
34	38	ASN	C
35	39	GLU	C
36	40	HIS	C
37	41	ILE	C
38	43	GLY	C
39	47	TRP	C
40	48	THR	C
41	49	ARG	C
42	50	TYR	C
43	51	GLN	C
44	52	PRO	C
45	53	VAL	C
46	54	SER	C
47	55	TYR	C
48	56	GLU	C
49	57	LEU	C
50	58	GLN	C
51	59	SER	C
52	60	ARG	C
53	61	GLY	C
54	62	GLY	C
55	63	ASN	C
56	64	ARG	C
57	65	ALA	C
58	66	GLN	C
59	67	PHE	C
60	68	ILE	C
61	69	ASP	C
62	70	MET	C
63	71	VAL	C
64	72	ASN	C
65	73	ARG	C
66	74	CYS	C
67	75	SER	C
68	76	ALA	C
69	77	ALA	C
70	78	GLY	C
71	79	VAL	C
72	80	ASP	C
73	81	ILE	C
74	82	TYR	C
75	83	VAL	C
76	84	ASP	C

Continued on next page

Table S13 – *Continued from previous page*

Homology index	Residue Index	Residue abbreviation	Module
77	85	THR	C
78	86	LEU	C
79	87	ILE	C
80	88	ASN	C
81	89	HIS	C
82	90	MET	C
83	91	ALA	C
84	92	ALA	C
85	93	GLY	C
86	108	PHE	C
87	110	ILE	C
88	111	TYR	C
89	112	SER	C
90	113	PRO	C
91	114	GLN	C
92	115	ASP	C
93	116	PHE	C
94	117	HIS	B
95	137	CYS	B
96	138	GLU	B
97	139	LEU	B
98	140	VAL	B
99	142	LEU	B
100	143	ALA	B
101	144	ASP	B
102	145	LEU	B
103	146	ASP	B
104	147	THR	B
105	148	ALA	B
106	149	SER	B
107	150	ASN	B
108	151	TYR	B
109	152	VAL	B
110	153	GLN	B
111	154	ASN	B
112	155	THR	B
113	156	ILE	B
114	157	ALA	B
115	158	ALA	B
116	159	TYR	B
117	160	ILE	B
118	161	ASN	B
119	162	ASP	B
120	163	LEU	B
121	164	GLN	B
122	165	ALA	B

Continued on next page

Table S13 – *Continued from previous page*

Homology index	Residue Index	Residue abbreviation	Module
123	166	ILE	C
124	167	GLY	C
125	168	VAL	C
126	169	LYS	C
127	170	GLY	C
128	171	PHE	B
129	172	ARG	D
130	173	PHE	B
131	174	ASP	B
132	175	ALA	B
133	176	SER	B
134	177	LYS	B
135	178	HIS	B
136	179	VAL	B
137	180	ALA	B
138	181	ALA	B
139	182	SER	B
140	183	ASP	B
141	184	ILE	B
142	185	GLN	B
143	186	SER	B
144	187	LEU	B
145	188	MET	B
146	189	ALA	B
147	190	LYS	B
148	191	VAL	B
149	192	ASN	B
150	195	PRO	B
151	196	VAL	B
152	197	VAL	B
153	198	PHE	C
154	199	GLN	B
155	200	GLU	B
156	201	VAL	B
157	202	ILE	B
158	203	ASP	B
159	204	GLN	B
160	211	ALA	B
161	212	SER	B
162	213	GLU	B
163	214	TYR	B
164	215	LEU	B
165	216	SER	B
166	217	THR	B
167	218	GLY	B
168	219	LEU	B

Continued on next page

Table S13 – *Continued from previous page*

Homology index	Residue Index	Residue abbreviation	Module
169	220	VAL	B
170	221	THR	D
171	222	GLU	D
172	223	PHE	D
173	224	LYS	D
174	226	SER	D
175	227	THR	D
176	228	GLU	D
177	229	LEU	D
178	230	GLY	D
179	231	ASN	D
180	232	THR	D
181	233	PHE	D
182	236	GLY	D
183	237	SER	A
184	238	LEU	A
185	239	ALA	A
186	244	PHE	A
187	245	GLY	D
188	246	GLU	D
189	247	GLY	D
190	249	GLY	D
191	250	PHE	B
192	251	MET	B
193	252	PRO	B
194	253	SER	B
195	254	SER	B
196	255	SER	B
197	256	ALA	B
198	257	VAL	D
199	258	VAL	D
200	259	PHE	D
201	260	VAL	A
202	261	ASP	D
203	262	ASN	D
204	263	HIS	D
205	264	ASP	D
206	265	ASN	D
207	266	GLN	D
208	277	THR	D
209	278	PHE	D
210	279	GLU	D
211	280	ASP	D
212	282	ARG	A
213	283	LEU	A
214	284	TYR	A

Continued on next page

Table S13 – *Continued from previous page*

Homology index	Residue Index	Residue abbreviation	Module
215	285	ASP	A
216	286	LEU	A
217	287	ALA	A
218	288	ASN	A
219	289	VAL	A
220	290	PHE	A
221	291	MET	A
222	292	LEU	A
223	293	ALA	A
224	294	TYR	A
225	296	TYR	D
226	297	GLY	D
227	298	TYR	D
228	299	PRO	A
229	300	LYS	D
230	301	VAL	A
231	302	MET	D
232	303	SER	C
233	304	SER	D
234	305	TYR	A
235	306	ASP	C
236	332	ASN	C
237	333	TRP	D
238	334	LYS	D
239	335	CYS	D
240	338	ARG	A
241	339	TRP	A
242	340	SER	A
243	341	TYR	A
244	342	ILE	A
245	343	ALA	A
246	344	GLY	A
247	345	GLY	A
248	346	VAL	A
249	347	ASP	A
250	348	PHE	A
251	349	ARG	A
252	350	ASN	A
253	351	ASN	A
254	352	THR	A
255	353	ALA	A
256	355	ASN	A
257	356	TRP	A
258	357	ALA	A
259	358	VAL	A
260	360	ASN	A

Continued on next page

Table S13 – *Continued from previous page*

Homology index	Residue Index	Residue abbreviation	Module
261	361	TRP	A
262	362	TRP	A
263	363	ASP	A
264	365	THR	A
265	366	ASN	A
266	367	ASN	A
267	368	GLN	A
268	369	ILE	A
269	370	SER	A
270	371	PHE	A
271	372	GLY	A
272	373	ARG	A
273	374	GLY	A
274	375	SER	A
275	377	GLY	A
276	378	HIS	A
277	379	MET	A
278	380	ALA	A
279	381	ILE	A
280	382	ASN	A
281	385	ASP	A
282	386	SER	A
283	387	THR	A
284	388	LEU	A
285	389	THR	A
286	390	ALA	A
287	391	THR	A
288	397	ALA	A
289	398	SER	A
290	401	TYR	A
291	402	CYS	A
292	403	ASN	A
293	404	VAL	A
294	433	ILE	A
295	434	GLY	A
296	435	ALA	A
297	437	ASP	A
298	438	ALA	A
299	439	MET	A
300	440	ALA	A
301	441	ILE	A
302	442	HIS	A

Table S14: Residues membership for the *N. polysaccharea* α-amylase (PDB code 1JG9, chain A)

Homology index	Residue Index	Residue abbreviation	Module
0	98	GLN	A
1	99	VAL	A
2	100	GLY	B
3	101	GLY	B
4	102	VAL	B
5	103	CYS	C
6	104	TYR	C
7	105	VAL	C
8	110	GLY	C
9	111	ASP	C
10	112	LEU	C
11	113	LYS	C
12	114	GLY	C
13	115	LEU	C
14	116	LYS	C
15	117	ASP	C
16	118	LYS	C
17	119	ILE	C
18	120	PRO	C
19	121	TYR	C
20	122	PHE	C
21	123	GLN	C
22	124	GLU	C
23	125	LEU	C
24	126	GLY	C
25	127	LEU	C
26	128	THR	C
27	129	TYR	C
28	130	LEU	C
29	131	HIS	C
30	132	LEU	C
31	133	MET	C
32	134	PRO	C
33	135	LEU	C
34	136	PHE	C
35	137	LYS	C
36	138	CYS	C
37	139	PRO	C
38	141	GLY	C
39	144	ASP	C
40	145	GLY	C
41	146	GLY	C
42	147	TYR	C
43	148	ALA	C

Continued on next page

Table S14 – *Continued from previous page*

Homology index	Residue Index	Residue abbreviation	Module
44	149	VAL	C
45	150	SER	C
46	151	SER	C
47	153	ARG	C
48	154	ASP	C
49	155	VAL	C
50	156	ASN	C
51	157	PRO	C
52	158	ALA	C
53	159	LEU	C
54	160	GLY	C
55	161	THR	C
56	162	ILE	C
57	163	GLY	C
58	164	ASP	C
59	165	LEU	C
60	166	ARG	C
61	167	GLU	C
62	168	VAL	C
63	169	ILE	C
64	170	ALA	C
65	171	ALA	C
66	172	LEU	C
67	173	HIS	C
68	174	GLU	C
69	175	ALA	C
70	176	GLY	C
71	177	ILE	C
72	178	SER	C
73	179	ALA	C
74	180	VAL	C
75	181	VAL	C
76	182	ASP	C
77	183	PHE	C
78	184	ILE	C
79	185	PHE	C
80	186	ASN	C
81	187	HIS	C
82	188	THR	C
83	189	SER	C
84	190	ASN	C
85	191	GLU	C
86	192	HIS	C
87	193	GLU	C
88	194	TRP	C
89	195	ALA	C

Continued on next page

Table S14 – *Continued from previous page*

Homology index	Residue Index	Residue abbreviation	Module
90	206	ASP	C
91	207	ASN	C
92	208	PHE	C
93	209	TYR	C
94	210	TYR	B
95	247	TRP	B
96	248	THR	B
97	249	THR	B
98	250	PHE	B
99	254	GLN	B
100	255	TRP	B
101	256	ASP	B
102	257	LEU	B
103	258	ASN	B
104	259	TYR	B
105	260	SER	B
106	261	ASN	B
107	262	PRO	B
108	263	TRP	B
109	264	VAL	B
110	265	PHE	B
111	266	ARG	B
112	267	ALA	B
113	268	MET	B
114	269	ALA	B
115	270	GLY	B
116	271	GLU	B
117	272	MET	B
118	273	LEU	B
119	274	PHE	B
120	275	LEU	B
121	276	ALA	B
122	277	ASN	B
123	278	LEU	C
124	279	GLY	C
125	280	VAL	C
126	281	ASP	C
127	282	ILE	C
128	283	LEU	B
129	284	ARG	D
130	285	MET	B
131	286	ASP	B
132	287	ALA	B
133	288	VAL	B
134	289	ALA	B
135	290	PHE	B

Continued on next page

Table S14 – *Continued from previous page*

Homology index	Residue Index	Residue abbreviation	Module
136	291	ILE	B
137	292	TRP	B
138	306	HIS	B
139	307	ALA	B
140	308	LEU	B
141	309	ILE	B
142	310	ARG	B
143	311	ALA	B
144	312	PHE	B
145	313	ASN	B
146	314	ALA	B
147	315	VAL	B
148	316	MET	B
149	317	ARG	B
150	323	VAL	B
151	324	PHE	B
152	325	PHE	B
153	326	LYS	C
154	327	SER	B
155	328	GLU	B
156	329	ALA	B
157	330	ILE	B
158	331	VAL	B
159	332	HIS	B
160	336	VAL	B
161	337	VAL	B
162	338	GLN	B
163	339	TYR	B
164	340	ILE	B
165	341	GLY	B
166	345	CYS	B
167	346	GLN	B
168	347	ILE	B
169	348	GLY	B
170	349	TYR	D
171	350	ASN	D
172	351	PRO	D
173	352	LEU	D
174	354	MET	D
175	355	ALA	D
176	356	LEU	D
177	357	LEU	D
178	358	TRP	D
179	359	ASN	D
180	360	THR	D
181	361	LEU	D

Continued on next page

Table S14 – *Continued from previous page*

Homology index	Residue Index	Residue abbreviation	Module
182	364	ARG	D
183	365	GLU	A
184	366	VAL	A
185	367	ASN	A
186	372	ALA	A
187	373	LEU	D
188	374	THR	D
189	375	TYR	D
190	376	ARG	D
191	377	HIS	B
192	378	ASN	B
193	381	GLU	B
194	382	HIS	B
195	383	THR	B
196	384	ALA	B
197	385	TRP	B
198	386	VAL	D
199	387	ASN	D
200	388	TYR	D
201	389	VAL	A
202	390	ARG	D
203	391	SER	D
204	392	HIS	D
205	393	ASP	D
206	394	ASP	D
207	448	SER	D
208	450	THR	D
209	451	ALA	D
210	452	ALA	D
211	453	ALA	D
212	464	ALA	A
213	467	ARG	A
214	468	ILE	A
215	469	LYS	A
216	470	LEU	A
217	471	LEU	A
218	472	TYR	A
219	473	SER	A
220	474	ILE	A
221	475	ALA	A
222	476	LEU	A
223	477	SER	A
224	478	THR	A
225	479	GLY	D
226	480	GLY	D
227	481	LEU	D

Continued on next page

Table S14 – *Continued from previous page*

Homology index	Residue Index	Residue abbreviation	Module
228	482	PRO	A
229	483	LEU	D
230	484	ILE	A
231	485	TYR	D
232	486	LEU	C
233	487	GLY	D
234	488	ASP	A
235	514	PRO	C
236	515	ARG	C
237	516	TYR	D
238	517	ASN	D
239	521	TYR	D
240	534	ILE	A
241	535	TYR	A
242	536	GLN	A
243	537	ASP	A
244	538	LEU	A
245	539	ARG	A
246	540	HIS	A
247	541	MET	A
248	542	ILE	A
249	543	ALA	A
250	544	VAL	A
251	545	ARG	A
252	546	GLN	A
253	547	SER	A
254	548	ASN	A
255	549	PRO	A
256	553	GLY	A
257	554	GLY	A
258	555	ARG	A
259	556	LEU	A
260	558	THR	A
261	559	PHE	A
262	560	ASN	A
263	561	THR	A
264	563	ASN	A
265	564	LYS	A
266	565	HIS	A
267	566	ILE	A
268	567	ILE	A
269	568	GLY	A
270	569	TYR	A
271	570	ILE	A
272	571	ARG	A
273	573	ASN	A

Continued on next page

Table S14 – *Continued from previous page*

Homology index	Residue Index	Residue abbreviation	Module
274	574	ALA	A
275	575	LEU	A
276	576	LEU	A
277	577	ALA	A
278	578	PHE	A
279	579	GLY	A
280	580	ASN	A
281	584	TYR	A
282	585	PRO	A
283	586	GLN	A
284	587	THR	A
285	588	VAL	A
286	589	THR	A
287	590	ALA	A
288	599	LYS	A
289	600	ALA	A
290	601	HIS	A
291	602	ASP	A
292	603	LEU	A
293	604	ILE	A
294	617	LEU	A
295	618	GLN	A
296	619	PRO	A
297	621	GLN	A
298	622	VAL	A
299	623	MET	A
300	624	TRP	A
301	625	LEU	A
302	626	GLU	A

Table S15: Residues membership for the *T. maritima* α-amylase (PDB code 1GJU, chain A)

Homology index	Residue Index	Residue abbreviation	Module
0	84	SER	A
1	85	VAL	A
2	86	VAL	B
3	87	TYR	B
4	88	GLY	B
5	89	SER	C
6	90	LEU	C
7	91	PRO	C
8	117	GLY	C

Continued on next page

Table S15 – *Continued from previous page*

Homology index	Residue Index	Residue abbreviation	Module
9	118	THR	C
10	119	PHE	C
11	120	PHE	C
12	121	LYS	C
13	122	MET	C
14	123	MET	C
15	124	LEU	C
16	125	LEU	C
17	126	LEU	C
18	127	PRO	C
19	128	PHE	C
20	129	VAL	C
21	130	LYS	C
22	131	SER	C
23	132	LEU	C
24	133	GLY	C
25	134	ALA	C
26	135	ASP	C
27	136	ALA	C
28	137	ILE	C
29	138	TYR	C
30	139	LEU	C
31	140	LEU	C
32	141	PRO	C
33	142	VAL	C
34	143	SER	C
35	144	ARG	C
36	145	MET	C
37	146	SER	C
38	154	ALA	C
39	155	PRO	C
40	156	SER	C
41	157	PRO	C
42	158	TYR	C
43	159	SER	C
44	160	VAL	C
45	161	LYS	C
46	162	ASN	C
47	164	MET	C
48	165	GLU	C
49	166	LEU	C
50	167	ASP	C
51	168	GLU	C
52	169	ARG	C
53	170	TYR	C
54	171	HIS	C

Continued on next page

Table S15 – *Continued from previous page*

Homology index	Residue Index	Residue abbreviation	Module
55	172	ASP	C
56	181	ASP	C
57	182	GLU	C
58	183	GLU	C
59	184	PHE	C
60	185	LYS	C
61	186	ALA	C
62	187	PHE	C
63	188	VAL	C
64	189	GLU	C
65	190	ALA	C
66	191	CYS	C
67	192	HIS	C
68	193	ILE	C
69	194	LEU	C
70	195	GLY	C
71	196	ILE	C
72	197	ARG	C
73	198	VAL	C
74	199	ILE	C
75	200	LEU	C
76	201	ASP	C
77	202	PHE	C
78	203	ILE	C
79	204	PRO	C
80	205	ARG	C
81	206	THR	C
82	207	ALA	C
83	208	ALA	C
84	209	ARG	C
85	210	ASP	C
86	211	SER	C
87	212	ASP	C
88	213	LEU	C
89	214	ILE	C
90	218	PRO	C
91	219	ASP	C
92	220	TRP	C
93	221	PHE	C
94	222	TYR	B
95	302	PRO	B
96	303	GLY	B
97	304	PHE	B
98	305	SER	B
99	318	VAL	B
100	319	THR	B

Continued on next page

Table S15 – *Continued from previous page*

Homology index	Residue Index	Residue abbreviation	Module
101	320	PHE	B
102	321	LEU	B
103	322	ARG	B
104	323	LEU	B
105	324	TYR	B
106	359	ASN	B
107	360	ARG	B
108	361	GLU	B
109	362	LEU	B
110	363	TRP	B
111	364	GLU	B
112	365	TYR	B
113	366	LEU	B
114	367	ALA	B
115	368	GLY	B
116	369	VAL	B
117	370	ILE	B
118	371	PRO	B
119	372	HIS	B
120	373	TYR	B
121	374	GLN	B
122	375	LYS	B
123	377	TYR	C
124	378	GLY	C
125	379	ILE	C
126	380	ASP	C
127	381	GLY	C
128	382	ALA	B
129	383	ARG	D
130	384	LEU	B
131	385	ASP	B
132	386	MET	B
133	387	GLY	B
134	388	HIS	B
135	389	ALA	B
136	390	LEU	B
137	391	PRO	B
138	392	LYS	B
139	393	GLU	B
140	394	LEU	B
141	395	LEU	B
142	396	ASP	B
143	397	LEU	B
144	398	ILE	B
145	399	ILE	B
146	400	LYS	B

Continued on next page

Table S15 – *Continued from previous page*

Homology index	Residue Index	Residue abbreviation	Module
147	401	ASN	B
148	402	VAL	B
149	403	LYS	B
150	409	PHE	B
151	410	VAL	B
152	411	MET	B
153	412	ILE	C
154	413	ALA	B
155	414	GLU	B
156	415	GLU	B
157	416	LEU	B
158	417	ASP	B
159	418	MET	B
160	421	ASP	B
161	422	LYS	B
162	423	ALA	B
163	424	SER	B
164	425	LYS	B
165	426	GLU	B
166	429	TYR	B
167	430	ASP	B
168	431	VAL	B
169	432	ILE	B
170	433	LEU	D
171	434	GLY	D
172	435	SER	D
173	436	SER	D
174	437	TRP	D
175	438	TYR	D
176	439	PHE	D
177	440	ALA	D
178	441	GLY	D
179	442	ARG	D
180	443	VAL	D
181	444	GLU	D
182	445	GLU	D
183	446	ILE	A
184	447	GLY	A
185	448	LYS	A
186	449	LEU	A
187	450	PRO	D
188	451	ASP	D
189	452	ILE	D
190	453	ALA	D
191	454	GLU	B
192	455	GLU	B

Continued on next page

Table S15 – *Continued from previous page*

Homology index	Residue Index	Residue abbreviation	Module
193	456	LEU	B
194	457	VAL	B
195	458	LEU	B
196	459	PRO	B
197	460	PHE	B
198	461	LEU	D
199	462	ALA	D
200	463	SER	D
201	464	VAL	A
202	465	GLU	D
203	466	THR	D
204	467	PRO	D
205	468	ASP	D
206	469	THR	D
207	470	PRO	D
208	471	ARG	D
209	472	ILE	D
210	473	ALA	D
211	474	THR	D
212	479	SER	A
213	481	MET	A
214	482	LYS	A
215	483	LYS	A
216	484	LEU	A
217	485	ALA	A
218	486	PRO	A
219	487	PHE	A
220	488	VAL	A
221	489	THR	A
222	490	TYR	A
223	491	PHE	A
224	492	LEU	A
225	493	PRO	D
226	495	SER	D
227	496	ILE	D
228	497	PRO	A
229	498	TYR	D
230	499	VAL	A
231	500	ASN	D
232	501	THR	C
233	502	GLY	D
234	503	GLN	A
235	541	TYR	C
236	542	VAL	C
237	543	LEU	D
238	544	HIS	D

Continued on next page

Table S15 – *Continued from previous page*

Homology index	Residue Index	Residue abbreviation	Module
239	545	TRP	D
240	552	VAL	A
241	553	LEU	A
242	554	ASN	A
243	555	PHE	A
244	556	ILE	A
245	557	LYS	A
246	558	LYS	A
247	559	LEU	A
248	560	ILE	A
249	561	LYS	A
250	562	VAL	A
251	563	ARG	A
252	564	HIS	A
253	565	GLU	A
254	566	PHE	A
255	567	LEU	A
256	572	ASN	A
257	573	GLY	A
258	574	LYS	A
259	575	PHE	A
260	576	GLU	A
261	577	ASN	A
262	578	LEU	A
263	579	THR	A
264	580	THR	A
265	581	LYS	A
266	582	ASP	A
267	583	LEU	A
268	584	VAL	A
269	585	MET	A
270	586	TYR	A
271	587	SER	A
272	588	TYR	A
273	593	GLN	A
274	594	LYS	A
275	595	ILE	A
276	596	VAL	A
277	597	ILE	A
278	598	ALA	A
279	599	ALA	A
280	600	ASN	A
281	603	LYS	A
282	604	GLU	A
283	605	PRO	A
284	606	LYS	A

Continued on next page

Table S15 – *Continued from previous page*

Homology index	Residue Index	Residue abbreviation	Module
285	607	GLU	A
286	608	ILE	A
287	609	THR	A
288	610	GLY	A
289	611	GLY	A
290	612	ARG	A
291	613	VAL	A
292	614	TRP	A
293	615	ASN	A
294	626	LEU	A
295	627	LYS	A
296	628	PRO	A
297	630	GLU	A
298	631	PHE	A
299	632	ALA	A
300	633	LEU	A
301	634	VAL	A
302	635	VAL	A

Table S16: Residues membership for the *P. stutzeri* α-amylase (PDB code 1GCY, chain A)

Homology index	Residue Index	Residue abbreviation	Module
0	17	GLU	A
1	18	ILE	A
2	19	ILE	B
3	20	LEU	B
4	21	GLN	B
5	22	GLY	C
6	23	PHE	C
7	27	VAL	C
8	34	ASP	C
9	35	TRP	C
10	36	TYR	C
11	37	ASN	C
12	38	ILE	C
13	39	LEU	C
14	40	ARG	C
15	41	GLN	C
16	42	GLN	C
17	43	ALA	C
18	44	ALA	C
19	45	THR	C

Continued on next page

Table S16 – *Continued from previous page*

Homology index	Residue Index	Residue abbreviation	Module
20	46	ILE	C
21	47	ALA	C
22	48	ALA	C
23	49	ASP	C
24	50	GLY	C
25	51	PHE	C
26	52	SER	C
27	53	ALA	C
28	54	ILE	C
29	55	TRP	C
30	56	MET	C
31	57	PRO	C
32	58	VAL	C
33	59	PRO	C
34	60	TRP	C
35	61	ARG	C
36	62	ASP	C
37	63	PHE	C
38	65	SER	C
39	75	GLY	C
40	76	GLU	C
41	77	GLY	C
42	78	TYR	C
43	79	PHE	C
44	80	TRP	C
45	81	HIS	C
46	82	ASP	C
47	83	PHE	C
48	84	ASN	C
49	85	LYS	C
50	86	ASN	C
51	87	GLY	C
52	88	ARG	C
53	89	TYR	C
54	90	GLY	C
55	91	SER	C
56	92	ASP	C
57	93	ALA	C
58	94	GLN	C
59	95	LEU	C
60	96	ARG	C
61	97	GLN	C
62	98	ALA	C
63	99	ALA	C
64	100	SER	C
65	101	ALA	C

Continued on next page

Table S16 – *Continued from previous page*

Homology index	Residue Index	Residue abbreviation	Module
66	102	LEU	C
67	103	GLY	C
68	104	GLY	C
69	105	ALA	C
70	106	GLY	C
71	107	VAL	C
72	108	LYS	C
73	109	VAL	C
74	110	LEU	C
75	111	TYR	C
76	112	ASP	C
77	113	VAL	C
78	114	VAL	C
79	115	PRO	C
80	116	ASN	C
81	117	HIS	C
82	118	MET	C
83	119	ASN	C
84	120	ARG	C
85	121	GLY	C
86	125	LYS	C
87	127	ILE	C
88	128	ASN	C
89	129	LEU	C
90	131	ALA	C
91	132	GLY	C
92	133	GLN	C
93	134	GLY	C
94	135	PHE	B
95	154	ASP	B
96	155	ARG	B
97	156	PHE	B
98	157	ILE	B
99	160	ASP	B
100	161	ALA	B
101	162	ASP	B
102	163	LEU	B
103	164	ASN	B
104	165	THR	B
105	166	GLY	B
106	167	HIS	B
107	168	PRO	B
108	169	GLN	B
109	170	VAL	B
110	171	TYR	B
111	172	GLY	B

Continued on next page

Table S16 – *Continued from previous page*

Homology index	Residue Index	Residue abbreviation	Module
112	173	MET	B
113	174	PHE	B
114	175	ARG	B
115	176	ASP	B
116	177	GLU	B
117	178	PHE	B
118	179	THR	B
119	180	ASN	B
120	181	LEU	B
121	182	ARG	B
122	183	SER	B
123	185	TYR	C
124	186	GLY	C
125	187	ALA	C
126	188	GLY	C
127	189	GLY	C
128	190	PHE	B
129	191	ARG	D
130	192	PHE	B
131	193	ASP	B
132	194	PHE	B
133	195	VAL	B
134	196	ARG	B
135	197	GLY	B
136	198	TYR	B
137	199	ALA	B
138	200	PRO	B
139	201	GLU	B
140	202	ARG	B
141	203	VAL	B
142	204	ASN	B
143	205	SER	B
144	206	TRP	B
145	207	MET	B
146	208	THR	B
147	209	ASP	B
148	210	SER	B
149	211	ALA	B
150	214	SER	B
151	215	PHE	B
152	216	CYS	B
153	217	VAL	C
154	218	GLY	B
155	219	GLU	B
156	220	LEU	B
157	221	TRP	B

Continued on next page

Table S16 – *Continued from previous page*

Homology index	Residue Index	Residue abbreviation	Module
158	222	LYS	B
159	223	GLY	B
160	242	ILE	B
161	243	LYS	B
162	244	ASP	B
163	245	TRP	B
164	247	ASP	B
165	248	ARG	B
166	250	LYS	B
167	251	CYS	B
168	252	PRO	B
169	253	VAL	B
170	254	PHE	D
171	255	ASP	D
172	256	PHE	D
173	257	ALA	D
174	259	LYS	D
175	260	GLU	D
176	261	ARG	D
177	262	MET	D
178	263	GLN	D
179	264	ASN	D
180	265	GLY	D
181	266	SER	D
182	267	ILE	D
183	268	ALA	A
184	269	ASP	A
185	270	TRP	A
186	271	LYS	A
187	272	HIS	D
188	273	GLY	D
189	274	LEU	D
190	276	GLY	D
191	277	ASN	B
192	278	PRO	B
193	282	TRP	B
194	283	ARG	B
195	284	GLU	B
196	285	VAL	B
197	286	ALA	B
198	287	VAL	D
199	288	THR	D
200	289	PHE	D
201	290	VAL	A
202	291	ASP	D
203	292	ASN	D

Continued on next page

Table S16 – *Continued from previous page*

Homology index	Residue Index	Residue abbreviation	Module
204	293	HIS	D
205	294	ASP	D
206	295	THR	D
207	296	GLY	D
208	309	ALA	D
209	310	LEU	D
210	311	GLN	D
211	312	ASP	D
212	313	GLY	A
213	314	LEU	A
214	315	ILE	A
215	316	ARG	A
216	317	GLN	A
217	318	ALA	A
218	319	TYR	A
219	320	ALA	A
220	321	TYR	A
221	322	ILE	A
222	323	LEU	A
223	324	THR	A
224	325	SER	A
225	326	PRO	D
226	327	GLY	D
227	328	THR	D
228	329	PRO	A
229	330	VAL	D
230	331	VAL	A
231	332	TYR	D
232	333	TRP	C
233	334	ASP	D
234	335	HIS	A
235	336	MET	C
236	337	TYR	C
237	338	ASP	D
238	339	TRP	D
239	340	GLY	D
240	341	TYR	A
241	342	GLY	A
242	343	ASP	A
243	344	PHE	A
244	345	ILE	A
245	346	ARG	A
246	347	GLN	A
247	348	LEU	A
248	349	ILE	A
249	350	GLN	A

Continued on next page

Table S16 – *Continued from previous page*

Homology index	Residue Index	Residue abbreviation	Module
250	351	VAL	A
251	352	ARG	A
252	353	ARG	A
253	354	ALA	A
254	355	ALA	A
255	356	GLY	A
256	360	ASP	A
257	361	SER	A
258	362	ALA	A
259	363	ILE	A
260	364	SER	A
261	365	PHE	A
262	366	HIS	A
263	367	SER	A
264	368	GLY	A
265	369	TYR	A
266	370	SER	A
267	371	GLY	A
268	372	LEU	A
269	373	VAL	A
270	374	ALA	A
271	375	THR	A
272	376	VAL	A
273	381	GLN	A
274	382	THR	A
275	383	LEU	A
276	384	VAL	A
277	385	VAL	A
278	386	ALA	A
279	387	LEU	A
280	388	ASN	A
281	389	SER	A
282	390	ASP	A
283	391	LEU	A
284	392	GLY	A
285	393	ASN	A
286	394	PRO	A
287	395	GLY	A
288	401	SER	A
289	402	PHE	A
290	403	SER	A
291	404	GLU	A
292	405	ALA	A
293	406	VAL	A
294	409	SER	A
295	410	ASN	A

Continued on next page

Table S16 – *Continued from previous page*

Homology index	Residue Index	Residue abbreviation	Module
296	411	GLY	A
297	412	GLN	A
298	413	VAL	A
299	414	ARG	A
300	415	VAL	A
301	416	TRP	A
302	417	ARG	A

Table S17: Residues membership for the *H. sapiens* α-amylase (PDB code 1HNY, chain A)

Homology index	Residue Index	Residue abbreviation	Module
0	11	THR	A
1	12	SER	A
2	13	ILE	B
3	14	VAL	B
4	15	HIS	B
5	16	LEU	C
6	17	PHE	C
7	18	GLU	C
8	19	TRP	C
9	20	ARG	C
10	21	TRP	C
11	22	VAL	C
12	23	ASP	C
13	24	ILE	C
14	25	ALA	C
15	26	LEU	C
16	27	GLU	C
17	28	CYS	C
18	29	GLU	C
19	30	ARG	C
20	32	LEU	C
21	33	ALA	C
22	34	PRO	C
23	35	LYS	C
24	36	GLY	C
25	37	PHE	C
26	38	GLY	C
27	39	GLY	C
28	40	VAL	C
29	41	GLN	C
30	42	VAL	C

Continued on next page

Table S17 – *Continued from previous page*

Homology index	Residue Index	Residue abbreviation	Module
31	43	SER	C
32	44	PRO	C
33	45	PRO	C
34	46	ASN	C
35	47	GLU	C
36	48	ASN	C
37	49	VAL	C
38	51	ILE	C
39	59	TRP	C
40	60	GLU	C
41	61	ARG	C
42	62	TYR	C
43	63	GLN	C
44	64	PRO	C
45	65	VAL	C
46	66	SER	C
47	67	TYR	C
48	68	LYS	C
49	69	LEU	C
50	70	CYS	C
51	71	THR	C
52	72	ARG	C
53	73	SER	C
54	74	GLY	C
55	75	ASN	C
56	76	GLU	C
57	77	ASP	C
58	78	GLU	C
59	79	PHE	C
60	80	ARG	C
61	81	ASN	C
62	82	MET	C
63	83	VAL	C
64	84	THR	C
65	85	ARG	C
66	86	CYS	C
67	87	ASN	C
68	88	ASN	C
69	89	VAL	C
70	90	GLY	C
71	91	VAL	C
72	92	ARG	C
73	93	ILE	C
74	94	TYR	C
75	95	VAL	C
76	96	ASP	C

Continued on next page

Table S17 – *Continued from previous page*

Homology index	Residue Index	Residue abbreviation	Module
77	97	ALA	C
78	98	VAL	C
79	99	ILE	C
80	100	ASN	C
81	101	HIS	C
82	102	MET	C
83	103	CYS	C
84	104	GLY	C
85	105	ASN	C
86	126	PHE	C
87	130	PRO	C
88	131	TYR	C
89	132	SER	C
90	133	GLY	C
91	134	TRP	C
92	135	ASP	C
93	136	PHE	C
94	137	ASN	B
95	160	CYS	B
96	161	ARG	B
97	162	LEU	B
98	163	THR	B
99	165	LEU	B
100	166	LEU	B
101	167	ASP	B
102	168	LEU	B
103	169	ALA	B
104	170	LEU	B
105	171	GLU	B
106	172	LYS	B
107	173	ASP	B
108	174	TYR	B
109	175	VAL	B
110	176	ARG	B
111	177	SER	B
112	178	LYS	B
113	179	ILE	B
114	180	ALA	B
115	181	GLU	B
116	182	TYR	B
117	183	MET	B
118	184	ASN	B
119	185	HIS	B
120	186	LEU	B
121	187	ILE	B
122	188	ASP	B

Continued on next page

Table S17 – *Continued from previous page*

Homology index	Residue Index	Residue abbreviation	Module
123	189	ILE	C
124	190	GLY	C
125	191	VAL	C
126	192	ALA	C
127	193	GLY	C
128	194	PHE	B
129	195	ARG	D
130	196	LEU	B
131	197	ASP	B
132	198	ALA	B
133	199	SER	B
134	200	LYS	B
135	201	HIS	B
136	202	MET	B
137	203	TRP	B
138	204	PRO	B
139	205	GLY	B
140	206	ASP	B
141	207	ILE	B
142	208	LYS	B
143	209	ALA	B
144	210	ILE	B
145	211	LEU	B
146	212	ASP	B
147	213	LYS	B
148	214	LEU	B
149	215	HIS	B
150	228	PRO	B
151	229	PHE	B
152	230	ILE	B
153	231	TYR	C
154	232	GLN	B
155	233	GLU	B
156	234	VAL	B
157	235	ILE	B
158	236	ASP	B
159	237	LEU	B
160	244	SER	B
161	245	SER	B
162	246	ASP	B
163	247	TYR	B
164	248	PHE	B
165	249	GLY	B
166	250	ASN	B
167	251	GLY	B
168	252	ARG	B

Continued on next page

Table S17 – *Continued from previous page*

Homology index	Residue Index	Residue abbreviation	Module
169	253	VAL	B
170	254	THR	D
171	255	GLU	D
172	256	PHE	D
173	257	LYS	D
174	259	GLY	D
175	260	ALA	D
176	261	LYS	D
177	262	LEU	D
178	263	GLY	D
179	264	THR	D
180	265	VAL	D
181	266	ILE	D
182	272	GLU	D
183	273	LYS	A
184	274	MET	A
185	275	SER	A
186	280	TRP	A
187	281	GLY	D
188	282	GLU	D
189	283	GLY	D
190	285	GLY	D
191	286	PHE	B
192	287	VAL	B
193	288	PRO	B
194	289	SER	B
195	290	ASP	B
196	291	ARG	B
197	292	ALA	B
198	293	LEU	D
199	294	VAL	D
200	295	PHE	D
201	296	VAL	A
202	297	ASP	D
203	298	ASN	D
204	299	HIS	D
205	300	ASP	D
206	301	ASN	D
207	302	GLN	D
208	314	THR	D
209	315	PHE	D
210	316	TRP	D
211	317	ASP	D
212	319	ARG	A
213	320	LEU	A
214	321	TYR	A

Continued on next page

Table S17 – *Continued from previous page*

Homology index	Residue Index	Residue abbreviation	Module
215	322	LYS	A
216	323	MET	A
217	324	ALA	A
218	325	VAL	A
219	326	GLY	A
220	327	PHE	A
221	328	MET	A
222	329	LEU	A
223	330	ALA	A
224	331	HIS	A
225	333	TYR	D
226	334	GLY	D
227	335	PHE	D
228	336	THR	A
229	337	ARG	D
230	338	VAL	A
231	339	MET	D
232	340	SER	C
233	341	SER	D
234	342	TYR	A
235	343	ARG	C
236	381	ASP	C
237	382	TRP	D
238	383	VAL	D
239	384	CYS	D
240	387	ARG	A
241	388	TRP	A
242	389	ARG	A
243	390	GLN	A
244	391	ILE	A
245	392	ARG	A
246	393	ASN	A
247	394	MET	A
248	395	VAL	A
249	396	ILE	A
250	397	PHE	A
251	398	ARG	A
252	399	ASN	A
253	400	VAL	A
254	401	VAL	A
255	402	ASP	A
256	403	GLY	A
257	404	GLN	A
258	405	PRO	A
259	406	PHE	A
260	408	ASN	A

Continued on next page

Table S17 – *Continued from previous page*

Homology index	Residue Index	Residue abbreviation	Module
261	409	TRP	A
262	410	TYR	A
263	411	ASP	A
264	413	GLY	A
265	414	SER	A
266	415	ASN	A
267	416	GLN	A
268	417	VAL	A
269	418	ALA	A
270	419	PHE	A
271	420	GLY	A
272	421	ARG	A
273	422	GLY	A
274	423	ASN	A
275	425	GLY	A
276	426	PHE	A
277	427	ILE	A
278	428	VAL	A
279	429	PHE	A
280	430	ASN	A
281	433	ASP	A
282	434	TRP	A
283	435	SER	A
284	436	PHE	A
285	437	SER	A
286	438	LEU	A
287	439	THR	A
288	445	PRO	A
289	446	ALA	A
290	449	TYR	A
291	450	CYS	A
292	451	ASP	A
293	452	VAL	A
294	479	ILE	A
295	480	SER	A
296	481	ASN	A
297	486	PRO	A
298	487	PHE	A
299	488	ILE	A
300	489	ALA	A
301	490	ILE	A
302	491	HIS	A

Table S18: Residues membership for the *T. maritima* α-amylase (PDB code 1GJW, chain A)

Homology index	Residue Index	Residue abbreviation	Module
0	84	SER	A
1	85	VAL	A
2	86	VAL	B
3	87	TYR	B
4	88	GLY	B
5	89	SER	C
6	90	LEU	C
7	91	PRO	C
8	117	GLY	C
9	118	THR	C
10	119	PHE	C
11	120	PHE	C
12	121	LYS	C
13	122	MET	C
14	123	MET	C
15	124	LEU	C
16	125	LEU	C
17	126	LEU	C
18	127	PRO	C
19	128	PHE	C
20	129	VAL	C
21	130	LYS	C
22	131	SER	C
23	132	LEU	C
24	133	GLY	C
25	134	ALA	C
26	135	ASP	C
27	136	ALA	C
28	137	ILE	C
29	138	TYR	C
30	139	LEU	C
31	140	LEU	C
32	141	PRO	C
33	142	VAL	C
34	143	SER	C
35	144	ARG	C
36	145	MET	C
37	146	SER	C
38	154	ALA	C
39	155	PRO	C
40	156	SER	C
41	157	PRO	C
42	158	TYR	C
43	159	SER	C

Continued on next page

Table S18 – *Continued from previous page*

Homology index	Residue Index	Residue abbreviation	Module
44	160	VAL	C
45	161	LYS	C
46	162	ASN	C
47	164	MET	C
48	165	GLU	C
49	166	LEU	C
50	167	ASP	C
51	168	GLU	C
52	169	ARG	C
53	170	TYR	C
54	171	HIS	C
55	172	ASP	C
56	181	ASP	C
57	182	GLU	C
58	183	GLU	C
59	184	PHE	C
60	185	LYS	C
61	186	ALA	C
62	187	PHE	C
63	188	VAL	C
64	189	GLU	C
65	190	ALA	C
66	191	CYS	C
67	192	HIS	C
68	193	ILE	C
69	194	LEU	C
70	195	GLY	C
71	196	ILE	C
72	197	ARG	C
73	198	VAL	C
74	199	ILE	C
75	200	LEU	C
76	201	ASP	C
77	202	PHE	C
78	203	ILE	C
79	204	PRO	C
80	205	ARG	C
81	206	THR	C
82	207	ALA	C
83	208	ALA	C
84	209	ARG	C
85	210	ASP	C
86	211	SER	C
87	212	ASP	C
88	213	LEU	C
89	214	ILE	C

Continued on next page

Table S18 – *Continued from previous page*

Homology index	Residue Index	Residue abbreviation	Module
90	218	PRO	C
91	219	ASP	C
92	220	TRP	C
93	221	PHE	C
94	222	TYR	B
95	302	PRO	B
96	303	GLY	B
97	304	PHE	B
98	305	SER	B
99	318	VAL	B
100	319	THR	B
101	320	PHE	B
102	321	LEU	B
103	322	ARG	B
104	323	LEU	B
105	324	TYR	B
106	359	ASN	B
107	360	ARG	B
108	361	GLU	B
109	362	LEU	B
110	363	TRP	B
111	364	GLU	B
112	365	TYR	B
113	366	LEU	B
114	367	ALA	B
115	368	GLY	B
116	369	VAL	B
117	370	ILE	B
118	371	PRO	B
119	372	HIS	B
120	373	TYR	B
121	374	GLN	B
122	375	LYS	B
123	377	TYR	C
124	378	GLY	C
125	379	ILE	C
126	380	ASP	C
127	381	GLY	C
128	382	ALA	B
129	383	ARG	D
130	384	LEU	B
131	385	ASP	B
132	386	MET	B
133	387	GLY	B
134	388	HIS	B
135	389	ALA	B

Continued on next page

Table S18 – *Continued from previous page*

Homology index	Residue Index	Residue abbreviation	Module
136	390	LEU	B
137	391	PRO	B
138	392	LYS	B
139	393	GLU	B
140	394	LEU	B
141	395	LEU	B
142	396	ASP	B
143	397	LEU	B
144	398	ILE	B
145	399	ILE	B
146	400	LYS	B
147	401	ASN	B
148	402	VAL	B
149	403	LYS	B
150	409	PHE	B
151	410	VAL	B
152	411	MET	B
153	412	ILE	C
154	413	ALA	B
155	414	GLU	B
156	415	GLU	B
157	416	LEU	B
158	417	ASP	B
159	418	MET	B
160	421	ASP	B
161	422	LYS	B
162	423	ALA	B
163	424	SER	B
164	425	LYS	B
165	426	GLU	B
166	429	TYR	B
167	430	ASP	B
168	431	VAL	B
169	432	ILE	B
170	433	LEU	D
171	434	GLY	D
172	435	SER	D
173	436	SER	D
174	437	TRP	D
175	438	TYR	D
176	439	PHE	D
177	440	ALA	D
178	441	GLY	D
179	442	ARG	D
180	443	VAL	D
181	444	GLU	D

Continued on next page

Table S18 – *Continued from previous page*

Homology index	Residue Index	Residue abbreviation	Module
182	445	GLU	D
183	446	ILE	A
184	447	GLY	A
185	448	LYS	A
186	449	LEU	A
187	450	PRO	D
188	451	ASP	D
189	452	ILE	D
190	453	ALA	D
191	454	GLU	B
192	455	GLU	B
193	456	LEU	B
194	457	VAL	B
195	458	LEU	B
196	459	PRO	B
197	460	PHE	B
198	461	LEU	D
199	462	ALA	D
200	463	SER	D
201	464	VAL	A
202	465	GLU	D
203	466	THR	D
204	467	PRO	D
205	468	ASP	D
206	469	THR	D
207	470	PRO	D
208	471	ARG	D
209	472	ILE	D
210	473	ALA	D
211	474	THR	D
212	479	SER	A
213	481	MET	A
214	482	LYS	A
215	483	LYS	A
216	484	LEU	A
217	485	ALA	A
218	486	PRO	A
219	487	PHE	A
220	488	VAL	A
221	489	THR	A
222	490	TYR	A
223	491	PHE	A
224	492	LEU	A
225	493	PRO	D
226	495	SER	D
227	496	ILE	D

Continued on next page

Table S18 – *Continued from previous page*

Homology index	Residue Index	Residue abbreviation	Module
228	497	PRO	A
229	498	TYR	D
230	499	VAL	A
231	500	ASN	D
232	501	THR	C
233	502	GLY	D
234	503	GLN	A
235	541	TYR	C
236	542	VAL	C
237	543	LEU	D
238	544	HIS	D
239	545	TRP	D
240	552	VAL	A
241	553	LEU	A
242	554	ASN	A
243	555	PHE	A
244	556	ILE	A
245	557	LYS	A
246	558	LYS	A
247	559	LEU	A
248	560	ILE	A
249	561	LYS	A
250	562	VAL	A
251	563	ARG	A
252	564	HIS	A
253	565	GLU	A
254	566	PHE	A
255	567	LEU	A
256	572	ASN	A
257	573	GLY	A
258	574	LYS	A
259	575	PHE	A
260	576	GLU	A
261	577	ASN	A
262	578	LEU	A
263	579	THR	A
264	580	THR	A
265	581	LYS	A
266	582	ASP	A
267	583	LEU	A
268	584	VAL	A
269	585	MET	A
270	586	TYR	A
271	587	SER	A
272	588	TYR	A
273	593	GLN	A

Continued on next page

Table S18 – *Continued from previous page*

Homology index	Residue Index	Residue abbreviation	Module
274	594	LYS	A
275	595	ILE	A
276	596	VAL	A
277	597	ILE	A
278	598	ALA	A
279	599	ALA	A
280	600	ASN	A
281	603	LYS	A
282	604	GLU	A
283	605	PRO	A
284	606	LYS	A
285	607	GLU	A
286	608	ILE	A
287	609	THR	A
288	610	GLY	A
289	611	GLY	A
290	612	ARG	A
291	613	VAL	A
292	614	TRP	A
293	615	ASN	A
294	626	LEU	A
295	627	LYS	A
296	628	PRO	A
297	630	GLU	A
298	631	PHE	A
299	632	ALA	A
300	633	LEU	A
301	634	VAL	A
302	635	VAL	A

Table S19: Residues membership for the *S. scrofa* α-amylase (PDB code 1HX0, chain A)

Homology index	Residue Index	Residue abbreviation	Module
0	11	THR	A
1	12	SER	A
2	13	ILE	B
3	14	VAL	B
4	15	HIS	B
5	16	LEU	C
6	17	PHE	C
7	18	GLU	C
8	19	TRP	C

Continued on next page

Table S19 – *Continued from previous page*

Homology index	Residue Index	Residue abbreviation	Module
9	20	ARG	C
10	21	TRP	C
11	22	VAL	C
12	23	ASP	C
13	24	ILE	C
14	25	ALA	C
15	26	LEU	C
16	27	GLU	C
17	28	CYS	C
18	29	GLU	C
19	30	ARG	C
20	32	LEU	C
21	33	GLY	C
22	34	PRO	C
23	35	LYS	C
24	36	GLY	C
25	37	PHE	C
26	38	GLY	C
27	39	GLY	C
28	40	VAL	C
29	41	GLN	C
30	42	VAL	C
31	43	SER	C
32	44	PRO	C
33	45	PRO	C
34	46	ASN	C
35	47	GLU	C
36	48	ASN	C
37	49	ILE	C
38	51	VAL	C
39	59	TRP	C
40	60	GLU	C
41	61	ARG	C
42	62	TYR	C
43	63	GLN	C
44	64	PRO	C
45	65	VAL	C
46	66	SER	C
47	67	TYR	C
48	68	LYS	C
49	69	LEU	C
50	70	CYS	C
51	71	THR	C
52	72	ARG	C
53	73	SER	C
54	74	GLY	C

Continued on next page

Table S19 – *Continued from previous page*

Homology index	Residue Index	Residue abbreviation	Module
55	75	ASN	C
56	76	GLU	C
57	77	ASN	C
58	78	GLU	C
59	79	PHE	C
60	80	ARG	C
61	81	ASP	C
62	82	MET	C
63	83	VAL	C
64	84	THR	C
65	85	ARG	C
66	86	CYS	C
67	87	ASN	C
68	88	ASN	C
69	89	VAL	C
70	90	GLY	C
71	91	VAL	C
72	92	ARG	C
73	93	ILE	C
74	94	TYR	C
75	95	VAL	C
76	96	ASP	C
77	97	ALA	C
78	98	VAL	C
79	99	ILE	C
80	100	ASN	C
81	101	HIS	C
82	102	MET	C
83	103	CYS	C
84	104	GLY	C
85	105	SER	C
86	126	PHE	C
87	130	PRO	C
88	131	TYR	C
89	132	SER	C
90	133	ALA	C
91	134	TRP	C
92	135	ASP	C
93	136	PHE	C
94	137	ASN	B
95	160	CYS	B
96	161	GLN	B
97	162	LEU	B
98	163	VAL	B
99	165	LEU	B
100	166	LEU	B

Continued on next page

Table S19 – *Continued from previous page*

Homology index	Residue Index	Residue abbreviation	Module
101	167	ASP	B
102	168	LEU	B
103	169	ALA	B
104	170	LEU	B
105	171	GLU	B
106	172	LYS	B
107	173	ASP	B
108	174	TYR	B
109	175	VAL	B
110	176	ARG	B
111	177	SER	B
112	178	MET	B
113	179	ILE	B
114	180	ALA	B
115	181	ASP	B
116	182	TYR	B
117	183	LEU	B
118	184	ASN	B
119	185	LYS	B
120	186	LEU	B
121	187	ILE	B
122	188	ASP	B
123	189	ILE	C
124	190	GLY	C
125	191	VAL	C
126	192	ALA	C
127	193	GLY	C
128	194	PHE	B
129	195	ARG	D
130	196	ILE	B
131	197	ASP	B
132	198	ALA	B
133	199	SER	B
134	200	LYS	B
135	201	HIS	B
136	202	MET	B
137	203	TRP	B
138	204	PRO	B
139	205	GLY	B
140	206	ASP	B
141	207	ILE	B
142	208	LYS	B
143	209	ALA	B
144	210	VAL	B
145	211	LEU	B
146	212	ASP	B

Continued on next page

Table S19 – *Continued from previous page*

Homology index	Residue Index	Residue abbreviation	Module
147	213	LYS	B
148	214	LEU	B
149	215	HIS	B
150	228	PRO	B
151	229	PHE	B
152	230	ILE	B
153	231	PHE	C
154	232	GLN	B
155	233	GLU	B
156	234	VAL	B
157	235	ILE	B
158	236	ASP	B
159	237	LEU	B
160	244	SER	B
161	245	SER	B
162	246	GLU	B
163	247	TYR	B
164	248	PHE	B
165	249	GLY	B
166	250	ASN	B
167	251	GLY	B
168	252	ARG	B
169	253	VAL	B
170	254	THR	D
171	255	GLU	D
172	256	PHE	D
173	257	LYS	D
174	259	GLY	D
175	260	ALA	D
176	261	LYS	D
177	262	LEU	D
178	263	GLY	D
179	264	THR	D
180	265	VAL	D
181	266	VAL	D
182	272	GLU	D
183	273	LYS	A
184	274	MET	A
185	275	SER	A
186	280	TRP	A
187	281	GLY	D
188	282	GLU	D
189	283	GLY	D
190	285	GLY	D
191	286	PHE	B
192	287	MET	B

Continued on next page

Table S19 – *Continued from previous page*

Homology index	Residue Index	Residue abbreviation	Module
193	288	PRO	B
194	289	SER	B
195	290	ASP	B
196	291	ARG	B
197	292	ALA	B
198	293	LEU	D
199	294	VAL	D
200	295	PHE	D
201	296	VAL	A
202	297	ASP	D
203	298	ASN	D
204	299	HIS	D
205	300	ASP	D
206	301	ASN	D
207	302	GLN	D
208	314	THR	D
209	315	PHE	D
210	316	TRP	D
211	317	ASP	D
212	319	ARG	A
213	320	LEU	A
214	321	TYR	A
215	322	LYS	A
216	323	ILE	A
217	324	ALA	A
218	325	VAL	A
219	326	GLY	A
220	327	PHE	A
221	328	MET	A
222	329	LEU	A
223	330	ALA	A
224	331	HIS	A
225	333	TYR	D
226	334	GLY	D
227	335	PHE	D
228	336	THR	A
229	337	ARG	D
230	338	VAL	A
231	339	MET	D
232	340	SER	C
233	341	SER	D
234	342	TYR	A
235	343	ARG	C
236	381	ASP	C
237	382	TRP	D
238	383	VAL	D

Continued on next page

Table S19 – *Continued from previous page*

Homology index	Residue Index	Residue abbreviation	Module
239	384	CYS	D
240	387	ARG	A
241	388	TRP	A
242	389	ARG	A
243	390	GLU	A
244	391	ILE	A
245	392	ARG	A
246	393	ASN	A
247	394	MET	A
248	395	VAL	A
249	396	TRP	A
250	397	PHE	A
251	398	ARG	A
252	399	ASN	A
253	400	VAL	A
254	401	VAL	A
255	402	ASP	A
256	403	GLY	A
257	404	GLN	A
258	405	PRO	A
259	406	PHE	A
260	408	ASN	A
261	409	TRP	A
262	410	TRP	A
263	411	ASP	A
264	413	GLY	A
265	414	SER	A
266	415	ASN	A
267	416	GLN	A
268	417	VAL	A
269	418	ALA	A
270	419	PHE	A
271	420	GLY	A
272	421	ARG	A
273	422	GLY	A
274	423	ASN	A
275	425	GLY	A
276	426	PHE	A
277	427	ILE	A
278	428	VAL	A
279	429	PHE	A
280	430	ASN	A
281	433	ASP	A
282	434	TRP	A
283	435	GLN	A
284	436	LEU	A

Continued on next page

Table S19 – *Continued from previous page*

Homology index	Residue Index	Residue abbreviation	Module
285	437	SER	A
286	438	SER	A
287	439	THR	A
288	445	PRO	A
289	446	GLY	A
290	449	TYR	A
291	450	CYS	A
292	451	ASP	A
293	452	VAL	A
294	479	ILE	A
295	480	SER	A
296	481	ASN	A
297	486	PRO	A
298	487	PHE	A
299	488	ILE	A
300	489	ALA	A
301	490	ILE	A
302	491	HIS	A

Table S20: Residues membership for the *B. stearothermophilus B* α-amylase (PDB code 1HVX, chain A)

Homology index	Residue Index	Residue abbreviation	Module
0	6	GLY	A
1	7	THR	A
2	8	MET	B
3	9	MET	B
4	10	GLN	B
5	11	TYR	C
6	12	PHE	C
7	16	LEU	C
8	21	THR	C
9	22	LEU	C
10	23	TRP	C
11	24	THR	C
12	25	LYS	C
13	26	VAL	C
14	27	ALA	C
15	28	ASN	C
16	29	GLU	C
17	30	ALA	C
18	31	ASN	C
19	32	ASN	C

Continued on next page

Table S20 – *Continued from previous page*

Homology index	Residue Index	Residue abbreviation	Module
20	33	LEU	C
21	34	SER	C
22	35	SER	C
23	36	LEU	C
24	37	GLY	C
25	38	ILE	C
26	39	THR	C
27	40	ALA	C
28	41	LEU	C
29	42	TRP	C
30	43	LEU	C
31	44	PRO	C
32	45	PRO	C
33	46	ALA	C
34	47	TYR	C
35	48	LYS	C
36	49	GLY	C
37	50	THR	C
38	53	SER	C
39	54	ASP	C
40	55	VAL	C
41	56	GLY	C
42	57	TYR	C
43	58	GLY	C
44	59	VAL	C
45	60	TYR	C
46	61	ASP	C
47	63	TYR	C
48	64	ASP	C
49	65	LEU	C
50	66	GLY	C
51	76	THR	C
52	77	LYS	C
53	78	TYR	C
54	79	GLY	C
55	80	THR	C
56	81	LYS	C
57	82	ALA	C
58	83	GLN	C
59	84	TYR	C
60	85	LEU	C
61	86	GLN	C
62	87	ALA	C
63	88	ILE	C
64	89	GLN	C
65	90	ALA	C

Continued on next page

Table S20 – *Continued from previous page*

**Homology index**	**Residue Index**	**Residue abbreviation**	**Module**
66	91	ALA	C
67	92	HIS	C
68	93	ALA	C
69	94	ALA	C
70	95	GLY	C
71	96	MET	C
72	97	GLN	C
73	98	VAL	C
74	99	TYR	C
75	100	ALA	C
76	101	ASP	C
77	102	VAL	C
78	103	VAL	C
79	104	PHE	C
80	105	ASP	C
81	106	HIS	C
82	107	LYS	C
83	108	GLY	C
84	109	GLY	C
85	110	ALA	C
86	142	PHE	C
87	155	LYS	C
88	156	TRP	C
89	157	ARG	C
90	158	TRP	C
91	159	TYR	C
92	160	HIS	C
93	161	PHE	C
94	162	ASP	B
95	197	ASP	B
96	198	TYR	B
97	199	LEU	B
98	200	MET	B
99	201	TYR	B
100	202	ALA	B
101	203	ASP	B
102	204	LEU	B
103	205	ASP	B
104	206	MET	B
105	207	ASP	B
106	208	HIS	B
107	209	PRO	B
108	210	GLU	B
109	211	VAL	B
110	212	VAL	B
111	213	THR	B

Continued on next page

Table S20 – *Continued from previous page*

Homology index	Residue Index	Residue abbreviation	Module
112	214	GLU	B
113	215	LEU	B
114	216	LYS	B
115	217	SER	B
116	218	TRP	B
117	219	GLY	B
118	220	LYS	B
119	221	TRP	B
120	222	TYR	B
121	223	VAL	B
122	224	ASN	B
123	226	THR	C
124	227	ASN	C
125	228	ILE	C
126	229	ASP	C
127	230	GLY	C
128	231	PHE	B
129	232	ARG	D
130	233	LEU	B
131	234	ASP	B
132	235	ALA	B
133	236	VAL	B
134	237	LYS	B
135	238	HIS	B
136	239	ILE	B
137	240	LYS	B
138	241	PHE	B
139	242	SER	B
140	243	PHE	B
141	244	PHE	B
142	245	PRO	B
143	246	ASP	B
144	247	TRP	B
145	248	LEU	B
146	249	SER	B
147	250	TYR	B
148	251	VAL	B
149	252	ARG	B
150	259	LEU	B
151	260	PHE	B
152	261	THR	B
153	262	VAL	C
154	263	GLY	B
155	264	GLU	B
156	265	TYR	B
157	266	TRP	B

Continued on next page

Table S20 – *Continued from previous page*

Homology index	Residue Index	Residue abbreviation	Module
158	267	SER	B
159	268	TYR	B
160	273	LEU	B
161	274	HIS	B
162	275	ASN	B
163	276	TYR	B
164	277	ILE	B
165	278	MET	B
166	283	THR	B
167	284	MET	B
168	285	SER	B
169	286	LEU	B
170	287	PHE	D
171	288	ASP	D
172	289	ALA	D
173	290	PRO	D
174	292	HIS	D
175	293	ASN	D
176	294	LYS	D
177	295	PHE	D
178	296	TYR	D
179	297	THR	D
180	298	ALA	D
181	299	SER	D
182	305	PHE	D
183	306	ASP	A
184	307	MET	A
185	308	ARG	A
186	310	LEU	A
187	311	MET	D
188	312	THR	D
189	313	ASN	D
190	316	MET	D
191	317	LYS	B
192	318	ASP	B
193	319	GLN	B
194	320	PRO	B
195	321	THR	B
196	322	LEU	B
197	323	ALA	B
198	324	VAL	D
199	325	THR	D
200	326	PHE	D
201	327	VAL	A
202	328	ASP	D
203	329	ASN	D

Continued on next page

Table S20 – *Continued from previous page*

Homology index	Residue Index	Residue abbreviation	Module
204	330	HIS	D
205	331	ASP	D
206	332	THR	D
207	333	GLU	D
208	341	TRP	D
209	342	VAL	D
210	343	ASP	D
211	344	PRO	D
212	345	TRP	A
213	346	PHE	A
214	347	LYS	A
215	348	PRO	A
216	349	LEU	A
217	350	ALA	A
218	351	TYR	A
219	352	ALA	A
220	353	PHE	A
221	354	ILE	A
222	355	LEU	A
223	356	THR	A
224	357	ARG	A
225	359	GLU	D
226	360	GLY	D
227	361	TYR	D
228	362	PRO	A
229	363	CYS	D
230	364	VAL	A
231	365	PHE	D
232	366	TYR	C
233	367	GLY	D
234	368	ASP	A
235	369	TYR	C
236	370	TYR	C
237	371	GLY	D
238	372	ILE	D
239	379	SER	D
240	380	LEU	A
241	381	LYS	A
242	382	SER	A
243	383	LYS	A
244	384	ILE	A
245	385	ASP	A
246	386	PRO	A
247	387	LEU	A
248	388	LEU	A
249	389	ILE	A

Continued on next page

Table S20 – *Continued from previous page*

Homology index	Residue Index	Residue abbreviation	Module
250	390	ALA	A
251	391	ARG	A
252	392	ARG	A
253	393	ASP	A
254	394	TYR	A
255	395	ALA	A
256	397	GLY	A
257	398	THR	A
258	399	GLN	A
259	400	HIS	A
260	401	ASP	A
261	402	TYR	A
262	403	LEU	A
263	404	ASP	A
264	405	HIS	A
265	406	SER	A
266	407	ASP	A
267	408	ILE	A
268	409	ILE	A
269	410	GLY	A
270	411	TRP	A
271	412	THR	A
272	413	ARG	A
273	414	GLU	A
274	415	GLY	A
275	424	LEU	A
276	425	ALA	A
277	426	ALA	A
278	427	LEU	A
279	428	ILE	A
280	429	THR	A
281	431	GLY	A
282	432	PRO	A
283	433	GLY	A
284	434	GLY	A
285	435	SER	A
286	436	LYS	A
287	437	TRP	A
288	446	GLY	A
289	447	LYS	A
290	449	PHE	A
291	450	TYR	A
292	451	ASP	A
293	452	LEU	A
294	472	VAL	A
295	473	ASN	A

Continued on next page

Table S20 – *Continued from previous page*

Homology index	Residue Index	Residue abbreviation	Module
296	474	GLY	A
297	476	SER	A
298	477	VAL	A
299	478	SER	A
300	479	VAL	A
301	480	TRP	A
302	481	VAL	A

Table S21: Residues membership for the *S. scrofa* α-amylase (PDB code 1JFH, chain A)

Homology index	Residue Index	Residue abbreviation	Module
0	11	THR	A
1	12	SER	A
2	13	ILE	B
3	14	VAL	B
4	15	HIS	B
5	16	LEU	C
6	17	PHE	C
7	18	GLU	C
8	19	TRP	C
9	20	ARG	C
10	21	TRP	C
11	22	VAL	C
12	23	ASP	C
13	24	ILE	C
14	25	ALA	C
15	26	LEU	C
16	27	GLU	C
17	28	CYS	C
18	29	GLU	C
19	30	ARG	C
20	32	LEU	C
21	33	GLY	C
22	34	PRO	C
23	35	LYS	C
24	36	GLY	C
25	37	PHE	C
26	38	GLY	C
27	39	GLY	C
28	40	VAL	C
29	41	GLN	C
30	42	VAL	C

Continued on next page

Table S21 – *Continued from previous page*

Homology index	Residue Index	Residue abbreviation	Module
31	43	SER	C
32	44	PRO	C
33	45	PRO	C
34	46	ASN	C
35	47	GLU	C
36	48	ASN	C
37	49	VAL	C
38	51	VAL	C
39	59	TRP	C
40	60	GLU	C
41	61	ARG	C
42	62	TYR	C
43	63	GLN	C
44	64	PRO	C
45	65	VAL	C
46	66	SER	C
47	67	TYR	C
48	68	LYS	C
49	69	LEU	C
50	70	CYS	C
51	71	THR	C
52	72	ARG	C
53	73	SER	C
54	74	GLY	C
55	75	ASN	C
56	76	GLU	C
57	77	ASN	C
58	78	GLU	C
59	79	PHE	C
60	80	ARG	C
61	81	ASP	C
62	82	MET	C
63	83	VAL	C
64	84	THR	C
65	85	ARG	C
66	86	CYS	C
67	87	ASN	C
68	88	ASN	C
69	89	VAL	C
70	90	GLY	C
71	91	VAL	C
72	92	ARG	C
73	93	ILE	C
74	94	TYR	C
75	95	VAL	C
76	96	ASP	C

Continued on next page

Table S21 – *Continued from previous page*

**Homology index**	**Residue Index**	**Residue abbreviation**	**Module**
77	97	ALA	C
78	98	VAL	C
79	99	ILE	C
80	100	ASN	C
81	101	HIS	C
82	102	MET	C
83	103	CYS	C
84	104	GLY	C
85	105	SER	C
86	126	PHE	C
87	130	PRO	C
88	131	TYR	C
89	132	SER	C
90	133	ALA	C
91	134	TRP	C
92	135	ASP	C
93	136	PHE	C
94	137	ASN	B
95	160	CYS	B
96	161	GLN	B
97	162	LEU	B
98	163	VAL	B
99	165	LEU	B
100	166	LEU	B
101	167	ASP	B
102	168	LEU	B
103	169	ALA	B
104	170	LEU	B
105	171	GLU	B
106	172	LYS	B
107	173	ASP	B
108	174	TYR	B
109	175	VAL	B
110	176	ARG	B
111	177	SER	B
112	178	MET	B
113	179	ILE	B
114	180	ALA	B
115	181	ASP	B
116	182	TYR	B
117	183	LEU	B
118	184	ASN	B
119	185	LYS	B
120	186	LEU	B
121	187	ILE	B
122	188	ASP	B

Continued on next page

Table S21 – *Continued from previous page*

Homology index	Residue Index	Residue abbreviation	Module
123	189	ILE	C
124	190	GLY	C
125	191	VAL	C
126	192	ALA	C
127	193	GLY	C
128	194	PHE	B
129	195	ARG	D
130	196	ILE	B
131	197	ASP	B
132	198	ALA	B
133	199	SER	B
134	200	LYS	B
135	201	HIS	B
136	202	MET	B
137	203	TRP	B
138	204	PRO	B
139	205	GLY	B
140	206	ASP	B
141	207	ILE	B
142	208	LYS	B
143	209	ALA	B
144	210	VAL	B
145	211	LEU	B
146	212	ASP	B
147	213	LYS	B
148	214	LEU	B
149	215	HIS	B
150	228	PRO	B
151	229	PHE	B
152	230	ILE	B
153	231	PHE	C
154	232	GLN	B
155	233	GLU	B
156	234	VAL	B
157	235	ILE	B
158	236	ASP	B
159	237	LEU	B
160	244	SER	B
161	245	SER	B
162	246	GLU	B
163	247	TYR	B
164	248	PHE	B
165	249	GLY	B
166	250	ASN	B
167	251	GLY	B
168	252	ARG	B

Continued on next page

Table S21 – *Continued from previous page*

Homology index	Residue Index	Residue abbreviation	Module
169	253	VAL	B
170	254	THR	D
171	255	GLU	D
172	256	PHE	D
173	257	LYS	D
174	259	GLY	D
175	260	ALA	D
176	261	LYS	D
177	262	LEU	D
178	263	GLY	D
179	264	THR	D
180	265	VAL	D
181	266	VAL	D
182	272	GLU	D
183	273	LYS	A
184	274	MET	A
185	275	SER	A
186	280	TRP	A
187	281	GLY	D
188	282	GLU	D
189	283	GLY	D
190	285	GLY	D
191	286	PHE	B
192	287	MET	B
193	288	PRO	B
194	289	SER	B
195	290	ASP	B
196	291	ARG	B
197	292	ALA	B
198	293	LEU	D
199	294	VAL	D
200	295	PHE	D
201	296	VAL	A
202	297	ASP	D
203	298	ASN	D
204	299	HIS	D
205	300	ASP	D
206	301	ASN	D
207	302	GLN	D
208	314	THR	D
209	315	PHE	D
210	316	TRP	D
211	317	ASP	D
212	319	ARG	A
213	320	LEU	A
214	321	TYR	A

Continued on next page

Table S21 – *Continued from previous page*

Homology index	Residue Index	Residue abbreviation	Module
215	322	LYS	A
216	323	ILE	A
217	324	ALA	A
218	325	VAL	A
219	326	GLY	A
220	327	PHE	A
221	328	MET	A
222	329	LEU	A
223	330	ALA	A
224	331	HIS	A
225	333	TYR	D
226	334	GLY	D
227	335	PHE	D
228	336	THR	A
229	337	ARG	D
230	338	VAL	A
231	339	MET	D
232	340	SER	C
233	341	SER	D
234	342	TYR	A
235	343	ARG	C
236	381	ASP	C
237	382	TRP	D
238	383	VAL	D
239	384	CYS	D
240	387	ARG	A
241	388	TRP	A
242	389	ARG	A
243	390	GLU	A
244	391	ILE	A
245	392	ARG	A
246	393	ASN	A
247	394	MET	A
248	395	VAL	A
249	396	TRP	A
250	397	PHE	A
251	398	ARG	A
252	399	ASN	A
253	400	VAL	A
254	401	VAL	A
255	402	ASP	A
256	403	GLY	A
257	404	GLU	A
258	405	PRO	A
259	406	PHE	A
260	408	ASN	A

Continued on next page

Table S21 – *Continued from previous page*

Homology index	Residue Index	Residue abbreviation	Module
261	409	TRP	A
262	410	TRP	A
263	411	ASP	A
264	413	GLY	A
265	414	SER	A
266	415	ASN	A
267	416	GLN	A
268	417	VAL	A
269	418	ALA	A
270	419	PHE	A
271	420	GLY	A
272	421	ARG	A
273	422	GLY	A
274	423	ASN	A
275	425	GLY	A
276	426	PHE	A
277	427	ILE	A
278	428	VAL	A
279	429	PHE	A
280	430	ASN	A
281	433	ASP	A
282	434	TRP	A
283	435	GLN	A
284	436	LEU	A
285	437	SER	A
286	438	SER	A
287	439	THR	A
288	445	PRO	A
289	446	GLY	A
290	449	TYR	A
291	450	CYS	A
292	451	ASP	A
293	452	VAL	A
294	479	ILE	A
295	480	SER	A
296	481	ASN	A
297	486	PRO	A
298	487	PHE	A
299	488	ILE	A
300	489	ALA	A
301	490	ILE	A
302	491	HIS	A

Table S22: Residues membership for the *T. molitor* α-amylase (PDB code 1JAE, chain A)

**Homology index**	**Residue Index**	**Residue abbreviation**	**Module**
0	11	ASN	A
1	12	SER	A
2	13	ILE	B
3	14	VAL	B
4	15	HIS	B
5	16	LEU	C
6	17	PHE	C
7	18	GLU	C
8	19	TRP	C
9	20	LYS	C
10	21	TRP	C
11	22	ASN	C
12	23	ASP	C
13	24	ILE	C
14	25	ALA	C
15	26	ASP	C
16	27	GLU	C
17	28	CYS	C
18	29	GLU	C
19	30	ARG	C
20	32	LEU	C
21	33	GLN	C
22	34	PRO	C
23	35	GLN	C
24	36	GLY	C
25	37	PHE	C
26	38	GLY	C
27	39	GLY	C
28	40	VAL	C
29	41	GLN	C
30	42	ILE	C
31	43	SER	C
32	44	PRO	C
33	45	PRO	C
34	46	ASN	C
35	47	GLU	C
36	48	TYR	C
37	49	LEU	C
38	51	ALA	C
39	57	TRP	C
40	58	GLU	C
41	59	ARG	C
42	60	TYR	C
43	61	GLN	C

Continued on next page

Table S22 – *Continued from previous page*

Homology index	Residue Index	Residue abbreviation	Module
44	62	PRO	C
45	63	VAL	C
46	64	SER	C
47	65	TYR	C
48	66	ILE	C
49	67	ILE	C
50	68	ASN	C
51	69	THR	C
52	70	ARG	C
53	71	SER	C
54	72	GLY	C
55	73	ASP	C
56	74	GLU	C
57	75	SER	C
58	76	ALA	C
59	77	PHE	C
60	78	THR	C
61	79	ASP	C
62	80	MET	C
63	81	THR	C
64	82	ARG	C
65	83	ARG	C
66	84	CYS	C
67	85	ASN	C
68	86	ASP	C
69	87	ALA	C
70	88	GLY	C
71	89	VAL	C
72	90	ARG	C
73	91	ILE	C
74	92	TYR	C
75	93	VAL	C
76	94	ASP	C
77	95	ALA	C
78	96	VAL	C
79	97	ILE	C
80	98	ASN	C
81	99	HIS	C
82	100	MET	C
83	101	THR	C
84	102	GLY	C
85	103	MET	C
86	120	TYR	C
87	124	PRO	C
88	125	TYR	C
89	126	GLY	C

Continued on next page

Table S22 – *Continued from previous page*

Homology index	Residue Index	Residue abbreviation	Module
90	127	SER	C
91	128	GLY	C
92	129	ASP	C
93	130	PHE	C
94	131	HIS	B
95	148	CYS	B
96	149	GLU	B
97	150	LEU	B
98	151	VAL	B
99	153	LEU	B
100	154	ARG	B
101	155	ASP	B
102	156	LEU	B
103	157	ASN	B
104	158	GLN	B
105	159	GLY	B
106	160	SER	B
107	161	ASP	B
108	162	TYR	B
109	163	VAL	B
110	164	ARG	B
111	165	GLY	B
112	166	VAL	B
113	167	LEU	B
114	168	ILE	B
115	169	ASP	B
116	170	TYR	B
117	171	MET	B
118	172	ASN	B
119	173	HIS	B
120	174	MET	B
121	175	ILE	B
122	176	ASP	B
123	177	LEU	C
124	178	GLY	C
125	179	VAL	C
126	180	ALA	C
127	181	GLY	C
128	182	PHE	B
129	183	ARG	D
130	184	VAL	B
131	185	ASP	B
132	186	ALA	B
133	187	ALA	B
134	188	LYS	B
135	189	HIS	B

Continued on next page

Table S22 – *Continued from previous page*

Homology index	Residue Index	Residue abbreviation	Module
136	190	MET	B
137	191	SER	B
138	192	PRO	B
139	193	GLY	B
140	194	ASP	B
141	195	LEU	B
142	196	SER	B
143	197	VAL	B
144	198	ILE	B
145	199	PHE	B
146	200	SER	B
147	201	GLY	B
148	202	LEU	B
149	203	LYS	B
150	217	PRO	B
151	218	PHE	B
152	219	ILE	B
153	220	TYR	C
154	221	GLN	B
155	222	GLU	B
156	223	VAL	B
157	224	ILE	B
158	225	ASP	B
159	226	LEU	B
160	233	LYS	B
161	234	ASN	B
162	235	GLU	B
163	236	TYR	B
164	237	THR	B
165	238	GLY	B
166	239	PHE	B
167	240	GLY	B
168	241	CYS	B
169	242	VAL	B
170	243	LEU	D
171	244	GLU	D
172	245	PHE	D
173	246	GLN	D
174	248	GLY	D
175	249	VAL	D
176	250	SER	D
177	251	LEU	D
178	252	GLY	D
179	253	ASN	D
180	254	ALA	D
181	255	PHE	D

Continued on next page

Table S22 – *Continued from previous page*

Homology index	Residue Index	Residue abbreviation	Module
182	259	ASN	D
183	260	GLN	A
184	261	LEU	A
185	262	LYS	A
186	267	TRP	A
187	268	GLY	D
188	269	PRO	D
189	270	GLU	D
190	272	GLY	D
191	273	LEU	B
192	274	LEU	B
193	275	GLU	B
194	276	GLY	B
195	277	LEU	B
196	278	ASP	B
197	279	ALA	B
198	280	VAL	D
199	281	VAL	D
200	282	PHE	D
201	283	VAL	A
202	284	ASP	D
203	285	ASN	D
204	286	HIS	D
205	287	ASP	D
206	288	ASN	D
207	289	GLN	D
208	298	THR	D
209	299	TYR	D
210	300	LYS	D
211	301	ASN	D
212	303	LYS	A
213	304	PRO	A
214	305	TYR	A
215	306	LYS	A
216	307	MET	A
217	308	ALA	A
218	309	ILE	A
219	310	ALA	A
220	311	PHE	A
221	312	MET	A
222	313	LEU	A
223	314	ALA	A
224	315	HIS	A
225	317	TYR	D
226	318	GLY	D
227	319	THR	D

Continued on next page

Table S22 – *Continued from previous page*

Homology index	Residue Index	Residue abbreviation	Module
228	320	THR	A
229	321	ARG	D
230	322	ILE	A
231	323	MET	D
232	324	SER	C
233	325	SER	D
234	326	PHE	A
235	327	ASP	C
236	357	GLY	C
237	358	TYR	D
238	359	VAL	D
239	360	CYS	D
240	363	ARG	A
241	364	TRP	A
242	365	ARG	A
243	366	GLN	A
244	367	VAL	A
245	368	TYR	A
246	369	GLY	A
247	370	MET	A
248	371	VAL	A
249	372	GLY	A
250	373	PHE	A
251	374	ARG	A
252	375	ASN	A
253	376	ALA	A
254	377	VAL	A
255	378	GLU	A
256	379	GLY	A
257	380	THR	A
258	381	GLN	A
259	382	VAL	A
260	384	ASN	A
261	385	TRP	A
262	386	TRP	A
263	387	SER	A
264	389	ASP	A
265	390	ASP	A
266	391	ASN	A
267	392	GLN	A
268	393	ILE	A
269	394	ALA	A
270	395	PHE	A
271	396	SER	A
272	397	ARG	A
273	398	GLY	A

Continued on next page

Table S22 – *Continued from previous page*

Homology index	Residue Index	Residue abbreviation	Module
274	399	SER	A
275	401	GLY	A
276	402	PHE	A
277	403	VAL	A
278	404	ALA	A
279	405	PHE	A
280	406	THR	A
281	408	GLY	A
282	409	GLY	A
283	410	ASP	A
284	411	LEU	A
285	412	ASN	A
286	413	GLN	A
287	414	ASN	A
288	420	PRO	A
289	421	ALA	A
290	424	TYR	A
291	425	CYS	A
292	426	ASP	A
293	427	VAL	A
294	454	LEU	A
295	455	GLY	A
296	456	SER	A
297	461	GLY	A
298	462	VAL	A
299	463	LEU	A
300	464	ALA	A
301	465	ILE	A
302	466	HIS	A

Table S23: Residues membership for the *N. polysaccharea* α-amylase (PDB code 1S46, chain A)

Homology index	Residue Index	Residue abbreviation	Module
0	98	GLN	A
1	99	VAL	A
2	100	GLY	B
3	101	GLY	B
4	102	VAL	B
5	103	CYS	C
6	104	TYR	C
7	105	VAL	C
8	110	GLY	C

Continued on next page

Table S23 – *Continued from previous page*

Homology index	Residue Index	Residue abbreviation	Module
9	111	ASP	C
10	112	LEU	C
11	113	LYS	C
12	114	GLY	C
13	115	LEU	C
14	116	LYS	C
15	117	ASP	C
16	118	LYS	C
17	119	ILE	C
18	120	PRO	C
19	121	TYR	C
20	122	PHE	C
21	123	GLN	C
22	124	GLU	C
23	125	LEU	C
24	126	GLY	C
25	127	LEU	C
26	128	THR	C
27	129	TYR	C
28	130	LEU	C
29	131	TYR	C
30	132	LEU	C
31	133	MET	C
32	134	PRO	C
33	135	LEU	C
34	136	PHE	C
35	137	LYS	C
36	138	CYS	C
37	139	PRO	C
38	141	GLY	C
39	144	ASP	C
40	145	GLY	C
41	146	GLY	C
42	147	TYR	C
43	148	ALA	C
44	149	VAL	C
45	150	SER	C
46	151	SER	C
47	153	ARG	C
48	154	ASP	C
49	155	VAL	C
50	156	ASN	C
51	157	PRO	C
52	158	ALA	C
53	159	LEU	C
54	160	GLY	C

Continued on next page

Table S23 – *Continued from previous page*

**Homology index**	**Residue Index**	**Residue abbreviation**	**Module**
55	161	THR	C
56	162	ILE	C
57	163	GLY	C
58	164	ASP	C
59	165	LEU	C
60	166	ARG	C
61	167	GLU	C
62	168	VAL	C
63	169	ILE	C
64	170	ALA	C
65	171	ALA	C
66	172	LEU	C
67	173	HIS	C
68	174	GLU	C
69	175	ALA	C
70	176	GLY	C
71	177	ILE	C
72	178	SER	C
73	179	ALA	C
74	180	VAL	C
75	181	VAL	C
76	182	ASP	C
77	183	PHE	C
78	184	ILE	C
79	185	PHE	C
80	186	ASN	C
81	187	HIS	C
82	188	THR	C
83	189	SER	C
84	190	ASN	C
85	191	GLU	C
86	192	HIS	C
87	193	GLU	C
88	194	TRP	C
89	195	ALA	C
90	206	ASP	C
91	207	ASN	C
92	208	PHE	C
93	209	TYR	C
94	210	TYR	B
95	247	TRP	B
96	248	THR	B
97	249	THR	B
98	250	PHE	B
99	254	GLN	B
100	255	TRP	B

Continued on next page

Table S23 – *Continued from previous page*

Homology index	Residue Index	Residue abbreviation	Module
101	256	ASP	B
102	257	LEU	B
103	258	ASN	B
104	259	TYR	B
105	260	SER	B
106	261	ASN	B
107	262	PRO	B
108	263	TRP	B
109	264	VAL	B
110	265	PHE	B
111	266	ARG	B
112	267	ALA	B
113	268	MET	B
114	269	ALA	B
115	270	GLY	B
116	271	GLU	B
117	272	MET	B
118	273	LEU	B
119	274	PHE	B
120	275	LEU	B
121	276	ALA	B
122	277	ASN	B
123	278	LEU	C
124	279	GLY	C
125	280	VAL	C
126	281	ASP	C
127	282	ILE	C
128	283	LEU	B
129	284	ARG	D
130	285	MET	B
131	286	ASP	B
132	287	ALA	B
133	288	VAL	B
134	289	ALA	B
135	290	PHE	B
136	291	ILE	B
137	292	TRP	B
138	306	HIS	B
139	307	ALA	B
140	308	LEU	B
141	309	ILE	B
142	310	ARG	B
143	311	ALA	B
144	312	PHE	B
145	313	ASN	B
146	314	ALA	B

Continued on next page

Table S23 – *Continued from previous page*

Homology index	Residue Index	Residue abbreviation	Module
147	315	VAL	B
148	316	MET	B
149	317	ARG	B
150	323	VAL	B
151	324	PHE	B
152	325	PHE	B
153	326	LYS	C
154	327	SER	B
155	328	GLN	B
156	329	ALA	B
157	330	ILE	B
158	331	VAL	B
159	332	HIS	B
160	336	VAL	B
161	337	VAL	B
162	338	GLN	B
163	339	TYR	B
164	340	ILE	B
165	341	GLY	B
166	345	CYS	B
167	346	GLN	B
168	347	ILE	B
169	348	GLY	B
170	349	TYR	D
171	350	ASN	D
172	351	PRO	D
173	352	LEU	D
174	354	MET	D
175	355	ALA	D
176	356	LEU	D
177	357	LEU	D
178	358	TRP	D
179	359	ASN	D
180	360	THR	D
181	361	LEU	D
182	364	ARG	D
183	365	GLU	A
184	366	VAL	A
185	367	ASN	A
186	372	ALA	A
187	373	LEU	D
188	374	THR	D
189	375	TYR	D
190	376	ARG	D
191	377	HIS	B
192	378	ASN	B

Continued on next page

Table S23 – *Continued from previous page*

Homology index	Residue Index	Residue abbreviation	Module
193	381	GLU	B
194	382	HIS	B
195	383	THR	B
196	384	ALA	B
197	385	TRP	B
198	386	VAL	D
199	387	ASN	D
200	388	TYR	D
201	389	VAL	A
202	390	ARG	D
203	391	SER	D
204	392	HIS	D
205	393	ASP	D
206	394	ASP	D
207	448	SER	D
208	450	THR	D
209	451	ALA	D
210	452	ALA	D
211	453	ALA	D
212	464	ALA	A
213	467	ARG	A
214	468	ILE	A
215	469	LYS	A
216	470	LEU	A
217	471	LEU	A
218	472	TYR	A
219	473	SER	A
220	474	ILE	A
221	475	ALA	A
222	476	LEU	A
223	477	SER	A
224	478	THR	A
225	479	GLY	D
226	480	GLY	D
227	481	LEU	D
228	482	PRO	A
229	483	LEU	D
230	484	ILE	A
231	485	TYR	D
232	486	LEU	C
233	487	GLY	D
234	488	ASP	A
235	514	PRO	C
236	515	ARG	C
237	516	TYR	D
238	517	ASN	D

Continued on next page

Table S23 – *Continued from previous page*

Homology index	Residue Index	Residue abbreviation	Module
239	521	TYR	D
240	534	ILE	A
241	535	TYR	A
242	536	GLN	A
243	537	GLY	A
244	538	LEU	A
245	539	ARG	A
246	540	HIS	A
247	541	MET	A
248	542	ILE	A
249	543	ALA	A
250	544	VAL	A
251	545	ARG	A
252	546	GLN	A
253	547	SER	A
254	548	ASN	A
255	549	PRO	A
256	553	GLY	A
257	554	GLY	A
258	555	ARG	A
259	556	LEU	A
260	558	THR	A
261	559	PHE	A
262	560	ASN	A
263	561	THR	A
264	563	ASN	A
265	564	LYS	A
266	565	HIS	A
267	566	ILE	A
268	567	ILE	A
269	568	GLY	A
270	569	TYR	A
271	570	ILE	A
272	571	ARG	A
273	573	ASN	A
274	574	ALA	A
275	575	LEU	A
276	576	LEU	A
277	577	ALA	A
278	578	PHE	A
279	579	GLY	A
280	580	ASN	A
281	584	TYR	A
282	585	PRO	A
283	586	GLN	A
284	587	THR	A

Continued on next page

Table S23 – *Continued from previous page*

Homology index	Residue Index	Residue abbreviation	Module
285	588	VAL	A
286	589	THR	A
287	590	ALA	A
288	599	LYS	A
289	600	ALA	A
290	601	HIS	A
291	602	ASP	A
292	603	LEU	A
293	604	ILE	A
294	617	LEU	A
295	618	GLN	A
296	619	PRO	A
297	621	GLN	A
298	622	VAL	A
299	623	MET	A
300	624	TRP	A
301	625	LEU	A
302	626	GLU	A

Table S24: Residues membership for the *T. molitor* α-amylase (PDB code 1TMQ, chain A)

Homology index	Residue Index	Residue abbreviation	Module
0	11	ASN	A
1	12	SER	A
2	13	ILE	B
3	14	VAL	B
4	15	HIS	B
5	16	LEU	C
6	17	PHE	C
7	18	GLU	C
8	19	TRP	C
9	20	LYS	C
10	21	TRP	C
11	22	ASN	C
12	23	ASP	C
13	24	ILE	C
14	25	ALA	C
15	26	ASP	C
16	27	GLU	C
17	28	CYS	C
18	29	GLU	C
19	30	ARG	C

Continued on next page

Table S24 – *Continued from previous page*

Homology index	Residue Index	Residue abbreviation	Module
20	32	LEU	C
21	33	GLN	C
22	34	PRO	C
23	35	GLN	C
24	36	GLY	C
25	37	PHE	C
26	38	GLY	C
27	39	GLY	C
28	40	VAL	C
29	41	GLN	C
30	42	ILE	C
31	43	SER	C
32	44	PRO	C
33	45	PRO	C
34	46	ASN	C
35	47	GLU	C
36	48	TYR	C
37	49	LEU	C
38	51	ALA	C
39	57	TRP	C
40	58	GLU	C
41	59	ARG	C
42	60	TYR	C
43	61	GLN	C
44	62	PRO	C
45	63	VAL	C
46	64	SER	C
47	65	TYR	C
48	66	ILE	C
49	67	ILE	C
50	68	ASN	C
51	69	THR	C
52	70	ARG	C
53	71	SER	C
54	72	GLY	C
55	73	ASP	C
56	74	GLU	C
57	75	SER	C
58	76	ALA	C
59	77	PHE	C
60	78	THR	C
61	79	ASP	C
62	80	MET	C
63	81	THR	C
64	82	ARG	C
65	83	ARG	C

Continued on next page

Table S24 – *Continued from previous page*

Homology index	Residue Index	Residue abbreviation	Module
66	84	CYS	C
67	85	ASN	C
68	86	ASP	C
69	87	ALA	C
70	88	GLY	C
71	89	VAL	C
72	90	ARG	C
73	91	ILE	C
74	92	TYR	C
75	93	VAL	C
76	94	ASP	C
77	95	ALA	C
78	96	VAL	C
79	97	ILE	C
80	98	ASN	C
81	99	HIS	C
82	100	MET	C
83	101	THR	C
84	102	GLY	C
85	103	MET	C
86	120	TYR	C
87	124	PRO	C
88	125	TYR	C
89	126	GLY	C
90	127	SER	C
91	128	GLY	C
92	129	ASP	C
93	130	PHE	C
94	131	HIS	B
95	148	CYS	B
96	149	GLU	B
97	150	LEU	B
98	151	VAL	B
99	153	LEU	B
100	154	ARG	B
101	155	ASP	B
102	156	LEU	B
103	157	ASN	B
104	158	GLN	B
105	159	GLY	B
106	160	SER	B
107	161	ASP	B
108	162	TYR	B
109	163	VAL	B
110	164	ARG	B
111	165	GLY	B

Continued on next page

Table S24 – *Continued from previous page*

**Homology index**	**Residue Index**	**Residue abbreviation**	**Module**
112	166	VAL	B
113	167	LEU	B
114	168	ILE	B
115	169	ASP	B
116	170	TYR	B
117	171	MET	B
118	172	ASN	B
119	173	HIS	B
120	174	MET	B
121	175	ILE	B
122	176	ASP	B
123	177	LEU	C
124	178	GLY	C
125	179	VAL	C
126	180	ALA	C
127	181	GLY	C
128	182	PHE	B
129	183	ARG	D
130	184	VAL	B
131	185	ASP	B
132	186	ALA	B
133	187	ALA	B
134	188	LYS	B
135	189	HIS	B
136	190	MET	B
137	191	SER	B
138	192	PRO	B
139	193	GLY	B
140	194	ASP	B
141	195	LEU	B
142	196	SER	B
143	197	VAL	B
144	198	ILE	B
145	199	PHE	B
146	200	SER	B
147	201	GLY	B
148	202	LEU	B
149	203	LYS	B
150	217	PRO	B
151	218	PHE	B
152	219	ILE	B
153	220	TYR	C
154	221	GLN	B
155	222	GLU	B
156	223	VAL	B
157	224	ILE	B

Continued on next page

Table S24 – *Continued from previous page*

Homology index	Residue Index	Residue abbreviation	Module
158	225	ASP	B
159	226	LEU	B
160	233	LYS	B
161	234	ASN	B
162	235	GLU	B
163	236	TYR	B
164	237	THR	B
165	238	GLY	B
166	239	PHE	B
167	240	GLY	B
168	241	CYS	B
169	242	VAL	B
170	243	LEU	D
171	244	GLU	D
172	245	PHE	D
173	246	GLN	D
174	248	GLY	D
175	249	VAL	D
176	250	SER	D
177	251	LEU	D
178	252	GLY	D
179	253	ASN	D
180	254	ALA	D
181	255	PHE	D
182	259	ASN	D
183	260	GLN	A
184	261	LEU	A
185	262	LYS	A
186	267	TRP	A
187	268	GLY	D
188	269	PRO	D
189	270	GLU	D
190	272	GLY	D
191	273	LEU	B
192	274	LEU	B
193	275	GLU	B
194	276	GLY	B
195	277	LEU	B
196	278	ASP	B
197	279	ALA	B
198	280	VAL	D
199	281	VAL	D
200	282	PHE	D
201	283	VAL	A
202	284	ASP	D
203	285	ASN	D

Continued on next page

Table S24 – *Continued from previous page*

Homology index	Residue Index	Residue abbreviation	Module
204	286	HIS	D
205	287	ASP	D
206	288	ASN	D
207	289	GLN	D
208	298	THR	D
209	299	TYR	D
210	300	LYS	D
211	301	ASN	D
212	303	LYS	A
213	304	PRO	A
214	305	TYR	A
215	306	LYS	A
216	307	MET	A
217	308	ALA	A
218	309	ILE	A
219	310	ALA	A
220	311	PHE	A
221	312	MET	A
222	313	LEU	A
223	314	ALA	A
224	315	HIS	A
225	317	TYR	D
226	318	GLY	D
227	319	THR	D
228	320	THR	A
229	321	ARG	D
230	322	ILE	A
231	323	MET	D
232	324	SER	C
233	325	SER	D
234	326	PHE	A
235	327	ASP	C
236	357	GLY	C
237	358	TYR	D
238	359	VAL	D
239	360	CYS	D
240	363	ARG	A
241	364	TRP	A
242	365	ARG	A
243	366	GLN	A
244	367	VAL	A
245	368	TYR	A
246	369	GLY	A
247	370	MET	A
248	371	VAL	A
249	372	GLY	A

Continued on next page

Table S24 – *Continued from previous page*

Homology index	Residue Index	Residue abbreviation	Module
250	373	PHE	A
251	374	ARG	A
252	375	ASN	A
253	376	ALA	A
254	377	VAL	A
255	378	GLU	A
256	379	GLY	A
257	380	THR	A
258	381	GLN	A
259	382	VAL	A
260	384	ASN	A
261	385	TRP	A
262	386	TRP	A
263	387	SER	A
264	389	ASP	A
265	390	ASP	A
266	391	ASN	A
267	392	GLN	A
268	393	ILE	A
269	394	ALA	A
270	395	PHE	A
271	396	SER	A
272	397	ARG	A
273	398	GLY	A
274	399	SER	A
275	401	GLY	A
276	402	PHE	A
277	403	VAL	A
278	404	ALA	A
279	405	PHE	A
280	406	THR	A
281	408	GLY	A
282	409	GLY	A
283	410	ASP	A
284	411	LEU	A
285	412	ASN	A
286	413	GLN	A
287	414	ASN	A
288	420	PRO	A
289	421	ALA	A
290	424	TYR	A
291	425	CYS	A
292	426	ASP	A
293	427	VAL	A
294	454	LEU	A
295	455	GLY	A

Continued on next page

Table S24 – *Continued from previous page*

Homology index	Residue Index	Residue abbreviation	Module
296	456	SER	A
297	461	GLY	A
298	462	VAL	A
299	463	LEU	A
300	464	ALA	A
301	465	ILE	A
302	466	HIS	A

Table S25: Residues membership for the *T. vulgaris R47* α-amylase (PDB code 1VB9, chain A)

Homology index	Residue Index	Residue abbreviation	Module
0	131	ALA	A
1	132	VAL	A
2	133	ILE	B
3	134	TYR	B
4	135	GLN	B
5	136	ILE	C
6	137	PHE	C
7	138	PRO	C
8	170	GLY	C
9	171	ASP	C
10	172	LEU	C
11	173	LYS	C
12	174	GLY	C
13	175	VAL	C
14	176	ILE	C
15	177	ASP	C
16	178	ARG	C
17	179	LEU	C
18	180	PRO	C
19	181	TYR	C
20	182	LEU	C
21	183	GLU	C
22	184	GLU	C
23	185	LEU	C
24	186	GLY	C
25	187	VAL	C
26	188	THR	C
27	189	ALA	C
28	190	LEU	C
29	191	TYR	C
30	192	PHE	C

Continued on next page

Table S25 – *Continued from previous page*

Homology index	Residue Index	Residue abbreviation	Module
31	193	THR	C
32	194	PRO	C
33	195	ILE	C
34	196	PHE	C
35	197	ALA	C
36	198	SER	C
37	199	PRO	C
38	200	SER	C
39	201	HIS	C
40	202	HIS	C
41	203	LYS	C
42	204	TYR	C
43	205	ASP	C
44	206	THR	C
45	207	ALA	C
46	208	ASP	C
47	210	LEU	C
48	211	ALA	C
49	212	ILE	C
50	213	ASP	C
51	214	PRO	C
52	215	GLN	C
53	216	PHE	C
54	217	GLY	C
55	218	ASP	C
56	219	LEU	C
57	220	PRO	C
58	221	THR	C
59	222	PHE	C
60	223	ARG	C
61	224	ARG	C
62	225	LEU	C
63	226	VAL	C
64	227	ASP	C
65	228	GLU	C
66	229	ALA	C
67	230	HIS	C
68	231	ARG	C
69	232	ARG	C
70	233	GLY	C
71	234	ILE	C
72	235	LYS	C
73	236	ILE	C
74	237	ILE	C
75	238	LEU	C
76	239	ASP	C

Continued on next page

Table S25 – *Continued from previous page*

**Homology index**	**Residue Index**	**Residue abbreviation**	**Module**
77	240	ALA	C
78	241	VAL	C
79	242	PHE	C
80	243	ASN	C
81	244	HIS	C
82	245	ALA	C
83	246	GLY	C
84	247	ASP	C
85	248	GLN	C
86	249	PHE	C
87	250	PHE	C
88	251	ALA	C
89	252	PHE	C
90	265	LYS	C
91	266	ASP	C
92	267	TRP	C
93	268	PHE	C
94	269	PHE	B
95	284	GLU	B
96	285	THR	B
97	286	PHE	B
98	287	ALA	B
99	293	MET	B
100	294	PRO	B
101	295	LYS	B
102	296	LEU	B
103	297	ARG	B
104	298	THR	B
105	299	GLU	B
106	300	ASN	B
107	301	PRO	B
108	302	GLU	B
109	303	VAL	B
110	304	LYS	B
111	305	GLU	B
112	306	TYR	B
113	307	LEU	B
114	308	PHE	B
115	309	ASP	B
116	310	VAL	B
117	311	ALA	B
118	312	ARG	B
119	313	PHE	B
120	314	TRP	B
121	315	MET	B
122	316	GLU	B

Continued on next page

Table S25 – *Continued from previous page*

Homology index	Residue Index	Residue abbreviation	Module
123	317	GLN	C
124	318	GLY	C
125	319	ILE	C
126	320	ASP	C
127	321	GLY	C
128	322	TRP	B
129	323	ARG	D
130	324	LEU	B
131	325	ASN	B
132	326	VAL	B
133	327	ALA	B
134	328	ASN	B
135	329	GLU	B
136	330	VAL	B
137	331	ASP	B
138	332	HIS	B
139	333	ALA	B
140	334	PHE	B
141	335	TRP	B
142	336	ARG	B
143	337	GLU	B
144	338	PHE	B
145	339	ARG	B
146	340	ARG	B
147	341	LEU	B
148	342	VAL	B
149	343	LYS	B
150	349	ALA	B
151	350	LEU	B
152	351	ILE	B
153	352	VAL	C
154	353	GLY	B
155	354	GLU	B
156	355	ILE	B
157	356	TRP	B
158	357	HIS	B
159	358	ASP	B
160	359	ALA	B
161	360	SER	B
162	361	GLY	B
163	362	TRP	B
164	363	LEU	B
165	364	MET	B
166	368	PHE	B
167	369	ASP	B
168	370	SER	B

Continued on next page

Table S25 – *Continued from previous page*

Homology index	Residue Index	Residue abbreviation	Module
169	371	VAL	B
170	372	MET	D
171	373	ASN	D
172	374	TYR	D
173	375	LEU	D
174	377	ARG	D
175	378	GLU	D
176	379	SER	D
177	380	VAL	D
178	381	ILE	D
179	382	ARG	D
180	383	PHE	D
181	384	PHE	D
182	389	ILE	D
183	390	HIS	A
184	391	ALA	A
185	392	GLU	A
186	397	GLU	A
187	398	LEU	D
188	399	THR	D
189	400	ARG	D
190	401	ALA	D
191	402	ARG	B
192	403	MET	B
193	409	ALA	B
194	410	ALA	B
195	411	GLN	B
196	412	GLY	B
197	413	LEU	B
198	414	TRP	D
199	415	ASN	D
200	416	LEU	D
201	417	LEU	A
202	418	ASP	D
203	419	SER	D
204	420	HIS	D
205	421	ASP	D
206	422	THR	D
207	423	GLU	D
208	424	ARG	D
209	425	PHE	D
210	426	LEU	D
211	427	THR	D
212	433	GLU	A
213	435	LYS	A
214	436	PHE	A

Continued on next page

Table S25 – *Continued from previous page*

Homology index	Residue Index	Residue abbreviation	Module
215	437	ARG	A
216	438	LEU	A
217	439	ALA	A
218	440	VAL	A
219	441	LEU	A
220	442	PHE	A
221	443	GLN	A
222	444	MET	A
223	445	THR	A
224	446	TYR	A
225	447	LEU	D
226	448	GLY	D
227	449	THR	D
228	450	PRO	A
229	451	LEU	D
230	452	ILE	A
231	453	TYR	D
232	454	TYR	C
233	455	GLY	D
234	456	ASP	A
235	470	ARG	C
236	471	PRO	C
237	472	MET	D
238	473	ILE	D
239	474	TRP	D
240	483	LEU	A
241	484	PHE	A
242	485	GLU	A
243	486	PHE	A
244	487	TYR	A
245	488	LYS	A
246	489	GLU	A
247	490	LEU	A
248	491	ILE	A
249	492	ARG	A
250	493	LEU	A
251	494	ARG	A
252	495	HIS	A
253	496	ARG	A
254	497	LEU	A
255	498	ALA	A
256	502	ARG	A
257	503	GLY	A
258	504	ASN	A
259	505	VAL	A
260	507	SER	A

Continued on next page

Table S25 – *Continued from previous page*

Homology index	Residue Index	Residue abbreviation	Module
261	508	TRP	A
262	509	HIS	A
263	510	ALA	A
264	513	GLN	A
265	514	ALA	A
266	515	ASN	A
267	516	LEU	A
268	517	TYR	A
269	518	ALA	A
270	519	PHE	A
271	520	VAL	A
272	521	ARG	A
273	526	GLN	A
274	527	HIS	A
275	528	VAL	A
276	529	GLY	A
277	530	VAL	A
278	531	VAL	A
279	532	LEU	A
280	533	ASN	A
281	536	GLY	A
282	537	GLU	A
283	538	LYS	A
284	539	GLN	A
285	540	THR	A
286	541	VAL	A
287	542	LEU	A
288	552	THR	A
289	553	TRP	A
290	554	LEU	A
291	555	ASP	A
292	556	CYS	A
293	557	LEU	A
294	573	LEU	A
295	574	ARG	A
296	575	PRO	A
297	577	GLN	A
298	578	GLY	A
299	579	MET	A
300	580	ILE	A
301	581	LEU	A
302	582	TRP	A

Table S26: Residues membership for the *H. sapiens* α-amylase (PDB code 1U33, chain A)

Homology index	Residue Index	Residue abbreviation	Module
0	11	THR	A
1	12	SER	A
2	13	ILE	B
3	14	VAL	B
4	15	HIS	B
5	16	LEU	C
6	17	PHE	C
7	18	GLU	C
8	19	TRP	C
9	20	ARG	C
10	21	TRP	C
11	22	VAL	C
12	23	ASP	C
13	24	ILE	C
14	25	ALA	C
15	26	LEU	C
16	27	GLU	C
17	28	CYS	C
18	29	GLU	C
19	30	ARG	C
20	32	LEU	C
21	33	ALA	C
22	34	PRO	C
23	35	LYS	C
24	36	GLY	C
25	37	PHE	C
26	38	GLY	C
27	39	GLY	C
28	40	VAL	C
29	41	GLN	C
30	42	VAL	C
31	43	SER	C
32	44	PRO	C
33	45	PRO	C
34	46	ASN	C
35	47	GLU	C
36	48	ASN	C
37	49	VAL	C
38	51	ILE	C
39	59	TRP	C
40	60	GLU	C
41	61	ARG	C
42	62	TYR	C
43	63	GLN	C

Continued on next page

Table S26 – *Continued from previous page*

Homology index	Residue Index	Residue abbreviation	Module
44	64	PRO	C
45	65	VAL	C
46	66	SER	C
47	67	TYR	C
48	68	LYS	C
49	69	LEU	C
50	70	CYS	C
51	71	THR	C
52	72	ARG	C
53	73	SER	C
54	74	GLY	C
55	75	ASN	C
56	76	GLU	C
57	77	ASP	C
58	78	GLU	C
59	79	PHE	C
60	80	ARG	C
61	81	ASN	C
62	82	MET	C
63	83	VAL	C
64	84	THR	C
65	85	ARG	C
66	86	CYS	C
67	87	ASN	C
68	88	ASN	C
69	89	VAL	C
70	90	GLY	C
71	91	VAL	C
72	92	ARG	C
73	93	ILE	C
74	94	TYR	C
75	95	VAL	C
76	96	ASP	C
77	97	ALA	C
78	98	VAL	C
79	99	ILE	C
80	100	ASN	C
81	101	HIS	C
82	102	MET	C
83	103	CYS	C
84	104	GLY	C
85	105	ASN	C
86	126	PHE	C
87	130	PRO	C
88	131	TYR	C
89	132	SER	C

Continued on next page

Table S26 – *Continued from previous page*

Homology index	Residue Index	Residue abbreviation	Module
90	133	GLY	C
91	134	TRP	C
92	135	ASP	C
93	136	PHE	C
94	137	ASN	B
95	160	CYS	B
96	161	ARG	B
97	162	LEU	B
98	163	THR	B
99	165	LEU	B
100	166	LEU	B
101	167	ASP	B
102	168	LEU	B
103	169	ALA	B
104	170	LEU	B
105	171	GLU	B
106	172	LYS	B
107	173	ASP	B
108	174	TYR	B
109	175	VAL	B
110	176	ARG	B
111	177	SER	B
112	178	LYS	B
113	179	ILE	B
114	180	ALA	B
115	181	GLU	B
116	182	TYR	B
117	183	MET	B
118	184	ASN	B
119	185	HIS	B
120	186	LEU	B
121	187	ILE	B
122	188	ASP	B
123	189	ILE	C
124	190	GLY	C
125	191	VAL	C
126	192	ALA	C
127	193	GLY	C
128	194	PHE	B
129	195	ARG	D
130	196	LEU	B
131	197	ASP	B
132	198	ALA	B
133	199	SER	B
134	200	LYS	B
135	201	HIS	B

Continued on next page

Table S26 – *Continued from previous page*

Homology index	Residue Index	Residue abbreviation	Module
136	202	MET	B
137	203	TRP	B
138	204	PRO	B
139	205	GLY	B
140	206	ASP	B
141	207	ILE	B
142	208	LYS	B
143	209	ALA	B
144	210	ILE	B
145	211	LEU	B
146	212	ASP	B
147	213	LYS	B
148	214	LEU	B
149	215	HIS	B
150	228	PRO	B
151	229	PHE	B
152	230	ILE	B
153	231	TYR	C
154	232	GLN	B
155	233	GLU	B
156	234	VAL	B
157	235	ILE	B
158	236	ASP	B
159	237	LEU	B
160	244	SER	B
161	245	SER	B
162	246	ASP	B
163	247	TYR	B
164	248	PHE	B
165	249	GLY	B
166	250	ASN	B
167	251	GLY	B
168	252	ARG	B
169	253	VAL	B
170	254	THR	D
171	255	GLU	D
172	256	PHE	D
173	257	LYS	D
174	259	GLY	D
175	260	ALA	D
176	261	LYS	D
177	262	LEU	D
178	263	GLY	D
179	264	THR	D
180	265	VAL	D
181	266	ILE	D

Continued on next page

Table S26 – *Continued from previous page*

Homology index	Residue Index	Residue abbreviation	Module
182	272	GLU	D
183	273	LYS	A
184	274	MET	A
185	275	SER	A
186	280	TRP	A
187	281	GLY	D
188	282	GLU	D
189	283	GLY	D
190	285	GLY	D
191	286	PHE	B
192	287	VAL	B
193	288	PRO	B
194	289	SER	B
195	290	ASP	B
196	291	ARG	B
197	292	ALA	B
198	293	LEU	D
199	294	VAL	D
200	295	PHE	D
201	296	VAL	A
202	297	ASP	D
203	298	ASN	D
204	299	HIS	D
205	300	ASP	D
206	301	ASN	D
207	302	GLN	D
208	314	THR	D
209	315	PHE	D
210	316	TRP	D
211	317	ASP	D
212	319	ARG	A
213	320	LEU	A
214	321	TYR	A
215	322	LYS	A
216	323	MET	A
217	324	ALA	A
218	325	VAL	A
219	326	GLY	A
220	327	PHE	A
221	328	MET	A
222	329	LEU	A
223	330	ALA	A
224	331	HIS	A
225	333	TYR	D
226	334	GLY	D
227	335	PHE	D

Continued on next page

Table S26 – *Continued from previous page*

Homology index	Residue Index	Residue abbreviation	Module
228	336	THR	A
229	337	ARG	D
230	338	VAL	A
231	339	MET	D
232	340	SER	C
233	341	SER	D
234	342	TYR	A
235	343	ARG	C
236	381	ASP	C
237	382	TRP	D
238	383	VAL	D
239	384	CYS	D
240	387	ARG	A
241	388	TRP	A
242	389	ARG	A
243	390	GLN	A
244	391	ILE	A
245	392	ARG	A
246	393	ASN	A
247	394	MET	A
248	395	VAL	A
249	396	ILE	A
250	397	PHE	A
251	398	ARG	A
252	399	ASN	A
253	400	VAL	A
254	401	VAL	A
255	402	ASP	A
256	403	GLY	A
257	404	GLN	A
258	405	PRO	A
259	406	PHE	A
260	408	ASN	A
261	409	TRP	A
262	410	TYR	A
263	411	ASP	A
264	413	GLY	A
265	414	SER	A
266	415	ASN	A
267	416	GLN	A
268	417	VAL	A
269	418	ALA	A
270	419	PHE	A
271	420	GLY	A
272	421	ARG	A
273	422	GLY	A

Continued on next page

Table S26 – *Continued from previous page*

Homology index	Residue Index	Residue abbreviation	Module
274	423	ASN	A
275	425	GLY	A
276	426	PHE	A
277	427	ILE	A
278	428	VAL	A
279	429	PHE	A
280	430	ASN	A
281	433	ASP	A
282	434	TRP	A
283	435	SER	A
284	436	PHE	A
285	437	SER	A
286	438	LEU	A
287	439	THR	A
288	445	PRO	A
289	446	ALA	A
290	449	TYR	A
291	450	CYS	A
292	451	ASP	A
293	452	VAL	A
294	479	ILE	A
295	480	SER	A
296	481	ASN	A
297	486	PRO	A
298	487	PHE	A
299	488	ILE	A
300	489	ALA	A
301	490	ILE	A
302	491	HIS	A

Table S27: Residues membership for the *T. thermosulfurigenes EM1* α-amylase (PDB code 1A47, chain A)

Homology index	Residue Index	Residue abbreviation	Module
0	15	ASP	A
1	16	VAL	A
2	17	ILE	B
3	18	TYR	B
4	19	GLN	B
5	20	ILE	C
6	21	VAL	C
7	22	THR	C
8	52	GLY	C

Continued on next page

Table S27 – *Continued from previous page*

Homology index	Residue Index	Residue abbreviation	Module
9	53	ASP	C
10	54	TRP	C
11	55	GLN	C
12	56	GLY	C
13	57	ILE	C
14	58	ILE	C
15	59	ASN	C
16	60	LYS	C
17	61	ILE	C
18	62	ASN	C
19	63	ASP	C
20	66	LEU	C
21	67	THR	C
22	68	GLY	C
23	69	MET	C
24	70	GLY	C
25	71	VAL	C
26	72	THR	C
27	73	ALA	C
28	74	ILE	C
29	75	TRP	C
30	76	ILE	C
31	77	SER	C
32	78	GLN	C
33	79	PRO	C
34	80	VAL	C
35	81	GLU	C
36	82	ASN	C
37	83	ILE	C
38	96	THR	C
39	98	TYR	C
40	99	HIS	C
41	100	GLY	C
42	101	TYR	C
43	102	TRP	C
44	103	ALA	C
45	104	ARG	C
46	105	ASP	C
47	107	LYS	C
48	108	ARG	C
49	109	THR	C
50	110	ASN	C
51	111	PRO	C
52	112	TYR	C
53	113	PHE	C
54	114	GLY	C

Continued on next page

Table S27 – *Continued from previous page*

Homology index	Residue Index	Residue abbreviation	Module
55	115	SER	C
56	116	PHE	C
57	117	THR	C
58	118	ASP	C
59	119	PHE	C
60	120	GLN	C
61	121	ASN	C
62	122	LEU	C
63	123	ILE	C
64	124	ASN	C
65	125	THR	C
66	126	ALA	C
67	127	HIS	C
68	128	ALA	C
69	129	HIS	C
70	130	ASN	C
71	131	ILE	C
72	132	LYS	C
73	133	VAL	C
74	134	ILE	C
75	135	ILE	C
76	136	ASP	C
77	137	PHE	C
78	138	ALA	C
79	139	PRO	C
80	140	ASN	C
81	141	HIS	C
82	142	THR	C
83	143	SER	C
84	144	PRO	C
85	145	ALA	C
86	157	ARG	C
87	164	LEU	C
88	165	LEU	C
89	166	GLY	C
90	173	ASN	C
91	174	GLY	C
92	175	TYR	C
93	176	PHE	C
94	177	HIS	B
95	193	ARG	B
96	194	ASN	B
97	195	LEU	B
98	196	PHE	B
99	198	LEU	B
100	199	ALA	B

Continued on next page

Table S27 – *Continued from previous page*

Homology index	Residue Index	Residue abbreviation	Module
101	200	ASP	B
102	201	LEU	B
103	202	ASN	B
104	203	GLN	B
105	204	GLN	B
106	205	ASN	B
107	206	SER	B
108	207	THR	B
109	208	ILE	B
110	209	ASP	B
111	210	SER	B
112	211	TYR	B
113	212	LEU	B
114	213	LYS	B
115	214	SER	B
116	215	ALA	B
117	216	ILE	B
118	217	LYS	B
119	218	VAL	B
120	219	TRP	B
121	220	LEU	B
122	221	ASP	B
123	222	MET	C
124	223	GLY	C
125	224	ILE	C
126	225	ASP	C
127	226	GLY	C
128	227	ILE	B
129	228	ARG	D
130	229	LEU	B
131	230	ASP	B
132	231	ALA	B
133	232	VAL	B
134	233	LYS	B
135	234	HIS	B
136	235	MET	B
137	236	PRO	B
138	237	PHE	B
139	238	GLY	B
140	239	TRP	B
141	240	GLN	B
142	241	LYS	B
143	242	ASN	B
144	243	PHE	B
145	244	MET	B
146	245	ASP	B

Continued on next page

Table S27 – *Continued from previous page*

Homology index	Residue Index	Residue abbreviation	Module
147	246	SER	B
148	247	ILE	B
149	248	LEU	B
150	253	VAL	B
151	254	PHE	B
152	255	THR	B
153	256	PHE	C
154	257	GLY	B
155	258	GLU	B
156	259	TRP	B
157	260	PHE	B
158	261	LEU	B
159	262	GLY	B
160	270	ASN	B
161	271	THR	B
162	272	TYR	B
163	273	PHE	B
164	275	ASN	B
165	276	GLU	B
166	278	GLY	B
167	279	MET	B
168	280	SER	B
169	281	LEU	B
170	282	LEU	D
171	283	ASP	D
172	284	PHE	D
173	285	ARG	D
174	287	SER	D
175	288	GLN	D
176	289	LYS	D
177	290	VAL	D
178	291	ARG	D
179	292	GLN	D
180	293	VAL	D
181	294	PHE	D
182	299	ASP	D
183	300	THR	A
184	301	MET	A
185	302	TYR	A
186	307	MET	A
187	308	ILE	D
188	309	GLN	D
189	310	SER	D
190	311	THR	D
191	312	ALA	B
192	313	SER	B

Continued on next page

Table S27 – *Continued from previous page*

Homology index	Residue Index	Residue abbreviation	Module
193	317	PHE	B
194	318	ILE	B
195	319	ASN	B
196	320	ASP	B
197	321	MET	B
198	322	VAL	D
199	323	THR	D
200	324	PHE	D
201	325	ILE	A
202	326	ASP	D
203	327	ASN	D
204	328	HIS	D
205	329	ASP	D
206	330	MET	D
207	331	ASP	D
208	332	ARG	D
209	333	PHE	D
210	334	TYR	D
211	335	ASN	D
212	339	THR	A
213	341	PRO	A
214	342	VAL	A
215	343	GLU	A
216	344	GLN	A
217	345	ALA	A
218	346	LEU	A
219	347	ALA	A
220	348	PHE	A
221	349	THR	A
222	350	LEU	A
223	351	THR	A
224	352	SER	A
225	353	ARG	D
226	354	GLY	D
227	355	VAL	D
228	356	PRO	A
229	357	ALA	D
230	358	ILE	A
231	359	TYR	D
232	360	TYR	C
233	361	GLY	D
234	362	THR	A
235	376	ALA	C
236	377	MET	C
237	378	MET	D
238	379	THR	D

Continued on next page

Table S27 – *Continued from previous page*

Homology index	Residue Index	Residue abbreviation	Module
239	381	PHE	D
240	387	ALA	A
241	388	TYR	A
242	389	ASN	A
243	390	VAL	A
244	391	ILE	A
245	392	LYS	A
246	393	LYS	A
247	394	LEU	A
248	395	ALA	A
249	396	PRO	A
250	397	LEU	A
251	398	ARG	A
252	399	LYS	A
253	400	SER	A
254	401	ASN	A
255	402	PRO	A
256	406	TYR	A
257	407	GLY	A
258	408	THR	A
259	409	THR	A
260	411	GLN	A
261	412	ARG	A
262	413	TRP	A
263	414	ILE	A
264	415	ASN	A
265	416	ASN	A
266	417	ASP	A
267	418	VAL	A
268	419	TYR	A
269	420	ILE	A
270	421	TYR	A
271	422	GLU	A
272	423	ARG	A
273	428	ASN	A
274	429	VAL	A
275	430	ALA	A
276	431	LEU	A
277	432	VAL	A
278	433	ALA	A
279	434	ILE	A
280	435	ASN	A
281	439	SER	A
282	440	THR	A
283	441	SER	A
284	442	TYR	A

Continued on next page

Table S27 – *Continued from previous page*

Homology index	Residue Index	Residue abbreviation	Module
285	443	ASN	A
286	444	ILE	A
287	445	THR	A
288	454	GLY	A
289	455	THR	A
290	457	THR	A
291	458	ASP	A
292	459	VAL	A
293	460	LEU	A
294	483	SER	A
295	484	ALA	A
296	485	GLY	A
297	486	GLU	A
298	487	VAL	A
299	488	ALA	A
300	489	VAL	A
301	490	TRP	A
302	491	GLN	A

Table S28: Residues membership for the *B. circulans* α-amylase (PDB code 1KCL, chain A)

Homology index	Residue Index	Residue abbreviation	Module
0	15	ASP	A
1	16	VAL	A
2	17	ILE	B
3	18	TYR	B
4	19	GLN	B
5	20	ILE	C
6	21	PHE	C
7	22	THR	C
8	52	GLY	C
9	53	ASP	C
10	54	TRP	C
11	55	GLN	C
12	56	GLY	C
13	57	ILE	C
14	58	ILE	C
15	59	ASN	C
16	60	LYS	C
17	61	ILE	C
18	62	ASN	C
19	63	ASP	C

Continued on next page

Table S28 – *Continued from previous page*

Homology index	Residue Index	Residue abbreviation	Module
20	66	LEU	C
21	67	THR	C
22	68	GLY	C
23	69	MET	C
24	70	GLY	C
25	71	VAL	C
26	72	THR	C
27	73	ALA	C
28	74	ILE	C
29	75	TRP	C
30	76	ILE	C
31	77	SER	C
32	78	GLN	C
33	79	PRO	C
34	80	VAL	C
35	81	GLU	C
36	82	ASN	C
37	83	ILE	C
38	95	THR	C
39	97	TYR	C
40	98	HIS	C
41	99	GLY	C
42	100	TYR	C
43	101	TRP	C
44	102	ALA	C
45	103	ARG	C
46	104	ASP	C
47	106	LYS	C
48	107	LYS	C
49	108	THR	C
50	109	ASN	C
51	110	PRO	C
52	111	ALA	C
53	112	TYR	C
54	113	GLY	C
55	114	THR	C
56	115	ILE	C
57	116	ALA	C
58	117	ASP	C
59	118	PHE	C
60	119	GLN	C
61	120	ASN	C
62	121	LEU	C
63	122	ILE	C
64	123	ALA	C
65	124	ALA	C

Continued on next page

Table S28 – *Continued from previous page*

**Homology index**	**Residue Index**	**Residue abbreviation**	**Module**
66	125	ALA	C
67	126	HIS	C
68	127	ALA	C
69	128	LYS	C
70	129	ASN	C
71	130	ILE	C
72	131	LYS	C
73	132	VAL	C
74	133	ILE	C
75	134	ILE	C
76	135	ASP	C
77	136	PHE	C
78	137	ALA	C
79	138	PRO	C
80	139	ASN	C
81	140	HIS	C
82	141	THR	C
83	142	SER	C
84	143	PRO	C
85	144	ALA	C
86	156	ARG	C
87	163	LEU	C
88	164	LEU	C
89	165	GLY	C
90	172	GLN	C
91	173	ASN	C
92	174	LEU	C
93	175	PHE	C
94	176	HIS	B
95	192	LYS	B
96	193	ASN	B
97	194	LEU	B
98	195	TYR	B
99	197	LEU	B
100	198	ALA	B
101	199	ASP	B
102	200	LEU	B
103	201	ASN	B
104	202	HIS	B
105	203	ASN	B
106	204	ASN	B
107	205	SER	B
108	206	THR	B
109	207	VAL	B
110	208	ASP	B
111	209	VAL	B

Continued on next page

Table S28 – *Continued from previous page*

Homology index	Residue Index	Residue abbreviation	Module
112	210	TYR	B
113	211	LEU	B
114	212	LYS	B
115	213	ASP	B
116	214	ALA	B
117	215	ILE	B
118	216	LYS	B
119	217	MET	B
120	218	TRP	B
121	219	LEU	B
122	220	ASP	B
123	221	LEU	C
124	222	GLY	C
125	223	ILE	C
126	224	ASP	C
127	225	GLY	C
128	226	ILE	B
129	227	ARG	D
130	228	MET	B
131	229	ASP	B
132	230	ALA	B
133	231	VAL	B
134	232	LYS	B
135	233	HIS	B
136	234	MET	B
137	235	PRO	B
138	236	PHE	B
139	237	GLY	B
140	238	TRP	B
141	239	GLN	B
142	240	LYS	B
143	241	SER	B
144	242	PHE	B
145	243	MET	B
146	244	ALA	B
147	245	ALA	B
148	246	VAL	B
149	247	ASN	B
150	252	VAL	B
151	253	PHE	B
152	254	THR	B
153	255	PHE	C
154	256	GLY	B
155	257	GLU	B
156	258	TRP	B
157	259	PHE	B

Continued on next page

Table S28 – *Continued from previous page*

Homology index	Residue Index	Residue abbreviation	Module
158	260	LEU	B
159	261	GLY	B
160	269	ASN	B
161	270	HIS	B
162	271	LYS	B
163	272	PHE	B
164	274	ASN	B
165	275	GLU	B
166	277	GLY	B
167	278	MET	B
168	279	SER	B
169	280	LEU	B
170	281	LEU	D
171	282	ASP	D
172	283	PHE	D
173	284	ARG	D
174	286	ALA	D
175	287	GLN	D
176	288	LYS	D
177	289	VAL	D
178	290	ARG	D
179	291	GLN	D
180	292	VAL	D
181	293	PHE	D
182	298	ASP	D
183	299	ASN	A
184	300	MET	A
185	301	TYR	A
186	306	MET	A
187	307	LEU	D
188	308	GLU	D
189	309	GLY	D
190	310	SER	D
191	311	ALA	B
192	312	ALA	B
193	316	GLN	B
194	317	VAL	B
195	318	ASP	B
196	319	ASP	B
197	320	GLN	B
198	321	VAL	D
199	322	THR	D
200	323	PHE	D
201	324	ILE	A
202	325	ASP	D
203	326	ASN	D

Continued on next page

Table S28 – *Continued from previous page*

Homology index	Residue Index	Residue abbreviation	Module
204	327	HIS	D
205	328	ASP	D
206	329	MET	D
207	330	GLU	D
208	331	ARG	D
209	332	PHE	D
210	333	HIS	D
211	334	ALA	D
212	339	ARG	A
213	341	LYS	A
214	342	LEU	A
215	343	GLU	A
216	344	GLN	A
217	345	ALA	A
218	346	LEU	A
219	347	ALA	A
220	348	PHE	A
221	349	THR	A
222	350	LEU	A
223	351	THR	A
224	352	SER	A
225	353	ARG	D
226	354	GLY	D
227	355	VAL	D
228	356	PRO	A
229	357	ALA	D
230	358	ILE	A
231	359	TYR	D
232	360	TYR	C
233	361	GLY	D
234	362	THR	A
235	376	ALA	C
236	377	ARG	C
237	378	ILE	D
238	379	PRO	D
239	381	PHE	D
240	387	ALA	A
241	388	TYR	A
242	389	GLN	A
243	390	VAL	A
244	391	ILE	A
245	392	GLN	A
246	393	LYS	A
247	394	LEU	A
248	395	ALA	A
249	396	PRO	A

Continued on next page

Table S28 – *Continued from previous page*

Homology index	Residue Index	Residue abbreviation	Module
250	397	LEU	A
251	398	ARG	A
252	399	LYS	A
253	400	CYS	A
254	401	ASN	A
255	402	PRO	A
256	406	TYR	A
257	407	GLY	A
258	408	SER	A
259	409	THR	A
260	411	GLU	A
261	412	ARG	A
262	413	TRP	A
263	414	ILE	A
264	415	ASN	A
265	416	ASN	A
266	417	ASP	A
267	418	VAL	A
268	419	LEU	A
269	420	ILE	A
270	421	TYR	A
271	422	GLU	A
272	423	ARG	A
273	428	ASN	A
274	429	VAL	A
275	430	ALA	A
276	431	VAL	A
277	432	VAL	A
278	433	ALA	A
279	434	VAL	A
280	435	ASN	A
281	439	ASN	A
282	440	ALA	A
283	441	PRO	A
284	442	ALA	A
285	443	SER	A
286	444	ILE	A
287	445	SER	A
288	454	GLY	A
289	455	SER	A
290	457	ASN	A
291	458	ASP	A
292	459	VAL	A
293	460	LEU	A
294	483	ALA	A
295	484	ALA	A

Continued on next page

Table S28 – *Continued from previous page*

Homology index	Residue Index	Residue abbreviation	Module
296	485	GLY	A
297	486	GLY	A
298	487	THR	A
299	488	ALA	A
300	489	VAL	A
301	490	TRP	A
302	491	GLN	A

Table S29: Residues membership for the *P. haloplanctis* α-amylase (PDB code 1AQH, chain A)

Homology index	Residue Index	Residue abbreviation	Module
0	3	THR	A
1	4	THR	A
2	5	PHE	B
3	6	VAL	B
4	7	HIS	B
5	8	LEU	C
6	9	PHE	C
7	10	GLU	C
8	11	TRP	C
9	12	ASN	C
10	13	TRP	C
11	14	GLN	C
12	15	ASP	C
13	16	VAL	C
14	17	ALA	C
15	18	GLN	C
16	19	GLU	C
17	20	CYS	C
18	21	GLU	C
19	22	GLN	C
20	24	LEU	C
21	25	GLY	C
22	26	PRO	C
23	27	LYS	C
24	28	GLY	C
25	29	TYR	C
26	30	ALA	C
27	31	ALA	C
28	32	VAL	C
29	33	GLN	C
30	34	VAL	C

Continued on next page

Table S29 – *Continued from previous page*

Homology index	Residue Index	Residue abbreviation	Module
31	35	SER	C
32	36	PRO	C
33	37	PRO	C
34	38	ASN	C
35	39	GLU	C
36	40	HIS	C
37	41	ILE	C
38	43	GLY	C
39	47	TRP	C
40	48	THR	C
41	49	ARG	C
42	50	TYR	C
43	51	GLN	C
44	52	PRO	C
45	53	VAL	C
46	54	SER	C
47	55	TYR	C
48	56	GLU	C
49	57	LEU	C
50	58	GLN	C
51	59	SER	C
52	60	ARG	C
53	61	GLY	C
54	62	GLY	C
55	63	ASN	C
56	64	ARG	C
57	65	ALA	C
58	66	GLN	C
59	67	PHE	C
60	68	ILE	C
61	69	ASP	C
62	70	MET	C
63	71	VAL	C
64	72	ASN	C
65	73	ARG	C
66	74	CYS	C
67	75	SER	C
68	76	ALA	C
69	77	ALA	C
70	78	GLY	C
71	79	VAL	C
72	80	ASP	C
73	81	ILE	C
74	82	TYR	C
75	83	VAL	C
76	84	ASP	C

Continued on next page

Table S29 – *Continued from previous page*

Homology index	Residue Index	Residue abbreviation	Module
77	85	THR	C
78	86	LEU	C
79	87	ILE	C
80	88	ASN	C
81	89	HIS	C
82	90	MET	C
83	91	ALA	C
84	92	ALA	C
85	93	GLY	C
86	108	PHE	C
87	110	ILE	C
88	111	TYR	C
89	112	SER	C
90	113	PRO	C
91	114	GLN	C
92	115	ASP	C
93	116	PHE	C
94	117	HIS	B
95	137	CYS	B
96	138	GLU	B
97	139	LEU	B
98	140	VAL	B
99	142	LEU	B
100	143	ALA	B
101	144	ASP	B
102	145	LEU	B
103	146	ASP	B
104	147	THR	B
105	148	ALA	B
106	149	SER	B
107	150	ASN	B
108	151	TYR	B
109	152	VAL	B
110	153	GLN	B
111	154	ASN	B
112	155	THR	B
113	156	ILE	B
114	157	ALA	B
115	158	ALA	B
116	159	TYR	B
117	160	ILE	B
118	161	ASN	B
119	162	ASP	B
120	163	LEU	B
121	164	GLN	B
122	165	ALA	B

Continued on next page

Table S29 – *Continued from previous page*

Homology index	Residue Index	Residue abbreviation	Module
123	166	ILE	C
124	167	GLY	C
125	168	VAL	C
126	169	LYS	C
127	170	GLY	C
128	171	PHE	B
129	172	ARG	D
130	173	PHE	B
131	174	ASP	B
132	175	ALA	B
133	176	SER	B
134	177	LYS	B
135	178	HIS	B
136	179	VAL	B
137	180	ALA	B
138	181	ALA	B
139	182	SER	B
140	183	ASP	B
141	184	ILE	B
142	185	GLN	B
143	186	SER	B
144	187	LEU	B
145	188	MET	B
146	189	ALA	B
147	190	LYS	B
148	191	VAL	B
149	192	ASN	B
150	195	PRO	B
151	196	VAL	B
152	197	VAL	B
153	198	PHE	C
154	199	GLN	B
155	200	GLU	B
156	201	VAL	B
157	202	ILE	B
158	203	ASP	B
159	204	GLN	B
160	211	ALA	B
161	212	SER	B
162	213	GLU	B
163	214	TYR	B
164	215	LEU	B
165	216	SER	B
166	217	THR	B
167	218	GLY	B
168	219	LEU	B

Continued on next page

Table S29 – *Continued from previous page*

Homology index	Residue Index	Residue abbreviation	Module
169	220	VAL	B
170	221	THR	D
171	222	GLU	D
172	223	PHE	D
173	224	LYS	D
174	226	SER	D
175	227	THR	D
176	228	GLU	D
177	229	LEU	D
178	230	GLY	D
179	231	ASN	D
180	232	THR	D
181	233	PHE	D
182	236	GLY	D
183	237	SER	A
184	238	LEU	A
185	239	ALA	A
186	244	PHE	A
187	245	GLY	D
188	246	GLU	D
189	247	GLY	D
190	249	GLY	D
191	250	PHE	B
192	251	MET	B
193	252	PRO	B
194	253	SER	B
195	254	SER	B
196	255	SER	B
197	256	ALA	B
198	257	VAL	D
199	258	VAL	D
200	259	PHE	D
201	260	VAL	A
202	261	ASP	D
203	262	ASN	D
204	263	HIS	D
205	264	ASP	D
206	265	ASN	D
207	266	GLN	D
208	277	THR	D
209	278	PHE	D
210	279	GLU	D
211	280	ASP	D
212	282	ARG	A
213	283	LEU	A
214	284	TYR	A

Continued on next page

Table S29 – *Continued from previous page*

Homology index	Residue Index	Residue abbreviation	Module
215	285	ASP	A
216	286	LEU	A
217	287	ALA	A
218	288	ASN	A
219	289	VAL	A
220	290	PHE	A
221	291	MET	A
222	292	LEU	A
223	293	ALA	A
224	294	TYR	A
225	296	TYR	D
226	297	GLY	D
227	298	TYR	D
228	299	PRO	A
229	300	LYS	D
230	301	VAL	A
231	302	MET	D
232	303	SER	C
233	304	SER	D
234	305	TYR	A
235	306	ASP	C
236	332	ASN	C
237	333	TRP	D
238	334	LYS	D
239	335	CYS	D
240	338	ARG	A
241	339	TRP	A
242	340	SER	A
243	341	TYR	A
244	342	ILE	A
245	343	ALA	A
246	344	GLY	A
247	345	GLY	A
248	346	VAL	A
249	347	ASP	A
250	348	PHE	A
251	349	ARG	A
252	350	ASN	A
253	351	ASN	A
254	352	THR	A
255	353	ALA	A
256	355	ASN	A
257	356	TRP	A
258	357	ALA	A
259	358	VAL	A
260	360	ASN	A

Continued on next page

Table S29 – *Continued from previous page*

Homology index	Residue Index	Residue abbreviation	Module
261	361	TRP	A
262	362	TRP	A
263	363	ASP	A
264	365	THR	A
265	366	ASN	A
266	367	ASN	A
267	368	GLN	A
268	369	ILE	A
269	370	SER	A
270	371	PHE	A
271	372	GLY	A
272	373	ARG	A
273	374	GLY	A
274	375	SER	A
275	377	GLY	A
276	378	HIS	A
277	379	MET	A
278	380	ALA	A
279	381	ILE	A
280	382	ASN	A
281	385	ASP	A
282	386	SER	A
283	387	THR	A
284	388	LEU	A
285	389	THR	A
286	390	ALA	A
287	391	THR	A
288	397	ALA	A
289	398	SER	A
290	401	TYR	A
291	402	CYS	A
292	403	ASN	A
293	404	VAL	A
294	433	ILE	A
295	434	GLY	A
296	435	ALA	A
297	437	ASP	A
298	438	ALA	A
299	439	MET	A
300	440	ALA	A
301	441	ILE	A
302	442	HIS	A

Table S30: Residues membership for the *H. vulgare* α-amylase (PDB code 1AMY, chain A)

Homology index	Residue Index	Residue abbreviation	Module
0	1	GLN	A
1	2	VAL	A
2	3	LEU	B
3	4	PHE	B
4	5	GLN	B
5	6	GLY	C
6	7	PHE	C
7	11	SER	C
8	17	GLY	C
9	18	TRP	C
10	19	TYR	C
11	20	ASN	C
12	21	PHE	C
13	22	LEU	C
14	23	MET	C
15	24	GLY	C
16	25	LYS	C
17	26	VAL	C
18	27	ASP	C
19	28	ASP	C
20	29	ILE	C
21	30	ALA	C
22	31	ALA	C
23	32	ALA	C
24	33	GLY	C
25	34	ILE	C
26	35	THR	C
27	36	HIS	C
28	37	VAL	C
29	38	TRP	C
30	39	LEU	C
31	40	PRO	C
32	41	PRO	C
33	42	ALA	C
34	43	SER	C
35	44	GLN	C
36	45	SER	C
37	46	VAL	C
38	47	ALA	C
39	48	GLU	C
40	49	GLN	C
41	50	GLY	C
42	51	TYR	C
43	52	MET	C

Continued on next page

Table S30 – *Continued from previous page*

Homology index	Residue Index	Residue abbreviation	Module
44	53	PRO	C
45	54	GLY	C
46	55	ARG	C
47	57	TYR	C
48	58	ASP	C
49	59	LEU	C
50	60	ASP	C
51	62	SER	C
52	63	LYS	C
53	64	TYR	C
54	65	GLY	C
55	66	ASN	C
56	67	LYS	C
57	68	ALA	C
58	69	GLN	C
59	70	LEU	C
60	71	LYS	C
61	72	SER	C
62	73	LEU	C
63	74	ILE	C
64	75	GLY	C
65	76	ALA	C
66	77	LEU	C
67	78	HIS	C
68	79	GLY	C
69	80	LYS	C
70	81	GLY	C
71	82	VAL	C
72	83	LYS	C
73	84	ALA	C
74	85	ILE	C
75	86	ALA	C
76	87	ASP	C
77	88	ILE	C
78	89	VAL	C
79	90	ILE	C
80	91	ASN	C
81	92	HIS	C
82	93	ARG	C
83	94	THR	C
84	95	ALA	C
85	96	GLU	C
86	107	PHE	C
87	117	ASP	C
88	118	TRP	C
89	119	GLY	C

Continued on next page

Table S30 – *Continued from previous page*

Homology index	Residue Index	Residue abbreviation	Module
90	120	PRO	C
91	121	HIS	C
92	122	MET	C
93	123	ILE	C
94	124	CYS	B
95	141	ALA	B
96	142	ASP	B
97	143	PHE	B
98	144	GLY	B
99	146	ALA	B
100	147	PRO	B
101	148	ASP	B
102	149	ILE	B
103	150	ASP	B
104	151	HIS	B
105	152	LEU	B
106	153	ASN	B
107	154	LEU	B
108	155	ARG	B
109	156	VAL	B
110	157	GLN	B
111	158	LYS	B
112	159	GLU	B
113	160	LEU	B
114	161	VAL	B
115	162	GLU	B
116	163	TRP	B
117	164	LEU	B
118	165	ASN	B
119	166	TRP	B
120	167	LEU	B
121	168	LYS	B
122	169	ALA	B
123	171	ILE	C
124	172	GLY	C
125	173	PHE	C
126	174	ASP	C
127	175	GLY	C
128	176	TRP	B
129	177	ARG	D
130	178	PHE	B
131	179	ASP	B
132	180	PHE	B
133	181	ALA	B
134	182	LYS	B
135	183	GLY	B

Continued on next page

Table S30 – *Continued from previous page*

Homology index	Residue Index	Residue abbreviation	Module
136	184	TYR	B
137	185	SER	B
138	186	ALA	B
139	187	ASP	B
140	188	VAL	B
141	189	ALA	B
142	190	LYS	B
143	191	ILE	B
144	192	TYR	B
145	193	ILE	B
146	194	ASP	B
147	195	ARG	B
148	196	SER	B
149	197	GLU	B
150	199	SER	B
151	200	PHE	B
152	201	ALA	B
153	202	VAL	C
154	203	ALA	B
155	204	GLU	B
156	205	ILE	B
157	206	TRP	B
158	207	THR	B
159	208	SER	B
160	228	LEU	B
161	229	VAL	B
162	230	ASN	B
163	231	TRP	B
164	232	VAL	B
165	233	ASP	B
166	240	PRO	B
167	241	ALA	B
168	242	THR	B
169	243	THR	B
170	244	PHE	D
171	245	ASP	D
172	246	PHE	D
173	247	THR	D
174	249	LYS	D
175	250	GLY	D
176	251	ILE	D
177	252	LEU	D
178	253	ASN	D
179	254	VAL	D
180	255	ALA	D
181	256	VAL	D

Continued on next page

Table S30 – *Continued from previous page*

Homology index	Residue Index	Residue abbreviation	Module
182	259	GLU	D
183	260	LEU	A
184	261	TRP	A
185	262	ARG	A
186	269	LYS	A
187	270	ALA	D
188	271	PRO	D
189	272	GLY	D
190	274	ILE	D
191	275	GLY	B
192	276	TRP	B
193	277	TRP	B
194	278	PRO	B
195	279	ALA	B
196	280	LYS	B
197	281	ALA	B
198	282	VAL	D
199	283	THR	D
200	284	PHE	D
201	285	VAL	A
202	286	ASP	D
203	287	ASN	D
204	288	HIS	D
205	289	ASP	D
206	290	THR	D
207	291	GLY	D
208	298	PRO	D
209	299	PHE	D
210	300	PRO	D
211	301	SER	D
212	302	ASP	A
213	303	ARG	A
214	304	VAL	A
215	305	MET	A
216	306	GLN	A
217	307	GLY	A
218	308	TYR	A
219	309	ALA	A
220	310	TYR	A
221	311	ILE	A
222	312	LEU	A
223	313	THR	A
224	314	HIS	A
225	315	PRO	D
226	316	GLY	D
227	317	THR	D

Continued on next page

Table S30 – *Continued from previous page*

Homology index	Residue Index	Residue abbreviation	Module
228	318	PRO	A
229	319	CYS	D
230	320	ILE	A
231	321	PHE	D
232	322	TYR	C
233	323	ASP	D
234	324	HIS	A
235	325	PHE	C
236	326	PHE	C
237	327	ASP	D
238	328	TRP	D
239	329	GLY	D
240	330	LEU	A
241	331	LYS	A
242	332	GLU	A
243	333	GLU	A
244	334	ILE	A
245	335	ASP	A
246	336	ARG	A
247	337	LEU	A
248	338	VAL	A
249	339	SER	A
250	340	VAL	A
251	341	ARG	A
252	342	THR	A
253	343	ARG	A
254	344	HIS	A
255	345	GLY	A
256	349	GLU	A
257	350	SER	A
258	351	LYS	A
259	352	LEU	A
260	354	ILE	A
261	355	ILE	A
262	356	GLU	A
263	357	ALA	A
264	358	ASP	A
265	359	ALA	A
266	360	ASP	A
267	361	LEU	A
268	362	TYR	A
269	363	LEU	A
270	364	ALA	A
271	365	GLU	A
272	366	ILE	A
273	368	GLY	A

Continued on next page

Table S30 – *Continued from previous page*

Homology index	Residue Index	Residue abbreviation	Module
274	369	LYS	A
275	370	VAL	A
276	371	ILE	A
277	372	VAL	A
278	373	LYS	A
279	374	LEU	A
280	375	GLY	A
281	376	PRO	A
282	377	ARG	A
283	378	TYR	A
284	379	ASP	A
285	380	VAL	A
286	381	GLY	A
287	382	ASN	A
288	387	GLY	A
289	388	PHE	A
290	389	LYS	A
291	390	VAL	A
292	391	ALA	A
293	392	ALA	A
294	393	HIS	A
295	394	GLY	A
296	395	ASN	A
297	396	ASP	A
298	397	TYR	A
299	398	ALA	A
300	399	VAL	A
301	400	TRP	A
302	401	GLU	A

Table S31: Residues membership for the *H. vulgare* α-amylase (PDB code 1AVA, chain A)

Homology index	Residue Index	Residue abbreviation	Module
0	1	GLN	A
1	2	VAL	A
2	3	LEU	B
3	4	PHE	B
4	5	GLN	B
5	6	GLY	C
6	7	PHE	C
7	11	SER	C
8	17	GLY	C

Continued on next page

Table S31 – *Continued from previous page*

Homology index	Residue Index	Residue abbreviation	Module
9	18	TRP	C
10	19	TYR	C
11	20	ASN	C
12	21	PHE	C
13	22	LEU	C
14	23	MET	C
15	24	GLY	C
16	25	LYS	C
17	26	VAL	C
18	27	ASP	C
19	28	ASP	C
20	29	ILE	C
21	30	ALA	C
22	31	ALA	C
23	32	ALA	C
24	33	GLY	C
25	34	ILE	C
26	35	THR	C
27	36	HIS	C
28	37	VAL	C
29	38	TRP	C
30	39	LEU	C
31	40	PRO	C
32	41	PRO	C
33	42	ALA	C
34	43	SER	C
35	44	GLN	C
36	45	SER	C
37	46	VAL	C
38	47	ALA	C
39	48	GLU	C
40	49	GLN	C
41	50	GLY	C
42	51	TYR	C
43	52	MET	C
44	53	PRO	C
45	54	GLY	C
46	55	ARG	C
47	57	TYR	C
48	58	ASP	C
49	59	LEU	C
50	60	ASP	C
51	62	SER	C
52	63	LYS	C
53	64	TYR	C
54	65	GLY	C

Continued on next page

Table S31 – *Continued from previous page*

**Homology index**	**Residue Index**	**Residue abbreviation**	**Module**
55	66	ASN	C
56	67	LYS	C
57	68	ALA	C
58	69	GLN	C
59	70	LEU	C
60	71	LYS	C
61	72	SER	C
62	73	LEU	C
63	74	ILE	C
64	75	GLY	C
65	76	ALA	C
66	77	LEU	C
67	78	HIS	C
68	79	GLY	C
69	80	LYS	C
70	81	GLY	C
71	82	VAL	C
72	83	LYS	C
73	84	ALA	C
74	85	ILE	C
75	86	ALA	C
76	87	ASP	C
77	88	ILE	C
78	89	VAL	C
79	90	ILE	C
80	91	ASN	C
81	92	HIS	C
82	93	ARG	C
83	94	THR	C
84	95	ALA	C
85	96	GLU	C
86	107	PHE	C
87	117	ASP	C
88	118	TRP	C
89	119	GLY	C
90	120	PRO	C
91	121	HIS	C
92	122	MET	C
93	123	ILE	C
94	124	CYS	B
95	141	ALA	B
96	142	ASP	B
97	143	PHE	B
98	144	GLY	B
99	146	ALA	B
100	147	PRO	B

Continued on next page

Table S31 – *Continued from previous page*

**Homology index**	**Residue Index**	**Residue abbreviation**	**Module**
101	148	ASP	B
102	149	ILE	B
103	150	ASP	B
104	151	HIS	B
105	152	LEU	B
106	153	ASN	B
107	154	LEU	B
108	155	ARG	B
109	156	VAL	B
110	157	GLN	B
111	158	LYS	B
112	159	GLU	B
113	160	LEU	B
114	161	VAL	B
115	162	GLU	B
116	163	TRP	B
117	164	LEU	B
118	165	ASN	B
119	166	TRP	B
120	167	LEU	B
121	168	LYS	B
122	169	ALA	B
123	171	ILE	C
124	172	GLY	C
125	173	PHE	C
126	174	ASP	C
127	175	GLY	C
128	176	TRP	B
129	177	ARG	D
130	178	PHE	B
131	179	ASP	B
132	180	PHE	B
133	181	ALA	B
134	182	LYS	B
135	183	GLY	B
136	184	TYR	B
137	185	SER	B
138	186	ALA	B
139	187	ASP	B
140	188	VAL	B
141	189	ALA	B
142	190	LYS	B
143	191	ILE	B
144	192	TYR	B
145	193	ILE	B
146	194	ASP	B

Continued on next page

Table S31 – *Continued from previous page*

Homology index	Residue Index	Residue abbreviation	Module
147	195	ARG	B
148	196	SER	B
149	197	GLU	B
150	199	SER	B
151	200	PHE	B
152	201	ALA	B
153	202	VAL	C
154	203	ALA	B
155	204	GLU	B
156	205	ILE	B
157	206	TRP	B
158	207	THR	B
159	208	SER	B
160	228	LEU	B
161	229	VAL	B
162	230	ASN	B
163	231	TRP	B
164	232	VAL	B
165	233	ASP	B
166	240	PRO	B
167	241	ALA	B
168	242	THR	B
169	243	THR	B
170	244	PHE	D
171	245	ASP	D
172	246	PHE	D
173	247	THR	D
174	249	LYS	D
175	250	GLY	D
176	251	ILE	D
177	252	LEU	D
178	253	ASN	D
179	254	VAL	D
180	255	ALA	D
181	256	VAL	D
182	259	GLU	D
183	260	LEU	A
184	261	TRP	A
185	262	ARG	A
186	269	LYS	A
187	270	ALA	D
188	271	PRO	D
189	272	GLY	D
190	274	ILE	D
191	275	GLY	B
192	276	TRP	B

Continued on next page

Table S31 – *Continued from previous page*

**Homology index**	**Residue Index**	**Residue abbreviation**	**Module**
193	277	TRP	B
194	278	PRO	B
195	279	ALA	B
196	280	LYS	B
197	281	ALA	B
198	282	VAL	D
199	283	THR	D
200	284	PHE	D
201	285	VAL	A
202	286	ASP	D
203	287	ASN	D
204	288	HIS	D
205	289	ASP	D
206	290	THR	D
207	291	GLY	D
208	298	PRO	D
209	299	PHE	D
210	300	PRO	D
211	301	SER	D
212	302	ASP	A
213	303	ARG	A
214	304	VAL	A
215	305	MET	A
216	306	GLN	A
217	307	GLY	A
218	308	TYR	A
219	309	ALA	A
220	310	TYR	A
221	311	ILE	A
222	312	LEU	A
223	313	THR	A
224	314	HIS	A
225	315	PRO	D
226	316	GLY	D
227	317	THR	D
228	318	PRO	A
229	319	CYS	D
230	320	ILE	A
231	321	PHE	D
232	322	TYR	C
233	323	ASP	D
234	324	HIS	A
235	325	PHE	C
236	326	PHE	C
237	327	ASP	D
238	328	TRP	D

Continued on next page

Table S31 – *Continued from previous page*

Homology index	Residue Index	Residue abbreviation	Module
239	329	GLY	D
240	330	LEU	A
241	331	LYS	A
242	332	GLU	A
243	333	GLU	A
244	334	ILE	A
245	335	ASP	A
246	336	ARG	A
247	337	LEU	A
248	338	VAL	A
249	339	SER	A
250	340	VAL	A
251	341	ARG	A
252	342	THR	A
253	343	ARG	A
254	344	HIS	A
255	345	GLY	A
256	349	GLU	A
257	350	SER	A
258	351	LYS	A
259	352	LEU	A
260	354	ILE	A
261	355	ILE	A
262	356	GLU	A
263	357	ALA	A
264	358	ASP	A
265	359	ALA	A
266	360	ASP	A
267	361	LEU	A
268	362	TYR	A
269	363	LEU	A
270	364	ALA	A
271	365	GLU	A
272	366	ILE	A
273	368	GLY	A
274	369	LYS	A
275	370	VAL	A
276	371	ILE	A
277	372	VAL	A
278	373	LYS	A
279	374	LEU	A
280	375	GLY	A
281	376	PRO	A
282	377	ARG	A
283	378	TYR	A
284	379	ASP	A

Continued on next page

Table S31 – *Continued from previous page*

Homology index	Residue Index	Residue abbreviation	Module
285	380	VAL	A
286	381	GLY	A
287	382	ASN	A
288	387	GLY	A
289	388	PHE	A
290	389	LYS	A
291	390	VAL	A
292	391	ALA	A
293	392	ALA	A
294	393	HIS	A
295	394	GLY	A
296	395	ASN	A
297	396	ASP	A
298	397	TYR	A
299	398	ALA	A
300	399	VAL	A
301	400	TRP	A
302	401	GLU	A

Table S32: Residues membership for the *P. haloplanctis* α-amylase (PDB code 1AQM, chain A)

Homology index	Residue Index	Residue abbreviation	Module
0	3	THR	A
1	4	THR	A
2	5	PHE	B
3	6	VAL	B
4	7	HIS	B
5	8	LEU	C
6	9	PHE	C
7	10	GLU	C
8	11	TRP	C
9	12	ASN	C
10	13	TRP	C
11	14	GLN	C
12	15	ASP	C
13	16	VAL	C
14	17	ALA	C
15	18	GLN	C
16	19	GLU	C
17	20	CYS	C
18	21	GLU	C
19	22	GLN	C

Continued on next page

Table S32 – *Continued from previous page*

Homology index	Residue Index	Residue abbreviation	Module
20	24	LEU	C
21	25	GLY	C
22	26	PRO	C
23	27	LYS	C
24	28	GLY	C
25	29	TYR	C
26	30	ALA	C
27	31	ALA	C
28	32	VAL	C
29	33	GLN	C
30	34	VAL	C
31	35	SER	C
32	36	PRO	C
33	37	PRO	C
34	38	ASN	C
35	39	GLU	C
36	40	HIS	C
37	41	ILE	C
38	43	GLY	C
39	47	TRP	C
40	48	THR	C
41	49	ARG	C
42	50	TYR	C
43	51	GLN	C
44	52	PRO	C
45	53	VAL	C
46	54	SER	C
47	55	TYR	C
48	56	GLU	C
49	57	LEU	C
50	58	GLN	C
51	59	SER	C
52	60	ARG	C
53	61	GLY	C
54	62	GLY	C
55	63	ASN	C
56	64	ARG	C
57	65	ALA	C
58	66	GLN	C
59	67	PHE	C
60	68	ILE	C
61	69	ASP	C
62	70	MET	C
63	71	VAL	C
64	72	ASN	C
65	73	ARG	C

Continued on next page

Table S32 – *Continued from previous page*

Homology index	Residue Index	Residue abbreviation	Module
66	74	CYS	C
67	75	SER	C
68	76	ALA	C
69	77	ALA	C
70	78	GLY	C
71	79	VAL	C
72	80	ASP	C
73	81	ILE	C
74	82	TYR	C
75	83	VAL	C
76	84	ASP	C
77	85	THR	C
78	86	LEU	C
79	87	ILE	C
80	88	ASN	C
81	89	HIS	C
82	90	MET	C
83	91	ALA	C
84	92	ALA	C
85	93	GLY	C
86	108	PHE	C
87	110	ILE	C
88	111	TYR	C
89	112	SER	C
90	113	PRO	C
91	114	GLN	C
92	115	ASP	C
93	116	PHE	C
94	117	HIS	B
95	137	CYS	B
96	138	GLU	B
97	139	LEU	B
98	140	VAL	B
99	142	LEU	B
100	143	ALA	B
101	144	ASP	B
102	145	LEU	B
103	146	ASP	B
104	147	THR	B
105	148	ALA	B
106	149	SER	B
107	150	ASN	B
108	151	TYR	B
109	152	VAL	B
110	153	GLN	B
111	154	ASN	B

Continued on next page

Table S32 – *Continued from previous page*

Homology index	Residue Index	Residue abbreviation	Module
112	155	THR	B
113	156	ILE	B
114	157	ALA	B
115	158	ALA	B
116	159	TYR	B
117	160	ILE	B
118	161	ASN	B
119	162	ASP	B
120	163	LEU	B
121	164	GLN	B
122	165	ALA	B
123	166	ILE	C
124	167	GLY	C
125	168	VAL	C
126	169	LYS	C
127	170	GLY	C
128	171	PHE	B
129	172	ARG	D
130	173	PHE	B
131	174	ASP	B
132	175	ALA	B
133	176	SER	B
134	177	LYS	B
135	178	HIS	B
136	179	VAL	B
137	180	ALA	B
138	181	ALA	B
139	182	SER	B
140	183	ASP	B
141	184	ILE	B
142	185	GLN	B
143	186	SER	B
144	187	LEU	B
145	188	MET	B
146	189	ALA	B
147	190	LYS	B
148	191	VAL	B
149	192	ASN	B
150	195	PRO	B
151	196	VAL	B
152	197	VAL	B
153	198	PHE	C
154	199	GLN	B
155	200	GLU	B
156	201	VAL	B
157	202	ILE	B

Continued on next page

Table S32 – *Continued from previous page*

Homology index	Residue Index	Residue abbreviation	Module
158	203	ASP	B
159	204	GLN	B
160	211	ALA	B
161	212	SER	B
162	213	GLU	B
163	214	TYR	B
164	215	LEU	B
165	216	SER	B
166	217	THR	B
167	218	GLY	B
168	219	LEU	B
169	220	VAL	B
170	221	THR	D
171	222	GLU	D
172	223	PHE	D
173	224	LYS	D
174	226	SER	D
175	227	THR	D
176	228	GLU	D
177	229	LEU	D
178	230	GLY	D
179	231	ASN	D
180	232	THR	D
181	233	PHE	D
182	236	GLY	D
183	237	SER	A
184	238	LEU	A
185	239	ALA	A
186	244	PHE	A
187	245	GLY	D
188	246	GLU	D
189	247	GLY	D
190	249	GLY	D
191	250	PHE	B
192	251	MET	B
193	252	PRO	B
194	253	SER	B
195	254	SER	B
196	255	SER	B
197	256	ALA	B
198	257	VAL	D
199	258	VAL	D
200	259	PHE	D
201	260	VAL	A
202	261	ASP	D
203	262	ASN	D

Continued on next page

Table S32 – *Continued from previous page*

Homology index	Residue Index	Residue abbreviation	Module
204	263	HIS	D
205	264	ASP	D
206	265	ASN	D
207	266	GLN	D
208	277	THR	D
209	278	PHE	D
210	279	GLU	D
211	280	ASP	D
212	282	ARG	A
213	283	LEU	A
214	284	TYR	A
215	285	ASP	A
216	286	LEU	A
217	287	ALA	A
218	288	ASN	A
219	289	VAL	A
220	290	PHE	A
221	291	MET	A
222	292	LEU	A
223	293	ALA	A
224	294	TYR	A
225	296	TYR	D
226	297	GLY	D
227	298	TYR	D
228	299	PRO	A
229	300	LYS	D
230	301	VAL	A
231	302	MET	D
232	303	SER	C
233	304	SER	D
234	305	TYR	A
235	306	ASP	C
236	332	ASN	C
237	333	TRP	D
238	334	LYS	D
239	335	CYS	D
240	338	ARG	A
241	339	TRP	A
242	340	SER	A
243	341	TYR	A
244	342	ILE	A
245	343	ALA	A
246	344	GLY	A
247	345	GLY	A
248	346	VAL	A
249	347	ASP	A

Continued on next page

Table S32 – *Continued from previous page*

Homology index	Residue Index	Residue abbreviation	Module
250	348	PHE	A
251	349	ARG	A
252	350	ASN	A
253	351	ASN	A
254	352	THR	A
255	353	ALA	A
256	355	ASN	A
257	356	TRP	A
258	357	ALA	A
259	358	VAL	A
260	360	ASN	A
261	361	TRP	A
262	362	TRP	A
263	363	ASP	A
264	365	THR	A
265	366	ASN	A
266	367	ASN	A
267	368	GLN	A
268	369	ILE	A
269	370	SER	A
270	371	PHE	A
271	372	GLY	A
272	373	ARG	A
273	374	GLY	A
274	375	SER	A
275	377	GLY	A
276	378	HIS	A
277	379	MET	A
278	380	ALA	A
279	381	ILE	A
280	382	ASN	A
281	385	ASP	A
282	386	SER	A
283	387	THR	A
284	388	LEU	A
285	389	THR	A
286	390	ALA	A
287	391	THR	A
288	397	ALA	A
289	398	SER	A
290	401	TYR	A
291	402	CYS	A
292	403	ASN	A
293	404	VAL	A
294	433	ILE	A
295	434	GLY	A

Continued on next page

Table S32 – *Continued from previous page*

Homology index	Residue Index	Residue abbreviation	Module
296	435	ALA	A
297	437	ASP	A
298	438	ALA	A
299	439	MET	A
300	440	ALA	A
301	441	ILE	A
302	442	HIS	A

Table S33: Residues membership for the *B. subtilis* α-amylase (PDB code 1BAG, chain A)

Homology index	Residue Index	Residue abbreviation	Module
0	9	GLY	A
1	10	THR	A
2	11	ILE	B
3	12	LEU	B
4	13	HIS	B
5	14	ALA	C
6	15	TRP	C
7	16	ASN	C
8	17	TRP	C
9	18	SER	C
10	19	PHE	C
11	20	ASN	C
12	21	THR	C
13	22	LEU	C
14	23	LYS	C
15	24	HIS	C
16	25	ASN	C
17	26	MET	C
18	27	LYS	C
19	28	ASP	C
20	29	ILE	C
21	30	HIS	C
22	31	ASP	C
23	32	ALA	C
24	33	GLY	C
25	34	TYR	C
26	35	THR	C
27	36	ALA	C
28	37	ILE	C
29	38	GLN	C
30	39	THR	C

Continued on next page

Table S33 – *Continued from previous page*

Homology index	Residue Index	Residue abbreviation	Module
31	40	SER	C
32	41	PRO	C
33	42	ILE	C
34	43	ASN	C
35	44	GLN	C
36	45	VAL	C
37	46	LYS	C
38	48	GLY	C
39	59	TYR	C
40	60	TRP	C
41	61	LEU	C
42	62	TYR	C
43	63	GLN	C
44	64	PRO	C
45	65	THR	C
46	66	SER	C
47	67	TYR	C
48	68	GLN	C
49	69	ILE	C
50	70	GLY	C
51	72	ARG	C
52	73	TYR	C
53	74	LEU	C
54	75	GLY	C
55	76	THR	C
56	77	GLU	C
57	78	GLN	C
58	79	GLU	C
59	80	PHE	C
60	81	LYS	C
61	82	GLU	C
62	83	MET	C
63	84	CYS	C
64	85	ALA	C
65	86	ALA	C
66	87	ALA	C
67	88	GLU	C
68	89	GLU	C
69	90	TYR	C
70	91	GLY	C
71	92	ILE	C
72	93	LYS	C
73	94	VAL	C
74	95	ILE	C
75	96	VAL	C
76	97	ASP	C

Continued on next page

Table S33 – *Continued from previous page*

Homology index	Residue Index	Residue abbreviation	Module
77	98	ALA	C
78	99	VAL	C
79	100	ILE	C
80	101	ASN	C
81	102	HIS	C
82	103	THR	C
83	104	THR	C
84	105	PHE	C
85	106	ASP	C
86	111	SER	C
87	113	GLU	C
88	114	VAL	C
89	115	LYS	C
90	118	PRO	C
91	119	ASN	C
92	120	TRP	C
93	121	THR	C
94	122	HIS	B
95	139	ASN	B
96	140	SER	B
97	141	LEU	B
98	142	LEU	B
99	144	LEU	B
100	145	TYR	B
101	146	ASP	B
102	147	TRP	B
103	148	ASN	B
104	149	THR	B
105	150	GLN	B
106	151	ASN	B
107	152	THR	B
108	153	GLN	B
109	154	VAL	B
110	155	GLN	B
111	156	SER	B
112	157	TYR	B
113	158	LEU	B
114	159	LYS	B
115	160	ARG	B
116	161	PHE	B
117	162	LEU	B
118	163	GLU	B
119	164	ARG	B
120	165	ALA	B
121	166	LEU	B
122	167	ASN	B

Continued on next page

Table S33 – *Continued from previous page*

Homology index	Residue Index	Residue abbreviation	Module
123	168	ASP	C
124	169	GLY	C
125	170	ALA	C
126	171	ASP	C
127	172	GLY	C
128	173	PHE	B
129	174	ARG	D
130	175	PHE	B
131	176	ASP	B
132	177	ALA	B
133	178	ALA	B
134	179	LYS	B
135	180	HIS	B
136	181	ILE	B
137	182	GLU	B
138	191	SER	B
139	192	GLN	B
140	193	PHE	B
141	194	TRP	B
142	195	PRO	B
143	196	ASN	B
144	197	ILE	B
145	198	THR	B
146	199	ASN	B
147	200	THR	B
148	201	SER	B
149	202	ALA	B
150	203	GLU	B
151	204	PHE	B
152	205	GLN	B
153	206	TYR	C
154	207	GLY	B
155	208	GLN	B
156	209	ILE	B
157	210	LEU	B
158	211	GLN	B
159	212	ASP	B
160	217	ASP	B
161	218	ALA	B
162	219	ALA	B
163	220	TYR	B
164	221	ALA	B
165	222	ASN	B
166	223	TYR	B
167	224	MET	B
168	225	ASP	B

Continued on next page

Table S33 – *Continued from previous page*

Homology index	Residue Index	Residue abbreviation	Module
169	226	VAL	B
170	227	THR	D
171	228	ALA	D
172	229	SER	D
173	230	ASN	D
174	232	GLY	D
175	233	HIS	D
176	234	SER	D
177	235	ILE	D
178	236	ARG	D
179	237	SER	D
180	238	ALA	D
181	239	LEU	D
182	243	ASN	D
183	244	LEU	A
184	245	GLY	A
185	246	VAL	A
186	249	ILE	A
187	250	SER	D
188	251	HIS	D
189	252	TYR	D
190	253	ALA	D
191	254	SER	B
192	255	ASP	B
193	257	SER	B
194	258	ALA	B
195	259	ASP	B
196	260	LYS	B
197	261	LEU	B
198	262	VAL	D
199	263	THR	D
200	264	TRP	D
201	265	VAL	A
202	266	GLU	D
203	267	SER	D
204	268	HIS	D
205	269	ASP	D
206	270	THR	D
207	271	TYR	D
208	279	THR	D
209	280	TRP	D
210	281	MET	D
211	282	SER	D
212	284	ASP	A
213	285	ASP	A
214	286	ILE	A

Continued on next page

Table S33 – *Continued from previous page*

Homology index	Residue Index	Residue abbreviation	Module
215	287	ARG	A
216	288	LEU	A
217	289	GLY	A
218	290	TRP	A
219	291	ALA	A
220	292	VAL	A
221	293	ILE	A
222	294	ALA	A
223	295	SER	A
224	296	ARG	A
225	297	SER	D
226	299	SER	D
227	300	THR	D
228	301	PRO	A
229	302	LEU	D
230	303	PHE	A
231	304	PHE	D
232	305	SER	C
233	306	ARG	D
234	307	PRO	A
235	316	PHE	C
236	325	ARG	C
237	326	GLY	D
238	327	SER	D
239	328	ALA	D
240	331	GLU	A
241	332	ASP	A
242	333	GLN	A
243	334	ALA	A
244	335	ILE	A
245	336	THR	A
246	337	ALA	A
247	338	VAL	A
248	339	ASN	A
249	340	ARG	A
250	341	PHE	A
251	342	HIS	A
252	343	ASN	A
253	344	VAL	A
254	345	MET	A
255	346	ALA	A
256	347	GLY	A
257	348	GLN	A
258	349	PRO	A
259	350	GLU	A
260	351	GLU	A

Continued on next page

Table S33 – *Continued from previous page*

Homology index	Residue Index	Residue abbreviation	Module
261	352	LEU	A
262	353	SER	A
263	354	ASN	A
264	357	GLY	A
265	358	ASN	A
266	359	ASN	A
267	360	GLN	A
268	362	PHE	A
269	363	MET	A
270	364	ASN	A
271	365	GLN	A
272	366	ARG	A
273	367	GLY	A
274	368	SER	A
275	370	GLY	A
276	371	VAL	A
277	372	VAL	A
278	373	LEU	A
279	374	ALA	A
280	375	ASN	A
281	378	SER	A
282	379	SER	A
283	380	SER	A
284	381	VAL	A
285	383	ILE	A
286	384	ASN	A
287	385	THR	A
288	391	ASP	A
289	392	GLY	A
290	394	TYR	A
291	395	ASP	A
292	396	ASN	A
293	397	LYS	A
294	414	ILE	A
295	415	ASN	A
296	416	ALA	A
297	418	SER	A
298	419	VAL	A
299	420	ALA	A
300	421	VAL	A
301	422	LEU	A
302	423	TYR	A

Table S34: Residues membership for the *P. haloplanctis* α-amylase (PDB code 1B0I, chain A)

Homology index	Residue Index	Residue abbreviation	Module
0	3	THR	A
1	4	THR	A
2	5	PHE	B
3	6	VAL	B
4	7	HIS	B
5	8	LEU	C
6	9	PHE	C
7	10	GLU	C
8	11	TRP	C
9	12	ASN	C
10	13	TRP	C
11	14	GLN	C
12	15	ASP	C
13	16	VAL	C
14	17	ALA	C
15	18	GLN	C
16	19	GLU	C
17	20	CYS	C
18	21	GLU	C
19	22	GLN	C
20	24	LEU	C
21	25	GLY	C
22	26	PRO	C
23	27	LYS	C
24	28	GLY	C
25	29	TYR	C
26	30	ALA	C
27	31	ALA	C
28	32	VAL	C
29	33	GLN	C
30	34	VAL	C
31	35	SER	C
32	36	PRO	C
33	37	PRO	C
34	38	ASN	C
35	39	GLU	C
36	40	HIS	C
37	41	ILE	C
38	43	GLY	C
39	47	TRP	C
40	48	THR	C
41	49	ARG	C
42	50	TYR	C
43	51	GLN	C

Continued on next page

Table S34 – *Continued from previous page*

Homology index	Residue Index	Residue abbreviation	Module
44	52	PRO	C
45	53	VAL	C
46	54	SER	C
47	55	TYR	C
48	56	GLU	C
49	57	LEU	C
50	58	GLN	C
51	59	SER	C
52	60	ARG	C
53	61	GLY	C
54	62	GLY	C
55	63	ASN	C
56	64	ARG	C
57	65	ALA	C
58	66	GLN	C
59	67	PHE	C
60	68	ILE	C
61	69	ASP	C
62	70	MET	C
63	71	VAL	C
64	72	ASN	C
65	73	ARG	C
66	74	CYS	C
67	75	SER	C
68	76	ALA	C
69	77	ALA	C
70	78	GLY	C
71	79	VAL	C
72	80	ASP	C
73	81	ILE	C
74	82	TYR	C
75	83	VAL	C
76	84	ASP	C
77	85	THR	C
78	86	LEU	C
79	87	ILE	C
80	88	ASN	C
81	89	HIS	C
82	90	MET	C
83	91	ALA	C
84	92	ALA	C
85	93	GLY	C
86	108	PHE	C
87	110	ILE	C
88	111	TYR	C
89	112	SER	C

Continued on next page

Table S34 – *Continued from previous page*

Homology index	Residue Index	Residue abbreviation	Module
90	113	PRO	C
91	114	GLN	C
92	115	ASP	C
93	116	PHE	C
94	117	HIS	B
95	137	CYS	B
96	138	GLU	B
97	139	LEU	B
98	140	VAL	B
99	142	LEU	B
100	143	ALA	B
101	144	ASP	B
102	145	LEU	B
103	146	ASP	B
104	147	THR	B
105	148	ALA	B
106	149	SER	B
107	150	ASN	B
108	151	TYR	B
109	152	VAL	B
110	153	GLN	B
111	154	ASN	B
112	155	THR	B
113	156	ILE	B
114	157	ALA	B
115	158	ALA	B
116	159	TYR	B
117	160	ILE	B
118	161	ASN	B
119	162	ASP	B
120	163	LEU	B
121	164	GLN	B
122	165	ALA	B
123	166	ILE	C
124	167	GLY	C
125	168	VAL	C
126	169	LYS	C
127	170	GLY	C
128	171	PHE	B
129	172	ARG	D
130	173	PHE	B
131	174	ASP	B
132	175	ALA	B
133	176	SER	B
134	177	LYS	B
135	178	HIS	B

Continued on next page

Table S34 – *Continued from previous page*

Homology index	Residue Index	Residue abbreviation	Module
136	179	VAL	B
137	180	ALA	B
138	181	ALA	B
139	182	SER	B
140	183	ASP	B
141	184	ILE	B
142	185	GLN	B
143	186	SER	B
144	187	LEU	B
145	188	MET	B
146	189	ALA	B
147	190	LYS	B
148	191	VAL	B
149	192	ASN	B
150	195	PRO	B
151	196	VAL	B
152	197	VAL	B
153	198	PHE	C
154	199	GLN	B
155	200	GLU	B
156	201	VAL	B
157	202	ILE	B
158	203	ASP	B
159	204	GLN	B
160	211	ALA	B
161	212	SER	B
162	213	GLU	B
163	214	TYR	B
164	215	LEU	B
165	216	SER	B
166	217	THR	B
167	218	GLY	B
168	219	LEU	B
169	220	VAL	B
170	221	THR	D
171	222	GLU	D
172	223	PHE	D
173	224	LYS	D
174	226	SER	D
175	227	THR	D
176	228	GLU	D
177	229	LEU	D
178	230	GLY	D
179	231	ASN	D
180	232	THR	D
181	233	PHE	D

Continued on next page

Table S34 – *Continued from previous page*

Homology index	Residue Index	Residue abbreviation	Module
182	236	GLY	D
183	237	SER	A
184	238	LEU	A
185	239	ALA	A
186	244	PHE	A
187	245	GLY	D
188	246	GLU	D
189	247	GLY	D
190	249	GLY	D
191	250	PHE	B
192	251	MET	B
193	252	PRO	B
194	253	SER	B
195	254	SER	B
196	255	SER	B
197	256	ALA	B
198	257	VAL	D
199	258	VAL	D
200	259	PHE	D
201	260	VAL	A
202	261	ASP	D
203	262	ASN	D
204	263	HIS	D
205	264	ASP	D
206	265	ASN	D
207	266	GLN	D
208	277	THR	D
209	278	PHE	D
210	279	GLU	D
211	280	ASP	D
212	282	ARG	A
213	283	LEU	A
214	284	TYR	A
215	285	ASP	A
216	286	LEU	A
217	287	ALA	A
218	288	ASN	A
219	289	VAL	A
220	290	PHE	A
221	291	MET	A
222	292	LEU	A
223	293	ALA	A
224	294	TYR	A
225	296	TYR	D
226	297	GLY	D
227	298	TYR	D

Continued on next page

Table S34 – *Continued from previous page*

Homology index	Residue Index	Residue abbreviation	Module
228	299	PRO	A
229	300	LYS	D
230	301	VAL	A
231	302	MET	D
232	303	SER	C
233	304	SER	D
234	305	TYR	A
235	306	ASP	C
236	332	ASN	C
237	333	TRP	D
238	334	LYS	D
239	335	CYS	D
240	338	ARG	A
241	339	TRP	A
242	340	SER	A
243	341	TYR	A
244	342	ILE	A
245	343	ALA	A
246	344	GLY	A
247	345	GLY	A
248	346	VAL	A
249	347	ASP	A
250	348	PHE	A
251	349	ARG	A
252	350	ASN	A
253	351	ASN	A
254	352	THR	A
255	353	ALA	A
256	355	ASN	A
257	356	TRP	A
258	357	ALA	A
259	358	VAL	A
260	360	ASN	A
261	361	TRP	A
262	362	TRP	A
263	363	ASP	A
264	365	THR	A
265	366	ASN	A
266	367	ASN	A
267	368	GLN	A
268	369	ILE	A
269	370	SER	A
270	371	PHE	A
271	372	GLY	A
272	373	ARG	A
273	374	GLY	A

Continued on next page

Table S34 – *Continued from previous page*

Homology index	Residue Index	Residue abbreviation	Module
274	375	SER	A
275	377	GLY	A
276	378	HIS	A
277	379	MET	A
278	380	ALA	A
279	381	ILE	A
280	382	ASN	A
281	385	ASP	A
282	386	SER	A
283	387	THR	A
284	388	LEU	A
285	389	THR	A
286	390	ALA	A
287	391	THR	A
288	397	ALA	A
289	398	SER	A
290	401	TYR	A
291	402	CYS	A
292	403	ASN	A
293	404	VAL	A
294	433	ILE	A
295	434	GLY	A
296	435	ALA	A
297	437	ASP	A
298	438	ALA	A
299	439	MET	A
300	440	ALA	A
301	441	ILE	A
302	442	HIS	A

Table S35: Residues membership for the *B. licheniformis* α-amylase (PDB code 1BLI, chain A)

Homology index	Residue Index	Residue abbreviation	Module
0	5	GLY	A
1	6	THR	A
2	7	LEU	B
3	8	MET	B
4	9	GLN	B
5	10	TYR	C
6	11	PHE	C
7	15	MET	C
8	20	GLN	C

Continued on next page

Table S35 – *Continued from previous page*

Homology index	Residue Index	Residue abbreviation	Module
9	21	HIS	C
10	22	TRP	C
11	23	LYS	C
12	24	ARG	C
13	25	LEU	C
14	26	GLN	C
15	27	ASN	C
16	28	ASP	C
17	29	SER	C
18	30	ALA	C
19	31	TYR	C
20	32	LEU	C
21	33	ALA	C
22	34	GLU	C
23	35	HIS	C
24	36	GLY	C
25	37	ILE	C
26	38	THR	C
27	39	ALA	C
28	40	VAL	C
29	41	TRP	C
30	42	ILE	C
31	43	PRO	C
32	44	PRO	C
33	45	ALA	C
34	46	TYR	C
35	47	LYS	C
36	48	GLY	C
37	49	THR	C
38	52	ALA	C
39	53	ASP	C
40	54	VAL	C
41	55	GLY	C
42	56	TYR	C
43	57	GLY	C
44	58	ALA	C
45	59	TYR	C
46	60	ASP	C
47	62	TYR	C
48	63	ASP	C
49	64	LEU	C
50	65	GLY	C
51	75	THR	C
52	76	LYS	C
53	77	TYR	C
54	78	GLY	C

Continued on next page

Table S35 – *Continued from previous page*

Homology index	Residue Index	Residue abbreviation	Module
55	79	THR	C
56	80	LYS	C
57	81	GLY	C
58	82	GLU	C
59	83	LEU	C
60	84	GLN	C
61	85	SER	C
62	86	ALA	C
63	87	ILE	C
64	88	LYS	C
65	89	SER	C
66	90	LEU	C
67	91	HIS	C
68	92	SER	C
69	93	ARG	C
70	94	ASP	C
71	95	ILE	C
72	96	ASN	C
73	97	VAL	C
74	98	TYR	C
75	99	GLY	C
76	100	ASP	C
77	101	VAL	C
78	102	VAL	C
79	103	ILE	C
80	104	ASN	C
81	105	HIS	C
82	106	LYS	C
83	107	GLY	C
84	108	GLY	C
85	109	ALA	C
86	141	PHE	C
87	154	LYS	C
88	155	TRP	C
89	156	HIS	C
90	157	TRP	C
91	158	TYR	C
92	159	HIS	C
93	160	PHE	C
94	161	ASP	B
95	194	ASP	B
96	195	TYR	B
97	196	LEU	B
98	197	MET	B
99	198	TYR	B
100	199	ALA	B

Continued on next page

Table S35 – *Continued from previous page*

Homology index	Residue Index	Residue abbreviation	Module
101	200	ASP	B
102	201	ILE	B
103	202	ASP	B
104	203	TYR	B
105	204	ASP	B
106	205	HIS	B
107	206	PRO	B
108	207	ASP	B
109	208	VAL	B
110	209	ALA	B
111	210	ALA	B
112	211	GLU	B
113	212	ILE	B
114	213	LYS	B
115	214	ARG	B
116	215	TRP	B
117	216	GLY	B
118	217	THR	B
119	218	TRP	B
120	219	TYR	B
121	220	ALA	B
122	221	ASN	B
123	223	LEU	C
124	224	GLN	C
125	225	LEU	C
126	226	ASP	C
127	227	GLY	C
128	228	PHE	B
129	229	ARG	D
130	230	LEU	B
131	231	ASP	B
132	232	ALA	B
133	233	VAL	B
134	234	LYS	B
135	235	HIS	B
136	236	ILE	B
137	237	LYS	B
138	238	PHE	B
139	239	SER	B
140	240	PHE	B
141	241	LEU	B
142	242	ARG	B
143	243	ASP	B
144	244	TRP	B
145	245	VAL	B
146	246	ASN	B

Continued on next page

Table S35 – *Continued from previous page*

Homology index	Residue Index	Residue abbreviation	Module
147	247	HIS	B
148	248	VAL	B
149	249	ARG	B
150	256	MET	B
151	257	PHE	B
152	258	THR	B
153	259	VAL	C
154	260	ALA	B
155	261	GLU	B
156	262	TYR	B
157	263	TRP	B
158	264	SER	B
159	265	TYR	B
160	270	LEU	B
161	271	GLU	B
162	272	ASN	B
163	273	TYR	B
164	274	LEU	B
165	275	ASN	B
166	280	ASN	B
167	281	HIS	B
168	282	SER	B
169	283	VAL	B
170	284	PHE	D
171	285	ASP	D
172	286	VAL	D
173	287	PRO	D
174	289	HIS	D
175	290	TYR	D
176	291	GLN	D
177	292	PHE	D
178	293	HIS	D
179	294	ALA	D
180	295	ALA	D
181	296	SER	D
182	302	TYR	D
183	303	ASP	A
184	304	MET	A
185	305	ARG	A
186	307	LEU	A
187	308	LEU	D
188	309	ASN	D
189	310	GLY	D
190	313	VAL	D
191	314	SER	B
192	315	LYS	B

Continued on next page

Table S35 – *Continued from previous page*

**Homology index**	**Residue Index**	**Residue abbreviation**	**Module**
193	316	HIS	B
194	317	PRO	B
195	318	LEU	B
196	319	LYS	B
197	320	SER	B
198	321	VAL	D
199	322	THR	D
200	323	PHE	D
201	324	VAL	A
202	325	ASP	D
203	326	ASN	D
204	327	HIS	D
205	328	ASP	D
206	329	THR	D
207	330	GLN	D
208	338	THR	D
209	339	VAL	D
210	340	GLN	D
211	341	THR	D
212	342	TRP	A
213	343	PHE	A
214	344	LYS	A
215	345	PRO	A
216	346	LEU	A
217	347	ALA	A
218	348	TYR	A
219	349	ALA	A
220	350	PHE	A
221	351	ILE	A
222	352	LEU	A
223	353	THR	A
224	354	ARG	A
225	356	SER	D
226	357	GLY	D
227	358	TYR	D
228	359	PRO	A
229	360	GLN	D
230	361	VAL	A
231	362	PHE	D
232	363	TYR	C
233	364	GLY	D
234	365	ASP	A
235	366	MET	C
236	367	TYR	C
237	368	GLY	D
238	369	THR	D

Continued on next page

Table S35 – *Continued from previous page*

**Homology index**	**Residue Index**	**Residue abbreviation**	**Module**
239	379	ALA	D
240	380	LEU	A
241	381	LYS	A
242	382	HIS	A
243	383	LYS	A
244	384	ILE	A
245	385	GLU	A
246	386	PRO	A
247	387	ILE	A
248	388	LEU	A
249	389	LYS	A
250	390	ALA	A
251	391	ARG	A
252	392	LYS	A
253	393	GLN	A
254	394	TYR	A
255	395	ALA	A
256	397	GLY	A
257	398	ALA	A
258	399	GLN	A
259	400	HIS	A
260	401	ASP	A
261	402	TYR	A
262	403	PHE	A
263	404	ASP	A
264	405	HIS	A
265	406	HIS	A
266	407	ASP	A
267	408	ILE	A
268	409	VAL	A
269	410	GLY	A
270	411	TRP	A
271	412	THR	A
272	413	ARG	A
273	414	GLU	A
274	415	GLY	A
275	424	LEU	A
276	425	ALA	A
277	426	ALA	A
278	427	LEU	A
279	428	ILE	A
280	429	THR	A
281	431	GLY	A
282	432	PRO	A
283	433	GLY	A
284	434	GLY	A

Continued on next page

Table S35 – *Continued from previous page*

**Homology index**	**Residue Index**	**Residue abbreviation**	**Module**
285	435	ALA	A
286	436	LYS	A
287	437	ARG	A
288	446	GLY	A
289	447	GLU	A
290	449	TRP	A
291	450	HIS	A
292	451	ASP	A
293	452	ILE	A
294	472	VAL	A
295	473	ASN	A
296	474	GLY	A
297	476	SER	A
298	477	VAL	A
299	478	SER	A
300	479	ILE	A
301	480	TYR	A
302	481	VAL	A

Table S36: Residues membership for the *P. amyloderamosa* α-amylase (PDB code 1BF2, chain A)

**Homology index**	**Residue Index**	**Residue abbreviation**	**Module**
0	179	ASP	A
1	180	VAL	A
2	181	ILE	B
3	182	TYR	B
4	183	GLU	B
5	184	VAL	C
6	185	HIS	C
7	186	VAL	C
8	202	GLY	C
9	203	THR	C
10	204	TYR	C
11	205	TYR	C
12	206	GLY	C
13	207	ALA	C
14	208	GLY	C
15	209	LEU	C
16	210	LYS	C
17	211	ALA	C
18	212	SER	C
19	213	TYR	C

Continued on next page

Table S36 – *Continued from previous page*

Homology index	Residue Index	Residue abbreviation	Module
20	214	LEU	C
21	215	ALA	C
22	216	SER	C
23	217	LEU	C
24	218	GLY	C
25	219	VAL	C
26	220	THR	C
27	221	ALA	C
28	222	VAL	C
29	223	GLU	C
30	224	PHE	C
31	225	LEU	C
32	226	PRO	C
33	227	VAL	C
34	228	GLN	C
35	229	GLU	C
36	230	THR	C
37	231	GLN	C
38	246	ASN	C
39	247	TYR	C
40	248	TRP	C
41	249	GLY	C
42	250	TYR	C
43	251	MET	C
44	252	THR	C
45	253	GLU	C
46	254	ASN	C
47	256	PHE	C
48	257	SER	C
49	258	PRO	C
50	259	ASP	C
51	260	ARG	C
52	261	ARG	C
53	262	TYR	C
54	263	ALA	C
55	264	TYR	C
56	272	THR	C
57	273	ALA	C
58	274	GLU	C
59	275	PHE	C
60	276	GLN	C
61	277	ALA	C
62	278	MET	C
63	279	VAL	C
64	280	GLN	C
65	281	ALA	C

Continued on next page

Table S36 – *Continued from previous page*

Homology index	Residue Index	Residue abbreviation	Module
66	282	PHE	C
67	283	HIS	C
68	284	ASN	C
69	285	ALA	C
70	286	GLY	C
71	287	ILE	C
72	288	LYS	C
73	289	VAL	C
74	290	TYR	C
75	291	MET	C
76	292	ASP	C
77	293	VAL	C
78	294	VAL	C
79	295	TYR	C
80	296	ASN	C
81	297	HIS	C
82	298	THR	C
83	299	ALA	C
84	300	GLU	C
85	301	GLY	C
86	315	TYR	C
87	316	SER	C
88	317	TRP	C
89	318	ARG	C
90	323	ALA	C
91	324	THR	C
92	325	TYR	C
93	326	TYR	C
94	327	GLU	B
95	337	ASP	B
96	338	ASN	B
97	339	THR	B
98	340	GLY	B
99	341	ILE	B
100	343	ALA	B
101	344	ASN	B
102	345	PHE	B
103	346	ASN	B
104	347	THR	B
105	348	TYR	B
106	349	ASN	B
107	350	THR	B
108	351	VAL	B
109	352	ALA	B
110	353	GLN	B
111	354	ASN	B

Continued on next page

Table S36 – *Continued from previous page*

Homology index	Residue Index	Residue abbreviation	Module
112	355	LEU	B
113	356	ILE	B
114	357	VAL	B
115	358	ASP	B
116	359	SER	B
117	360	LEU	B
118	361	ALA	B
119	362	TYR	B
120	363	TRP	B
121	364	ALA	B
122	365	ASN	B
123	367	MET	C
124	368	GLY	C
125	369	VAL	C
126	370	ASP	C
127	371	GLY	C
128	372	PHE	B
129	373	ARG	D
130	374	PHE	B
131	375	ASP	B
132	376	LEU	B
133	377	ALA	B
134	378	SER	B
135	379	VAL	B
136	380	LEU	B
137	381	GLY	B
138	409	ASN	B
139	410	VAL	B
140	411	ALA	B
141	412	ILE	B
142	413	ASN	B
143	414	ARG	B
144	415	ILE	B
145	416	LEU	B
146	417	ARG	B
147	418	GLU	B
148	419	PHE	B
149	420	THR	B
150	430	LEU	B
151	431	ASP	B
152	432	LEU	B
153	433	PHE	C
154	434	ALA	B
155	435	GLU	B
156	436	PRO	B
157	443	SER	B

Continued on next page

Table S36 – *Continued from previous page*

Homology index	Residue Index	Residue abbreviation	Module
158	444	TYR	B
159	445	GLN	B
160	446	LEU	B
161	447	GLY	B
162	448	GLY	B
163	449	PHE	B
164	450	PRO	B
165	451	GLN	B
166	452	GLY	B
167	453	TRP	B
168	454	SER	B
169	455	GLU	B
170	456	TRP	D
171	457	ASN	D
172	458	GLY	D
173	459	LEU	D
174	461	ARG	D
175	462	ASP	D
176	463	SER	D
177	464	LEU	D
178	465	ARG	D
179	466	GLN	D
180	467	ALA	D
181	468	GLN	D
182	475	THR	D
183	476	ILE	A
184	477	TYR	A
185	478	VAL	A
186	480	GLN	A
187	481	ASP	D
188	482	ALA	D
189	483	ASN	D
190	488	SER	D
191	489	SER	B
192	490	ASN	B
193	498	SER	B
194	499	PRO	B
195	500	TRP	B
196	501	ASN	B
197	502	SER	B
198	503	ILE	D
199	504	ASN	D
200	505	PHE	D
201	506	ILE	A
202	507	ASP	D
203	508	VAL	D

Continued on next page

Table S36 – *Continued from previous page*

Homology index	Residue Index	Residue abbreviation	Module
204	509	HIS	D
205	510	ASP	D
206	511	GLY	D
207	512	MET	D
208	513	THR	D
209	514	LEU	D
210	515	LYS	D
211	516	ASP	D
212	559	ARG	A
213	561	ALA	A
214	562	ALA	A
215	563	ARG	A
216	564	THR	A
217	565	GLY	A
218	566	MET	A
219	567	ALA	A
220	568	PHE	A
221	569	GLU	A
222	570	MET	A
223	571	LEU	A
224	572	SER	A
225	573	ALA	D
226	574	GLY	D
227	575	THR	D
228	576	PRO	A
229	577	LEU	D
230	578	MET	A
231	579	GLN	D
232	580	GLY	C
233	581	GLY	D
234	582	ASP	A
235	602	ASN	C
236	603	TRP	C
237	604	LEU	D
238	605	THR	D
239	606	TYR	D
240	615	PHE	A
241	616	TYR	A
242	617	THR	A
243	618	PHE	A
244	619	ALA	A
245	620	GLN	A
246	621	ARG	A
247	622	LEU	A
248	623	ILE	A
249	624	ALA	A

Continued on next page

Table S36 – *Continued from previous page*

Homology index	Residue Index	Residue abbreviation	Module
250	625	PHE	A
251	626	ARG	A
252	627	LYS	A
253	628	ALA	A
254	629	HIS	A
255	630	PRO	A
256	635	SER	A
257	636	SER	A
258	637	TRP	A
259	638	TYR	A
260	645	TRP	A
261	646	TYR	A
262	647	GLN	A
263	648	PRO	A
264	662	SER	A
265	663	ASN	A
266	664	TYR	A
267	665	ALA	A
268	666	ILE	A
269	667	ALA	A
270	668	TYR	A
271	669	ALA	A
272	670	ILE	A
273	671	ASN	A
274	672	GLY	A
275	681	ILE	A
276	682	TYR	A
277	683	VAL	A
278	684	ALA	A
279	685	TYR	A
280	686	ASN	A
281	690	SER	A
282	691	SER	A
283	692	VAL	A
284	693	THR	A
285	694	PHE	A
286	695	THR	A
287	696	LEU	A
288	703	THR	A
289	704	GLN	A
290	706	TYR	A
291	707	ARG	A
292	708	VAL	A
293	709	THR	A
294	739	GLN	A
295	740	CYS	A

Continued on next page

Table S36 – *Continued from previous page*

Homology index	Residue Index	Residue abbreviation	Module
296	741	GLY	A
297	743	SER	A
298	744	LEU	A
299	745	LEU	A
300	746	LEU	A
301	747	LEU	A
302	748	ILE	A

Table S37: Residues membership for the *T. vulgaris R47* α-amylase (PDB code 1BVZ, chain A)

Homology index	Residue Index	Residue abbreviation	Module
0	131	ALA	A
1	132	VAL	A
2	133	ILE	B
3	134	TYR	B
4	135	GLN	B
5	136	ILE	C
6	137	PHE	C
7	138	PRO	C
8	170	GLY	C
9	171	ASP	C
10	172	LEU	C
11	173	LYS	C
12	174	GLY	C
13	175	VAL	C
14	176	ILE	C
15	177	ASP	C
16	178	ARG	C
17	179	LEU	C
18	180	PRO	C
19	181	TYR	C
20	182	LEU	C
21	183	GLU	C
22	184	GLU	C
23	185	LEU	C
24	186	GLY	C
25	187	VAL	C
26	188	THR	C
27	189	ALA	C
28	190	LEU	C
29	191	TYR	C
30	192	PHE	C

Continued on next page

Table S37 – *Continued from previous page*

Homology index	Residue Index	Residue abbreviation	Module
31	193	THR	C
32	194	PRO	C
33	195	ILE	C
34	196	PHE	C
35	197	ALA	C
36	198	SER	C
37	199	PRO	C
38	200	SER	C
39	201	HIS	C
40	202	HIS	C
41	203	LYS	C
42	204	TYR	C
43	205	ASP	C
44	206	THR	C
45	207	ALA	C
46	208	ASP	C
47	210	LEU	C
48	211	ALA	C
49	212	ILE	C
50	213	ASP	C
51	214	PRO	C
52	215	GLN	C
53	216	PHE	C
54	217	GLY	C
55	218	ASP	C
56	219	LEU	C
57	220	PRO	C
58	221	THR	C
59	222	PHE	C
60	223	ARG	C
61	224	ARG	C
62	225	LEU	C
63	226	VAL	C
64	227	ASP	C
65	228	GLU	C
66	229	ALA	C
67	230	HIS	C
68	231	ARG	C
69	232	ARG	C
70	233	GLY	C
71	234	ILE	C
72	235	LYS	C
73	236	ILE	C
74	237	ILE	C
75	238	LEU	C
76	239	ASP	C

Continued on next page

Table S37 – *Continued from previous page*

Homology index	Residue Index	Residue abbreviation	Module
77	240	ALA	C
78	241	VAL	C
79	242	PHE	C
80	243	ASN	C
81	244	HIS	C
82	245	ALA	C
83	246	GLY	C
84	247	ASP	C
85	248	GLN	C
86	249	PHE	C
87	250	PHE	C
88	251	ALA	C
89	252	PHE	C
90	265	LYS	C
91	266	ASP	C
92	267	TRP	C
93	268	PHE	C
94	269	PHE	B
95	284	GLU	B
96	285	THR	B
97	286	PHE	B
98	287	ALA	B
99	293	MET	B
100	294	PRO	B
101	295	LYS	B
102	296	LEU	B
103	297	ARG	B
104	298	THR	B
105	299	GLU	B
106	300	ASN	B
107	301	PRO	B
108	302	GLU	B
109	303	VAL	B
110	304	LYS	B
111	305	GLU	B
112	306	TYR	B
113	307	LEU	B
114	308	PHE	B
115	309	ASP	B
116	310	VAL	B
117	311	ALA	B
118	312	ARG	B
119	313	PHE	B
120	314	TRP	B
121	315	MET	B
122	316	GLU	B

Continued on next page

Table S37 – *Continued from previous page*

Homology index	Residue Index	Residue abbreviation	Module
123	317	GLN	C
124	318	GLY	C
125	319	ILE	C
126	320	ASP	C
127	321	GLY	C
128	322	TRP	B
129	323	ARG	D
130	324	LEU	B
131	325	ASP	B
132	326	VAL	B
133	327	ALA	B
134	328	ASN	B
135	329	GLU	B
136	330	VAL	B
137	331	ASP	B
138	332	HIS	B
139	333	ALA	B
140	334	PHE	B
141	335	TRP	B
142	336	ARG	B
143	337	GLU	B
144	338	PHE	B
145	339	ARG	B
146	340	ARG	B
147	341	LEU	B
148	342	VAL	B
149	343	LYS	B
150	349	ALA	B
151	350	LEU	B
152	351	ILE	B
153	352	VAL	C
154	353	GLY	B
155	354	GLU	B
156	355	ILE	B
157	356	TRP	B
158	357	HIS	B
159	358	ASP	B
160	359	ALA	B
161	360	SER	B
162	361	GLY	B
163	362	TRP	B
164	363	LEU	B
165	364	MET	B
166	368	PHE	B
167	369	ASP	B
168	370	SER	B

Continued on next page

Table S37 – *Continued from previous page*

Homology index	Residue Index	Residue abbreviation	Module
169	371	VAL	B
170	372	MET	D
171	373	ASN	D
172	374	TYR	D
173	375	LEU	D
174	377	ARG	D
175	378	GLU	D
176	379	SER	D
177	380	VAL	D
178	381	ILE	D
179	382	ARG	D
180	383	PHE	D
181	384	PHE	D
182	389	ILE	D
183	390	HIS	A
184	391	ALA	A
185	392	GLU	A
186	397	GLU	A
187	398	LEU	D
188	399	THR	D
189	400	ARG	D
190	401	ALA	D
191	402	ARG	B
192	403	MET	B
193	409	ALA	B
194	410	ALA	B
195	411	GLN	B
196	412	GLY	B
197	413	LEU	B
198	414	TRP	D
199	415	ASN	D
200	416	LEU	D
201	417	LEU	A
202	418	ASP	D
203	419	SER	D
204	420	HIS	D
205	421	ASP	D
206	422	THR	D
207	423	GLU	D
208	424	ARG	D
209	425	PHE	D
210	426	LEU	D
211	427	THR	D
212	433	GLU	A
213	435	LYS	A
214	436	PHE	A

Continued on next page

Table S37 – *Continued from previous page*

**Homology index**	**Residue Index**	**Residue abbreviation**	**Module**
215	437	ARG	A
216	438	LEU	A
217	439	ALA	A
218	440	VAL	A
219	441	LEU	A
220	442	PHE	A
221	443	GLN	A
222	444	MET	A
223	445	THR	A
224	446	TYR	A
225	447	LEU	D
226	448	GLY	D
227	449	THR	D
228	450	PRO	A
229	451	LEU	D
230	452	ILE	A
231	453	TYR	D
232	454	TYR	C
233	455	GLY	D
234	456	ASP	A
235	470	ARG	C
236	471	PRO	C
237	472	MET	D
238	473	ILE	D
239	474	TRP	D
240	483	LEU	A
241	484	PHE	A
242	485	GLU	A
243	486	PHE	A
244	487	TYR	A
245	488	LYS	A
246	489	GLU	A
247	490	LEU	A
248	491	ILE	A
249	492	ARG	A
250	493	LEU	A
251	494	ARG	A
252	495	HIS	A
253	496	ARG	A
254	497	LEU	A
255	498	ALA	A
256	502	ARG	A
257	503	GLY	A
258	504	ASN	A
259	505	VAL	A
260	507	SER	A

Continued on next page

Table S37 – *Continued from previous page*

Homology index	Residue Index	Residue abbreviation	Module
261	508	TRP	A
262	509	HIS	A
263	510	ALA	A
264	513	GLN	A
265	514	ALA	A
266	515	ASN	A
267	516	LEU	A
268	517	TYR	A
269	518	ALA	A
270	519	PHE	A
271	520	VAL	A
272	521	ARG	A
273	526	GLN	A
274	527	HIS	A
275	528	VAL	A
276	529	GLY	A
277	530	VAL	A
278	531	VAL	A
279	532	LEU	A
280	533	ASN	A
281	536	GLY	A
282	537	GLU	A
283	538	LYS	A
284	539	GLN	A
285	540	THR	A
286	541	VAL	A
287	542	LEU	A
288	552	THR	A
289	553	TRP	A
290	554	LEU	A
291	555	ASP	A
292	556	CYS	A
293	557	LEU	A
294	574	ARG	A
295	575	PRO	A
296	576	TYR	A
297	577	GLN	A
298	578	GLY	A
299	579	MET	A
300	580	ILE	A
301	581	LEU	A
302	582	TRP	A

Table S38: Residues membership for the *H. sapiens* α-amylase (PDB code 1BSI, chain A)

**Homology index**	**Residue Index**	**Residue abbreviation**	**Module**
0	11	THR	A
1	12	SER	A
2	13	ILE	B
3	14	VAL	B
4	15	HIS	B
5	16	LEU	C
6	17	PHE	C
7	18	GLU	C
8	19	TRP	C
9	20	ARG	C
10	21	TRP	C
11	22	VAL	C
12	23	ASP	C
13	24	ILE	C
14	25	ALA	C
15	26	LEU	C
16	27	GLU	C
17	28	CYS	C
18	29	GLU	C
19	30	ARG	C
20	32	LEU	C
21	33	ALA	C
22	34	PRO	C
23	35	LYS	C
24	36	GLY	C
25	37	PHE	C
26	38	GLY	C
27	39	GLY	C
28	40	VAL	C
29	41	GLN	C
30	42	VAL	C
31	43	SER	C
32	44	PRO	C
33	45	PRO	C
34	46	ASN	C
35	47	GLU	C
36	48	ASN	C
37	49	VAL	C
38	51	ILE	C
39	59	TRP	C
40	60	GLU	C
41	61	ARG	C
42	62	TYR	C
43	63	GLN	C

Continued on next page

Table S38 – *Continued from previous page*

Homology index	Residue Index	Residue abbreviation	Module
44	64	PRO	C
45	65	VAL	C
46	66	SER	C
47	67	TYR	C
48	68	LYS	C
49	69	LEU	C
50	70	CYS	C
51	71	THR	C
52	72	ARG	C
53	73	SER	C
54	74	GLY	C
55	75	ASN	C
56	76	GLU	C
57	77	ASP	C
58	78	GLU	C
59	79	PHE	C
60	80	ARG	C
61	81	ASN	C
62	82	MET	C
63	83	VAL	C
64	84	THR	C
65	85	ARG	C
66	86	CYS	C
67	87	ASN	C
68	88	ASN	C
69	89	VAL	C
70	90	GLY	C
71	91	VAL	C
72	92	ARG	C
73	93	ILE	C
74	94	TYR	C
75	95	VAL	C
76	96	ASP	C
77	97	ALA	C
78	98	VAL	C
79	99	ILE	C
80	100	ASN	C
81	101	HIS	C
82	102	MET	C
83	103	CYS	C
84	104	GLY	C
85	105	ASN	C
86	126	PHE	C
87	130	PRO	C
88	131	TYR	C
89	132	SER	C

Continued on next page

Table S38 – *Continued from previous page*

Homology index	Residue Index	Residue abbreviation	Module
90	133	GLY	C
91	134	TRP	C
92	135	ASP	C
93	136	PHE	C
94	137	ASN	B
95	160	CYS	B
96	161	ARG	B
97	162	LEU	B
98	163	THR	B
99	165	LEU	B
100	166	LEU	B
101	167	ASP	B
102	168	LEU	B
103	169	ALA	B
104	170	LEU	B
105	171	GLU	B
106	172	LYS	B
107	173	ASP	B
108	174	TYR	B
109	175	VAL	B
110	176	ARG	B
111	177	SER	B
112	178	LYS	B
113	179	ILE	B
114	180	ALA	B
115	181	GLU	B
116	182	TYR	B
117	183	MET	B
118	184	ASN	B
119	185	HIS	B
120	186	LEU	B
121	187	ILE	B
122	188	ASP	B
123	189	ILE	C
124	190	GLY	C
125	191	VAL	C
126	192	ALA	C
127	193	GLY	C
128	194	PHE	B
129	195	ARG	D
130	196	LEU	B
131	197	ASP	B
132	198	ALA	B
133	199	SER	B
134	200	LYS	B
135	201	HIS	B

Continued on next page

Table S38 – *Continued from previous page*

Homology index	Residue Index	Residue abbreviation	Module
136	202	MET	B
137	203	TRP	B
138	204	PRO	B
139	205	GLY	B
140	206	ASP	B
141	207	ILE	B
142	208	LYS	B
143	209	ALA	B
144	210	ILE	B
145	211	LEU	B
146	212	ASP	B
147	213	LYS	B
148	214	LEU	B
149	215	HIS	B
150	228	PRO	B
151	229	PHE	B
152	230	ILE	B
153	231	TYR	C
154	232	GLN	B
155	233	GLU	B
156	234	VAL	B
157	235	ILE	B
158	236	ASP	B
159	237	LEU	B
160	244	SER	B
161	245	SER	B
162	246	ASP	B
163	247	TYR	B
164	248	PHE	B
165	249	GLY	B
166	250	ASN	B
167	251	GLY	B
168	252	ARG	B
169	253	VAL	B
170	254	THR	D
171	255	GLU	D
172	256	PHE	D
173	257	LYS	D
174	259	GLY	D
175	260	ALA	D
176	261	LYS	D
177	262	LEU	D
178	263	GLY	D
179	264	THR	D
180	265	VAL	D
181	266	ILE	D

Continued on next page

Table S38 – *Continued from previous page*

Homology index	Residue Index	Residue abbreviation	Module
182	272	GLU	D
183	273	LYS	A
184	274	MET	A
185	275	SER	A
186	280	TRP	A
187	281	GLY	D
188	282	GLU	D
189	283	GLY	D
190	285	GLY	D
191	286	PHE	B
192	287	VAL	B
193	288	PRO	B
194	289	SER	B
195	290	ASP	B
196	291	ARG	B
197	292	ALA	B
198	293	LEU	D
199	294	VAL	D
200	295	PHE	D
201	296	VAL	A
202	297	ASP	D
203	298	ASN	D
204	299	HIS	D
205	300	ASP	D
206	301	ASN	D
207	302	GLN	D
208	314	THR	D
209	315	PHE	D
210	316	TRP	D
211	317	ASP	D
212	319	ARG	A
213	320	LEU	A
214	321	TYR	A
215	322	LYS	A
216	323	MET	A
217	324	ALA	A
218	325	VAL	A
219	326	GLY	A
220	327	PHE	A
221	328	MET	A
222	329	LEU	A
223	330	ALA	A
224	331	HIS	A
225	333	TYR	D
226	334	GLY	D
227	335	PHE	D

Continued on next page

Table S38 – *Continued from previous page*

Homology index	Residue Index	Residue abbreviation	Module
228	336	THR	A
229	337	ARG	D
230	338	VAL	A
231	339	MET	D
232	340	SER	C
233	341	SER	D
234	342	TYR	A
235	343	ARG	C
236	381	ASP	C
237	382	TRP	D
238	383	VAL	D
239	384	CYS	D
240	387	ARG	A
241	388	TRP	A
242	389	ARG	A
243	390	GLN	A
244	391	ILE	A
245	392	ARG	A
246	393	ASN	A
247	394	MET	A
248	395	VAL	A
249	396	ILE	A
250	397	PHE	A
251	398	ARG	A
252	399	ASN	A
253	400	VAL	A
254	401	VAL	A
255	402	ASP	A
256	403	GLY	A
257	404	GLN	A
258	405	PRO	A
259	406	PHE	A
260	408	ASN	A
261	409	TRP	A
262	410	TYR	A
263	411	ASP	A
264	413	GLY	A
265	414	SER	A
266	415	ASN	A
267	416	GLN	A
268	417	VAL	A
269	418	ALA	A
270	419	PHE	A
271	420	GLY	A
272	421	ARG	A
273	422	GLY	A

Continued on next page

Table S38 – *Continued from previous page*

Homology index	Residue Index	Residue abbreviation	Module
274	423	ASN	A
275	425	GLY	A
276	426	PHE	A
277	427	ILE	A
278	428	VAL	A
279	429	PHE	A
280	430	ASN	A
281	433	ASP	A
282	434	TRP	A
283	435	SER	A
284	436	PHE	A
285	437	SER	A
286	438	LEU	A
287	439	THR	A
288	445	PRO	A
289	446	ALA	A
290	449	TYR	A
291	450	CYS	A
292	451	ASP	A
293	452	VAL	A
294	479	ILE	A
295	480	SER	A
296	481	ASN	A
297	486	PRO	A
298	487	PHE	A
299	488	ILE	A
300	489	ALA	A
301	490	ILE	A
302	491	HIS	A

Table S39: Residues membership for the *B. circulans,s8* α-amylase (PDB code 1CGT, chain A)

Homology index	Residue Index	Residue abbreviation	Module
0	15	ASP	A
1	16	VAL	A
2	17	ILE	B
3	18	TYR	B
4	19	GLN	B
5	20	VAL	C
6	21	PHE	C
7	22	THR	C
8	52	GLY	C

Continued on next page

Table S39 – *Continued from previous page*

Homology index	Residue Index	Residue abbreviation	Module
9	53	ASP	C
10	54	TRP	C
11	55	GLN	C
12	56	GLY	C
13	57	LEU	C
14	58	ILE	C
15	59	ASN	C
16	60	LYS	C
17	61	ILE	C
18	62	ASN	C
19	63	ASP	C
20	66	PHE	C
21	67	SER	C
22	68	ASP	C
23	69	LEU	C
24	70	GLY	C
25	71	VAL	C
26	72	THR	C
27	73	ALA	C
28	74	LEU	C
29	75	TRP	C
30	76	ILE	C
31	77	SER	C
32	78	GLN	C
33	79	PRO	C
34	80	VAL	C
35	81	GLU	C
36	82	ASN	C
37	83	ILE	C
38	95	THR	C
39	97	TYR	C
40	98	HIS	C
41	99	GLY	C
42	100	TYR	C
43	101	TRP	C
44	102	ALA	C
45	103	ARG	C
46	104	ASP	C
47	106	LYS	C
48	107	LYS	C
49	108	THR	C
50	109	ASN	C
51	110	PRO	C
52	111	TYR	C
53	112	PHE	C
54	113	GLY	C

Continued on next page

Table S39 – *Continued from previous page*

Homology index	Residue Index	Residue abbreviation	Module
55	114	THR	C
56	115	MET	C
57	116	ALA	C
58	117	ASP	C
59	118	PHE	C
60	119	GLN	C
61	120	ASN	C
62	121	LEU	C
63	122	ILE	C
64	123	THR	C
65	124	THR	C
66	125	ALA	C
67	126	HIS	C
68	127	ALA	C
69	128	LYS	C
70	129	GLY	C
71	130	ILE	C
72	131	LYS	C
73	132	ILE	C
74	133	VAL	C
75	134	ILE	C
76	135	ASP	C
77	136	PHE	C
78	137	ALA	C
79	138	PRO	C
80	139	ASN	C
81	140	HIS	C
82	141	THR	C
83	142	SER	C
84	143	PRO	C
85	144	ALA	C
86	156	ARG	C
87	163	LEU	C
88	164	VAL	C
89	165	GLY	C
90	172	ASN	C
91	173	GLY	C
92	174	TYR	C
93	175	PHE	C
94	176	HIS	B
95	192	LYS	B
96	193	ASN	B
97	194	LEU	B
98	195	TYR	B
99	197	LEU	B
100	198	ALA	B

Continued on next page

Table S39 – *Continued from previous page*

Homology index	Residue Index	Residue abbreviation	Module
101	199	ASP	B
102	200	PHE	B
103	201	ASN	B
104	202	HIS	B
105	203	ASN	B
106	204	ASN	B
107	205	ALA	B
108	206	THR	B
109	207	ILE	B
110	208	ASP	B
111	209	LYS	B
112	210	TYR	B
113	211	PHE	B
114	212	LYS	B
115	213	ASP	B
116	214	ALA	B
117	215	ILE	B
118	216	LYS	B
119	217	LEU	B
120	218	TRP	B
121	219	LEU	B
122	220	ASP	B
123	221	MET	C
124	222	GLY	C
125	223	VAL	C
126	224	ASP	C
127	225	GLY	C
128	226	ILE	B
129	227	ARG	D
130	228	VAL	B
131	229	ASP	B
132	230	ALA	B
133	231	VAL	B
134	232	LYS	B
135	233	HIS	B
136	234	MET	B
137	235	PRO	B
138	236	LEU	B
139	237	GLY	B
140	238	TRP	B
141	239	GLN	B
142	240	LYS	B
143	241	SER	B
144	242	TRP	B
145	243	MET	B
146	244	SER	B

Continued on next page

Table S39 – *Continued from previous page*

Homology index	Residue Index	Residue abbreviation	Module
147	245	SER	B
148	246	ILE	B
149	247	TYR	B
150	252	VAL	B
151	253	PHE	B
152	254	THR	B
153	255	PHE	C
154	256	GLY	B
155	257	GLU	B
156	258	TRP	B
157	259	PHE	B
158	260	LEU	B
159	261	GLY	B
160	269	ASN	B
161	270	THR	B
162	271	ASP	B
163	272	PHE	B
164	274	ASN	B
165	275	LYS	B
166	277	GLY	B
167	278	MET	B
168	279	SER	B
169	280	LEU	B
170	281	LEU	D
171	282	ASP	D
172	283	PHE	D
173	284	ARG	D
174	286	ASN	D
175	287	SER	D
176	288	ALA	D
177	289	VAL	D
178	290	ARG	D
179	291	ASN	D
180	292	VAL	D
181	293	PHE	D
182	298	SER	D
183	299	ASN	A
184	300	MET	A
185	301	TYR	A
186	306	MET	A
187	307	ILE	D
188	308	ASN	D
189	309	SER	D
190	310	THR	D
191	311	ALA	B
192	312	THR	B

Continued on next page

Table S39 – *Continued from previous page*

Homology index	Residue Index	Residue abbreviation	Module
193	316	GLN	B
194	317	VAL	B
195	318	ASN	B
196	319	ASP	B
197	320	GLN	B
198	321	VAL	D
199	322	THR	D
200	323	PHE	D
201	324	ILE	A
202	325	ASP	D
203	326	ASN	D
204	327	HIS	D
205	328	ASP	D
206	329	MET	D
207	330	ASP	D
208	331	ARG	D
209	332	PHE	D
210	333	LYS	D
211	334	THR	D
212	339	ASN	A
213	341	ARG	A
214	342	LEU	A
215	343	GLU	A
216	344	GLN	A
217	345	ALA	A
218	346	LEU	A
219	347	ALA	A
220	348	PHE	A
221	349	THR	A
222	350	LEU	A
223	351	THR	A
224	352	SER	A
225	353	ARG	D
226	354	GLY	D
227	355	VAL	D
228	356	PRO	A
229	357	ALA	D
230	358	ILE	A
231	359	TYR	D
232	360	TYR	C
233	361	GLY	D
234	362	THR	A
235	376	ALA	C
236	377	LYS	C
237	378	MET	D
238	379	PRO	D

Continued on next page

Table S39 – *Continued from previous page*

Homology index	Residue Index	Residue abbreviation	Module
239	381	PHE	D
240	387	ALA	A
241	388	PHE	A
242	389	ASN	A
243	390	VAL	A
244	391	ILE	A
245	392	SER	A
246	393	LYS	A
247	394	LEU	A
248	395	ALA	A
249	396	PRO	A
250	397	LEU	A
251	398	ARG	A
252	399	LYS	A
253	400	SER	A
254	401	ASN	A
255	402	PRO	A
256	406	TYR	A
257	407	GLY	A
258	408	SER	A
259	409	THR	A
260	411	GLN	A
261	412	ARG	A
262	413	TRP	A
263	414	ILE	A
264	415	ASN	A
265	416	ASN	A
266	417	ASP	A
267	418	VAL	A
268	419	TYR	A
269	420	VAL	A
270	421	TYR	A
271	422	GLU	A
272	423	ARG	A
273	428	SER	A
274	429	VAL	A
275	430	ALA	A
276	431	VAL	A
277	432	VAL	A
278	433	ALA	A
279	434	VAL	A
280	435	ASN	A
281	439	SER	A
282	440	THR	A
283	441	SER	A
284	442	ALA	A

Continued on next page

Table S39 – *Continued from previous page*

Homology index	Residue Index	Residue abbreviation	Module
285	443	SER	A
286	444	ILE	A
287	445	THR	A
288	454	GLY	A
289	455	SER	A
290	457	THR	A
291	458	ASP	A
292	459	VAL	A
293	460	LEU	A
294	482	ALA	A
295	483	ALA	A
296	484	GLY	A
297	485	ALA	A
298	486	THR	A
299	487	ALA	A
300	488	VAL	A
301	489	TRP	A
302	490	GLN	A

Table S40: Residues membership for the *B. circulans* α-amylase (PDB code 1CDG, chain A)

Homology index	Residue Index	Residue abbreviation	Module
0	15	ASP	A
1	16	VAL	A
2	17	ILE	B
3	18	TYR	B
4	19	GLN	B
5	20	ILE	C
6	21	PHE	C
7	22	THR	C
8	52	GLY	C
9	53	ASP	C
10	54	TRP	C
11	55	GLN	C
12	56	GLY	C
13	57	ILE	C
14	58	ILE	C
15	59	ASN	C
16	60	LYS	C
17	61	ILE	C
18	62	ASN	C
19	63	ASP	C

Continued on next page

Table S40 – *Continued from previous page*

Homology index	Residue Index	Residue abbreviation	Module
20	66	LEU	C
21	67	THR	C
22	68	GLY	C
23	69	MET	C
24	70	GLY	C
25	71	VAL	C
26	72	THR	C
27	73	ALA	C
28	74	ILE	C
29	75	TRP	C
30	76	ILE	C
31	77	SER	C
32	78	GLN	C
33	79	PRO	C
34	80	VAL	C
35	81	GLU	C
36	82	ASN	C
37	83	ILE	C
38	95	THR	C
39	97	TYR	C
40	98	HIS	C
41	99	GLY	C
42	100	TYR	C
43	101	TRP	C
44	102	ALA	C
45	103	ARG	C
46	104	ASP	C
47	106	LYS	C
48	107	LYS	C
49	108	THR	C
50	109	ASN	C
51	110	PRO	C
52	111	ALA	C
53	112	TYR	C
54	113	GLY	C
55	114	THR	C
56	115	ILE	C
57	116	ALA	C
58	117	ASP	C
59	118	PHE	C
60	119	GLN	C
61	120	ASN	C
62	121	LEU	C
63	122	ILE	C
64	123	ALA	C
65	124	ALA	C

Continued on next page

Table S40 – *Continued from previous page*

Homology index	Residue Index	Residue abbreviation	Module
66	125	ALA	C
67	126	HIS	C
68	127	ALA	C
69	128	LYS	C
70	129	ASN	C
71	130	ILE	C
72	131	LYS	C
73	132	VAL	C
74	133	ILE	C
75	134	ILE	C
76	135	ASP	C
77	136	PHE	C
78	137	ALA	C
79	138	PRO	C
80	139	ASN	C
81	140	HIS	C
82	141	THR	C
83	142	SER	C
84	143	PRO	C
85	144	ALA	C
86	156	ARG	C
87	163	LEU	C
88	164	LEU	C
89	165	GLY	C
90	172	GLN	C
91	173	ASN	C
92	174	LEU	C
93	175	PHE	C
94	176	HIS	B
95	192	LYS	B
96	193	ASN	B
97	194	LEU	B
98	195	TYR	B
99	197	LEU	B
100	198	ALA	B
101	199	ASP	B
102	200	LEU	B
103	201	ASN	B
104	202	HIS	B
105	203	ASN	B
106	204	ASN	B
107	205	SER	B
108	206	THR	B
109	207	VAL	B
110	208	ASP	B
111	209	VAL	B

Continued on next page

Table S40 – *Continued from previous page*

Homology index	Residue Index	Residue abbreviation	Module
112	210	TYR	B
113	211	LEU	B
114	212	LYS	B
115	213	ASP	B
116	214	ALA	B
117	215	ILE	B
118	216	LYS	B
119	217	MET	B
120	218	TRP	B
121	219	LEU	B
122	220	ASP	B
123	221	LEU	C
124	222	GLY	C
125	223	ILE	C
126	224	ASP	C
127	225	GLY	C
128	226	ILE	B
129	227	ARG	D
130	228	MET	B
131	229	ASP	B
132	230	ALA	B
133	231	VAL	B
134	232	LYS	B
135	233	HIS	B
136	234	MET	B
137	235	PRO	B
138	236	PHE	B
139	237	GLY	B
140	238	TRP	B
141	239	GLN	B
142	240	LYS	B
143	241	SER	B
144	242	PHE	B
145	243	MET	B
146	244	ALA	B
147	245	ALA	B
148	246	VAL	B
149	247	ASN	B
150	252	VAL	B
151	253	PHE	B
152	254	THR	B
153	255	PHE	C
154	256	GLY	B
155	257	GLU	B
156	258	TRP	B
157	259	PHE	B

Continued on next page

Table S40 – *Continued from previous page*

Homology index	Residue Index	Residue abbreviation	Module
158	260	LEU	B
159	261	GLY	B
160	269	ASN	B
161	270	HIS	B
162	271	LYS	B
163	272	PHE	B
164	274	ASN	B
165	275	GLU	B
166	277	GLY	B
167	278	MET	B
168	279	SER	B
169	280	LEU	B
170	281	LEU	D
171	282	ASP	D
172	283	PHE	D
173	284	ARG	D
174	286	ALA	D
175	287	GLN	D
176	288	LYS	D
177	289	VAL	D
178	290	ARG	D
179	291	GLN	D
180	292	VAL	D
181	293	PHE	D
182	298	ASP	D
183	299	ASN	A
184	300	MET	A
185	301	TYR	A
186	306	MET	A
187	307	LEU	D
188	308	GLU	D
189	309	GLY	D
190	310	SER	D
191	311	ALA	B
192	312	ALA	B
193	316	GLN	B
194	317	VAL	B
195	318	ASP	B
196	319	ASP	B
197	320	GLN	B
198	321	VAL	D
199	322	THR	D
200	323	PHE	D
201	324	ILE	A
202	325	ASP	D
203	326	ASN	D

Continued on next page

Table S40 – *Continued from previous page*

Homology index	Residue Index	Residue abbreviation	Module
204	327	HIS	D
205	328	ASP	D
206	329	MET	D
207	330	GLU	D
208	331	ARG	D
209	332	PHE	D
210	333	HIS	D
211	334	ALA	D
212	339	ARG	A
213	341	LYS	A
214	342	LEU	A
215	343	GLU	A
216	344	GLN	A
217	345	ALA	A
218	346	LEU	A
219	347	ALA	A
220	348	PHE	A
221	349	THR	A
222	350	LEU	A
223	351	THR	A
224	352	SER	A
225	353	ARG	D
226	354	GLY	D
227	355	VAL	D
228	356	PRO	A
229	357	ALA	D
230	358	ILE	A
231	359	TYR	D
232	360	TYR	C
233	361	GLY	D
234	362	THR	A
235	376	ALA	C
236	377	ARG	C
237	378	ILE	D
238	379	PRO	D
239	381	PHE	D
240	387	ALA	A
241	388	TYR	A
242	389	GLN	A
243	390	VAL	A
244	391	ILE	A
245	392	GLN	A
246	393	LYS	A
247	394	LEU	A
248	395	ALA	A
249	396	PRO	A

Continued on next page

Table S40 – *Continued from previous page*

Homology index	Residue Index	Residue abbreviation	Module
250	397	LEU	A
251	398	ARG	A
252	399	LYS	A
253	400	CYS	A
254	401	ASN	A
255	402	PRO	A
256	406	TYR	A
257	407	GLY	A
258	408	SER	A
259	409	THR	A
260	411	GLU	A
261	412	ARG	A
262	413	TRP	A
263	414	ILE	A
264	415	ASN	A
265	416	ASN	A
266	417	ASP	A
267	418	VAL	A
268	419	LEU	A
269	420	ILE	A
270	421	TYR	A
271	422	GLU	A
272	423	ARG	A
273	428	ASN	A
274	429	VAL	A
275	430	ALA	A
276	431	VAL	A
277	432	VAL	A
278	433	ALA	A
279	434	VAL	A
280	435	ASN	A
281	439	ASN	A
282	440	ALA	A
283	441	PRO	A
284	442	ALA	A
285	443	SER	A
286	444	ILE	A
287	445	SER	A
288	454	GLY	A
289	455	SER	A
290	457	ASN	A
291	458	ASP	A
292	459	VAL	A
293	460	LEU	A
294	483	ALA	A
295	484	ALA	A

Continued on next page

Table S40 – *Continued from previous page*

Homology index	Residue Index	Residue abbreviation	Module
296	485	GLY	A
297	486	GLY	A
298	487	THR	A
299	488	ALA	A
300	489	VAL	A
301	490	TRP	A
302	491	GLN	A

Table S41: Residues membership for the *T. thermosulfurigenes EM1* α-amylase (PDB code 1CIU, chain A)

Homology index	Residue Index	Residue abbreviation	Module
0	15	ASP	A
1	16	VAL	A
2	17	ILE	B
3	18	TYR	B
4	19	GLN	B
5	20	ILE	C
6	21	VAL	C
7	22	THR	C
8	52	GLY	C
9	53	ASP	C
10	54	TRP	C
11	55	GLN	C
12	56	GLY	C
13	57	ILE	C
14	58	ILE	C
15	59	ASN	C
16	60	LYS	C
17	61	ILE	C
18	62	ASN	C
19	63	ASP	C
20	66	LEU	C
21	67	THR	C
22	68	GLY	C
23	69	MET	C
24	70	GLY	C
25	71	VAL	C
26	72	THR	C
27	73	ALA	C
28	74	ILE	C
29	75	TRP	C
30	76	ILE	C

Continued on next page

Table S41 – *Continued from previous page*

Homology index	Residue Index	Residue abbreviation	Module
31	77	SER	C
32	78	GLN	C
33	79	PRO	C
34	80	VAL	C
35	81	GLU	C
36	82	ASN	C
37	83	ILE	C
38	96	THR	C
39	98	TYR	C
40	99	HIS	C
41	100	GLY	C
42	101	TYR	C
43	102	TRP	C
44	103	ALA	C
45	104	ARG	C
46	105	ASP	C
47	107	LYS	C
48	108	ARG	C
49	109	THR	C
50	110	ASN	C
51	111	PRO	C
52	112	TYR	C
53	113	PHE	C
54	114	GLY	C
55	115	SER	C
56	116	PHE	C
57	117	THR	C
58	118	ASP	C
59	119	PHE	C
60	120	GLN	C
61	121	ASN	C
62	122	LEU	C
63	123	ILE	C
64	124	ASN	C
65	125	THR	C
66	126	ALA	C
67	127	HIS	C
68	128	ALA	C
69	129	HIS	C
70	130	ASN	C
71	131	ILE	C
72	132	LYS	C
73	133	VAL	C
74	134	ILE	C
75	135	ILE	C
76	136	ASP	C

Continued on next page

Table S41 – *Continued from previous page*

Homology index	Residue Index	Residue abbreviation	Module
77	137	PHE	C
78	138	ALA	C
79	139	PRO	C
80	140	ASN	C
81	141	HIS	C
82	142	THR	C
83	143	SER	C
84	144	PRO	C
85	145	ALA	C
86	157	ARG	C
87	164	LEU	C
88	165	LEU	C
89	166	GLY	C
90	173	ASN	C
91	174	GLY	C
92	175	TYR	C
93	176	PHE	C
94	177	HIS	B
95	193	ARG	B
96	194	ASN	B
97	195	LEU	B
98	196	PHE	B
99	198	LEU	B
100	199	ALA	B
101	200	ASP	B
102	201	LEU	B
103	202	ASN	B
104	203	GLN	B
105	204	GLN	B
106	205	ASN	B
107	206	SER	B
108	207	THR	B
109	208	ILE	B
110	209	ASP	B
111	210	SER	B
112	211	TYR	B
113	212	LEU	B
114	213	LYS	B
115	214	SER	B
116	215	ALA	B
117	216	ILE	B
118	217	LYS	B
119	218	VAL	B
120	219	TRP	B
121	220	LEU	B
122	221	ASP	B

Continued on next page

Table S41 – *Continued from previous page*

Homology index	Residue Index	Residue abbreviation	Module
123	222	MET	C
124	223	GLY	C
125	224	ILE	C
126	225	ASP	C
127	226	GLY	C
128	227	ILE	B
129	228	ARG	D
130	229	LEU	B
131	230	ASP	B
132	231	ALA	B
133	232	VAL	B
134	233	LYS	B
135	234	HIS	B
136	235	MET	B
137	236	PRO	B
138	237	PHE	B
139	238	GLY	B
140	239	TRP	B
141	240	GLN	B
142	241	LYS	B
143	242	ASN	B
144	243	PHE	B
145	244	MET	B
146	245	ASP	B
147	246	SER	B
148	247	ILE	B
149	248	LEU	B
150	253	VAL	B
151	254	PHE	B
152	255	THR	B
153	256	PHE	C
154	257	GLY	B
155	258	GLU	B
156	259	TRP	B
157	260	PHE	B
158	261	LEU	B
159	262	GLY	B
160	270	ASN	B
161	271	THR	B
162	272	TYR	B
163	273	PHE	B
164	275	ASN	B
165	276	GLU	B
166	278	GLY	B
167	279	MET	B
168	280	SER	B

Continued on next page

Table S41 – *Continued from previous page*

Homology index	Residue Index	Residue abbreviation	Module
169	281	LEU	B
170	282	LEU	D
171	283	ASP	D
172	284	PHE	D
173	285	ARG	D
174	287	SER	D
175	288	GLN	D
176	289	LYS	D
177	290	VAL	D
178	291	ARG	D
179	292	GLN	D
180	293	VAL	D
181	294	PHE	D
182	299	ASP	D
183	300	THR	A
184	301	MET	A
185	302	TYR	A
186	307	MET	A
187	308	ILE	D
188	309	GLN	D
189	310	SER	D
190	311	THR	D
191	312	ALA	B
192	313	SER	B
193	317	PHE	B
194	318	ILE	B
195	319	ASN	B
196	320	ASP	B
197	321	MET	B
198	322	VAL	D
199	323	THR	D
200	324	PHE	D
201	325	ILE	A
202	326	ASP	D
203	327	ASN	D
204	328	HIS	D
205	329	ASP	D
206	330	MET	D
207	331	ASP	D
208	332	ARG	D
209	333	PHE	D
210	334	TYR	D
211	335	ASN	D
212	339	THR	A
213	341	PRO	A
214	342	VAL	A

Continued on next page

Table S41 – *Continued from previous page*

Homology index	Residue Index	Residue abbreviation	Module
215	343	GLU	A
216	344	GLN	A
217	345	ALA	A
218	346	LEU	A
219	347	ALA	A
220	348	PHE	A
221	349	THR	A
222	350	LEU	A
223	351	THR	A
224	352	SER	A
225	353	ARG	D
226	354	GLY	D
227	355	VAL	D
228	356	PRO	A
229	357	ALA	D
230	358	ILE	A
231	359	TYR	D
232	360	TYR	C
233	361	GLY	D
234	362	THR	A
235	376	ALA	C
236	377	MET	C
237	378	MET	D
238	379	THR	D
239	381	PHE	D
240	387	ALA	A
241	388	TYR	A
242	389	ASN	A
243	390	VAL	A
244	391	ILE	A
245	392	LYS	A
246	393	LYS	A
247	394	LEU	A
248	395	ALA	A
249	396	PRO	A
250	397	LEU	A
251	398	ARG	A
252	399	LYS	A
253	400	SER	A
254	401	ASN	A
255	402	PRO	A
256	406	TYR	A
257	407	GLY	A
258	408	THR	A
259	409	THR	A
260	411	GLN	A

Continued on next page

Table S41 – *Continued from previous page*

Homology index	Residue Index	Residue abbreviation	Module
261	412	ARG	A
262	413	TRP	A
263	414	ILE	A
264	415	ASN	A
265	416	ASN	A
266	417	ASP	A
267	418	VAL	A
268	419	TYR	A
269	420	ILE	A
270	421	TYR	A
271	422	GLU	A
272	423	ARG	A
273	428	ASN	A
274	429	VAL	A
275	430	ALA	A
276	431	LEU	A
277	432	VAL	A
278	433	ALA	A
279	434	ILE	A
280	435	ASN	A
281	439	SER	A
282	440	THR	A
283	441	SER	A
284	442	TYR	A
285	443	ASN	A
286	444	ILE	A
287	445	THR	A
288	454	GLY	A
289	455	THR	A
290	457	THR	A
291	458	ASP	A
292	459	VAL	A
293	460	LEU	A
294	483	SER	A
295	484	ALA	A
296	485	GLY	A
297	486	GLU	A
298	487	VAL	A
299	488	ALA	A
300	489	VAL	A
301	490	TRP	A
302	491	GLN	A

Table S42: Residues membership for the *B. circulans,s8* α-amylase (PDB code 1CGU, chain A)

Homology index	Residue Index	Residue abbreviation	Module
0	15	ASP	A
1	16	VAL	A
2	17	ILE	B
3	18	TYR	B
4	19	GLN	B
5	20	VAL	C
6	21	PHE	C
7	22	THR	C
8	52	GLY	C
9	53	ASP	C
10	54	TRP	C
11	55	GLN	C
12	56	GLY	C
13	57	LEU	C
14	58	ILE	C
15	59	ASN	C
16	60	LYS	C
17	61	ILE	C
18	62	ASN	C
19	63	ASP	C
20	66	PHE	C
21	67	SER	C
22	68	ASP	C
23	69	LEU	C
24	70	GLY	C
25	71	VAL	C
26	72	THR	C
27	73	ALA	C
28	74	LEU	C
29	75	TRP	C
30	76	ILE	C
31	77	SER	C
32	78	GLN	C
33	79	PRO	C
34	80	VAL	C
35	81	GLU	C
36	82	ASN	C
37	83	ILE	C
38	95	THR	C
39	97	TYR	C
40	98	HIS	C
41	99	GLY	C
42	100	TYR	C
43	101	TRP	C

Continued on next page

Table S42 – *Continued from previous page*

Homology index	Residue Index	Residue abbreviation	Module
44	102	ALA	C
45	103	ARG	C
46	104	ASP	C
47	106	LYS	C
48	107	LYS	C
49	108	THR	C
50	109	ASN	C
51	110	PRO	C
52	111	TYR	C
53	112	PHE	C
54	113	GLY	C
55	114	THR	C
56	115	MET	C
57	116	ALA	C
58	117	ASP	C
59	118	PHE	C
60	119	GLN	C
61	120	ASN	C
62	121	LEU	C
63	122	ILE	C
64	123	THR	C
65	124	THR	C
66	125	ALA	C
67	126	HIS	C
68	127	ALA	C
69	128	LYS	C
70	129	GLY	C
71	130	ILE	C
72	131	LYS	C
73	132	ILE	C
74	133	VAL	C
75	134	ILE	C
76	135	ASP	C
77	136	PHE	C
78	137	ALA	C
79	138	PRO	C
80	139	ASN	C
81	140	HIS	C
82	141	THR	C
83	142	SER	C
84	143	PRO	C
85	144	ALA	C
86	156	ARG	C
87	163	LEU	C
88	164	VAL	C
89	165	GLY	C

Continued on next page

Table S42 – *Continued from previous page*

Homology index	Residue Index	Residue abbreviation	Module
90	172	ASN	C
91	173	GLY	C
92	174	TYR	C
93	175	PHE	C
94	176	HIS	B
95	192	LYS	B
96	193	ASN	B
97	194	LEU	B
98	195	TYR	B
99	197	LEU	B
100	198	ALA	B
101	199	ASP	B
102	200	PHE	B
103	201	ASN	B
104	202	HIS	B
105	203	ASN	B
106	204	ASN	B
107	205	ALA	B
108	206	THR	B
109	207	ILE	B
110	208	ASP	B
111	209	LYS	B
112	210	TYR	B
113	211	PHE	B
114	212	LYS	B
115	213	ASP	B
116	214	ALA	B
117	215	ILE	B
118	216	LYS	B
119	217	LEU	B
120	218	TRP	B
121	219	LEU	B
122	220	ASP	B
123	221	MET	C
124	222	GLY	C
125	223	VAL	C
126	224	ASP	C
127	225	GLY	C
128	226	ILE	B
129	227	ARG	D
130	228	VAL	B
131	229	ALA	B
132	230	ALA	B
133	231	VAL	B
134	232	LYS	B
135	233	HIS	B

Continued on next page

Table S42 – *Continued from previous page*

Homology index	Residue Index	Residue abbreviation	Module
136	234	MET	B
137	235	PRO	B
138	236	LEU	B
139	237	GLY	B
140	238	TRP	B
141	239	GLN	B
142	240	LYS	B
143	241	SER	B
144	242	TRP	B
145	243	MET	B
146	244	SER	B
147	245	SER	B
148	246	ILE	B
149	247	TYR	B
150	252	VAL	B
151	253	PHE	B
152	254	THR	B
153	255	PHE	C
154	256	GLY	B
155	257	GLU	B
156	258	TRP	B
157	259	PHE	B
158	260	LEU	B
159	261	GLY	B
160	269	ASN	B
161	270	THR	B
162	271	ASP	B
163	272	PHE	B
164	274	ASN	B
165	275	LYS	B
166	277	GLY	B
167	278	MET	B
168	279	SER	B
169	280	LEU	B
170	281	LEU	D
171	282	ASP	D
172	283	PHE	D
173	284	ARG	D
174	286	ASN	D
175	287	SER	D
176	288	ALA	D
177	289	VAL	D
178	290	ARG	D
179	291	ASN	D
180	292	VAL	D
181	293	PHE	D

Continued on next page

Table S42 – *Continued from previous page*

**Homology index**	**Residue Index**	**Residue abbreviation**	**Module**
182	298	SER	D
183	299	ASN	A
184	300	MET	A
185	301	TYR	A
186	306	MET	A
187	307	ILE	D
188	308	ASN	D
189	309	SER	D
190	310	THR	D
191	311	ALA	B
192	312	THR	B
193	316	GLN	B
194	317	VAL	B
195	318	ASN	B
196	319	ASP	B
197	320	GLN	B
198	321	VAL	D
199	322	THR	D
200	323	PHE	D
201	324	ILE	A
202	325	ASP	D
203	326	ASN	D
204	327	HIS	D
205	328	ASP	D
206	329	MET	D
207	330	ASP	D
208	331	ARG	D
209	332	PHE	D
210	333	LYS	D
211	334	THR	D
212	339	ASN	A
213	341	ARG	A
214	342	LEU	A
215	343	GLU	A
216	344	GLN	A
217	345	ALA	A
218	346	LEU	A
219	347	ALA	A
220	348	PHE	A
221	349	THR	A
222	350	LEU	A
223	351	THR	A
224	352	SER	A
225	353	ARG	D
226	354	GLY	D
227	355	VAL	D

Continued on next page

Table S42 – *Continued from previous page*

Homology index	Residue Index	Residue abbreviation	Module
228	356	PRO	A
229	357	ALA	D
230	358	ILE	A
231	359	TYR	D
232	360	TYR	C
233	361	GLY	D
234	362	THR	A
235	376	ALA	C
236	377	LYS	C
237	378	MET	D
238	379	PRO	D
239	381	PHE	D
240	387	ALA	A
241	388	PHE	A
242	389	ASN	A
243	390	VAL	A
244	391	ILE	A
245	392	SER	A
246	393	LYS	A
247	394	LEU	A
248	395	ALA	A
249	396	PRO	A
250	397	LEU	A
251	398	ARG	A
252	399	LYS	A
253	400	SER	A
254	401	ASN	A
255	402	PRO	A
256	406	TYR	A
257	407	GLY	A
258	408	SER	A
259	409	THR	A
260	411	GLN	A
261	412	ARG	A
262	413	TRP	A
263	414	ILE	A
264	415	ASN	A
265	416	ASN	A
266	417	ASP	A
267	418	VAL	A
268	419	TYR	A
269	420	VAL	A
270	421	TYR	A
271	422	GLU	A
272	423	ARG	A
273	428	SER	A

Continued on next page

Table S42 – *Continued from previous page*

**Homology index**	**Residue Index**	**Residue abbreviation**	**Module**
274	429	VAL	A
275	430	ALA	A
276	431	VAL	A
277	432	VAL	A
278	433	ALA	A
279	434	VAL	A
280	435	ASN	A
281	439	SER	A
282	440	THR	A
283	441	SER	A
284	442	ALA	A
285	443	SER	A
286	444	ILE	A
287	445	THR	A
288	454	GLY	A
289	455	SER	A
290	457	THR	A
291	458	ASP	A
292	459	VAL	A
293	460	LEU	A
294	482	ALA	A
295	483	ALA	A
296	484	GLY	A
297	485	ALA	A
298	486	THR	A
299	487	ALA	A
300	488	VAL	A
301	489	TRP	A
302	490	GLN	A

Table S43: Residues membership for the *B. circulans* α-amylase (PDB code 1CXE, chain A)

**Homology index**	**Residue Index**	**Residue abbreviation**	**Module**
0	15	ASP	A
1	16	VAL	A
2	17	ILE	B
3	18	TYR	B
4	19	GLN	B
5	20	ILE	C
6	21	PHE	C
7	22	THR	C
8	52	GLY	C

Continued on next page

Table S43 – *Continued from previous page*

Homology index	Residue Index	Residue abbreviation	Module
9	53	ASP	C
10	54	TRP	C
11	55	GLN	C
12	56	GLY	C
13	57	ILE	C
14	58	ILE	C
15	59	ASN	C
16	60	LYS	C
17	61	ILE	C
18	62	ASN	C
19	63	ASP	C
20	66	LEU	C
21	67	THR	C
22	68	GLY	C
23	69	MET	C
24	70	GLY	C
25	71	VAL	C
26	72	THR	C
27	73	ALA	C
28	74	ILE	C
29	75	TRP	C
30	76	ILE	C
31	77	SER	C
32	78	GLN	C
33	79	PRO	C
34	80	VAL	C
35	81	GLU	C
36	82	ASN	C
37	83	ILE	C
38	95	THR	C
39	97	TYR	C
40	98	HIS	C
41	99	GLY	C
42	100	TYR	C
43	101	TRP	C
44	102	ALA	C
45	103	ARG	C
46	104	ASP	C
47	106	LYS	C
48	107	LYS	C
49	108	THR	C
50	109	ASN	C
51	110	PRO	C
52	111	ALA	C
53	112	TYR	C
54	113	GLY	C

Continued on next page

Table S43 – *Continued from previous page*

Homology index	Residue Index	Residue abbreviation	Module
55	114	THR	C
56	115	ILE	C
57	116	ALA	C
58	117	ASP	C
59	118	PHE	C
60	119	GLN	C
61	120	ASN	C
62	121	LEU	C
63	122	ILE	C
64	123	ALA	C
65	124	ALA	C
66	125	ALA	C
67	126	HIS	C
68	127	ALA	C
69	128	LYS	C
70	129	ASN	C
71	130	ILE	C
72	131	LYS	C
73	132	VAL	C
74	133	ILE	C
75	134	ILE	C
76	135	ASP	C
77	136	PHE	C
78	137	ALA	C
79	138	PRO	C
80	139	ASN	C
81	140	HIS	C
82	141	THR	C
83	142	SER	C
84	143	PRO	C
85	144	ALA	C
86	156	ARG	C
87	163	LEU	C
88	164	LEU	C
89	165	GLY	C
90	172	GLN	C
91	173	ASN	C
92	174	LEU	C
93	175	PHE	C
94	176	HIS	B
95	192	LYS	B
96	193	ASN	B
97	194	LEU	B
98	195	TYR	B
99	197	LEU	B
100	198	ALA	B

Continued on next page

Table S43 – *Continued from previous page*

Homology index	Residue Index	Residue abbreviation	Module
101	199	ASP	B
102	200	LEU	B
103	201	ASN	B
104	202	HIS	B
105	203	ASN	B
106	204	ASN	B
107	205	SER	B
108	206	THR	B
109	207	VAL	B
110	208	ASP	B
111	209	VAL	B
112	210	TYR	B
113	211	LEU	B
114	212	LYS	B
115	213	ASP	B
116	214	ALA	B
117	215	ILE	B
118	216	LYS	B
119	217	MET	B
120	218	TRP	B
121	219	LEU	B
122	220	ASP	B
123	221	LEU	C
124	222	GLY	C
125	223	ILE	C
126	224	ASP	C
127	225	GLY	C
128	226	ILE	B
129	227	ARG	D
130	228	MET	B
131	229	ASP	B
132	230	ALA	B
133	231	VAL	B
134	232	LYS	B
135	233	HIS	B
136	234	MET	B
137	235	PRO	B
138	236	PHE	B
139	237	GLY	B
140	238	TRP	B
141	239	GLN	B
142	240	LYS	B
143	241	SER	B
144	242	PHE	B
145	243	MET	B
146	244	ALA	B

Continued on next page

Table S43 – *Continued from previous page*

Homology index	Residue Index	Residue abbreviation	Module
147	245	ALA	B
148	246	VAL	B
149	247	ASN	B
150	252	VAL	B
151	253	PHE	B
152	254	THR	B
153	255	PHE	C
154	256	GLY	B
155	257	GLU	B
156	258	TRP	B
157	259	PHE	B
158	260	LEU	B
159	261	GLY	B
160	269	ASN	B
161	270	HIS	B
162	271	LYS	B
163	272	PHE	B
164	274	ASN	B
165	275	GLU	B
166	277	GLY	B
167	278	MET	B
168	279	SER	B
169	280	LEU	B
170	281	LEU	D
171	282	ASP	D
172	283	PHE	D
173	284	ARG	D
174	286	ALA	D
175	287	GLN	D
176	288	LYS	D
177	289	VAL	D
178	290	ARG	D
179	291	GLN	D
180	292	VAL	D
181	293	PHE	D
182	298	ASP	D
183	299	ASN	A
184	300	MET	A
185	301	TYR	A
186	306	MET	A
187	307	LEU	D
188	308	GLU	D
189	309	GLY	D
190	310	SER	D
191	311	ALA	B
192	312	ALA	B

Continued on next page

Table S43 – *Continued from previous page*

Homology index	Residue Index	Residue abbreviation	Module
193	316	GLN	B
194	317	VAL	B
195	318	ASP	B
196	319	ASP	B
197	320	GLN	B
198	321	VAL	D
199	322	THR	D
200	323	PHE	D
201	324	ILE	A
202	325	ASP	D
203	326	ASN	D
204	327	HIS	D
205	328	ASP	D
206	329	MET	D
207	330	GLU	D
208	331	ARG	D
209	332	PHE	D
210	333	HIS	D
211	334	ALA	D
212	339	ARG	A
213	341	LYS	A
214	342	LEU	A
215	343	GLU	A
216	344	GLN	A
217	345	ALA	A
218	346	LEU	A
219	347	ALA	A
220	348	PHE	A
221	349	THR	A
222	350	LEU	A
223	351	THR	A
224	352	SER	A
225	353	ARG	D
226	354	GLY	D
227	355	VAL	D
228	356	PRO	A
229	357	ALA	D
230	358	ILE	A
231	359	TYR	D
232	360	TYR	C
233	361	GLY	D
234	362	THR	A
235	376	ALA	C
236	377	ARG	C
237	378	ILE	D
238	379	PRO	D

Continued on next page

Table S43 – *Continued from previous page*

Homology index	Residue Index	Residue abbreviation	Module
239	381	PHE	D
240	387	ALA	A
241	388	TYR	A
242	389	GLN	A
243	390	VAL	A
244	391	ILE	A
245	392	GLN	A
246	393	LYS	A
247	394	LEU	A
248	395	ALA	A
249	396	PRO	A
250	397	LEU	A
251	398	ARG	A
252	399	LYS	A
253	400	CYS	A
254	401	ASN	A
255	402	PRO	A
256	406	TYR	A
257	407	GLY	A
258	408	SER	A
259	409	THR	A
260	411	GLU	A
261	412	ARG	A
262	413	TRP	A
263	414	ILE	A
264	415	ASN	A
265	416	ASN	A
266	417	ASP	A
267	418	VAL	A
268	419	LEU	A
269	420	ILE	A
270	421	TYR	A
271	422	GLU	A
272	423	ARG	A
273	428	ASN	A
274	429	VAL	A
275	430	ALA	A
276	431	VAL	A
277	432	VAL	A
278	433	ALA	A
279	434	VAL	A
280	435	ASN	A
281	439	ASN	A
282	440	ALA	A
283	441	PRO	A
284	442	ALA	A

Continued on next page

Table S43 – *Continued from previous page*

Homology index	Residue Index	Residue abbreviation	Module
285	443	SER	A
286	444	ILE	A
287	445	SER	A
288	454	GLY	A
289	455	SER	A
290	457	ASN	A
291	458	ASP	A
292	459	VAL	A
293	460	LEU	A
294	483	ALA	A
295	484	ALA	A
296	485	GLY	A
297	486	GLY	A
298	487	THR	A
299	488	ALA	A
300	489	VAL	A
301	490	TRP	A
302	491	GLN	A

Table S44: Residues membership for the *T. molitor* α-amylase (PDB code 1CLV, chain A)

Homology index	Residue Index	Residue abbreviation	Module
0	11	ASN	A
1	12	SER	A
2	13	ILE	B
3	14	VAL	B
4	15	HIS	B
5	16	LEU	C
6	17	PHE	C
7	18	GLU	C
8	19	TRP	C
9	20	LYS	C
10	21	TRP	C
11	22	ASN	C
12	23	ASP	C
13	24	ILE	C
14	25	ALA	C
15	26	ASP	C
16	27	GLU	C
17	28	CYS	C
18	29	GLU	C
19	30	ARG	C

Continued on next page

Table S44 – *Continued from previous page*

Homology index	Residue Index	Residue abbreviation	Module
20	32	LEU	C
21	33	GLN	C
22	34	PRO	C
23	35	GLN	C
24	36	GLY	C
25	37	PHE	C
26	38	GLY	C
27	39	GLY	C
28	40	VAL	C
29	41	GLN	C
30	42	ILE	C
31	43	SER	C
32	44	PRO	C
33	45	PRO	C
34	46	ASN	C
35	47	GLU	C
36	48	TYR	C
37	49	LEU	C
38	51	ALA	C
39	57	TRP	C
40	58	GLU	C
41	59	ARG	C
42	60	TYR	C
43	61	GLN	C
44	62	PRO	C
45	63	VAL	C
46	64	SER	C
47	65	TYR	C
48	66	ILE	C
49	67	ILE	C
50	68	ASN	C
51	69	THR	C
52	70	ARG	C
53	71	SER	C
54	72	GLY	C
55	73	ASP	C
56	74	GLU	C
57	75	SER	C
58	76	ALA	C
59	77	PHE	C
60	78	THR	C
61	79	ASP	C
62	80	MET	C
63	81	THR	C
64	82	ARG	C
65	83	ARG	C

Continued on next page

Table S44 – *Continued from previous page*

Homology index	Residue Index	Residue abbreviation	Module
66	84	CYS	C
67	85	ASN	C
68	86	ASP	C
69	87	ALA	C
70	88	GLY	C
71	89	VAL	C
72	90	ARG	C
73	91	ILE	C
74	92	TYR	C
75	93	VAL	C
76	94	ASP	C
77	95	ALA	C
78	96	VAL	C
79	97	ILE	C
80	98	ASN	C
81	99	HIS	C
82	100	MET	C
83	101	THR	C
84	102	GLY	C
85	103	MET	C
86	120	TYR	C
87	124	PRO	C
88	125	TYR	C
89	126	GLY	C
90	127	SER	C
91	128	GLY	C
92	129	ASP	C
93	130	PHE	C
94	131	HIS	B
95	148	CYS	B
96	149	GLU	B
97	150	LEU	B
98	151	VAL	B
99	153	LEU	B
100	154	ARG	B
101	155	ASP	B
102	156	LEU	B
103	157	ASN	B
104	158	GLN	B
105	159	GLY	B
106	160	SER	B
107	161	ASP	B
108	162	TYR	B
109	163	VAL	B
110	164	ARG	B
111	165	GLY	B

Continued on next page

Table S44 – *Continued from previous page*

Homology index	Residue Index	Residue abbreviation	Module
112	166	VAL	B
113	167	LEU	B
114	168	ILE	B
115	169	ASP	B
116	170	TYR	B
117	171	MET	B
118	172	ASN	B
119	173	HIS	B
120	174	MET	B
121	175	ILE	B
122	176	ASP	B
123	177	LEU	C
124	178	GLY	C
125	179	VAL	C
126	180	ALA	C
127	181	GLY	C
128	182	PHE	B
129	183	ARG	D
130	184	VAL	B
131	185	ASP	B
132	186	ALA	B
133	187	ALA	B
134	188	LYS	B
135	189	HIS	B
136	190	MET	B
137	191	SER	B
138	192	PRO	B
139	193	GLY	B
140	194	ASP	B
141	195	LEU	B
142	196	SER	B
143	197	VAL	B
144	198	ILE	B
145	199	PHE	B
146	200	SER	B
147	201	GLY	B
148	202	LEU	B
149	203	LYS	B
150	217	PRO	B
151	218	PHE	B
152	219	ILE	B
153	220	TYR	C
154	221	GLN	B
155	222	GLU	B
156	223	VAL	B
157	224	ILE	B

Continued on next page

Table S44 – *Continued from previous page*

Homology index	Residue Index	Residue abbreviation	Module
158	225	ASP	B
159	226	LEU	B
160	233	LYS	B
161	234	ASN	B
162	235	GLU	B
163	236	TYR	B
164	237	THR	B
165	238	GLY	B
166	239	PHE	B
167	240	GLY	B
168	241	CYS	B
169	242	VAL	B
170	243	LEU	D
171	244	GLU	D
172	245	PHE	D
173	246	GLN	D
174	248	GLY	D
175	249	VAL	D
176	250	SER	D
177	251	LEU	D
178	252	GLY	D
179	253	ASN	D
180	254	ALA	D
181	255	PHE	D
182	259	ASN	D
183	260	GLN	A
184	261	LEU	A
185	262	LYS	A
186	267	TRP	A
187	268	GLY	D
188	269	PRO	D
189	270	GLU	D
190	272	GLY	D
191	273	LEU	B
192	274	LEU	B
193	275	GLU	B
194	276	GLY	B
195	277	LEU	B
196	278	ASP	B
197	279	ALA	B
198	280	VAL	D
199	281	VAL	D
200	282	PHE	D
201	283	VAL	A
202	284	ASP	D
203	285	ASN	D

Continued on next page

Table S44 – *Continued from previous page*

Homology index	Residue Index	Residue abbreviation	Module
204	286	HIS	D
205	287	ASP	D
206	288	ASN	D
207	289	GLN	D
208	298	THR	D
209	299	TYR	D
210	300	LYS	D
211	301	ASN	D
212	303	LYS	A
213	304	PRO	A
214	305	TYR	A
215	306	LYS	A
216	307	MET	A
217	308	ALA	A
218	309	ILE	A
219	310	ALA	A
220	311	PHE	A
221	312	MET	A
222	313	LEU	A
223	314	ALA	A
224	315	HIS	A
225	317	TYR	D
226	318	GLY	D
227	319	THR	D
228	320	THR	A
229	321	ARG	D
230	322	ILE	A
231	323	MET	D
232	324	SER	C
233	325	SER	D
234	326	PHE	A
235	327	ASP	C
236	357	GLY	C
237	358	TYR	D
238	359	VAL	D
239	360	CYS	D
240	363	ARG	A
241	364	TRP	A
242	365	ARG	A
243	366	GLN	A
244	367	VAL	A
245	368	TYR	A
246	369	GLY	A
247	370	MET	A
248	371	VAL	A
249	372	GLY	A

Continued on next page

Table S44 – *Continued from previous page*

Homology index	Residue Index	Residue abbreviation	Module
250	373	PHE	A
251	374	ARG	A
252	375	ASN	A
253	376	ALA	A
254	377	VAL	A
255	378	GLU	A
256	379	GLY	A
257	380	THR	A
258	381	GLN	A
259	382	VAL	A
260	384	ASN	A
261	385	TRP	A
262	386	TRP	A
263	387	SER	A
264	389	ASP	A
265	390	ASP	A
266	391	ASN	A
267	392	GLN	A
268	393	ILE	A
269	394	ALA	A
270	395	PHE	A
271	396	SER	A
272	397	ARG	A
273	398	GLY	A
274	399	SER	A
275	401	GLY	A
276	402	PHE	A
277	403	VAL	A
278	404	ALA	A
279	405	PHE	A
280	406	THR	A
281	408	GLY	A
282	409	GLY	A
283	410	ASP	A
284	411	LEU	A
285	412	ASN	A
286	413	GLN	A
287	414	ASN	A
288	420	PRO	A
289	421	ALA	A
290	424	TYR	A
291	425	CYS	A
292	426	ASP	A
293	427	VAL	A
294	454	LEU	A
295	455	GLY	A

Continued on next page

Table S44 – *Continued from previous page*

Homology index	Residue Index	Residue abbreviation	Module
296	456	SER	A
297	461	GLY	A
298	462	VAL	A
299	463	LEU	A
300	464	ALA	A
301	465	ILE	A
302	466	HIS	A

Table S45: Residues membership for the *B. circulans* α-amylase (PDB code 1CXK, chain A)

Homology index	Residue Index	Residue abbreviation	Module
0	15	ASP	A
1	16	VAL	A
2	17	ILE	B
3	18	TYR	B
4	19	GLN	B
5	20	ILE	C
6	21	PHE	C
7	22	THR	C
8	52	GLY	C
9	53	ASP	C
10	54	TRP	C
11	55	GLN	C
12	56	GLY	C
13	57	ILE	C
14	58	ILE	C
15	59	ASN	C
16	60	LYS	C
17	61	ILE	C
18	62	ASN	C
19	63	ASP	C
20	66	LEU	C
21	67	THR	C
22	68	GLY	C
23	69	MET	C
24	70	GLY	C
25	71	VAL	C
26	72	THR	C
27	73	ALA	C
28	74	ILE	C
29	75	TRP	C
30	76	ILE	C

Continued on next page

Table S45 – *Continued from previous page*

Homology index	Residue Index	Residue abbreviation	Module
31	77	SER	C
32	78	GLN	C
33	79	PRO	C
34	80	VAL	C
35	81	GLU	C
36	82	ASN	C
37	83	ILE	C
38	95	THR	C
39	97	TYR	C
40	98	HIS	C
41	99	GLY	C
42	100	TYR	C
43	101	TRP	C
44	102	ALA	C
45	103	ARG	C
46	104	ASP	C
47	106	LYS	C
48	107	LYS	C
49	108	THR	C
50	109	ASN	C
51	110	PRO	C
52	111	ALA	C
53	112	TYR	C
54	113	GLY	C
55	114	THR	C
56	115	ILE	C
57	116	ALA	C
58	117	ASP	C
59	118	PHE	C
60	119	GLN	C
61	120	ASN	C
62	121	LEU	C
63	122	ILE	C
64	123	ALA	C
65	124	ALA	C
66	125	ALA	C
67	126	HIS	C
68	127	ALA	C
69	128	LYS	C
70	129	ASN	C
71	130	ILE	C
72	131	LYS	C
73	132	VAL	C
74	133	ILE	C
75	134	ILE	C
76	135	ASP	C

Continued on next page

Table S45 – *Continued from previous page*

Homology index	Residue Index	Residue abbreviation	Module
77	136	PHE	C
78	137	ALA	C
79	138	PRO	C
80	139	ASN	C
81	140	HIS	C
82	141	THR	C
83	142	SER	C
84	143	PRO	C
85	144	ALA	C
86	156	ARG	C
87	163	LEU	C
88	164	LEU	C
89	165	GLY	C
90	172	GLN	C
91	173	ASN	C
92	174	LEU	C
93	175	PHE	C
94	176	HIS	B
95	192	LYS	B
96	193	ASN	B
97	194	LEU	B
98	195	TYR	B
99	197	LEU	B
100	198	ALA	B
101	199	ASP	B
102	200	LEU	B
103	201	ASN	B
104	202	HIS	B
105	203	ASN	B
106	204	ASN	B
107	205	SER	B
108	206	THR	B
109	207	VAL	B
110	208	ASP	B
111	209	VAL	B
112	210	TYR	B
113	211	LEU	B
114	212	LYS	B
115	213	ASP	B
116	214	ALA	B
117	215	ILE	B
118	216	LYS	B
119	217	MET	B
120	218	TRP	B
121	219	LEU	B
122	220	ASP	B

Continued on next page

Table S45 – *Continued from previous page*

Homology index	Residue Index	Residue abbreviation	Module
123	221	LEU	C
124	222	GLY	C
125	223	ILE	C
126	224	ASP	C
127	225	GLY	C
128	226	ILE	B
129	227	ARG	D
130	228	MET	B
131	229	ASN	B
132	230	ALA	B
133	231	VAL	B
134	232	LYS	B
135	233	HIS	B
136	234	MET	B
137	235	PRO	B
138	236	PHE	B
139	237	GLY	B
140	238	TRP	B
141	239	GLN	B
142	240	LYS	B
143	241	SER	B
144	242	PHE	B
145	243	MET	B
146	244	ALA	B
147	245	ALA	B
148	246	VAL	B
149	247	ASN	B
150	252	VAL	B
151	253	PHE	B
152	254	THR	B
153	255	PHE	C
154	256	GLY	B
155	257	GLN	B
156	258	TRP	B
157	259	PHE	B
158	260	LEU	B
159	261	GLY	B
160	269	ASN	B
161	270	HIS	B
162	271	LYS	B
163	272	PHE	B
164	274	ASN	B
165	275	GLU	B
166	277	GLY	B
167	278	MET	B
168	279	SER	B

Continued on next page

Table S45 – *Continued from previous page*

Homology index	Residue Index	Residue abbreviation	Module
169	280	LEU	B
170	281	LEU	D
171	282	ASP	D
172	283	PHE	D
173	284	ARG	D
174	286	ALA	D
175	287	GLN	D
176	288	LYS	D
177	289	VAL	D
178	290	ARG	D
179	291	GLN	D
180	292	VAL	D
181	293	PHE	D
182	298	ASP	D
183	299	ASN	A
184	300	MET	A
185	301	TYR	A
186	306	MET	A
187	307	LEU	D
188	308	GLU	D
189	309	GLY	D
190	310	SER	D
191	311	ALA	B
192	312	ALA	B
193	316	GLN	B
194	317	VAL	B
195	318	ASP	B
196	319	ASP	B
197	320	GLN	B
198	321	VAL	D
199	322	THR	D
200	323	PHE	D
201	324	ILE	A
202	325	ASP	D
203	326	ASN	D
204	327	HIS	D
205	328	ASP	D
206	329	MET	D
207	330	GLU	D
208	331	ARG	D
209	332	PHE	D
210	333	HIS	D
211	334	ALA	D
212	339	ARG	A
213	341	LYS	A
214	342	LEU	A

Continued on next page

Table S45 – *Continued from previous page*

Homology index	Residue Index	Residue abbreviation	Module
215	343	GLU	A
216	344	GLN	A
217	345	ALA	A
218	346	LEU	A
219	347	ALA	A
220	348	PHE	A
221	349	THR	A
222	350	LEU	A
223	351	THR	A
224	352	SER	A
225	353	ARG	D
226	354	GLY	D
227	355	VAL	D
228	356	PRO	A
229	357	ALA	D
230	358	ILE	A
231	359	TYR	D
232	360	TYR	C
233	361	GLY	D
234	362	THR	A
235	376	ALA	C
236	377	ARG	C
237	378	ILE	D
238	379	PRO	D
239	381	PHE	D
240	387	ALA	A
241	388	TYR	A
242	389	GLN	A
243	390	VAL	A
244	391	ILE	A
245	392	GLN	A
246	393	LYS	A
247	394	LEU	A
248	395	ALA	A
249	396	PRO	A
250	397	LEU	A
251	398	ARG	A
252	399	LYS	A
253	400	CYS	A
254	401	ASN	A
255	402	PRO	A
256	406	TYR	A
257	407	GLY	A
258	408	SER	A
259	409	THR	A
260	411	GLU	A

Continued on next page

Table S45 – *Continued from previous page*

Homology index	Residue Index	Residue abbreviation	Module
261	412	ARG	A
262	413	TRP	A
263	414	ILE	A
264	415	ASN	A
265	416	ASN	A
266	417	ASP	A
267	418	VAL	A
268	419	LEU	A
269	420	ILE	A
270	421	TYR	A
271	422	GLU	A
272	423	ARG	A
273	428	ASN	A
274	429	VAL	A
275	430	ALA	A
276	431	VAL	A
277	432	VAL	A
278	433	ALA	A
279	434	VAL	A
280	435	ASN	A
281	439	ASN	A
282	440	ALA	A
283	441	PRO	A
284	442	ALA	A
285	443	SER	A
286	444	ILE	A
287	445	SER	A
288	454	GLY	A
289	455	SER	A
290	457	ASN	A
291	458	ASP	A
292	459	VAL	A
293	460	LEU	A
294	483	ALA	A
295	484	ALA	A
296	485	GLY	A
297	486	GLY	A
298	487	THR	A
299	488	ALA	A
300	489	VAL	A
301	490	TRP	A
302	491	GLN	A

Table S46: Residues membership for the *B. circulans* α-amylase (PDB code 1CXF, chain A)

Homology index	Residue Index	Residue abbreviation	Module
0	15	ASP	A
1	16	VAL	A
2	17	ILE	B
3	18	TYR	B
4	19	GLN	B
5	20	ILE	C
6	21	PHE	C
7	22	THR	C
8	52	GLY	C
9	53	ASP	C
10	54	TRP	C
11	55	GLN	C
12	56	GLY	C
13	57	ILE	C
14	58	ILE	C
15	59	ASN	C
16	60	LYS	C
17	61	ILE	C
18	62	ASN	C
19	63	ASP	C
20	66	LEU	C
21	67	THR	C
22	68	GLY	C
23	69	MET	C
24	70	GLY	C
25	71	VAL	C
26	72	THR	C
27	73	ALA	C
28	74	ILE	C
29	75	TRP	C
30	76	ILE	C
31	77	SER	C
32	78	GLN	C
33	79	PRO	C
34	80	VAL	C
35	81	GLU	C
36	82	ASN	C
37	83	ILE	C
38	95	THR	C
39	97	TYR	C
40	98	HIS	C
41	99	GLY	C
42	100	TYR	C
43	101	TRP	C

Continued on next page

Table S46 – *Continued from previous page*

**Homology index**	**Residue Index**	**Residue abbreviation**	**Module**
44	102	ALA	C
45	103	ARG	C
46	104	ASP	C
47	106	LYS	C
48	107	LYS	C
49	108	THR	C
50	109	ASN	C
51	110	PRO	C
52	111	ALA	C
53	112	TYR	C
54	113	GLY	C
55	114	THR	C
56	115	ILE	C
57	116	ALA	C
58	117	ASP	C
59	118	PHE	C
60	119	GLN	C
61	120	ASN	C
62	121	LEU	C
63	122	ILE	C
64	123	ALA	C
65	124	ALA	C
66	125	ALA	C
67	126	HIS	C
68	127	ALA	C
69	128	LYS	C
70	129	ASN	C
71	130	ILE	C
72	131	LYS	C
73	132	VAL	C
74	133	ILE	C
75	134	ILE	C
76	135	ASP	C
77	136	PHE	C
78	137	ALA	C
79	138	PRO	C
80	139	ASN	C
81	140	HIS	C
82	141	THR	C
83	142	SER	C
84	143	PRO	C
85	144	ALA	C
86	156	ARG	C
87	163	LEU	C
88	164	LEU	C
89	165	GLY	C

Continued on next page

Table S46 – *Continued from previous page*

Homology index	Residue Index	Residue abbreviation	Module
90	172	GLN	C
91	173	ASN	C
92	174	LEU	C
93	175	PHE	C
94	176	HIS	B
95	192	LYS	B
96	193	ASN	B
97	194	LEU	B
98	195	TYR	B
99	197	LEU	B
100	198	ALA	B
101	199	ASP	B
102	200	LEU	B
103	201	ASN	B
104	202	HIS	B
105	203	ASN	B
106	204	ASN	B
107	205	SER	B
108	206	THR	B
109	207	VAL	B
110	208	ASP	B
111	209	VAL	B
112	210	TYR	B
113	211	LEU	B
114	212	LYS	B
115	213	ASP	B
116	214	ALA	B
117	215	ILE	B
118	216	LYS	B
119	217	MET	B
120	218	TRP	B
121	219	LEU	B
122	220	ASP	B
123	221	LEU	C
124	222	GLY	C
125	223	ILE	C
126	224	ASP	C
127	225	GLY	C
128	226	ILE	B
129	227	ARG	D
130	228	MET	B
131	229	ASN	B
132	230	ALA	B
133	231	VAL	B
134	232	LYS	B
135	233	HIS	B

Continued on next page

Table S46 – *Continued from previous page*

Homology index	Residue Index	Residue abbreviation	Module
136	234	MET	B
137	235	PRO	B
138	236	PHE	B
139	237	GLY	B
140	238	TRP	B
141	239	GLN	B
142	240	LYS	B
143	241	SER	B
144	242	PHE	B
145	243	MET	B
146	244	ALA	B
147	245	ALA	B
148	246	VAL	B
149	247	ASN	B
150	252	VAL	B
151	253	PHE	B
152	254	THR	B
153	255	PHE	C
154	256	GLY	B
155	257	GLN	B
156	258	TRP	B
157	259	PHE	B
158	260	LEU	B
159	261	GLY	B
160	269	ASN	B
161	270	HIS	B
162	271	LYS	B
163	272	PHE	B
164	274	ASN	B
165	275	GLU	B
166	277	GLY	B
167	278	MET	B
168	279	SER	B
169	280	LEU	B
170	281	LEU	D
171	282	ASP	D
172	283	PHE	D
173	284	ARG	D
174	286	ALA	D
175	287	GLN	D
176	288	LYS	D
177	289	VAL	D
178	290	ARG	D
179	291	GLN	D
180	292	VAL	D
181	293	PHE	D

Continued on next page

Table S46 – *Continued from previous page*

Homology index	Residue Index	Residue abbreviation	Module
182	298	ASP	D
183	299	ASN	A
184	300	MET	A
185	301	TYR	A
186	306	MET	A
187	307	LEU	D
188	308	GLU	D
189	309	GLY	D
190	310	SER	D
191	311	ALA	B
192	312	ALA	B
193	316	GLN	B
194	317	VAL	B
195	318	ASP	B
196	319	ASP	B
197	320	GLN	B
198	321	VAL	D
199	322	THR	D
200	323	PHE	D
201	324	ILE	A
202	325	ASP	D
203	326	ASN	D
204	327	HIS	D
205	328	ASP	D
206	329	MET	D
207	330	GLU	D
208	331	ARG	D
209	332	PHE	D
210	333	HIS	D
211	334	ALA	D
212	339	ARG	A
213	341	LYS	A
214	342	LEU	A
215	343	GLU	A
216	344	GLN	A
217	345	ALA	A
218	346	LEU	A
219	347	ALA	A
220	348	PHE	A
221	349	THR	A
222	350	LEU	A
223	351	THR	A
224	352	SER	A
225	353	ARG	D
226	354	GLY	D
227	355	VAL	D

Continued on next page

Table S46 – *Continued from previous page*

Homology index	Residue Index	Residue abbreviation	Module
228	356	PRO	A
229	357	ALA	D
230	358	ILE	A
231	359	TYR	D
232	360	TYR	C
233	361	GLY	D
234	362	THR	A
235	376	ALA	C
236	377	ARG	C
237	378	ILE	D
238	379	PRO	D
239	381	PHE	D
240	387	ALA	A
241	388	TYR	A
242	389	GLN	A
243	390	VAL	A
244	391	ILE	A
245	392	GLN	A
246	393	LYS	A
247	394	LEU	A
248	395	ALA	A
249	396	PRO	A
250	397	LEU	A
251	398	ARG	A
252	399	LYS	A
253	400	CYS	A
254	401	ASN	A
255	402	PRO	A
256	406	TYR	A
257	407	GLY	A
258	408	SER	A
259	409	THR	A
260	411	GLU	A
261	412	ARG	A
262	413	TRP	A
263	414	ILE	A
264	415	ASN	A
265	416	ASN	A
266	417	ASP	A
267	418	VAL	A
268	419	LEU	A
269	420	ILE	A
270	421	TYR	A
271	422	GLU	A
272	423	ARG	A
273	428	ASN	A

Continued on next page

Table S46 – *Continued from previous page*

Homology index	Residue Index	Residue abbreviation	Module
274	429	VAL	A
275	430	ALA	A
276	431	VAL	A
277	432	VAL	A
278	433	ALA	A
279	434	VAL	A
280	435	ASN	A
281	439	ASN	A
282	440	ALA	A
283	441	PRO	A
284	442	ALA	A
285	443	SER	A
286	444	ILE	A
287	445	SER	A
288	454	GLY	A
289	455	SER	A
290	457	ASN	A
291	458	ASP	A
292	459	VAL	A
293	460	LEU	A
294	483	ALA	A
295	484	ALA	A
296	485	GLY	A
297	486	GLY	A
298	487	THR	A
299	488	ALA	A
300	489	VAL	A
301	490	TRP	A
302	491	GLN	A

Table S47: Residues membership for the *B. stearothermophilus* α-amylase (PDB code 1CYG, chain A)

Homology index	Residue Index	Residue abbreviation	Module
0	12	ASP	A
1	13	VAL	A
2	14	VAL	B
3	15	TYR	B
4	16	GLN	B
5	17	ILE	C
6	18	VAL	C
7	19	VAL	C
8	49	GLY	C

Continued on next page

Table S47 – *Continued from previous page*

Homology index	Residue Index	Residue abbreviation	Module
9	50	ASP	C
10	51	TRP	C
11	52	GLN	C
12	53	GLY	C
13	54	ILE	C
14	55	ILE	C
15	56	ASN	C
16	57	LYS	C
17	58	ILE	C
18	59	ASN	C
19	60	ASP	C
20	63	LEU	C
21	64	THR	C
22	65	ASP	C
23	66	MET	C
24	67	GLY	C
25	68	VAL	C
26	69	THR	C
27	70	ALA	C
28	71	ILE	C
29	72	TRP	C
30	73	ILE	C
31	74	SER	C
32	75	GLN	C
33	76	PRO	C
34	77	VAL	C
35	78	GLU	C
36	79	ASN	C
37	80	VAL	C
38	91	ALA	C
39	93	TYR	C
40	94	HIS	C
41	95	GLY	C
42	96	TYR	C
43	97	TRP	C
44	98	ALA	C
45	99	ARG	C
46	100	ASP	C
47	102	LYS	C
48	103	LYS	C
49	104	PRO	C
50	105	ASN	C
51	106	PRO	C
52	107	PHE	C
53	108	PHE	C
54	109	GLY	C

Continued on next page

Table S47 – *Continued from previous page*

**Homology index**	**Residue Index**	**Residue abbreviation**	**Module**
55	110	THR	C
56	111	LEU	C
57	112	SER	C
58	113	ASP	C
59	114	PHE	C
60	115	GLN	C
61	116	ARG	C
62	117	LEU	C
63	118	VAL	C
64	119	ASP	C
65	120	ALA	C
66	121	ALA	C
67	122	HIS	C
68	123	ALA	C
69	124	LYS	C
70	125	GLY	C
71	126	ILE	C
72	127	LYS	C
73	128	VAL	C
74	129	ILE	C
75	130	ILE	C
76	131	ASP	C
77	132	PHE	C
78	133	ALA	C
79	134	PRO	C
80	135	ASN	C
81	136	HIS	C
82	137	THR	C
83	138	SER	C
84	139	PRO	C
85	140	ALA	C
86	152	ARG	C
87	159	LEU	C
88	160	LEU	C
89	161	GLY	C
90	168	ASN	C
91	169	MET	C
92	170	TYR	C
93	171	PHE	C
94	172	HIS	B
95	188	ARG	B
96	189	ASN	B
97	190	LEU	B
98	191	PHE	B
99	193	LEU	B
100	194	ALA	B

Continued on next page

Table S47 – *Continued from previous page*

Homology index	Residue Index	Residue abbreviation	Module
101	195	ASP	B
102	196	LEU	B
103	197	ASN	B
104	198	HIS	B
105	199	GLN	B
106	200	ASN	B
107	201	PRO	B
108	202	VAL	B
109	203	ILE	B
110	204	ASP	B
111	205	ARG	B
112	206	TYR	B
113	207	LEU	B
114	208	LYS	B
115	209	ASP	B
116	210	ALA	B
117	211	VAL	B
118	212	LYS	B
119	213	MET	B
120	214	TRP	B
121	215	ILE	B
122	216	ASP	B
123	217	MET	C
124	218	GLY	C
125	219	ILE	C
126	220	ASP	C
127	221	GLY	C
128	222	ILE	B
129	223	ARG	D
130	224	MET	B
131	225	ASP	B
132	226	ALA	B
133	227	VAL	B
134	228	LYS	B
135	229	HIS	B
136	230	MET	B
137	231	PRO	B
138	232	PHE	B
139	233	GLY	B
140	234	TRP	B
141	235	GLN	B
142	236	LYS	B
143	237	SER	B
144	238	LEU	B
145	239	MET	B
146	240	ASP	B

Continued on next page

Table S47 – *Continued from previous page*

Homology index	Residue Index	Residue abbreviation	Module
147	241	GLU	B
148	242	ILE	B
149	243	ASP	B
150	248	VAL	B
151	249	PHE	B
152	250	THR	B
153	251	PHE	C
154	252	GLY	B
155	253	GLU	B
156	254	TRP	B
157	255	PHE	B
158	256	LEU	B
159	257	SER	B
160	265	ASN	B
161	266	HIS	B
162	267	TYR	B
163	268	PHE	B
164	270	ASN	B
165	271	GLU	B
166	273	GLY	B
167	274	MET	B
168	275	SER	B
169	276	LEU	B
170	277	LEU	D
171	278	ASP	D
172	279	PHE	D
173	280	ARG	D
174	282	GLY	D
175	283	GLN	D
176	284	LYS	D
177	285	LEU	D
178	286	ARG	D
179	287	GLN	D
180	288	VAL	D
181	289	LEU	D
182	294	ASP	D
183	295	ASN	A
184	296	TRP	A
185	297	TYR	A
186	302	MET	A
187	303	ILE	D
188	304	GLN	D
189	305	ASP	D
190	306	THR	D
191	307	ALA	B
192	308	SER	B

Continued on next page

Table S47 – *Continued from previous page*

Homology index	Residue Index	Residue abbreviation	Module
193	312	GLU	B
194	313	VAL	B
195	314	LEU	B
196	315	ASP	B
197	316	GLN	B
198	317	VAL	D
199	318	THR	D
200	319	PHE	D
201	320	ILE	A
202	321	ASP	D
203	322	ASN	D
204	323	HIS	D
205	324	ASP	D
206	325	MET	D
207	326	ASP	D
208	327	ARG	D
209	328	PHE	D
210	329	MET	D
211	330	ILE	D
212	335	PRO	A
213	337	LYS	A
214	338	VAL	A
215	339	ASP	A
216	340	MET	A
217	341	ALA	A
218	342	LEU	A
219	343	ALA	A
220	344	VAL	A
221	345	LEU	A
222	346	LEU	A
223	347	THR	A
224	348	SER	A
225	349	ARG	D
226	350	GLY	D
227	351	VAL	D
228	352	PRO	A
229	353	ASN	D
230	354	ILE	A
231	355	TYR	D
232	356	TYR	C
233	357	GLY	D
234	358	THR	A
235	372	LYS	C
236	373	MET	C
237	374	MET	D
238	375	SER	D

Continued on next page

Table S47 – *Continued from previous page*

Homology index	Residue Index	Residue abbreviation	Module
239	377	PHE	D
240	383	ALA	A
241	384	TYR	A
242	385	GLN	A
243	386	VAL	A
244	387	ILE	A
245	388	GLN	A
246	389	LYS	A
247	390	LEU	A
248	391	SER	A
249	392	SER	A
250	393	LEU	A
251	394	ARG	A
252	395	ARG	A
253	396	ASN	A
254	397	ASN	A
255	398	PRO	A
256	402	TYR	A
257	403	GLY	A
258	404	ASP	A
259	405	THR	A
260	407	GLN	A
261	408	ARG	A
262	409	TRP	A
263	410	ILE	A
264	411	ASN	A
265	412	GLY	A
266	413	ASP	A
267	414	VAL	A
268	415	TYR	A
269	416	VAL	A
270	417	TYR	A
271	418	GLU	A
272	419	ARG	A
273	424	ASP	A
274	425	VAL	A
275	426	VAL	A
276	427	LEU	A
277	428	VAL	A
278	429	ALA	A
279	430	VAL	A
280	431	ASN	A
281	435	SER	A
282	436	SER	A
283	437	ASN	A
284	438	TYR	A

Continued on next page

Table S47 – *Continued from previous page*

Homology index	Residue Index	Residue abbreviation	Module
285	439	SER	A
286	440	ILE	A
287	441	THR	A
288	450	GLY	A
289	451	THR	A
290	453	THR	A
291	454	ASP	A
292	455	GLN	A
293	456	LEU	A
294	478	LEU	A
295	479	GLY	A
296	480	PRO	A
297	482	GLU	A
298	483	VAL	A
299	484	GLY	A
300	485	VAL	A
301	486	TRP	A
302	487	ALA	A

Table S48: Residues membership for the *B. circulans* α-amylase (PDB code 1CXL, chain A)

Homology index	Residue Index	Residue abbreviation	Module
0	15	ASP	A
1	16	VAL	A
2	17	ILE	B
3	18	TYR	B
4	19	GLN	B
5	20	ILE	C
6	21	PHE	C
7	22	THR	C
8	52	GLY	C
9	53	ASP	C
10	54	TRP	C
11	55	GLN	C
12	56	GLY	C
13	57	ILE	C
14	58	ILE	C
15	59	ASN	C
16	60	LYS	C
17	61	ILE	C
18	62	ASN	C
19	63	ASP	C

Continued on next page

Table S48 – *Continued from previous page*

**Homology index**	**Residue Index**	**Residue abbreviation**	**Module**
20	66	LEU	C
21	67	THR	C
22	68	GLY	C
23	69	MET	C
24	70	GLY	C
25	71	VAL	C
26	72	THR	C
27	73	ALA	C
28	74	ILE	C
29	75	TRP	C
30	76	ILE	C
31	77	SER	C
32	78	GLN	C
33	79	PRO	C
34	80	VAL	C
35	81	GLU	C
36	82	ASN	C
37	83	ILE	C
38	95	THR	C
39	97	TYR	C
40	98	HIS	C
41	99	GLY	C
42	100	TYR	C
43	101	TRP	C
44	102	ALA	C
45	103	ARG	C
46	104	ASP	C
47	106	LYS	C
48	107	LYS	C
49	108	THR	C
50	109	ASN	C
51	110	PRO	C
52	111	ALA	C
53	112	TYR	C
54	113	GLY	C
55	114	THR	C
56	115	ILE	C
57	116	ALA	C
58	117	ASP	C
59	118	PHE	C
60	119	GLN	C
61	120	ASN	C
62	121	LEU	C
63	122	ILE	C
64	123	ALA	C
65	124	ALA	C

Continued on next page

Table S48 – *Continued from previous page*

Homology index	Residue Index	Residue abbreviation	Module
66	125	ALA	C
67	126	HIS	C
68	127	ALA	C
69	128	LYS	C
70	129	ASN	C
71	130	ILE	C
72	131	LYS	C
73	132	VAL	C
74	133	ILE	C
75	134	ILE	C
76	135	ASP	C
77	136	PHE	C
78	137	ALA	C
79	138	PRO	C
80	139	ASN	C
81	140	HIS	C
82	141	THR	C
83	142	SER	C
84	143	PRO	C
85	144	ALA	C
86	156	ARG	C
87	163	LEU	C
88	164	LEU	C
89	165	GLY	C
90	172	GLN	C
91	173	ASN	C
92	174	LEU	C
93	175	PHE	C
94	176	HIS	B
95	192	LYS	B
96	193	ASN	B
97	194	LEU	B
98	195	TYR	B
99	197	LEU	B
100	198	ALA	B
101	199	ASP	B
102	200	LEU	B
103	201	ASN	B
104	202	HIS	B
105	203	ASN	B
106	204	ASN	B
107	205	SER	B
108	206	THR	B
109	207	VAL	B
110	208	ASP	B
111	209	VAL	B

Continued on next page

Table S48 – *Continued from previous page*

Homology index	Residue Index	Residue abbreviation	Module
112	210	TYR	B
113	211	LEU	B
114	212	LYS	B
115	213	ASP	B
116	214	ALA	B
117	215	ILE	B
118	216	LYS	B
119	217	MET	B
120	218	TRP	B
121	219	LEU	B
122	220	ASP	B
123	221	LEU	C
124	222	GLY	C
125	223	ILE	C
126	224	ASP	C
127	225	GLY	C
128	226	ILE	B
129	227	ARG	D
130	228	MET	B
131	229	ASP	B
132	230	ALA	B
133	231	VAL	B
134	232	LYS	B
135	233	HIS	B
136	234	MET	B
137	235	PRO	B
138	236	PHE	B
139	237	GLY	B
140	238	TRP	B
141	239	GLN	B
142	240	LYS	B
143	241	SER	B
144	242	PHE	B
145	243	MET	B
146	244	ALA	B
147	245	ALA	B
148	246	VAL	B
149	247	ASN	B
150	252	VAL	B
151	253	PHE	B
152	254	THR	B
153	255	PHE	C
154	256	GLY	B
155	257	GLN	B
156	258	TRP	B
157	259	PHE	B

Continued on next page

Table S48 – *Continued from previous page*

Homology index	Residue Index	Residue abbreviation	Module
158	260	LEU	B
159	261	GLY	B
160	269	ASN	B
161	270	HIS	B
162	271	LYS	B
163	272	PHE	B
164	274	ASN	B
165	275	GLU	B
166	277	GLY	B
167	278	MET	B
168	279	SER	B
169	280	LEU	B
170	281	LEU	D
171	282	ASP	D
172	283	PHE	D
173	284	ARG	D
174	286	ALA	D
175	287	GLN	D
176	288	LYS	D
177	289	VAL	D
178	290	ARG	D
179	291	GLN	D
180	292	VAL	D
181	293	PHE	D
182	298	ASP	D
183	299	ASN	A
184	300	MET	A
185	301	TYR	A
186	306	MET	A
187	307	LEU	D
188	308	GLU	D
189	309	GLY	D
190	310	SER	D
191	311	ALA	B
192	312	ALA	B
193	316	GLN	B
194	317	VAL	B
195	318	ASP	B
196	319	ASP	B
197	320	GLN	B
198	321	VAL	D
199	322	THR	D
200	323	PHE	D
201	324	ILE	A
202	325	ASP	D
203	326	ASN	D

Continued on next page

Table S48 – *Continued from previous page*

Homology index	Residue Index	Residue abbreviation	Module
204	327	HIS	D
205	328	ASP	D
206	329	MET	D
207	330	GLU	D
208	331	ARG	D
209	332	PHE	D
210	333	HIS	D
211	334	ALA	D
212	339	ARG	A
213	341	LYS	A
214	342	LEU	A
215	343	GLU	A
216	344	GLN	A
217	345	ALA	A
218	346	LEU	A
219	347	ALA	A
220	348	PHE	A
221	349	THR	A
222	350	LEU	A
223	351	THR	A
224	352	SER	A
225	353	ARG	D
226	354	GLY	D
227	355	VAL	D
228	356	PRO	A
229	357	ALA	D
230	358	ILE	A
231	359	TYR	D
232	360	TYR	C
233	361	GLY	D
234	362	THR	A
235	376	ALA	C
236	377	ARG	C
237	378	ILE	D
238	379	PRO	D
239	381	PHE	D
240	387	ALA	A
241	388	TYR	A
242	389	GLN	A
243	390	VAL	A
244	391	ILE	A
245	392	GLN	A
246	393	LYS	A
247	394	LEU	A
248	395	ALA	A
249	396	PRO	A

Continued on next page

Table S48 – *Continued from previous page*

Homology index	Residue Index	Residue abbreviation	Module
250	397	LEU	A
251	398	ARG	A
252	399	LYS	A
253	400	SER	A
254	401	ASN	A
255	402	PRO	A
256	406	TYR	A
257	407	GLY	A
258	408	SER	A
259	409	THR	A
260	411	GLU	A
261	412	ARG	A
262	413	TRP	A
263	414	ILE	A
264	415	ASN	A
265	416	ASN	A
266	417	ASP	A
267	418	VAL	A
268	419	LEU	A
269	420	ILE	A
270	421	TYR	A
271	422	GLU	A
272	423	ARG	A
273	428	ASN	A
274	429	VAL	A
275	430	ALA	A
276	431	VAL	A
277	432	VAL	A
278	433	ALA	A
279	434	VAL	A
280	435	ASN	A
281	439	ASN	A
282	440	ALA	A
283	441	PRO	A
284	442	ALA	A
285	443	SER	A
286	444	ILE	A
287	445	SER	A
288	454	GLY	A
289	455	SER	A
290	457	ASN	A
291	458	ASP	A
292	459	VAL	A
293	460	LEU	A
294	483	ALA	A
295	484	ALA	A

Continued on next page

Table S48 – *Continued from previous page*

Homology index	Residue Index	Residue abbreviation	Module
296	485	GLY	A
297	486	GLY	A
298	487	THR	A
299	488	ALA	A
300	489	VAL	A
301	490	TRP	A
302	491	GLN	A

Table S49: Residues membership for the *S. scrofa* α-amylase (PDB code 1DHK, chain A)

Homology index	Residue Index	Residue abbreviation	Module
0	11	THR	A
1	12	SER	A
2	13	ILE	B
3	14	VAL	B
4	15	HIS	B
5	16	LEU	C
6	17	PHE	C
7	18	GLU	C
8	19	TRP	C
9	20	ARG	C
10	21	TRP	C
11	22	VAL	C
12	23	ASP	C
13	24	ILE	C
14	25	ALA	C
15	26	LEU	C
16	27	GLU	C
17	28	CYS	C
18	29	GLU	C
19	30	ARG	C
20	32	LEU	C
21	33	GLY	C
22	34	PRO	C
23	35	LYS	C
24	36	GLY	C
25	37	PHE	C
26	38	GLY	C
27	39	GLY	C
28	40	VAL	C
29	41	GLN	C
30	42	VAL	C

Continued on next page

Table S49 – *Continued from previous page*

Homology index	Residue Index	Residue abbreviation	Module
31	43	SER	C
32	44	PRO	C
33	45	PRO	C
34	46	ASN	C
35	47	GLU	C
36	48	ASN	C
37	49	VAL	C
38	51	VAL	C
39	59	TRP	C
40	60	GLU	C
41	61	ARG	C
42	62	TYR	C
43	63	GLN	C
44	64	PRO	C
45	65	VAL	C
46	66	SER	C
47	67	TYR	C
48	68	LYS	C
49	69	LEU	C
50	70	CYS	C
51	71	THR	C
52	72	ARG	C
53	73	SER	C
54	74	GLY	C
55	75	ASN	C
56	76	GLU	C
57	77	ASN	C
58	78	GLU	C
59	79	PHE	C
60	80	ARG	C
61	81	ASP	C
62	82	MET	C
63	83	VAL	C
64	84	THR	C
65	85	ARG	C
66	86	CYS	C
67	87	ASN	C
68	88	ASN	C
69	89	VAL	C
70	90	GLY	C
71	91	VAL	C
72	92	ARG	C
73	93	ILE	C
74	94	TYR	C
75	95	VAL	C
76	96	ASP	C

Continued on next page

Table S49 – *Continued from previous page*

Homology index	Residue Index	Residue abbreviation	Module
77	97	ALA	C
78	98	VAL	C
79	99	ILE	C
80	100	ASN	C
81	101	HIS	C
82	102	MET	C
83	103	CYS	C
84	104	GLY	C
85	105	SER	C
86	126	PHE	C
87	130	PRO	C
88	131	TYR	C
89	132	SER	C
90	133	ALA	C
91	134	TRP	C
92	135	ASP	C
93	136	PHE	C
94	137	ASN	B
95	160	CYS	B
96	161	GLN	B
97	162	LEU	B
98	163	VAL	B
99	165	LEU	B
100	166	LEU	B
101	167	ASP	B
102	168	LEU	B
103	169	ALA	B
104	170	LEU	B
105	171	GLU	B
106	172	LYS	B
107	173	ASP	B
108	174	TYR	B
109	175	VAL	B
110	176	ARG	B
111	177	SER	B
112	178	MET	B
113	179	ILE	B
114	180	ALA	B
115	181	ASP	B
116	182	TYR	B
117	183	LEU	B
118	184	ASN	B
119	185	LYS	B
120	186	LEU	B
121	187	ILE	B
122	188	ASP	B

Continued on next page

Table S49 – *Continued from previous page*

Homology index	Residue Index	Residue abbreviation	Module
123	189	ILE	C
124	190	GLY	C
125	191	VAL	C
126	192	ALA	C
127	193	GLY	C
128	194	PHE	B
129	195	ARG	D
130	196	ILE	B
131	197	ASP	B
132	198	ALA	B
133	199	SER	B
134	200	LYS	B
135	201	HIS	B
136	202	MET	B
137	203	TRP	B
138	204	PRO	B
139	205	GLY	B
140	206	ASP	B
141	207	ILE	B
142	208	LYS	B
143	209	ALA	B
144	210	VAL	B
145	211	LEU	B
146	212	ASP	B
147	213	LYS	B
148	214	LEU	B
149	215	HIS	B
150	228	PRO	B
151	229	PHE	B
152	230	ILE	B
153	231	PHE	C
154	232	GLN	B
155	233	GLU	B
156	234	VAL	B
157	235	ILE	B
158	236	ASP	B
159	237	LEU	B
160	244	SER	B
161	245	SER	B
162	246	GLU	B
163	247	TYR	B
164	248	PHE	B
165	249	GLY	B
166	250	ASN	B
167	251	GLY	B
168	252	ARG	B

Continued on next page

Table S49 – *Continued from previous page*

Homology index	Residue Index	Residue abbreviation	Module
169	253	VAL	B
170	254	THR	D
171	255	GLU	D
172	256	PHE	D
173	257	LYS	D
174	259	GLY	D
175	260	ALA	D
176	261	LYS	D
177	262	LEU	D
178	263	GLY	D
179	264	THR	D
180	265	VAL	D
181	266	VAL	D
182	272	GLU	D
183	273	LYS	A
184	274	MET	A
185	275	SER	A
186	280	TRP	A
187	281	GLY	D
188	282	GLU	D
189	283	GLY	D
190	285	GLY	D
191	286	PHE	B
192	287	MET	B
193	288	PRO	B
194	289	SER	B
195	290	ASP	B
196	291	ARG	B
197	292	ALA	B
198	293	LEU	D
199	294	VAL	D
200	295	PHE	D
201	296	VAL	A
202	297	ASP	D
203	298	ASN	D
204	299	HIS	D
205	300	ASP	D
206	301	ASN	D
207	302	GLN	D
208	314	THR	D
209	315	PHE	D
210	316	TRP	D
211	317	ASP	D
212	319	ARG	A
213	320	LEU	A
214	321	TYR	A

Continued on next page

Table S49 – *Continued from previous page*

Homology index	Residue Index	Residue abbreviation	Module
215	322	LYS	A
216	323	VAL	A
217	324	ALA	A
218	325	VAL	A
219	326	GLY	A
220	327	PHE	A
221	328	MET	A
222	329	LEU	A
223	330	ALA	A
224	331	HIS	A
225	333	TYR	D
226	334	GLY	D
227	335	PHE	D
228	336	THR	A
229	337	ARG	D
230	338	VAL	A
231	339	MET	D
232	340	SER	C
233	341	SER	D
234	342	TYR	A
235	343	ARG	C
236	381	ASP	C
237	382	TRP	D
238	383	VAL	D
239	384	CYS	D
240	387	ARG	A
241	388	TRP	A
242	389	ARG	A
243	390	GLU	A
244	391	ILE	A
245	392	ARG	A
246	393	ASN	A
247	394	MET	A
248	395	VAL	A
249	396	TRP	A
250	397	PHE	A
251	398	ARG	A
252	399	ASN	A
253	400	VAL	A
254	401	VAL	A
255	402	ASP	A
256	403	GLY	A
257	404	GLN	A
258	405	PRO	A
259	406	PHE	A
260	408	ASN	A

Continued on next page

Table S49 – *Continued from previous page*

Homology index	Residue Index	Residue abbreviation	Module
261	409	TRP	A
262	410	TRP	A
263	411	ASP	A
264	413	GLY	A
265	414	SER	A
266	415	ASN	A
267	416	GLN	A
268	417	VAL	A
269	418	ALA	A
270	419	PHE	A
271	420	GLY	A
272	421	ARG	A
273	422	GLY	A
274	423	ASN	A
275	425	GLY	A
276	426	PHE	A
277	427	ILE	A
278	428	VAL	A
279	429	PHE	A
280	430	ASN	A
281	433	ASP	A
282	434	TRP	A
283	435	GLN	A
284	436	LEU	A
285	437	SER	A
286	438	SER	A
287	439	THR	A
288	445	PRO	A
289	446	GLY	A
290	449	TYR	A
291	450	CYS	A
292	451	ASP	A
293	452	VAL	A
294	479	ILE	A
295	480	SER	A
296	481	ASN	A
297	486	PRO	A
298	487	PHE	A
299	488	ILE	A
300	489	ALA	A
301	490	ILE	A
302	491	HIS	A

Table S50: Residues membership for the *B. circulans* α-amylase (PDB code 1D3C, chain A)

Homology index	Residue Index	Residue abbreviation	Module
0	15	ASP	A
1	16	VAL	A
2	17	ILE	B
3	18	TYR	B
4	19	GLN	B
5	20	ILE	C
6	21	PHE	C
7	22	THR	C
8	52	GLY	C
9	53	ASP	C
10	54	TRP	C
11	55	GLN	C
12	56	GLY	C
13	57	ILE	C
14	58	ILE	C
15	59	ASN	C
16	60	LYS	C
17	61	ILE	C
18	62	ASN	C
19	63	ASP	C
20	66	LEU	C
21	67	THR	C
22	68	GLY	C
23	69	MET	C
24	70	GLY	C
25	71	VAL	C
26	72	THR	C
27	73	ALA	C
28	74	ILE	C
29	75	TRP	C
30	76	ILE	C
31	77	SER	C
32	78	GLN	C
33	79	PRO	C
34	80	VAL	C
35	81	GLU	C
36	82	ASN	C
37	83	ILE	C
38	95	THR	C
39	97	TYR	C
40	98	HIS	C
41	99	GLY	C
42	100	TYR	C
43	101	TRP	C

Continued on next page

Table S50 – *Continued from previous page*

Homology index	Residue Index	Residue abbreviation	Module
44	102	ALA	C
45	103	ARG	C
46	104	ASP	C
47	106	LYS	C
48	107	LYS	C
49	108	THR	C
50	109	ASN	C
51	110	PRO	C
52	111	ALA	C
53	112	TYR	C
54	113	GLY	C
55	114	THR	C
56	115	ILE	C
57	116	ALA	C
58	117	ASP	C
59	118	PHE	C
60	119	GLN	C
61	120	ASN	C
62	121	LEU	C
63	122	ILE	C
64	123	ALA	C
65	124	ALA	C
66	125	ALA	C
67	126	HIS	C
68	127	ALA	C
69	128	LYS	C
70	129	ASN	C
71	130	ILE	C
72	131	LYS	C
73	132	VAL	C
74	133	ILE	C
75	134	ILE	C
76	135	ASP	C
77	136	PHE	C
78	137	ALA	C
79	138	PRO	C
80	139	ASN	C
81	140	HIS	C
82	141	THR	C
83	142	SER	C
84	143	PRO	C
85	144	ALA	C
86	156	ARG	C
87	163	LEU	C
88	164	LEU	C
89	165	GLY	C

Continued on next page

Table S50 – *Continued from previous page*

Homology index	Residue Index	Residue abbreviation	Module
90	172	GLN	C
91	173	ASN	C
92	174	LEU	C
93	175	PHE	C
94	176	HIS	B
95	192	LYS	B
96	193	ASN	B
97	194	LEU	B
98	195	TYR	B
99	197	LEU	B
100	198	ALA	B
101	199	ASP	B
102	200	LEU	B
103	201	ASN	B
104	202	HIS	B
105	203	ASN	B
106	204	ASN	B
107	205	SER	B
108	206	THR	B
109	207	VAL	B
110	208	ASP	B
111	209	VAL	B
112	210	TYR	B
113	211	LEU	B
114	212	LYS	B
115	213	ASP	B
116	214	ALA	B
117	215	ILE	B
118	216	LYS	B
119	217	MET	B
120	218	TRP	B
121	219	LEU	B
122	220	ASP	B
123	221	LEU	C
124	222	GLY	C
125	223	ILE	C
126	224	ASP	C
127	225	GLY	C
128	226	ILE	B
129	227	ARG	D
130	228	MET	B
131	229	ASN	B
132	230	ALA	B
133	231	VAL	B
134	232	LYS	B
135	233	HIS	B

Continued on next page

Table S50 – *Continued from previous page*

Homology index	Residue Index	Residue abbreviation	Module
136	234	MET	B
137	235	PRO	B
138	236	PHE	B
139	237	GLY	B
140	238	TRP	B
141	239	GLN	B
142	240	LYS	B
143	241	SER	B
144	242	PHE	B
145	243	MET	B
146	244	ALA	B
147	245	ALA	B
148	246	VAL	B
149	247	ASN	B
150	252	VAL	B
151	253	PHE	B
152	254	THR	B
153	255	PHE	C
154	256	GLY	B
155	257	GLN	B
156	258	TRP	B
157	259	PHE	B
158	260	LEU	B
159	261	GLY	B
160	269	ASN	B
161	270	HIS	B
162	271	LYS	B
163	272	PHE	B
164	274	ASN	B
165	275	GLU	B
166	277	GLY	B
167	278	MET	B
168	279	SER	B
169	280	LEU	B
170	281	LEU	D
171	282	ASP	D
172	283	PHE	D
173	284	ARG	D
174	286	ALA	D
175	287	GLN	D
176	288	LYS	D
177	289	VAL	D
178	290	ARG	D
179	291	GLN	D
180	292	VAL	D
181	293	PHE	D

Continued on next page

Table S50 – *Continued from previous page*

Homology index	Residue Index	Residue abbreviation	Module
182	298	ASP	D
183	299	ASN	A
184	300	MET	A
185	301	TYR	A
186	306	MET	A
187	307	LEU	D
188	308	GLU	D
189	309	GLY	D
190	310	SER	D
191	311	ALA	B
192	312	ALA	B
193	316	GLN	B
194	317	VAL	B
195	318	ASP	B
196	319	ASP	B
197	320	GLN	B
198	321	VAL	D
199	322	THR	D
200	323	PHE	D
201	324	ILE	A
202	325	ASP	D
203	326	ASN	D
204	327	HIS	D
205	328	ASP	D
206	329	MET	D
207	330	GLU	D
208	331	ARG	D
209	332	PHE	D
210	333	HIS	D
211	334	ALA	D
212	339	ARG	A
213	341	LYS	A
214	342	LEU	A
215	343	GLU	A
216	344	GLN	A
217	345	ALA	A
218	346	LEU	A
219	347	ALA	A
220	348	PHE	A
221	349	THR	A
222	350	LEU	A
223	351	THR	A
224	352	SER	A
225	353	ARG	D
226	354	GLY	D
227	355	VAL	D

Continued on next page

Table S50 – *Continued from previous page*

Homology index	Residue Index	Residue abbreviation	Module
228	356	PRO	A
229	357	ALA	D
230	358	ILE	A
231	359	TYR	D
232	360	TYR	C
233	361	GLY	D
234	362	THR	A
235	376	ALA	C
236	377	ARG	C
237	378	ILE	D
238	379	PRO	D
239	381	PHE	D
240	387	ALA	A
241	388	TYR	A
242	389	GLN	A
243	390	VAL	A
244	391	ILE	A
245	392	GLN	A
246	393	LYS	A
247	394	LEU	A
248	395	ALA	A
249	396	PRO	A
250	397	LEU	A
251	398	ARG	A
252	399	LYS	A
253	400	CYS	A
254	401	ASN	A
255	402	PRO	A
256	406	TYR	A
257	407	GLY	A
258	408	SER	A
259	409	THR	A
260	411	GLU	A
261	412	ARG	A
262	413	TRP	A
263	414	ILE	A
264	415	ASN	A
265	416	ASN	A
266	417	ASP	A
267	418	VAL	A
268	419	LEU	A
269	420	ILE	A
270	421	TYR	A
271	422	GLU	A
272	423	ARG	A
273	428	ASN	A

Continued on next page

Table S50 – *Continued from previous page*

Homology index	Residue Index	Residue abbreviation	Module
274	429	VAL	A
275	430	ALA	A
276	431	VAL	A
277	432	VAL	A
278	433	ALA	A
279	434	VAL	A
280	435	ASN	A
281	439	ASN	A
282	440	ALA	A
283	441	PRO	A
284	442	ALA	A
285	443	SER	A
286	444	ILE	A
287	445	SER	A
288	454	GLY	A
289	455	SER	A
290	457	ASN	A
291	458	ASP	A
292	459	VAL	A
293	460	LEU	A
294	483	ALA	A
295	484	ALA	A
296	485	GLY	A
297	486	GLY	A
298	487	THR	A
299	488	ALA	A
300	489	VAL	A
301	490	TRP	A
302	491	GLN	A

Table S51: Residues membership for the *S. solfataricus KM1* α-amylase (PDB code 1EHA, chain A)

Homology index	Residue Index	Residue abbreviation	Module
0	102	LEU	A
1	103	ILE	A
2	104	ILE	B
3	105	TYR	B
4	106	GLU	B
5	107	ILE	C
6	108	HIS	C
7	109	VAL	C
8	116	GLY	C

Continued on next page

Table S51 – *Continued from previous page*

Homology index	Residue Index	Residue abbreviation	Module
9	117	THR	C
10	118	PHE	C
11	119	GLU	C
12	120	GLY	C
13	121	VAL	C
14	122	ILE	C
15	123	ARG	C
16	124	LYS	C
17	125	LEU	C
18	126	ASP	C
19	127	TYR	C
20	128	LEU	C
21	129	LYS	C
22	130	ASP	C
23	131	LEU	C
24	132	GLY	C
25	133	ILE	C
26	134	THR	C
27	135	ALA	C
28	136	ILE	C
29	137	GLU	C
30	138	ILE	C
31	139	MET	C
32	140	PRO	C
33	141	ILE	C
34	142	ALA	C
35	143	GLN	C
36	144	PHE	C
37	145	PRO	C
38	146	GLY	C
39	149	ASP	C
40	150	TRP	C
41	151	GLY	C
42	152	TYR	C
43	153	ASP	C
44	154	GLY	C
45	155	VAL	C
46	156	TYR	C
47	158	TYR	C
48	159	ALA	C
49	160	VAL	C
50	161	GLN	C
51	162	ASN	C
52	163	SER	C
53	164	TYR	C
54	165	GLY	C

Continued on next page

Table S51 – *Continued from previous page*

Homology index	Residue Index	Residue abbreviation	Module
55	166	GLY	C
56	167	PRO	C
57	168	GLU	C
58	169	GLY	C
59	170	PHE	C
60	171	ARG	C
61	172	LYS	C
62	173	LEU	C
63	174	VAL	C
64	175	ASP	C
65	176	GLU	C
66	177	ALA	C
67	178	HIS	C
68	179	LYS	C
69	180	LYS	C
70	181	GLY	C
71	182	LEU	C
72	183	GLY	C
73	184	VAL	C
74	185	ILE	C
75	186	LEU	C
76	187	ASP	C
77	188	VAL	C
78	189	VAL	C
79	190	TYR	C
80	191	ASN	C
81	192	HIS	C
82	193	VAL	C
83	194	GLY	C
84	195	PRO	C
85	196	GLU	C
86	198	ASN	C
87	199	TYR	C
88	200	MET	C
89	201	VAL	C
90	204	GLY	C
91	205	PRO	C
92	206	TYR	C
93	207	PHE	C
94	208	SER	B
95	212	LYS	B
96	213	THR	B
97	214	PRO	B
98	215	TRP	B
99	216	GLY	B
100	217	LEU	B

Continued on next page

Table S51 – *Continued from previous page*

Homology index	Residue Index	Residue abbreviation	Module
101	218	THR	B
102	219	PHE	B
103	220	ASN	B
104	221	PHE	B
105	222	ASP	B
106	226	SER	B
107	227	ASP	B
108	228	GLU	B
109	229	VAL	B
110	230	ARG	B
111	231	LYS	B
112	232	PHE	B
113	233	ILE	B
114	234	LEU	B
115	235	GLU	B
116	236	ASN	B
117	237	VAL	B
118	238	GLU	B
119	239	TYR	B
120	240	TRP	B
121	241	ILE	B
122	242	LYS	B
123	244	TYR	C
124	245	ASN	C
125	246	VAL	C
126	247	ASP	C
127	248	GLY	C
128	249	PHE	B
129	250	ARG	D
130	251	LEU	B
131	252	ASP	B
132	253	ALA	B
133	254	VAL	B
134	255	HIS	B
135	256	ALA	B
136	257	ILE	B
137	258	ILE	B
138	263	LYS	B
139	264	HIS	B
140	265	ILE	B
141	266	LEU	B
142	267	GLU	B
143	268	GLU	B
144	269	ILE	B
145	270	ALA	B
146	271	ASP	B

Continued on next page

Table S51 – *Continued from previous page*

Homology index	Residue Index	Residue abbreviation	Module
147	272	VAL	B
148	273	VAL	B
149	274	HIS	B
150	278	ARG	B
151	279	ILE	B
152	280	VAL	B
153	281	ILE	C
154	282	ALA	B
155	283	GLU	B
156	284	SER	B
157	285	ASP	B
158	286	LEU	B
159	287	ASN	B
160	288	ASP	B
161	289	PRO	B
162	290	ARG	B
163	291	VAL	B
164	292	VAL	B
165	293	ASN	B
166	302	ILE	B
167	303	ASP	B
168	304	ALA	B
169	305	GLN	B
170	306	TRP	D
171	307	VAL	D
172	308	ASP	D
173	309	ASP	D
174	311	HIS	D
175	312	HIS	D
176	313	SER	D
177	314	ILE	D
178	315	HIS	D
179	316	ALA	D
180	317	TYR	D
181	318	LEU	D
182	330	GLY	D
183	331	ASN	A
184	332	LEU	A
185	333	ASP	A
186	338	SER	A
187	339	TYR	D
188	340	LYS	D
189	341	ASP	D
190	342	VAL	D
191	343	PHE	B
192	344	VAL	B

Continued on next page

Table S51 – *Continued from previous page*

Homology index	Residue Index	Residue abbreviation	Module
193	365	ASP	B
194	366	GLY	B
195	367	CYS	B
196	368	ASN	B
197	369	PHE	B
198	370	VAL	D
199	371	VAL	D
200	372	TYR	D
201	373	ILE	A
202	374	GLN	D
203	375	ASN	D
204	376	HIS	D
205	377	ASP	D
206	378	GLN	D
207	379	VAL	D
208	387	ARG	D
209	388	ILE	D
210	389	ILE	D
211	390	LYS	D
212	394	ARG	A
213	396	SER	A
214	397	TYR	A
215	398	LYS	A
216	399	ILE	A
217	400	ALA	A
218	401	ALA	A
219	402	ALA	A
220	403	LEU	A
221	404	TYR	A
222	405	LEU	A
223	406	LEU	A
224	407	SER	A
225	408	PRO	D
226	409	TYR	D
227	410	ILE	D
228	411	PRO	A
229	412	MET	D
230	413	ILE	A
231	414	PHE	D
232	415	MET	C
233	416	GLY	D
234	417	GLU	A
235	463	SER	C
236	464	LYS	C
237	465	LEU	D
238	466	SER	D

Continued on next page

Table S51 – *Continued from previous page*

Homology index	Residue Index	Residue abbreviation	Module
239	467	TRP	D
240	473	ILE	A
241	474	PHE	A
242	475	SER	A
243	476	PHE	A
244	477	TYR	A
245	478	LYS	A
246	479	ILE	A
247	480	LEU	A
248	481	ILE	A
249	482	LYS	A
250	483	MET	A
251	484	ARG	A
252	485	LYS	A
253	486	GLU	A
254	487	LEU	A
255	488	SER	A
256	492	ASP	A
257	493	ARG	A
258	494	ARG	A
259	495	VAL	A
260	496	ASN	A
261	497	VAL	A
262	498	VAL	A
263	499	ASN	A
264	500	GLY	A
265	501	GLU	A
266	502	ASN	A
267	503	TRP	A
268	504	LEU	A
269	505	ILE	A
270	506	ILE	A
271	507	LYS	A
272	508	GLY	A
273	509	ARG	A
274	510	GLU	A
275	511	TYR	A
276	512	PHE	A
277	513	SER	A
278	514	LEU	A
279	515	TYR	A
280	516	VAL	A
281	518	SER	A
282	519	LYS	A
283	520	SER	A
284	521	SER	A

Continued on next page

Table S51 – *Continued from previous page*

Homology index	Residue Index	Residue abbreviation	Module
285	522	ILE	A
286	523	GLU	A
287	524	VAL	A
288	527	SER	A
289	528	GLY	A
290	529	THR	A
291	530	LEU	A
292	531	LEU	A
293	532	LEU	A
294	549	PHE	A
295	550	ASP	A
296	551	LYS	A
297	552	GLY	A
298	553	PHE	A
299	554	ALA	A
300	555	LEU	A
301	556	TYR	A
302	557	LYS	A

Table S52: Residues membership for the *S. solfataricus KM1* α-amylase (PDB code 1EH9, chain A)

Homology index	Residue Index	Residue abbreviation	Module
0	102	LEU	A
1	103	ILE	A
2	104	ILE	B
3	105	TYR	B
4	106	GLU	B
5	107	ILE	C
6	108	HIS	C
7	109	VAL	C
8	116	GLY	C
9	117	THR	C
10	118	PHE	C
11	119	GLU	C
12	120	GLY	C
13	121	VAL	C
14	122	ILE	C
15	123	ARG	C
16	124	LYS	C
17	125	LEU	C
18	126	ASP	C
19	127	TYR	C

Continued on next page

Table S52 – *Continued from previous page*

Homology index	Residue Index	Residue abbreviation	Module
20	128	LEU	C
21	129	LYS	C
22	130	ASP	C
23	131	LEU	C
24	132	GLY	C
25	133	ILE	C
26	134	THR	C
27	135	ALA	C
28	136	ILE	C
29	137	GLU	C
30	138	ILE	C
31	139	MET	C
32	140	PRO	C
33	141	ILE	C
34	142	ALA	C
35	143	GLN	C
36	144	PHE	C
37	145	PRO	C
38	146	GLY	C
39	149	ASP	C
40	150	TRP	C
41	151	GLY	C
42	152	TYR	C
43	153	ASP	C
44	154	GLY	C
45	155	VAL	C
46	156	TYR	C
47	158	TYR	C
48	159	ALA	C
49	160	VAL	C
50	161	GLN	C
51	162	ASN	C
52	163	SER	C
53	164	TYR	C
54	165	GLY	C
55	166	GLY	C
56	167	PRO	C
57	168	GLU	C
58	169	GLY	C
59	170	PHE	C
60	171	ARG	C
61	172	LYS	C
62	173	LEU	C
63	174	VAL	C
64	175	ASP	C
65	176	GLU	C

Continued on next page

Table S52 – *Continued from previous page*

Homology index	Residue Index	Residue abbreviation	Module
66	177	ALA	C
67	178	HIS	C
68	179	LYS	C
69	180	LYS	C
70	181	GLY	C
71	182	LEU	C
72	183	GLY	C
73	184	VAL	C
74	185	ILE	C
75	186	LEU	C
76	187	ASP	C
77	188	VAL	C
78	189	VAL	C
79	190	TYR	C
80	191	ASN	C
81	192	HIS	C
82	193	VAL	C
83	194	GLY	C
84	195	PRO	C
85	196	GLU	C
86	198	ASN	C
87	199	TYR	C
88	200	MET	C
89	201	VAL	C
90	204	GLY	C
91	205	PRO	C
92	206	TYR	C
93	207	PHE	C
94	208	SER	B
95	212	LYS	B
96	213	THR	B
97	214	PRO	B
98	215	TRP	B
99	216	GLY	B
100	217	LEU	B
101	218	THR	B
102	219	PHE	B
103	220	ASN	B
104	221	PHE	B
105	222	ASP	B
106	226	SER	B
107	227	ASP	B
108	228	GLU	B
109	229	VAL	B
110	230	ARG	B
111	231	LYS	B

Continued on next page

Table S52 – *Continued from previous page*

Homology index	Residue Index	Residue abbreviation	Module
112	232	PHE	B
113	233	ILE	B
114	234	LEU	B
115	235	GLU	B
116	236	ASN	B
117	237	VAL	B
118	238	GLU	B
119	239	TYR	B
120	240	TRP	B
121	241	ILE	B
122	242	LYS	B
123	244	TYR	C
124	245	ASN	C
125	246	VAL	C
126	247	ASP	C
127	248	GLY	C
128	249	PHE	B
129	250	ARG	D
130	251	LEU	B
131	252	ASP	B
132	253	ALA	B
133	254	VAL	B
134	255	HIS	B
135	256	ALA	B
136	257	ILE	B
137	258	ILE	B
138	263	LYS	B
139	264	HIS	B
140	265	ILE	B
141	266	LEU	B
142	267	GLU	B
143	268	GLU	B
144	269	ILE	B
145	270	ALA	B
146	271	ASP	B
147	272	VAL	B
148	273	VAL	B
149	274	HIS	B
150	278	ARG	B
151	279	ILE	B
152	280	VAL	B
153	281	ILE	C
154	282	ALA	B
155	283	GLU	B
156	284	SER	B
157	285	ASP	B

Continued on next page

Table S52 – *Continued from previous page*

Homology index	Residue Index	Residue abbreviation	Module
158	286	LEU	B
159	287	ASN	B
160	288	ASP	B
161	289	PRO	B
162	290	ARG	B
163	291	VAL	B
164	292	VAL	B
165	293	ASN	B
166	302	ILE	B
167	303	ASP	B
168	304	ALA	B
169	305	GLN	B
170	306	TRP	D
171	307	VAL	D
172	308	ASP	D
173	309	ASP	D
174	311	HIS	D
175	312	HIS	D
176	313	SER	D
177	314	ILE	D
178	315	HIS	D
179	316	ALA	D
180	317	TYR	D
181	318	LEU	D
182	330	GLY	D
183	331	ASN	A
184	332	LEU	A
185	333	ASP	A
186	338	SER	A
187	339	TYR	D
188	340	LYS	D
189	341	ASP	D
190	342	VAL	D
191	343	PHE	B
192	344	VAL	B
193	365	ASP	B
194	366	GLY	B
195	367	CYS	B
196	368	ASN	B
197	369	PHE	B
198	370	VAL	D
199	371	VAL	D
200	372	TYR	D
201	373	ILE	A
202	374	GLN	D
203	375	ASN	D

Continued on next page

Table S52 – *Continued from previous page*

Homology index	Residue Index	Residue abbreviation	Module
204	376	HIS	D
205	377	ASP	D
206	378	GLN	D
207	379	VAL	D
208	387	ARG	D
209	388	ILE	D
210	389	ILE	D
211	390	LYS	D
212	394	ARG	A
213	396	SER	A
214	397	TYR	A
215	398	LYS	A
216	399	ILE	A
217	400	ALA	A
218	401	ALA	A
219	402	ALA	A
220	403	LEU	A
221	404	TYR	A
222	405	LEU	A
223	406	LEU	A
224	407	SER	A
225	408	PRO	D
226	409	TYR	D
227	410	ILE	D
228	411	PRO	A
229	412	MET	D
230	413	ILE	A
231	414	PHE	D
232	415	MET	C
233	416	GLY	D
234	417	GLU	A
235	463	SER	C
236	464	LYS	C
237	465	LEU	D
238	466	SER	D
239	467	TRP	D
240	473	ILE	A
241	474	PHE	A
242	475	SER	A
243	476	PHE	A
244	477	TYR	A
245	478	LYS	A
246	479	ILE	A
247	480	LEU	A
248	481	ILE	A
249	482	LYS	A

Continued on next page

Table S52 – *Continued from previous page*

Homology index	Residue Index	Residue abbreviation	Module
250	483	MET	A
251	484	ARG	A
252	485	LYS	A
253	486	GLU	A
254	487	LEU	A
255	488	SER	A
256	492	ASP	A
257	493	ARG	A
258	494	ARG	A
259	495	VAL	A
260	496	ASN	A
261	497	VAL	A
262	498	VAL	A
263	499	ASN	A
264	500	GLY	A
265	501	GLU	A
266	502	ASN	A
267	503	TRP	A
268	504	LEU	A
269	505	ILE	A
270	506	ILE	A
271	507	LYS	A
272	508	GLY	A
273	509	ARG	A
274	510	GLU	A
275	511	TYR	A
276	512	PHE	A
277	513	SER	A
278	514	LEU	A
279	515	TYR	A
280	516	VAL	A
281	518	SER	A
282	519	LYS	A
283	520	SER	A
284	521	SER	A
285	522	ILE	A
286	523	GLU	A
287	524	VAL	A
288	527	SER	A
289	528	GLY	A
290	529	THR	A
291	530	LEU	A
292	531	LEU	A
293	532	LEU	A
294	549	PHE	A
295	550	ASP	A

Continued on next page

Table S52 – *Continued from previous page*

Homology index	Residue Index	Residue abbreviation	Module
296	551	LYS	A
297	552	GLY	A
298	553	PHE	A
299	554	ALA	A
300	555	LEU	A
301	556	TYR	A
302	557	LYS	A

Table S53: Residues membership for the *T. vulgaris R47* α-amylase (PDB code 1VFO, chain A)

Homology index	Residue Index	Residue abbreviation	Module
0	131	ALA	A
1	132	VAL	A
2	133	ILE	B
3	134	TYR	B
4	135	GLN	B
5	136	ILE	C
6	137	PHE	C
7	138	PRO	C
8	170	GLY	C
9	171	ASP	C
10	172	LEU	C
11	173	LYS	C
12	174	GLY	C
13	175	VAL	C
14	176	ILE	C
15	177	ASP	C
16	178	ARG	C
17	179	LEU	C
18	180	PRO	C
19	181	TYR	C
20	182	LEU	C
21	183	GLU	C
22	184	GLU	C
23	185	LEU	C
24	186	GLY	C
25	187	VAL	C
26	188	THR	C
27	189	ALA	C
28	190	LEU	C
29	191	TYR	C
30	192	PHE	C

Continued on next page

Table S53 – *Continued from previous page*

Homology index	Residue Index	Residue abbreviation	Module
31	193	THR	C
32	194	PRO	C
33	195	ILE	C
34	196	PHE	C
35	197	ALA	C
36	198	SER	C
37	199	PRO	C
38	200	SER	C
39	201	HIS	C
40	202	HIS	C
41	203	LYS	C
42	204	TYR	C
43	205	ASP	C
44	206	THR	C
45	207	ALA	C
46	208	ASP	C
47	210	LEU	C
48	211	ALA	C
49	212	ILE	C
50	213	ASP	C
51	214	PRO	C
52	215	GLN	C
53	216	PHE	C
54	217	GLY	C
55	218	ASP	C
56	219	LEU	C
57	220	PRO	C
58	221	THR	C
59	222	PHE	C
60	223	ARG	C
61	224	ARG	C
62	225	LEU	C
63	226	VAL	C
64	227	ASP	C
65	228	GLU	C
66	229	ALA	C
67	230	HIS	C
68	231	ARG	C
69	232	ARG	C
70	233	GLY	C
71	234	ILE	C
72	235	LYS	C
73	236	ILE	C
74	237	ILE	C
75	238	LEU	C
76	239	ASP	C

Continued on next page

Table S53 – *Continued from previous page*

Homology index	Residue Index	Residue abbreviation	Module
77	240	ALA	C
78	241	VAL	C
79	242	PHE	C
80	243	ASN	C
81	244	HIS	C
82	245	ALA	C
83	246	GLY	C
84	247	ASP	C
85	248	GLN	C
86	249	PHE	C
87	250	PHE	C
88	251	ALA	C
89	252	PHE	C
90	265	LYS	C
91	266	ASP	C
92	267	TRP	C
93	268	PHE	C
94	269	PHE	B
95	284	GLU	B
96	285	THR	B
97	286	PHE	B
98	287	ALA	B
99	293	MET	B
100	294	PRO	B
101	295	LYS	B
102	296	LEU	B
103	297	ARG	B
104	298	THR	B
105	299	GLU	B
106	300	ASN	B
107	301	PRO	B
108	302	GLU	B
109	303	VAL	B
110	304	LYS	B
111	305	GLU	B
112	306	TYR	B
113	307	LEU	B
114	308	PHE	B
115	309	ASP	B
116	310	VAL	B
117	311	ALA	B
118	312	ARG	B
119	313	PHE	B
120	314	TRP	B
121	315	MET	B
122	316	GLU	B

Continued on next page

Table S53 – *Continued from previous page*

Homology index	Residue Index	Residue abbreviation	Module
123	317	GLN	C
124	318	GLY	C
125	319	ILE	C
126	320	ASP	C
127	321	GLY	C
128	322	TRP	B
129	323	ARG	D
130	324	LEU	B
131	325	ASN	B
132	326	VAL	B
133	327	ALA	B
134	328	ASN	B
135	329	GLU	B
136	330	VAL	B
137	331	ASP	B
138	332	HIS	B
139	333	ALA	B
140	334	PHE	B
141	335	TRP	B
142	336	ARG	B
143	337	GLU	B
144	338	PHE	B
145	339	ARG	B
146	340	ARG	B
147	341	LEU	B
148	342	VAL	B
149	343	LYS	B
150	349	ALA	B
151	350	LEU	B
152	351	ILE	B
153	352	VAL	C
154	353	GLY	B
155	354	GLU	B
156	355	ILE	B
157	356	TRP	B
158	357	HIS	B
159	358	ASP	B
160	359	ALA	B
161	360	SER	B
162	361	GLY	B
163	362	TRP	B
164	363	LEU	B
165	364	MET	B
166	368	PHE	B
167	369	ASP	B
168	370	SER	B

Continued on next page

Table S53 – *Continued from previous page*

Homology index	Residue Index	Residue abbreviation	Module
169	371	VAL	B
170	372	MET	D
171	373	ASN	D
172	374	TYR	D
173	375	LEU	D
174	377	ARG	D
175	378	GLU	D
176	379	SER	D
177	380	VAL	D
178	381	ILE	D
179	382	ARG	D
180	383	PHE	D
181	384	PHE	D
182	389	ILE	D
183	390	HIS	A
184	391	ALA	A
185	392	GLU	A
186	397	GLU	A
187	398	LEU	D
188	399	THR	D
189	400	ARG	D
190	401	ALA	D
191	402	ARG	B
192	403	MET	B
193	409	ALA	B
194	410	ALA	B
195	411	GLN	B
196	412	GLY	B
197	413	LEU	B
198	414	TRP	D
199	415	ASN	D
200	416	LEU	D
201	417	LEU	A
202	418	ASP	D
203	419	SER	D
204	420	HIS	D
205	421	ASN	D
206	422	THR	D
207	423	GLU	D
208	424	ARG	D
209	425	PHE	D
210	426	LEU	D
211	427	THR	D
212	433	GLU	A
213	435	LYS	A
214	436	PHE	A

Continued on next page

Table S53 – *Continued from previous page*

Homology index	Residue Index	Residue abbreviation	Module
215	437	ARG	A
216	438	LEU	A
217	439	ALA	A
218	440	VAL	A
219	441	LEU	A
220	442	PHE	A
221	443	GLN	A
222	444	MET	A
223	445	THR	A
224	446	TYR	A
225	447	LEU	D
226	448	GLY	D
227	449	THR	D
228	450	PRO	A
229	451	LEU	D
230	452	ILE	A
231	453	TYR	D
232	454	TYR	C
233	455	GLY	D
234	456	ASP	A
235	470	ARG	C
236	471	PRO	C
237	472	MET	D
238	473	ILE	D
239	474	TRP	D
240	483	LEU	A
241	484	PHE	A
242	485	GLU	A
243	486	PHE	A
244	487	TYR	A
245	488	LYS	A
246	489	GLU	A
247	490	LEU	A
248	491	ILE	A
249	492	ARG	A
250	493	LEU	A
251	494	ARG	A
252	495	HIS	A
253	496	ARG	A
254	497	LEU	A
255	498	ALA	A
256	502	ARG	A
257	503	GLY	A
258	504	ASN	A
259	505	VAL	A
260	507	SER	A

Continued on next page

Table S53 – *Continued from previous page*

Homology index	Residue Index	Residue abbreviation	Module
261	508	TRP	A
262	509	HIS	A
263	510	ALA	A
264	513	GLN	A
265	514	ALA	A
266	515	ASN	A
267	516	LEU	A
268	517	TYR	A
269	518	ALA	A
270	519	PHE	A
271	520	VAL	A
272	521	ARG	A
273	526	GLN	A
274	527	HIS	A
275	528	VAL	A
276	529	GLY	A
277	530	VAL	A
278	531	VAL	A
279	532	LEU	A
280	533	ASN	A
281	536	GLY	A
282	537	GLU	A
283	538	LYS	A
284	539	GLN	A
285	540	THR	A
286	541	VAL	A
287	542	LEU	A
288	552	THR	A
289	553	TRP	A
290	554	LEU	A
291	555	ASP	A
292	556	CYS	A
293	557	LEU	A
294	574	ARG	A
295	575	PRO	A
296	576	TYR	A
297	577	GLN	A
298	578	GLY	A
299	579	MET	A
300	580	ILE	A
301	581	LEU	A
302	582	TRP	A

Table S54: Residues membership for the *N. polysaccharea* α-amylase (PDB code 1G5A, chain A)

Homology index	Residue Index	Residue abbreviation	Module
0	98	GLN	A
1	99	VAL	A
2	100	GLY	B
3	101	GLY	B
4	102	VAL	B
5	103	CYS	C
6	104	TYR	C
7	105	VAL	C
8	110	GLY	C
9	111	ASP	C
10	112	LEU	C
11	113	LYS	C
12	114	GLY	C
13	115	LEU	C
14	116	LYS	C
15	117	ASP	C
16	118	LYS	C
17	119	ILE	C
18	120	PRO	C
19	121	TYR	C
20	122	PHE	C
21	123	GLN	C
22	124	GLU	C
23	125	LEU	C
24	126	GLY	C
25	127	LEU	C
26	128	THR	C
27	129	TYR	C
28	130	LEU	C
29	131	HIS	C
30	132	LEU	C
31	133	MET	C
32	134	PRO	C
33	135	LEU	C
34	136	PHE	C
35	137	LYS	C
36	138	CYS	C
37	139	PRO	C
38	141	GLY	C
39	144	ASP	C
40	145	GLY	C
41	146	GLY	C
42	147	TYR	C
43	148	ALA	C

Continued on next page

Table S54 – *Continued from previous page*

Homology index	Residue Index	Residue abbreviation	Module
44	149	VAL	C
45	150	SER	C
46	151	SER	C
47	153	ARG	C
48	154	ASP	C
49	155	VAL	C
50	156	ASN	C
51	157	PRO	C
52	158	ALA	C
53	159	LEU	C
54	160	GLY	C
55	161	THR	C
56	162	ILE	C
57	163	GLY	C
58	164	ASP	C
59	165	LEU	C
60	166	ARG	C
61	167	GLU	C
62	168	VAL	C
63	169	ILE	C
64	170	ALA	C
65	171	ALA	C
66	172	LEU	C
67	173	HIS	C
68	174	GLU	C
69	175	ALA	C
70	176	GLY	C
71	177	ILE	C
72	178	SER	C
73	179	ALA	C
74	180	VAL	C
75	181	VAL	C
76	182	ASP	C
77	183	PHE	C
78	184	ILE	C
79	185	PHE	C
80	186	ASN	C
81	187	HIS	C
82	188	THR	C
83	189	SER	C
84	190	ASN	C
85	191	GLU	C
86	192	HIS	C
87	193	GLU	C
88	194	TRP	C
89	195	ALA	C

Continued on next page

Table S54 – *Continued from previous page*

Homology index	Residue Index	Residue abbreviation	Module
90	206	ASP	C
91	207	ASN	C
92	208	PHE	C
93	209	TYR	C
94	210	TYR	B
95	247	TRP	B
96	248	THR	B
97	249	THR	B
98	250	PHE	B
99	254	GLN	B
100	255	TRP	B
101	256	ASP	B
102	257	LEU	B
103	258	ASN	B
104	259	TYR	B
105	260	SER	B
106	261	ASN	B
107	262	PRO	B
108	263	TRP	B
109	264	VAL	B
110	265	PHE	B
111	266	ARG	B
112	267	ALA	B
113	268	MET	B
114	269	ALA	B
115	270	GLY	B
116	271	GLU	B
117	272	MET	B
118	273	LEU	B
119	274	PHE	B
120	275	LEU	B
121	276	ALA	B
122	277	ASN	B
123	278	LEU	C
124	279	GLY	C
125	280	VAL	C
126	281	ASP	C
127	282	ILE	C
128	283	LEU	B
129	284	ARG	D
130	285	MET	B
131	286	ASP	B
132	287	ALA	B
133	288	VAL	B
134	289	ALA	B
135	290	PHE	B

Continued on next page

Table S54 – *Continued from previous page*

Homology index	Residue Index	Residue abbreviation	Module
136	291	ILE	B
137	292	TRP	B
138	306	HIS	B
139	307	ALA	B
140	308	LEU	B
141	309	ILE	B
142	310	ARG	B
143	311	ALA	B
144	312	PHE	B
145	313	ASN	B
146	314	ALA	B
147	315	VAL	B
148	316	MET	B
149	317	ARG	B
150	323	VAL	B
151	324	PHE	B
152	325	PHE	B
153	326	LYS	C
154	327	SER	B
155	328	GLU	B
156	329	ALA	B
157	330	ILE	B
158	331	VAL	B
159	332	HIS	B
160	336	VAL	B
161	337	VAL	B
162	338	GLN	B
163	339	TYR	B
164	340	ILE	B
165	341	GLY	B
166	345	CYS	B
167	346	GLN	B
168	347	ILE	B
169	348	GLY	B
170	349	TYR	D
171	350	ASN	D
172	351	PRO	D
173	352	LEU	D
174	354	MET	D
175	355	ALA	D
176	356	LEU	D
177	357	LEU	D
178	358	TRP	D
179	359	ASN	D
180	360	THR	D
181	361	LEU	D

Continued on next page

Table S54 – *Continued from previous page*

Homology index	Residue Index	Residue abbreviation	Module
182	364	ARG	D
183	365	GLU	A
184	366	VAL	A
185	367	ASN	A
186	372	ALA	A
187	373	LEU	D
188	374	THR	D
189	375	TYR	D
190	376	ARG	D
191	377	HIS	B
192	378	ASN	B
193	381	GLU	B
194	382	HIS	B
195	383	THR	B
196	384	ALA	B
197	385	TRP	B
198	386	VAL	D
199	387	ASN	D
200	388	TYR	D
201	389	VAL	A
202	390	ARG	D
203	391	SER	D
204	392	HIS	D
205	393	ASP	D
206	394	ASP	D
207	448	SER	D
208	450	THR	D
209	451	ALA	D
210	452	ALA	D
211	453	ALA	D
212	464	ALA	A
213	467	ARG	A
214	468	ILE	A
215	469	LYS	A
216	470	LEU	A
217	471	LEU	A
218	472	TYR	A
219	473	SER	A
220	474	ILE	A
221	475	ALA	A
222	476	LEU	A
223	477	SER	A
224	478	THR	A
225	479	GLY	D
226	480	GLY	D
227	481	LEU	D

Continued on next page

Table S54 – *Continued from previous page*

Homology index	Residue Index	Residue abbreviation	Module
228	482	PRO	A
229	483	LEU	D
230	484	ILE	A
231	485	TYR	D
232	486	LEU	C
233	487	GLY	D
234	488	ASP	A
235	514	PRO	C
236	515	ARG	C
237	516	TYR	D
238	517	ASN	D
239	521	TYR	D
240	534	ILE	A
241	535	TYR	A
242	536	GLN	A
243	537	ASP	A
244	538	LEU	A
245	539	ARG	A
246	540	HIS	A
247	541	MET	A
248	542	ILE	A
249	543	ALA	A
250	544	VAL	A
251	545	ARG	A
252	546	GLN	A
253	547	SER	A
254	548	ASN	A
255	549	PRO	A
256	553	GLY	A
257	554	GLY	A
258	555	ARG	A
259	556	LEU	A
260	558	THR	A
261	559	PHE	A
262	560	ASN	A
263	561	THR	A
264	563	ASN	A
265	564	LYS	A
266	565	HIS	A
267	566	ILE	A
268	567	ILE	A
269	568	GLY	A
270	569	TYR	A
271	570	ILE	A
272	571	ARG	A
273	573	ASN	A

Continued on next page

Table S54 – *Continued from previous page*

**Homology index**	**Residue Index**	**Residue abbreviation**	**Module**
274	574	ALA	A
275	575	LEU	A
276	576	LEU	A
277	577	ALA	A
278	578	PHE	A
279	579	GLY	A
280	580	ASN	A
281	584	TYR	A
282	585	PRO	A
283	586	GLN	A
284	587	THR	A
285	588	VAL	A
286	589	THR	A
287	590	ALA	A
288	599	LYS	A
289	600	ALA	A
290	601	HIS	A
291	602	ASP	A
292	603	LEU	A
293	604	ILE	A
294	617	LEU	A
295	618	GLN	A
296	619	PRO	A
297	621	GLN	A
298	622	VAL	A
299	623	MET	A
300	624	TRP	A
301	625	LEU	A
302	626	GLU	A

Table S55: Residues membership for the *T. molitor* α-amylase (PDB code 1VIW, chain A)

**Homology index**	**Residue Index**	**Residue abbreviation**	**Module**
0	11	ASN	A
1	12	SER	A
2	13	ILE	B
3	14	VAL	B
4	15	HIS	B
5	16	LEU	C
6	17	PHE	C
7	18	GLU	C
8	19	TRP	C

Continued on next page

Table S55 – *Continued from previous page*

Homology index	Residue Index	Residue abbreviation	Module
9	20	LYS	C
10	21	TRP	C
11	22	ASN	C
12	23	ASP	C
13	24	ILE	C
14	25	ALA	C
15	26	ASP	C
16	27	GLU	C
17	28	CYS	C
18	29	GLU	C
19	30	ARG	C
20	32	LEU	C
21	33	GLN	C
22	34	PRO	C
23	35	GLN	C
24	36	GLY	C
25	37	PHE	C
26	38	GLY	C
27	39	GLY	C
28	40	VAL	C
29	41	GLN	C
30	42	ILE	C
31	43	SER	C
32	44	PRO	C
33	45	PRO	C
34	46	ASN	C
35	47	GLU	C
36	48	TYR	C
37	49	LEU	C
38	51	ALA	C
39	57	TRP	C
40	58	GLU	C
41	59	ARG	C
42	60	TYR	C
43	61	GLN	C
44	62	PRO	C
45	63	VAL	C
46	64	SER	C
47	65	TYR	C
48	66	ILE	C
49	67	ILE	C
50	68	ASN	C
51	69	THR	C
52	70	ARG	C
53	71	SER	C
54	72	GLY	C

Continued on next page

Table S55 – *Continued from previous page*

Homology index	Residue Index	Residue abbreviation	Module
55	73	ASN	C
56	74	GLU	C
57	75	SER	C
58	76	ALA	C
59	77	PHE	C
60	78	THR	C
61	79	ASP	C
62	80	MET	C
63	81	THR	C
64	82	ARG	C
65	83	ARG	C
66	84	CYS	C
67	85	ASN	C
68	86	ASP	C
69	87	ALA	C
70	88	GLY	C
71	89	VAL	C
72	90	ARG	C
73	91	ILE	C
74	92	TYR	C
75	93	VAL	C
76	94	ASP	C
77	95	ALA	C
78	96	VAL	C
79	97	ILE	C
80	98	ASN	C
81	99	HIS	C
82	100	MET	C
83	101	THR	C
84	102	GLY	C
85	103	MET	C
86	120	TYR	C
87	124	PRO	C
88	125	TYR	C
89	126	GLY	C
90	127	SER	C
91	128	GLY	C
92	129	ASP	C
93	130	PHE	C
94	131	HIS	B
95	148	CYS	B
96	149	GLU	B
97	150	LEU	B
98	151	VAL	B
99	153	LEU	B
100	154	ARG	B

Continued on next page

Table S55 – *Continued from previous page*

Homology index	Residue Index	Residue abbreviation	Module
101	155	ASP	B
102	156	LEU	B
103	157	ASN	B
104	158	ALA	B
105	159	GLY	B
106	160	SER	B
107	161	ASP	B
108	162	TYR	B
109	163	VAL	B
110	164	ARG	B
111	165	GLY	B
112	166	VAL	B
113	167	LEU	B
114	168	ILE	B
115	169	ASP	B
116	170	TYR	B
117	171	MET	B
118	172	ASN	B
119	173	HIS	B
120	174	MET	B
121	175	ILE	B
122	176	ASP	B
123	177	LEU	C
124	178	GLY	C
125	179	VAL	C
126	180	ALA	C
127	181	GLY	C
128	182	PHE	B
129	183	ARG	D
130	184	VAL	B
131	185	ASP	B
132	186	ALA	B
133	187	ALA	B
134	188	LYS	B
135	189	HIS	B
136	190	MET	B
137	191	SER	B
138	192	PRO	B
139	193	GLY	B
140	194	ASP	B
141	195	LEU	B
142	196	SER	B
143	197	VAL	B
144	198	ILE	B
145	199	PHE	B
146	200	ASP	B

Continued on next page

Table S55 – *Continued from previous page*

Homology index	Residue Index	Residue abbreviation	Module
147	201	GLY	B
148	202	LEU	B
149	203	LYS	B
150	217	PRO	B
151	218	PHE	B
152	219	ILE	B
153	220	TYR	C
154	221	GLN	B
155	222	GLU	B
156	223	VAL	B
157	224	ILE	B
158	225	ASP	B
159	226	LEU	B
160	233	LYS	B
161	234	ASN	B
162	235	GLU	B
163	236	TYR	B
164	237	THR	B
165	238	GLY	B
166	239	PHE	B
167	240	GLY	B
168	241	CYS	B
169	242	VAL	B
170	243	LEU	D
171	244	GLU	D
172	245	PHE	D
173	246	GLN	D
174	248	GLY	D
175	249	VAL	D
176	250	SER	D
177	251	LEU	D
178	252	GLY	D
179	253	ASN	D
180	254	ALA	D
181	255	PHE	D
182	259	ASN	D
183	260	GLN	A
184	261	LEU	A
185	262	LYS	A
186	267	TRP	A
187	268	GLY	D
188	269	PRO	D
189	270	GLU	D
190	272	GLY	D
191	273	LEU	B
192	274	LEU	B

Continued on next page

Table S55 – *Continued from previous page*

Homology index	Residue Index	Residue abbreviation	Module
193	275	GLU	B
194	276	GLY	B
195	277	LEU	B
196	278	ASP	B
197	279	ALA	B
198	280	VAL	D
199	281	VAL	D
200	282	PHE	D
201	283	VAL	A
202	284	ASP	D
203	285	ASN	D
204	286	HIS	D
205	287	ASP	D
206	288	ASN	D
207	289	GLN	D
208	298	THR	D
209	299	TYR	D
210	300	LYS	D
211	301	ASN	D
212	303	LYS	A
213	304	PRO	A
214	305	TYR	A
215	306	LYS	A
216	307	MET	A
217	308	ALA	A
218	309	ILE	A
219	310	ALA	A
220	311	PHE	A
221	312	MET	A
222	313	LEU	A
223	314	ALA	A
224	315	HIS	A
225	317	TYR	D
226	318	GLY	D
227	319	THR	D
228	320	THR	A
229	321	ARG	D
230	322	ILE	A
231	323	MET	D
232	324	SER	C
233	325	SER	D
234	326	PHE	A
235	327	ASP	C
236	357	GLY	C
237	358	TYR	D
238	359	VAL	D

Continued on next page

Table S55 – *Continued from previous page*

Homology index	Residue Index	Residue abbreviation	Module
239	360	CYS	D
240	363	ARG	A
241	364	TRP	A
242	365	ARG	A
243	366	GLN	A
244	367	VAL	A
245	368	TYR	A
246	369	GLY	A
247	370	MET	A
248	371	VAL	A
249	372	GLY	A
250	373	PHE	A
251	374	ARG	A
252	375	ASN	A
253	376	ALA	A
254	377	VAL	A
255	378	GLU	A
256	379	GLY	A
257	380	THR	A
258	381	GLN	A
259	382	VAL	A
260	384	ASN	A
261	385	TRP	A
262	386	TRP	A
263	387	SER	A
264	389	ASP	A
265	390	ASP	A
266	391	ASN	A
267	392	GLN	A
268	393	ILE	A
269	394	ALA	A
270	395	PHE	A
271	396	SER	A
272	397	ARG	A
273	398	GLY	A
274	399	SER	A
275	401	GLY	A
276	402	PHE	A
277	403	VAL	A
278	404	ALA	A
279	405	PHE	A
280	406	THR	A
281	408	GLY	A
282	409	GLY	A
283	410	ASP	A
284	411	LEU	A

Continued on next page

Table S55 – *Continued from previous page*

Homology index	Residue Index	Residue abbreviation	Module
285	412	ASN	A
286	413	GLN	A
287	414	ASN	A
288	420	PRO	A
289	421	ALA	A
290	424	TYR	A
291	425	CYS	A
292	426	ASP	A
293	427	VAL	A
294	454	LEU	A
295	455	GLY	A
296	456	SER	A
297	461	GLY	A
298	462	VAL	A
299	463	LEU	A
300	464	ALA	A
301	465	ILE	A
302	466	HIS	A

Table S56: Residues membership for the *B. cereus* α-amylase (PDB code 1UOK, chain A)

Homology index	Residue Index	Residue abbreviation	Module
0	9	SER	A
1	10	VAL	A
2	11	VAL	B
3	12	TYR	B
4	13	GLN	B
5	14	ILE	C
6	15	TYR	C
7	16	PRO	C
8	28	GLY	C
9	29	ASP	C
10	30	LEU	C
11	31	ARG	C
12	32	GLY	C
13	33	ILE	C
14	34	ILE	C
15	35	SER	C
16	36	LYS	C
17	37	LEU	C
18	38	ASP	C
19	39	TYR	C

Continued on next page

Table S56 – *Continued from previous page*

Homology index	Residue Index	Residue abbreviation	Module
20	40	LEU	C
21	41	LYS	C
22	42	GLU	C
23	43	LEU	C
24	44	GLY	C
25	45	ILE	C
26	46	ASP	C
27	47	VAL	C
28	48	ILE	C
29	49	TRP	C
30	50	LEU	C
31	51	SER	C
32	52	PRO	C
33	53	VAL	C
34	54	TYR	C
35	55	GLU	C
36	56	SER	C
37	57	PRO	C
38	58	ASN	C
39	60	ASP	C
40	61	ASN	C
41	62	GLY	C
42	63	TYR	C
43	64	ASP	C
44	65	ILE	C
45	66	SER	C
46	67	ASP	C
47	69	CYS	C
48	70	LYS	C
49	71	ILE	C
50	72	MET	C
51	73	ASN	C
52	74	GLU	C
53	75	PHE	C
54	76	GLY	C
55	77	THR	C
56	78	MET	C
57	79	GLU	C
58	80	ASP	C
59	81	TRP	C
60	82	ASP	C
61	83	GLU	C
62	84	LEU	C
63	85	LEU	C
64	86	HIS	C
65	87	GLU	C

Continued on next page

Table S56 – *Continued from previous page*

Homology index	Residue Index	Residue abbreviation	Module
66	88	MET	C
67	89	HIS	C
68	90	GLU	C
69	91	ARG	C
70	92	ASN	C
71	93	MET	C
72	94	LYS	C
73	95	LEU	C
74	96	MET	C
75	97	MET	C
76	98	ASP	C
77	99	LEU	C
78	100	VAL	C
79	101	VAL	C
80	102	ASN	C
81	103	HIS	C
82	104	THR	C
83	105	SER	C
84	106	ASP	C
85	107	GLU	C
86	108	HIS	C
87	109	ASN	C
88	110	TRP	C
89	111	PHE	C
90	123	ARG	C
91	124	ASP	C
92	125	TYR	C
93	126	TYR	C
94	127	ILE	B
95	160	LEU	B
96	161	HIS	B
97	162	LEU	B
98	163	PHE	B
99	167	GLN	B
100	168	PRO	B
101	169	ASP	B
102	170	LEU	B
103	171	ASN	B
104	172	TRP	B
105	173	ASP	B
106	174	ASN	B
107	175	GLU	B
108	176	LYS	B
109	177	VAL	B
110	178	ARG	B
111	179	GLN	B

Continued on next page

Table S56 – *Continued from previous page*

Homology index	Residue Index	Residue abbreviation	Module
112	180	ASP	B
113	181	VAL	B
114	182	TYR	B
115	183	GLU	B
116	184	MET	B
117	185	MET	B
118	186	LYS	B
119	187	PHE	B
120	188	TRP	B
121	189	LEU	B
122	190	GLU	B
123	191	LYS	C
124	192	GLY	C
125	193	ILE	C
126	194	ASP	C
127	195	GLY	C
128	196	PHE	B
129	197	ARG	D
130	198	MET	B
131	199	ASP	B
132	200	VAL	B
133	201	ILE	B
134	202	ASN	B
135	203	PHE	B
136	204	ILE	B
137	205	SER	B
138	234	HIS	B
139	235	LYS	B
140	236	TYR	B
141	237	LEU	B
142	238	HIS	B
143	239	GLU	B
144	240	MET	B
145	241	ASN	B
146	242	GLU	B
147	243	GLU	B
148	244	VAL	B
149	245	LEU	B
150	250	ILE	B
151	251	MET	B
152	252	THR	B
153	253	VAL	C
154	254	GLY	B
155	255	GLU	B
156	256	MET	B
157	257	PRO	B

Continued on next page

Table S56 – *Continued from previous page*

Homology index	Residue Index	Residue abbreviation	Module
158	258	GLY	B
159	259	VAL	B
160	264	ALA	B
161	265	LYS	B
162	266	LEU	B
163	267	TYR	B
164	268	THR	B
165	269	GLY	B
166	275	LEU	B
167	276	GLN	B
168	277	MET	B
169	278	VAL	B
170	279	PHE	D
171	280	GLN	D
172	281	PHE	D
173	282	GLU	D
174	284	MET	D
175	285	ASP	D
176	286	LEU	D
177	287	ASP	D
178	288	SER	D
179	289	GLY	D
180	290	GLU	D
181	291	GLY	D
182	300	SER	D
183	301	LEU	A
184	302	LEU	A
185	303	THR	A
186	308	LEU	A
187	309	THR	D
188	310	LYS	D
189	311	TRP	D
190	312	GLN	D
191	313	LYS	B
192	314	ALA	B
193	317	HIS	B
194	318	THR	B
195	319	GLY	B
196	320	TRP	B
197	321	ASN	B
198	322	SER	D
199	323	LEU	D
200	324	TYR	D
201	325	TRP	A
202	326	ASN	D
203	327	ASN	D

Continued on next page

Table S56 – *Continued from previous page*

Homology index	Residue Index	Residue abbreviation	Module
204	328	HIS	D
205	329	ASP	D
206	330	GLN	D
207	331	PRO	D
208	332	ARG	D
209	333	VAL	D
210	334	VAL	D
211	335	SER	D
212	344	ARG	A
213	347	SER	A
214	348	ALA	A
215	349	LYS	A
216	350	MET	A
217	351	LEU	A
218	352	ALA	A
219	353	THR	A
220	354	VAL	A
221	355	LEU	A
222	356	HIS	A
223	357	MET	A
224	358	MET	A
225	359	LYS	D
226	360	GLY	D
227	361	THR	D
228	362	PRO	A
229	363	TYR	D
230	364	ILE	A
231	365	TYR	D
232	366	GLN	C
233	367	GLY	D
234	368	GLU	A
235	420	THR	C
236	421	PRO	C
237	422	MET	D
238	423	GLN	D
239	424	TRP	D
240	460	ILE	A
241	461	PHE	A
242	462	TYR	A
243	463	TYR	A
244	464	TYR	A
245	465	LYS	A
246	466	LYS	A
247	467	LEU	A
248	468	ILE	A
249	469	GLU	A

Continued on next page

Table S56 – *Continued from previous page*

Homology index	Residue Index	Residue abbreviation	Module
250	470	LEU	A
251	471	ARG	A
252	472	LYS	A
253	473	ASN	A
254	474	ASN	A
255	475	GLU	A
256	479	TYR	A
257	480	GLY	A
258	481	SER	A
259	482	TYR	A
260	484	LEU	A
261	485	ILE	A
262	486	LEU	A
263	487	GLU	A
264	489	ASN	A
265	490	PRO	A
266	491	SER	A
267	492	ILE	A
268	493	PHE	A
269	494	ALA	A
270	495	TYR	A
271	496	VAL	A
272	497	ARG	A
273	502	GLU	A
274	503	LYS	A
275	504	LEU	A
276	505	LEU	A
277	506	VAL	A
278	507	ILE	A
279	508	ALA	A
280	509	ASN	A
281	513	GLU	A
282	514	GLU	A
283	515	CYS	A
284	516	ILE	A
285	517	PHE	A
286	518	GLU	A
287	519	LEU	A
288	527	GLU	A
289	528	VAL	A
290	530	LEU	A
291	531	LEU	A
292	532	ILE	A
293	533	HIS	A
294	547	LEU	A
295	548	ARG	A

Continued on next page

Table S56 – *Continued from previous page*

Homology index	Residue Index	Residue abbreviation	Module
296	549	PRO	A
297	551	GLU	A
298	552	ALA	A
299	553	MET	A
300	554	VAL	A
301	555	PHE	A
302	556	LYS	A

Table S57: Residues membership for the *H. sapiens* α-amylase (PDB code 1SMD, chain A)

Homology index	Residue Index	Residue abbreviation	Module
0	11	THR	A
1	12	SER	A
2	13	ILE	B
3	14	VAL	B
4	15	HIS	B
5	16	LEU	C
6	17	PHE	C
7	18	GLU	C
8	19	TRP	C
9	20	ARG	C
10	21	TRP	C
11	22	VAL	C
12	23	ASP	C
13	24	ILE	C
14	25	ALA	C
15	26	LEU	C
16	27	GLU	C
17	28	CYS	C
18	29	GLU	C
19	30	ARG	C
20	32	LEU	C
21	33	ALA	C
22	34	PRO	C
23	35	LYS	C
24	36	GLY	C
25	37	PHE	C
26	38	GLY	C
27	39	GLY	C
28	40	VAL	C
29	41	GLN	C
30	42	VAL	C

Continued on next page

Table S57 – *Continued from previous page*

Homology index	Residue Index	Residue abbreviation	Module
31	43	SER	C
32	44	PRO	C
33	45	PRO	C
34	46	ASN	C
35	47	GLU	C
36	48	ASN	C
37	49	VAL	C
38	51	ILE	C
39	59	TRP	C
40	60	GLU	C
41	61	ARG	C
42	62	TYR	C
43	63	GLN	C
44	64	PRO	C
45	65	VAL	C
46	66	SER	C
47	67	TYR	C
48	68	LYS	C
49	69	LEU	C
50	70	CYS	C
51	71	THR	C
52	72	ARG	C
53	73	SER	C
54	74	GLY	C
55	75	ASN	C
56	76	GLU	C
57	77	ASP	C
58	78	GLU	C
59	79	PHE	C
60	80	ARG	C
61	81	ASN	C
62	82	MET	C
63	83	VAL	C
64	84	THR	C
65	85	ARG	C
66	86	CYS	C
67	87	ASN	C
68	88	ASN	C
69	89	VAL	C
70	90	GLY	C
71	91	VAL	C
72	92	ARG	C
73	93	ILE	C
74	94	TYR	C
75	95	VAL	C
76	96	ASP	C

Continued on next page

Table S57 – *Continued from previous page*

Homology index	Residue Index	Residue abbreviation	Module
77	97	ALA	C
78	98	VAL	C
79	99	ILE	C
80	100	ASN	C
81	101	HIS	C
82	102	MET	C
83	103	CYS	C
84	104	GLY	C
85	105	ASN	C
86	126	PHE	C
87	130	PRO	C
88	131	TYR	C
89	132	SER	C
90	133	GLY	C
91	134	TRP	C
92	135	ASP	C
93	136	PHE	C
94	137	ASN	B
95	160	CYS	B
96	161	ARG	B
97	162	LEU	B
98	163	SER	B
99	165	LEU	B
100	166	LEU	B
101	167	ASP	B
102	168	LEU	B
103	169	ALA	B
104	170	LEU	B
105	171	GLY	B
106	172	LYS	B
107	173	ASP	B
108	174	TYR	B
109	175	VAL	B
110	176	ARG	B
111	177	SER	B
112	178	LYS	B
113	179	ILE	B
114	180	ALA	B
115	181	GLU	B
116	182	TYR	B
117	183	MET	B
118	184	ASN	B
119	185	HIS	B
120	186	LEU	B
121	187	ILE	B
122	188	ASP	B

Continued on next page

Table S57 – *Continued from previous page*

Homology index	Residue Index	Residue abbreviation	Module
123	189	ILE	C
124	190	GLY	C
125	191	VAL	C
126	192	ALA	C
127	193	GLY	C
128	194	PHE	B
129	195	ARG	D
130	196	ILE	B
131	197	ASP	B
132	198	ALA	B
133	199	SER	B
134	200	LYS	B
135	201	HIS	B
136	202	MET	B
137	203	TRP	B
138	204	PRO	B
139	205	GLY	B
140	206	ASP	B
141	207	ILE	B
142	208	LYS	B
143	209	ALA	B
144	210	ILE	B
145	211	LEU	B
146	212	ASP	B
147	213	LYS	B
148	214	LEU	B
149	215	HIS	B
150	228	PRO	B
151	229	PHE	B
152	230	ILE	B
153	231	TYR	C
154	232	GLN	B
155	233	GLU	B
156	234	VAL	B
157	235	ILE	B
158	236	ASP	B
159	237	LEU	B
160	244	SER	B
161	245	SER	B
162	246	ASP	B
163	247	TYR	B
164	248	PHE	B
165	249	GLY	B
166	250	ASN	B
167	251	GLY	B
168	252	ARG	B

Continued on next page

Table S57 – *Continued from previous page*

Homology index	Residue Index	Residue abbreviation	Module
169	253	VAL	B
170	254	THR	D
171	255	GLU	D
172	256	PHE	D
173	257	LYS	D
174	259	GLY	D
175	260	ALA	D
176	261	LYS	D
177	262	LEU	D
178	263	GLY	D
179	264	THR	D
180	265	VAL	D
181	266	ILE	D
182	272	GLU	D
183	273	LYS	A
184	274	MET	A
185	275	SER	A
186	280	TRP	A
187	281	GLY	D
188	282	GLU	D
189	283	GLY	D
190	285	GLY	D
191	286	PHE	B
192	287	MET	B
193	288	PRO	B
194	289	SER	B
195	290	ASP	B
196	291	ARG	B
197	292	ALA	B
198	293	LEU	D
199	294	VAL	D
200	295	PHE	D
201	296	VAL	A
202	297	ASP	D
203	298	ASN	D
204	299	HIS	D
205	300	ASP	D
206	301	ASN	D
207	302	GLN	D
208	314	THR	D
209	315	PHE	D
210	316	TRP	D
211	317	ASP	D
212	319	ARG	A
213	320	LEU	A
214	321	TYR	A

Continued on next page

Table S57 – *Continued from previous page*

**Homology index**	**Residue Index**	**Residue abbreviation**	**Module**
215	322	LYS	A
216	323	MET	A
217	324	ALA	A
218	325	VAL	A
219	326	GLY	A
220	327	PHE	A
221	328	MET	A
222	329	LEU	A
223	330	ALA	A
224	331	HIS	A
225	333	TYR	D
226	334	GLY	D
227	335	PHE	D
228	336	THR	A
229	337	ARG	D
230	338	VAL	A
231	339	MET	D
232	340	SER	C
233	341	SER	D
234	342	TYR	A
235	343	ARG	C
236	381	ASP	C
237	382	TRP	D
238	383	VAL	D
239	384	CYS	D
240	387	ARG	A
241	388	TRP	A
242	389	ARG	A
243	390	GLN	A
244	391	ILE	A
245	392	ARG	A
246	393	ASN	A
247	394	MET	A
248	395	VAL	A
249	396	ASN	A
250	397	PHE	A
251	398	ARG	A
252	399	ASN	A
253	400	VAL	A
254	401	VAL	A
255	402	ASP	A
256	403	GLY	A
257	404	GLN	A
258	405	PRO	A
259	406	PHE	A
260	408	ASN	A

Continued on next page

Table S57 – *Continued from previous page*

Homology index	Residue Index	Residue abbreviation	Module
261	409	TRP	A
262	410	TYR	A
263	411	ASP	A
264	413	GLY	A
265	414	SER	A
266	415	ASN	A
267	416	GLN	A
268	417	VAL	A
269	418	ALA	A
270	419	PHE	A
271	420	GLY	A
272	421	ARG	A
273	422	GLY	A
274	423	ASN	A
275	425	GLY	A
276	426	PHE	A
277	427	ILE	A
278	428	VAL	A
279	429	PHE	A
280	430	ASN	A
281	433	ASP	A
282	434	TRP	A
283	435	THR	A
284	436	PHE	A
285	437	SER	A
286	438	LEU	A
287	439	THR	A
288	445	PRO	A
289	446	ALA	A
290	449	TYR	A
291	450	CYS	A
292	451	ASP	A
293	452	VAL	A
294	479	ILE	A
295	480	SER	A
296	481	ASN	A
297	486	PRO	A
298	487	PHE	A
299	488	ILE	A
300	489	ALA	A
301	490	ILE	A
302	491	HIS	A

Table S58: Residues membership for the *B. circulans* α-amylase (PDB code 1OT1, chain A)

Homology index	Residue Index	Residue abbreviation	Module
0	15	ASP	A
1	16	VAL	A
2	17	ILE	B
3	18	TYR	B
4	19	GLN	B
5	20	ILE	C
6	21	PHE	C
7	22	THR	C
8	52	GLY	C
9	53	ASP	C
10	54	TRP	C
11	55	GLN	C
12	56	GLY	C
13	57	ILE	C
14	58	ILE	C
15	59	ASN	C
16	60	LYS	C
17	61	ILE	C
18	62	ASN	C
19	63	ASP	C
20	66	LEU	C
21	67	THR	C
22	68	GLY	C
23	69	MET	C
24	70	GLY	C
25	71	VAL	C
26	72	THR	C
27	73	ALA	C
28	74	ILE	C
29	75	TRP	C
30	76	ILE	C
31	77	SER	C
32	78	GLN	C
33	79	PRO	C
34	80	VAL	C
35	81	GLU	C
36	82	ASN	C
37	83	ILE	C
38	95	THR	C
39	97	TYR	C
40	98	HIS	C
41	99	GLY	C
42	100	TYR	C
43	101	TRP	C

Continued on next page

Table S58 – *Continued from previous page*

Homology index	Residue Index	Residue abbreviation	Module
44	102	ALA	C
45	103	ARG	C
46	104	ASP	C
47	106	LYS	C
48	107	LYS	C
49	108	THR	C
50	109	ASN	C
51	110	PRO	C
52	111	ALA	C
53	112	TYR	C
54	113	GLY	C
55	114	THR	C
56	115	ILE	C
57	116	ALA	C
58	117	ASP	C
59	118	PHE	C
60	119	GLN	C
61	120	ASN	C
62	121	LEU	C
63	122	ILE	C
64	123	ALA	C
65	124	ALA	C
66	125	ALA	C
67	126	HIS	C
68	127	ALA	C
69	128	LYS	C
70	129	ASN	C
71	130	ILE	C
72	131	LYS	C
73	132	VAL	C
74	133	ILE	C
75	134	ILE	C
76	135	ALA	C
77	136	PHE	C
78	137	ALA	C
79	138	PRO	C
80	139	ASN	C
81	140	HIS	C
82	141	THR	C
83	142	SER	C
84	143	PRO	C
85	144	ALA	C
86	156	ARG	C
87	163	LEU	C
88	164	LEU	C
89	165	GLY	C

Continued on next page

Table S58 – *Continued from previous page*

Homology index	Residue Index	Residue abbreviation	Module
90	172	GLN	C
91	173	ASN	C
92	174	LEU	C
93	175	PHE	C
94	176	HIS	B
95	192	LYS	B
96	193	ASN	B
97	194	LEU	B
98	195	TYR	B
99	197	LEU	B
100	198	ALA	B
101	199	ASP	B
102	200	LEU	B
103	201	ASN	B
104	202	HIS	B
105	203	ASN	B
106	204	ASN	B
107	205	SER	B
108	206	THR	B
109	207	VAL	B
110	208	ASP	B
111	209	VAL	B
112	210	TYR	B
113	211	LEU	B
114	212	LYS	B
115	213	ASP	B
116	214	ALA	B
117	215	ILE	B
118	216	LYS	B
119	217	MET	B
120	218	TRP	B
121	219	LEU	B
122	220	ASP	B
123	221	LEU	C
124	222	GLY	C
125	223	ILE	C
126	224	ASP	C
127	225	GLY	C
128	226	ILE	B
129	227	ARG	D
130	228	MET	B
131	229	ASP	B
132	230	ALA	B
133	231	VAL	B
134	232	LYS	B
135	233	HIS	B

Continued on next page

Table S58 – *Continued from previous page*

Homology index	Residue Index	Residue abbreviation	Module
136	234	MET	B
137	235	PRO	B
138	236	PHE	B
139	237	GLY	B
140	238	TRP	B
141	239	GLN	B
142	240	LYS	B
143	241	SER	B
144	242	PHE	B
145	243	MET	B
146	244	ALA	B
147	245	ALA	B
148	246	VAL	B
149	247	ASN	B
150	252	VAL	B
151	253	PHE	B
152	254	THR	B
153	255	PHE	C
154	256	GLY	B
155	257	GLU	B
156	258	TRP	B
157	259	PHE	B
158	260	LEU	B
159	261	GLY	B
160	269	ASN	B
161	270	HIS	B
162	271	LYS	B
163	272	PHE	B
164	274	ASN	B
165	275	GLU	B
166	277	GLY	B
167	278	MET	B
168	279	SER	B
169	280	LEU	B
170	281	LEU	D
171	282	ASP	D
172	283	PHE	D
173	284	ARG	D
174	286	ALA	D
175	287	GLN	D
176	288	LYS	D
177	289	VAL	D
178	290	ARG	D
179	291	GLN	D
180	292	VAL	D
181	293	PHE	D

Continued on next page

Table S58 – *Continued from previous page*

Homology index	Residue Index	Residue abbreviation	Module
182	298	ASP	D
183	299	ASN	A
184	300	MET	A
185	301	TYR	A
186	306	MET	A
187	307	LEU	D
188	308	GLU	D
189	309	GLY	D
190	310	SER	D
191	311	ALA	B
192	312	ALA	B
193	316	GLN	B
194	317	VAL	B
195	318	ASP	B
196	319	ASP	B
197	320	GLN	B
198	321	VAL	D
199	322	THR	D
200	323	PHE	D
201	324	ILE	A
202	325	ASP	D
203	326	ASN	D
204	327	HIS	D
205	328	ASP	D
206	329	MET	D
207	330	GLU	D
208	331	ARG	D
209	332	PHE	D
210	333	HIS	D
211	334	ALA	D
212	339	ARG	A
213	341	LYS	A
214	342	LEU	A
215	343	GLU	A
216	344	GLN	A
217	345	ALA	A
218	346	LEU	A
219	347	ALA	A
220	348	PHE	A
221	349	THR	A
222	350	LEU	A
223	351	THR	A
224	352	SER	A
225	353	ARG	D
226	354	GLY	D
227	355	VAL	D

Continued on next page

Table S58 – *Continued from previous page*

Homology index	Residue Index	Residue abbreviation	Module
228	356	PRO	A
229	357	ALA	D
230	358	ILE	A
231	359	TYR	D
232	360	TYR	C
233	361	GLY	D
234	362	THR	A
235	376	ALA	C
236	377	ARG	C
237	378	ILE	D
238	379	PRO	D
239	381	PHE	D
240	387	ALA	A
241	388	TYR	A
242	389	GLN	A
243	390	VAL	A
244	391	ILE	A
245	392	GLN	A
246	393	LYS	A
247	394	LEU	A
248	395	ALA	A
249	396	PRO	A
250	397	LEU	A
251	398	ARG	A
252	399	LYS	A
253	400	SER	A
254	401	ASN	A
255	402	PRO	A
256	406	TYR	A
257	407	GLY	A
258	408	SER	A
259	409	THR	A
260	411	GLU	A
261	412	ARG	A
262	413	TRP	A
263	414	ILE	A
264	415	ASN	A
265	416	ASN	A
266	417	ASP	A
267	418	VAL	A
268	419	LEU	A
269	420	ILE	A
270	421	TYR	A
271	422	GLU	A
272	423	ARG	A
273	428	ASN	A

Continued on next page

Table S58 – *Continued from previous page*

Homology index	Residue Index	Residue abbreviation	Module
274	429	VAL	A
275	430	ALA	A
276	431	VAL	A
277	432	VAL	A
278	433	ALA	A
279	434	VAL	A
280	435	ASN	A
281	439	ASN	A
282	440	ALA	A
283	441	PRO	A
284	442	ALA	A
285	443	SER	A
286	444	ILE	A
287	445	SER	A
288	454	GLY	A
289	455	SER	A
290	457	ASN	A
291	458	ASP	A
292	459	VAL	A
293	460	LEU	A
294	483	ALA	A
295	484	ALA	A
296	485	GLY	A
297	486	GLY	A
298	487	THR	A
299	488	ALA	A
300	489	VAL	A
301	490	TRP	A
302	491	GLN	A

Table S59: Residues membership for the *T. vulgaris R47* α-amylase (PDB code 1WZL, chain A)

Homology index	Residue Index	Residue abbreviation	Module
0	131	ALA	A
1	132	VAL	A
2	133	ILE	B
3	134	TYR	B
4	135	GLN	B
5	136	ILE	C
6	137	PHE	C
7	138	PRO	C
8	170	GLY	C

Continued on next page

Table S59 – *Continued from previous page*

Homology index	Residue Index	Residue abbreviation	Module
9	171	ASP	C
10	172	LEU	C
11	173	LYS	C
12	174	GLY	C
13	175	VAL	C
14	176	ILE	C
15	177	ASP	C
16	178	ARG	C
17	179	LEU	C
18	180	PRO	C
19	181	TYR	C
20	182	LEU	C
21	183	GLU	C
22	184	GLU	C
23	185	LEU	C
24	186	GLY	C
25	187	VAL	C
26	188	THR	C
27	189	ALA	C
28	190	LEU	C
29	191	TYR	C
30	192	PHE	C
31	193	THR	C
32	194	PRO	C
33	195	ILE	C
34	196	PHE	C
35	197	ALA	C
36	198	SER	C
37	199	PRO	C
38	200	SER	C
39	201	HIS	C
40	202	HIS	C
41	203	LYS	C
42	204	TYR	C
43	205	ASP	C
44	206	THR	C
45	207	ALA	C
46	208	ASP	C
47	210	LEU	C
48	211	ALA	C
49	212	ILE	C
50	213	ASP	C
51	214	PRO	C
52	215	GLN	C
53	216	PHE	C
54	217	GLY	C

Continued on next page

Table S59 – *Continued from previous page*

Homology index	Residue Index	Residue abbreviation	Module
55	218	ASP	C
56	219	LEU	C
57	220	PRO	C
58	221	THR	C
59	222	PHE	C
60	223	ARG	C
61	224	ARG	C
62	225	LEU	C
63	226	VAL	C
64	227	ASP	C
65	228	GLU	C
66	229	ALA	C
67	230	HIS	C
68	231	ARG	C
69	232	ARG	C
70	233	GLY	C
71	234	ILE	C
72	235	LYS	C
73	236	ILE	C
74	237	ILE	C
75	238	LEU	C
76	239	ASP	C
77	240	ALA	C
78	241	VAL	C
79	242	PHE	C
80	243	ASN	C
81	244	HIS	C
82	245	ALA	C
83	246	GLY	C
84	247	ASP	C
85	248	GLN	C
86	249	PHE	C
87	250	PHE	C
88	251	ALA	C
89	252	PHE	C
90	265	LYS	C
91	266	ASP	C
92	267	TRP	C
93	268	PHE	C
94	269	PHE	B
95	284	GLU	B
96	285	THR	B
97	286	PHE	B
98	287	ALA	B
99	293	MET	B
100	294	PRO	B

Continued on next page

Table S59 – *Continued from previous page*

Homology index	Residue Index	Residue abbreviation	Module
101	295	LYS	B
102	296	LEU	B
103	297	ARG	B
104	298	THR	B
105	299	GLU	B
106	300	ASN	B
107	301	PRO	B
108	302	GLU	B
109	303	VAL	B
110	304	LYS	B
111	305	GLU	B
112	306	TYR	B
113	307	LEU	B
114	308	PHE	B
115	309	ASP	B
116	310	VAL	B
117	311	ALA	B
118	312	ARG	B
119	313	PHE	B
120	314	TRP	B
121	315	MET	B
122	316	GLU	B
123	317	GLN	C
124	318	GLY	C
125	319	ILE	C
126	320	ASP	C
127	321	GLY	C
128	322	TRP	B
129	323	ARG	D
130	324	LEU	B
131	325	ASP	B
132	326	VAL	B
133	327	ALA	B
134	328	ASN	B
135	329	GLU	B
136	330	VAL	B
137	331	ASP	B
138	332	HIS	B
139	333	ALA	B
140	334	PHE	B
141	335	TRP	B
142	336	ARG	B
143	337	GLU	B
144	338	PHE	B
145	339	ARG	B
146	340	ARG	B

Continued on next page

Table S59 – *Continued from previous page*

**Homology index**	**Residue Index**	**Residue abbreviation**	**Module**
147	341	LEU	B
148	342	VAL	B
149	343	LYS	B
150	349	ALA	B
151	350	LEU	B
152	351	ILE	B
153	352	VAL	C
154	353	GLY	B
155	354	GLU	B
156	355	ILE	B
157	356	TRP	B
158	357	HIS	B
159	358	ASP	B
160	359	ALA	B
161	360	SER	B
162	361	GLY	B
163	362	TRP	B
164	363	LEU	B
165	364	MET	B
166	368	PHE	B
167	369	ASP	B
168	370	SER	B
169	371	VAL	B
170	372	MET	D
171	373	ASN	D
172	374	TYR	D
173	375	LEU	D
174	377	ARG	D
175	378	GLU	D
176	379	SER	D
177	380	VAL	D
178	381	ILE	D
179	382	ARG	D
180	383	PHE	D
181	384	PHE	D
182	389	ILE	D
183	390	HIS	A
184	391	ALA	A
185	392	GLU	A
186	397	GLU	A
187	398	LEU	D
188	399	THR	D
189	400	ARG	D
190	401	ALA	D
191	402	ARG	B
192	403	MET	B

Continued on next page

Table S59 – *Continued from previous page*

Homology index	Residue Index	Residue abbreviation	Module
193	409	ALA	B
194	410	ALA	B
195	411	GLN	B
196	412	GLY	B
197	413	LEU	B
198	414	TRP	D
199	415	ASN	D
200	416	LEU	D
201	417	LEU	A
202	418	ASP	D
203	419	SER	D
204	420	HIS	D
205	421	ASP	D
206	422	THR	D
207	423	GLU	D
208	424	ARG	D
209	425	PHE	D
210	426	LEU	D
211	427	THR	D
212	433	GLU	A
213	435	LYS	A
214	436	PHE	A
215	437	ARG	A
216	438	LEU	A
217	439	ALA	A
218	440	VAL	A
219	441	LEU	A
220	442	PHE	A
221	443	GLN	A
222	444	MET	A
223	445	THR	A
224	446	TYR	A
225	447	LEU	D
226	448	GLY	D
227	449	THR	D
228	450	PRO	A
229	451	LEU	D
230	452	ILE	A
231	453	TYR	D
232	454	TYR	C
233	455	GLY	D
234	456	ASP	A
235	470	ARG	C
236	471	PRO	C
237	472	MET	D
238	473	ILE	D

Continued on next page

Table S59 – *Continued from previous page*

Homology index	Residue Index	Residue abbreviation	Module
239	474	TRP	D
240	483	LEU	A
241	484	PHE	A
242	485	GLU	A
243	486	PHE	A
244	487	TYR	A
245	488	LYS	A
246	489	GLU	A
247	490	LEU	A
248	491	ILE	A
249	492	ARG	A
250	493	LEU	A
251	494	ARG	A
252	495	HIS	A
253	496	ARG	A
254	497	LEU	A
255	498	ALA	A
256	502	ARG	A
257	503	GLY	A
258	504	ASN	A
259	505	VAL	A
260	507	SER	A
261	508	TRP	A
262	509	HIS	A
263	510	ALA	A
264	513	GLN	A
265	514	ALA	A
266	515	ASN	A
267	516	LEU	A
268	517	TYR	A
269	518	ALA	A
270	519	PHE	A
271	520	VAL	A
272	521	ARG	A
273	526	GLN	A
274	527	HIS	A
275	528	VAL	A
276	529	GLY	A
277	530	VAL	A
278	531	VAL	A
279	532	LEU	A
280	533	ASN	A
281	536	GLY	A
282	537	GLU	A
283	538	LYS	A
284	539	GLN	A

Continued on next page

Table S59 – *Continued from previous page*

Homology index	Residue Index	Residue abbreviation	Module
285	540	THR	A
286	541	VAL	A
287	542	LEU	A
288	552	THR	A
289	553	TRP	A
290	554	LEU	A
291	555	ASP	A
292	556	CYS	A
293	557	LEU	A
294	574	ARG	A
295	575	PRO	A
296	576	TYR	A
297	577	GLN	A
298	578	GLY	A
299	579	MET	A
300	580	ILE	A
301	581	LEU	A
302	582	TRP	A

Table S60: Residues membership for the *N. polysaccharea* α-amylase (PDB code 1ZS2, chain A)

Homology index	Residue Index	Residue abbreviation	Module
0	98	GLN	A
1	99	VAL	A
2	100	GLY	B
3	101	GLY	B
4	102	VAL	B
5	103	CYS	C
6	104	TYR	C
7	105	VAL	C
8	110	GLY	C
9	111	ASP	C
10	112	LEU	C
11	113	LYS	C
12	114	GLY	C
13	115	LEU	C
14	116	LYS	C
15	117	ASP	C
16	118	LYS	C
17	119	ILE	C
18	120	PRO	C
19	121	TYR	C

Continued on next page

Table S60 – *Continued from previous page*

Homology index	Residue Index	Residue abbreviation	Module
20	122	PHE	C
21	123	GLN	C
22	124	GLU	C
23	125	LEU	C
24	126	GLY	C
25	127	LEU	C
26	128	THR	C
27	129	TYR	C
28	130	LEU	C
29	131	TYR	C
30	132	LEU	C
31	133	MET	C
32	134	PRO	C
33	135	LEU	C
34	136	PHE	C
35	137	LYS	C
36	138	CYS	C
37	139	PRO	C
38	141	GLY	C
39	144	ASP	C
40	145	GLY	C
41	146	GLY	C
42	147	TYR	C
43	148	ALA	C
44	149	VAL	C
45	150	SER	C
46	151	SER	C
47	153	ARG	C
48	154	ASP	C
49	155	VAL	C
50	156	ASN	C
51	157	PRO	C
52	158	ALA	C
53	159	LEU	C
54	160	GLY	C
55	161	THR	C
56	162	ILE	C
57	163	GLY	C
58	164	ASP	C
59	165	LEU	C
60	166	ARG	C
61	167	GLU	C
62	168	VAL	C
63	169	ILE	C
64	170	ALA	C
65	171	ALA	C

Continued on next page

Table S60 – *Continued from previous page*

Homology index	Residue Index	Residue abbreviation	Module
66	172	LEU	C
67	173	HIS	C
68	174	GLU	C
69	175	ALA	C
70	176	GLY	C
71	177	ILE	C
72	178	SER	C
73	179	ALA	C
74	180	VAL	C
75	181	VAL	C
76	182	ASP	C
77	183	PHE	C
78	184	ILE	C
79	185	PHE	C
80	186	ASN	C
81	187	HIS	C
82	188	THR	C
83	189	SER	C
84	190	ASN	C
85	191	GLU	C
86	192	HIS	C
87	193	GLU	C
88	194	TRP	C
89	195	ALA	C
90	206	ASP	C
91	207	ASN	C
92	208	PHE	C
93	209	TYR	C
94	210	TYR	B
95	247	TRP	B
96	248	THR	B
97	249	THR	B
98	250	PHE	B
99	254	GLN	B
100	255	TRP	B
101	256	ASP	B
102	257	LEU	B
103	258	ASN	B
104	259	TYR	B
105	260	SER	B
106	261	ASN	B
107	262	PRO	B
108	263	TRP	B
109	264	VAL	B
110	265	PHE	B
111	266	ARG	B

Continued on next page

Table S60 – *Continued from previous page*

Homology index	Residue Index	Residue abbreviation	Module
112	267	ALA	B
113	268	MET	B
114	269	ALA	B
115	270	GLY	B
116	271	GLU	B
117	272	MET	B
118	273	LEU	B
119	274	PHE	B
120	275	LEU	B
121	276	ALA	B
122	277	ASN	B
123	278	LEU	C
124	279	GLY	C
125	280	VAL	C
126	281	ASP	C
127	282	ILE	C
128	283	LEU	B
129	284	ARG	D
130	285	MET	B
131	286	ASP	B
132	287	ALA	B
133	288	VAL	B
134	289	ALA	B
135	290	PHE	B
136	291	ILE	B
137	292	TRP	B
138	306	HIS	B
139	307	ALA	B
140	308	LEU	B
141	309	ILE	B
142	310	ARG	B
143	311	ALA	B
144	312	PHE	B
145	313	ASN	B
146	314	ALA	B
147	315	VAL	B
148	316	MET	B
149	317	ARG	B
150	323	VAL	B
151	324	PHE	B
152	325	PHE	B
153	326	LYS	C
154	327	SER	B
155	328	GLN	B
156	329	ALA	B
157	330	ILE	B

Continued on next page

Table S60 – *Continued from previous page*

Homology index	Residue Index	Residue abbreviation	Module
158	331	VAL	B
159	332	HIS	B
160	336	VAL	B
161	337	VAL	B
162	338	GLN	B
163	339	TYR	B
164	340	ILE	B
165	341	GLY	B
166	345	CYS	B
167	346	GLN	B
168	347	ILE	B
169	348	GLY	B
170	349	TYR	D
171	350	ASN	D
172	351	PRO	D
173	352	LEU	D
174	354	MET	D
175	355	ALA	D
176	356	LEU	D
177	357	LEU	D
178	358	TRP	D
179	359	ASN	D
180	360	THR	D
181	361	LEU	D
182	364	ARG	D
183	365	GLU	A
184	366	VAL	A
185	367	ASN	A
186	372	ALA	A
187	373	LEU	D
188	374	THR	D
189	375	TYR	D
190	376	ARG	D
191	377	HIS	B
192	378	ASN	B
193	381	GLU	B
194	382	HIS	B
195	383	THR	B
196	384	ALA	B
197	385	TRP	B
198	386	VAL	D
199	387	ASN	D
200	388	TYR	D
201	389	VAL	A
202	390	ARG	D
203	391	SER	D

Continued on next page

Table S60 – *Continued from previous page*

Homology index	Residue Index	Residue abbreviation	Module
204	392	HIS	D
205	393	ASP	D
206	394	ASP	D
207	448	SER	D
208	450	THR	D
209	451	ALA	D
210	452	ALA	D
211	453	ALA	D
212	464	ALA	A
213	467	ARG	A
214	468	ILE	A
215	469	LYS	A
216	470	LEU	A
217	471	LEU	A
218	472	TYR	A
219	473	SER	A
220	474	ILE	A
221	475	ALA	A
222	476	LEU	A
223	477	SER	A
224	478	THR	A
225	479	GLY	D
226	480	GLY	D
227	481	LEU	D
228	482	PRO	A
229	483	LEU	D
230	484	ILE	A
231	485	TYR	D
232	486	LEU	C
233	487	GLY	D
234	488	ASP	A
235	514	PRO	C
236	515	ARG	C
237	516	TYR	D
238	517	ASN	D
239	521	TYR	D
240	534	ILE	A
241	535	TYR	A
242	536	GLN	A
243	537	GLY	A
244	538	LEU	A
245	539	ARG	A
246	540	HIS	A
247	541	MET	A
248	542	ILE	A
249	543	ALA	A

Continued on next page

Table S60 – *Continued from previous page*

Homology index	Residue Index	Residue abbreviation	Module
250	544	VAL	A
251	545	ARG	A
252	546	GLN	A
253	547	SER	A
254	548	ASN	A
255	549	PRO	A
256	553	GLY	A
257	554	GLY	A
258	555	ARG	A
259	556	LEU	A
260	558	THR	A
261	559	PHE	A
262	560	ASN	A
263	561	THR	A
264	563	ASN	A
265	564	LYS	A
266	565	HIS	A
267	566	ILE	A
268	567	ILE	A
269	568	GLY	A
270	569	TYR	A
271	570	ILE	A
272	571	ARG	A
273	573	ASN	A
274	574	ALA	A
275	575	LEU	A
276	576	LEU	A
277	577	ALA	A
278	578	PHE	A
279	579	GLY	A
280	580	ASN	A
281	584	TYR	A
282	585	PRO	A
283	586	GLN	A
284	587	THR	A
285	588	VAL	A
286	589	THR	A
287	590	ALA	A
288	599	LYS	A
289	600	ALA	A
290	601	HIS	A
291	602	ASP	A
292	603	LEU	A
293	604	ILE	A
294	617	LEU	A
295	618	GLN	A

Continued on next page

Table S60 – *Continued from previous page*

Homology index	Residue Index	Residue abbreviation	Module
296	619	PRO	A
297	621	GLN	A
298	622	VAL	A
299	623	MET	A
300	624	TRP	A
301	625	LEU	A
302	626	GLU	A

Table S61: Residues membership for the *T. vulgaris R47* α-amylase (PDB code 1WZM, chain A)

Homology index	Residue Index	Residue abbreviation	Module
0	131	ALA	A
1	132	VAL	A
2	133	ILE	B
3	134	TYR	B
4	135	GLN	B
5	136	ILE	C
6	137	PHE	C
7	138	PRO	C
8	170	GLY	C
9	171	ASP	C
10	172	LEU	C
11	173	LYS	C
12	174	GLY	C
13	175	VAL	C
14	176	ILE	C
15	177	ASP	C
16	178	ARG	C
17	179	LEU	C
18	180	PRO	C
19	181	TYR	C
20	182	LEU	C
21	183	GLU	C
22	184	GLU	C
23	185	LEU	C
24	186	GLY	C
25	187	VAL	C
26	188	THR	C
27	189	ALA	C
28	190	LEU	C
29	191	TYR	C
30	192	PHE	C

Continued on next page

Table S61 – *Continued from previous page*

Homology index	Residue Index	Residue abbreviation	Module
31	193	THR	C
32	194	PRO	C
33	195	ILE	C
34	196	PHE	C
35	197	ALA	C
36	198	SER	C
37	199	PRO	C
38	200	SER	C
39	201	HIS	C
40	202	HIS	C
41	203	LYS	C
42	204	TYR	C
43	205	ASP	C
44	206	THR	C
45	207	ALA	C
46	208	ASP	C
47	210	LEU	C
48	211	ALA	C
49	212	ILE	C
50	213	ASP	C
51	214	PRO	C
52	215	GLN	C
53	216	PHE	C
54	217	GLY	C
55	218	ASP	C
56	219	LEU	C
57	220	PRO	C
58	221	THR	C
59	222	PHE	C
60	223	ARG	C
61	224	ARG	C
62	225	LEU	C
63	226	VAL	C
64	227	ASP	C
65	228	GLU	C
66	229	ALA	C
67	230	HIS	C
68	231	ARG	C
69	232	ARG	C
70	233	GLY	C
71	234	ILE	C
72	235	LYS	C
73	236	ILE	C
74	237	ILE	C
75	238	LEU	C
76	239	ASP	C

Continued on next page

Table S61 – *Continued from previous page*

Homology index	Residue Index	Residue abbreviation	Module
77	240	ALA	C
78	241	VAL	C
79	242	PHE	C
80	243	ASN	C
81	244	HIS	C
82	245	ALA	C
83	246	GLY	C
84	247	ASP	C
85	248	GLN	C
86	249	PHE	C
87	250	PHE	C
88	251	ALA	C
89	252	PHE	C
90	265	LYS	C
91	266	ASP	C
92	267	TRP	C
93	268	PHE	C
94	269	PHE	B
95	284	GLU	B
96	285	THR	B
97	286	PHE	B
98	287	ALA	B
99	293	MET	B
100	294	PRO	B
101	295	LYS	B
102	296	LEU	B
103	297	ARG	B
104	298	THR	B
105	299	GLU	B
106	300	ASN	B
107	301	PRO	B
108	302	GLU	B
109	303	VAL	B
110	304	LYS	B
111	305	GLU	B
112	306	TYR	B
113	307	LEU	B
114	308	PHE	B
115	309	ASP	B
116	310	VAL	B
117	311	ALA	B
118	312	ARG	B
119	313	PHE	B
120	314	TRP	B
121	315	MET	B
122	316	GLU	B

Continued on next page

Table S61 – *Continued from previous page*

Homology index	Residue Index	Residue abbreviation	Module
123	317	GLN	C
124	318	GLY	C
125	319	ILE	C
126	320	ASP	C
127	321	GLY	C
128	322	TRP	B
129	323	ARG	D
130	324	LEU	B
131	325	ASP	B
132	326	VAL	B
133	327	ALA	B
134	328	ASN	B
135	329	GLU	B
136	330	VAL	B
137	331	ASP	B
138	332	HIS	B
139	333	ALA	B
140	334	PHE	B
141	335	TRP	B
142	336	ARG	B
143	337	GLU	B
144	338	PHE	B
145	339	ARG	B
146	340	ARG	B
147	341	LEU	B
148	342	VAL	B
149	343	LYS	B
150	349	ALA	B
151	350	LEU	B
152	351	ILE	B
153	352	VAL	C
154	353	GLY	B
155	354	GLU	B
156	355	ILE	B
157	356	TRP	B
158	357	HIS	B
159	358	ASP	B
160	359	ALA	B
161	360	SER	B
162	361	GLY	B
163	362	TRP	B
164	363	LEU	B
165	364	MET	B
166	368	PHE	B
167	369	ASP	B
168	370	SER	B

Continued on next page

Table S61 – *Continued from previous page*

Homology index	Residue Index	Residue abbreviation	Module
169	371	VAL	B
170	372	MET	D
171	373	ASN	D
172	374	TYR	D
173	375	LEU	D
174	377	ARG	D
175	378	GLU	D
176	379	SER	D
177	380	VAL	D
178	381	ILE	D
179	382	ARG	D
180	383	PHE	D
181	384	PHE	D
182	389	ILE	D
183	390	HIS	A
184	391	ALA	A
185	392	GLU	A
186	397	GLU	A
187	398	LEU	D
188	399	THR	D
189	400	ARG	D
190	401	ALA	D
191	402	ARG	B
192	403	MET	B
193	409	ALA	B
194	410	ALA	B
195	411	GLN	B
196	412	GLY	B
197	413	LEU	B
198	414	TRP	D
199	415	ASN	D
200	416	LEU	D
201	417	LEU	A
202	418	ASP	D
203	419	SER	D
204	420	HIS	D
205	421	ASP	D
206	422	THR	D
207	423	GLU	D
208	424	ARG	D
209	425	PHE	D
210	426	LEU	D
211	427	THR	D
212	433	GLU	A
213	435	LYS	A
214	436	PHE	A

Continued on next page

Table S61 – *Continued from previous page*

Homology index	Residue Index	Residue abbreviation	Module
215	437	ARG	A
216	438	LEU	A
217	439	ALA	A
218	440	VAL	A
219	441	LEU	A
220	442	PHE	A
221	443	GLN	A
222	444	MET	A
223	445	THR	A
224	446	TYR	A
225	447	LEU	D
226	448	GLY	D
227	449	THR	D
228	450	PRO	A
229	451	LEU	D
230	452	ILE	A
231	453	TYR	D
232	454	TYR	C
233	455	GLY	D
234	456	ASP	A
235	470	ARG	C
236	471	PRO	C
237	472	MET	D
238	473	ILE	D
239	474	TRP	D
240	483	LEU	A
241	484	PHE	A
242	485	GLU	A
243	486	PHE	A
244	487	TYR	A
245	488	LYS	A
246	489	GLU	A
247	490	LEU	A
248	491	ILE	A
249	492	ARG	A
250	493	LEU	A
251	494	ARG	A
252	495	HIS	A
253	496	ARG	A
254	497	LEU	A
255	498	ALA	A
256	502	ARG	A
257	503	GLY	A
258	504	ASN	A
259	505	VAL	A
260	507	SER	A

Continued on next page

Table S61 – *Continued from previous page*

**Homology index**	**Residue Index**	**Residue abbreviation**	**Module**
261	508	TRP	A
262	509	HIS	A
263	510	ALA	A
264	513	GLN	A
265	514	ALA	A
266	515	ASN	A
267	516	LEU	A
268	517	TYR	A
269	518	ALA	A
270	519	PHE	A
271	520	VAL	A
272	521	ARG	A
273	526	GLN	A
274	527	HIS	A
275	528	VAL	A
276	529	GLY	A
277	530	VAL	A
278	531	VAL	A
279	532	LEU	A
280	533	ASN	A
281	536	GLY	A
282	537	GLU	A
283	538	LYS	A
284	539	GLN	A
285	540	THR	A
286	541	VAL	A
287	542	LEU	A
288	552	THR	A
289	553	TRP	A
290	554	LEU	A
291	555	ASP	A
292	556	CYS	A
293	557	LEU	A
294	574	ARG	A
295	575	PRO	A
296	576	TYR	A
297	577	GLN	A
298	578	GLY	A
299	579	MET	A
300	580	ILE	A
301	581	LEU	A
302	582	TRP	A

Table S62: Residues membership for the *H. sapiens* α-amylase (PDB code 2QMK, chain A)

Homology index	Residue Index	Residue abbreviation	Module
0	11	THR	A
1	12	SER	A
2	13	ILE	B
3	14	VAL	B
4	15	HIS	B
5	16	LEU	C
6	17	PHE	C
7	18	GLU	C
8	19	TRP	C
9	20	ARG	C
10	21	TRP	C
11	22	VAL	C
12	23	ASP	C
13	24	ILE	C
14	25	ALA	C
15	26	LEU	C
16	27	GLU	C
17	28	CYS	C
18	29	GLU	C
19	30	ARG	C
20	32	LEU	C
21	33	ALA	C
22	34	PRO	C
23	35	LYS	C
24	36	GLY	C
25	37	PHE	C
26	38	GLY	C
27	39	GLY	C
28	40	VAL	C
29	41	GLN	C
30	42	VAL	C
31	43	SER	C
32	44	PRO	C
33	45	PRO	C
34	46	ASN	C
35	47	GLU	C
36	48	ASN	C
37	49	VAL	C
38	51	ILE	C
39	59	TRP	C
40	60	GLU	C
41	61	ARG	C
42	62	TYR	C
43	63	GLN	C

Continued on next page

Table S62 – *Continued from previous page*

Homology index	Residue Index	Residue abbreviation	Module
44	64	PRO	C
45	65	VAL	C
46	66	SER	C
47	67	TYR	C
48	68	LYS	C
49	69	LEU	C
50	70	CYS	C
51	71	THR	C
52	72	ARG	C
53	73	SER	C
54	74	GLY	C
55	75	ASN	C
56	76	GLU	C
57	77	ASP	C
58	78	GLU	C
59	79	PHE	C
60	80	ARG	C
61	81	ASN	C
62	82	MET	C
63	83	VAL	C
64	84	THR	C
65	85	ARG	C
66	86	CYS	C
67	87	ASN	C
68	88	ASN	C
69	89	VAL	C
70	90	GLY	C
71	91	VAL	C
72	92	ARG	C
73	93	ILE	C
74	94	TYR	C
75	95	VAL	C
76	96	ASP	C
77	97	ALA	C
78	98	VAL	C
79	99	ILE	C
80	100	ASN	C
81	101	HIS	C
82	102	MET	C
83	103	CYS	C
84	104	GLY	C
85	105	ASN	C
86	126	PHE	C
87	130	PRO	C
88	131	TYR	C
89	132	SER	C

Continued on next page

Table S62 – *Continued from previous page*

Homology index	Residue Index	Residue abbreviation	Module
90	133	GLY	C
91	134	TRP	C
92	135	ASP	C
93	136	PHE	C
94	137	ASN	B
95	160	CYS	B
96	161	ARG	B
97	162	LEU	B
98	163	THR	B
99	165	LEU	B
100	166	LEU	B
101	167	ASP	B
102	168	LEU	B
103	169	ALA	B
104	170	LEU	B
105	171	GLU	B
106	172	LYS	B
107	173	ASP	B
108	174	TYR	B
109	175	VAL	B
110	176	ARG	B
111	177	SER	B
112	178	LYS	B
113	179	ILE	B
114	180	ALA	B
115	181	GLU	B
116	182	TYR	B
117	183	MET	B
118	184	ASN	B
119	185	HIS	B
120	186	LEU	B
121	187	ILE	B
122	188	ASP	B
123	189	ILE	C
124	190	GLY	C
125	191	VAL	C
126	192	ALA	C
127	193	GLY	C
128	194	PHE	B
129	195	ARG	D
130	196	LEU	B
131	197	ASP	B
132	198	ALA	B
133	199	SER	B
134	200	LYS	B
135	201	HIS	B

Continued on next page

Table S62 – *Continued from previous page*

Homology index	Residue Index	Residue abbreviation	Module
136	202	MET	B
137	203	TRP	B
138	204	PRO	B
139	205	GLY	B
140	206	ASP	B
141	207	ILE	B
142	208	LYS	B
143	209	ALA	B
144	210	ILE	B
145	211	LEU	B
146	212	ASP	B
147	213	LYS	B
148	214	LEU	B
149	215	HIS	B
150	228	PRO	B
151	229	PHE	B
152	230	ILE	B
153	231	TYR	C
154	232	GLN	B
155	233	GLU	B
156	234	VAL	B
157	235	ILE	B
158	236	ASP	B
159	237	LEU	B
160	244	SER	B
161	245	SER	B
162	246	ASP	B
163	247	TYR	B
164	248	PHE	B
165	249	GLY	B
166	250	ASN	B
167	251	GLY	B
168	252	ARG	B
169	253	VAL	B
170	254	THR	D
171	255	GLU	D
172	256	PHE	D
173	257	LYS	D
174	259	GLY	D
175	260	ALA	D
176	261	LYS	D
177	262	LEU	D
178	263	GLY	D
179	264	THR	D
180	265	VAL	D
181	266	ILE	D

Continued on next page

Table S62 – *Continued from previous page*

Homology index	Residue Index	Residue abbreviation	Module
182	272	GLU	D
183	273	LYS	A
184	274	MET	A
185	275	SER	A
186	280	TRP	A
187	281	GLY	D
188	282	GLU	D
189	283	GLY	D
190	285	GLY	D
191	286	PHE	B
192	287	VAL	B
193	288	PRO	B
194	289	SER	B
195	290	ASP	B
196	291	ARG	B
197	292	ALA	B
198	293	LEU	D
199	294	VAL	D
200	295	PHE	D
201	296	VAL	A
202	297	ASP	D
203	298	ASN	D
204	299	HIS	D
205	300	ASP	D
206	301	ASN	D
207	302	GLN	D
208	314	THR	D
209	315	PHE	D
210	316	TRP	D
211	317	ASP	D
212	319	ARG	A
213	320	LEU	A
214	321	TYR	A
215	322	LYS	A
216	323	MET	A
217	324	ALA	A
218	325	VAL	A
219	326	GLY	A
220	327	PHE	A
221	328	MET	A
222	329	LEU	A
223	330	ALA	A
224	331	HIS	A
225	333	TYR	D
226	334	GLY	D
227	335	PHE	D

Continued on next page

Table S62 – *Continued from previous page*

Homology index	Residue Index	Residue abbreviation	Module
228	336	THR	A
229	337	ARG	D
230	338	VAL	A
231	339	MET	D
232	340	SER	C
233	341	SER	D
234	342	TYR	A
235	343	ARG	C
236	381	ASP	C
237	382	TRP	D
238	383	VAL	D
239	384	CYS	D
240	387	ARG	A
241	388	TRP	A
242	389	ARG	A
243	390	GLN	A
244	391	ILE	A
245	392	ARG	A
246	393	ASN	A
247	394	MET	A
248	395	VAL	A
249	396	ILE	A
250	397	PHE	A
251	398	ARG	A
252	399	ASN	A
253	400	VAL	A
254	401	VAL	A
255	402	ASP	A
256	403	GLY	A
257	404	GLN	A
258	405	PRO	A
259	406	PHE	A
260	408	ASN	A
261	409	TRP	A
262	410	TYR	A
263	411	ASP	A
264	413	GLY	A
265	414	SER	A
266	415	ASN	A
267	416	GLN	A
268	417	VAL	A
269	418	ALA	A
270	419	PHE	A
271	420	GLY	A
272	421	ARG	A
273	422	GLY	A

Continued on next page

Table S62 – *Continued from previous page*

Homology index	Residue Index	Residue abbreviation	Module
274	423	ASN	A
275	425	GLY	A
276	426	PHE	A
277	427	ILE	A
278	428	VAL	A
279	429	PHE	A
280	430	ASN	A
281	433	ASP	A
282	434	TRP	A
283	435	SER	A
284	436	PHE	A
285	437	SER	A
286	438	LEU	A
287	439	THR	A
288	445	PRO	A
289	446	ALA	A
290	449	TYR	A
291	450	CYS	A
292	451	ASP	A
293	452	VAL	A
294	479	ILE	A
295	480	SER	A
296	481	ASN	A
297	486	PRO	A
298	487	PHE	A
299	488	ILE	A
300	489	ALA	A
301	490	ILE	A
302	491	HIS	A

Table S63: Residues membership for the *A.niger* α-amylase (PDB code 2AAA, chain A)

Homology index	Residue Index	Residue abbreviation	Module
0	9	GLN	A
1	10	SER	A
2	11	ILE	B
3	12	TYR	B
4	13	PHE	B
5	14	LEU	C
6	15	LEU	C
7	16	THR	C
8	40	GLY	C

Continued on next page

Table S63 – *Continued from previous page*

Homology index	Residue Index	Residue abbreviation	Module
9	41	SER	C
10	42	TRP	C
11	43	GLN	C
12	44	GLY	C
13	45	ILE	C
14	46	ILE	C
15	47	ASP	C
16	48	HIS	C
17	49	LEU	C
18	50	ASP	C
19	51	TYR	C
20	52	ILE	C
21	53	GLU	C
22	54	GLY	C
23	55	MET	C
24	56	GLY	C
25	57	PHE	C
26	58	THR	C
27	59	ALA	C
28	60	ILE	C
29	61	TRP	C
30	62	ILE	C
31	63	SER	C
32	64	PRO	C
33	65	ILE	C
34	66	THR	C
35	67	GLU	C
36	68	GLN	C
37	69	LEU	C
38	77	GLU	C
39	79	TYR	C
40	80	HIS	C
41	81	GLY	C
42	82	TYR	C
43	83	TRP	C
44	84	GLN	C
45	85	GLN	C
46	86	LYS	C
47	88	TYR	C
48	89	ASP	C
49	90	VAL	C
50	91	ASN	C
51	92	SER	C
52	93	ASN	C
53	94	PHE	C
54	95	GLY	C

Continued on next page

Table S63 – *Continued from previous page*

Homology index	Residue Index	Residue abbreviation	Module
55	96	THR	C
56	97	ALA	C
57	98	ASP	C
58	99	ASN	C
59	100	LEU	C
60	101	LYS	C
61	102	SER	C
62	103	LEU	C
63	104	SER	C
64	105	ASP	C
65	106	ALA	C
66	107	LEU	C
67	108	HIS	C
68	109	ALA	C
69	110	ARG	C
70	111	GLY	C
71	112	MET	C
72	113	TYR	C
73	114	LEU	C
74	115	MET	C
75	116	VAL	C
76	117	ASP	C
77	118	VAL	C
78	119	VAL	C
79	120	PRO	C
80	121	ASP	C
81	122	HIS	C
82	123	MET	C
83	124	GLY	C
84	125	TYR	C
85	126	ALA	C
86	137	PHE	C
87	139	PRO	C
88	140	PHE	C
89	141	ASP	C
90	143	SER	C
91	144	SER	C
92	145	TYR	C
93	146	PHE	C
94	147	HIS	B
95	164	CYS	B
96	165	TRP	B
97	166	GLU	B
98	167	GLY	B
99	173	LEU	B
100	174	PRO	B

Continued on next page

Table S63 – *Continued from previous page*

Homology index	Residue Index	Residue abbreviation	Module
101	175	ASP	B
102	176	LEU	B
103	177	ASP	B
104	178	THR	B
105	179	THR	B
106	180	GLU	B
107	181	THR	B
108	182	ALA	B
109	183	VAL	B
110	184	ARG	B
111	185	THR	B
112	186	ILE	B
113	187	TRP	B
114	188	TYR	B
115	189	ASP	B
116	190	TRP	B
117	191	VAL	B
118	192	ALA	B
119	193	ASP	B
120	194	LEU	B
121	195	VAL	B
122	196	SER	B
123	198	TYR	C
124	199	SER	C
125	200	VAL	C
126	201	ASP	C
127	202	GLY	C
128	203	LEU	B
129	204	ARG	D
130	205	ILE	B
131	206	ASP	B
132	207	SER	B
133	208	VAL	B
134	209	LEU	B
135	210	GLU	B
136	211	VAL	B
137	212	GLN	B
138	213	PRO	B
139	214	ASP	B
140	215	PHE	B
141	216	PHE	B
142	217	PRO	B
143	218	GLY	B
144	219	TYR	B
145	220	ASN	B
146	221	LYS	B

Continued on next page

Table S63 – *Continued from previous page*

Homology index	Residue Index	Residue abbreviation	Module
147	222	ALA	B
148	223	SER	B
149	224	GLY	B
150	225	VAL	B
151	226	TYR	B
152	227	CYS	B
153	228	VAL	C
154	229	GLY	B
155	230	GLU	B
156	231	ILE	B
157	232	ASP	B
158	233	ASN	B
159	234	GLY	B
160	239	ASP	B
161	240	CYS	B
162	241	PRO	B
163	242	TYR	B
164	243	GLN	B
165	244	LYS	B
166	246	LEU	B
167	247	ASP	B
168	248	GLY	B
169	249	VAL	B
170	250	LEU	D
171	251	ASN	D
172	252	TYR	D
173	253	PRO	D
174	255	TYR	D
175	256	TRP	D
176	257	GLN	D
177	258	LEU	D
178	259	LEU	D
179	260	TYR	D
180	261	ALA	D
181	262	PHE	D
182	267	GLY	D
183	268	SER	A
184	269	ILE	A
185	270	SER	A
186	275	MET	A
187	276	ILE	D
188	277	LYS	D
189	278	SER	D
190	279	VAL	D
191	280	ALA	B
192	281	SER	B

Continued on next page

Table S63 – *Continued from previous page*

Homology index	Residue Index	Residue abbreviation	Module
193	285	ASP	B
194	286	PRO	B
195	287	THR	B
196	288	LEU	B
197	289	LEU	B
198	290	GLY	D
199	291	ASN	D
200	292	PHE	D
201	293	ILE	A
202	294	GLU	D
203	295	ASN	D
204	296	HIS	D
205	297	ASP	D
206	298	ASN	D
207	299	PRO	D
208	300	ARG	D
209	301	PHE	D
210	302	ALA	D
211	303	LYS	D
212	308	TYR	A
213	310	GLN	A
214	311	ALA	A
215	312	LYS	A
216	313	ASN	A
217	314	VAL	A
218	315	LEU	A
219	316	SER	A
220	317	TYR	A
221	318	ILE	A
222	319	PHE	A
223	320	LEU	A
224	321	SER	A
225	322	ASP	D
226	323	GLY	D
227	324	ILE	D
228	325	PRO	A
229	326	ILE	D
230	327	VAL	A
231	328	TYR	D
232	329	ALA	C
233	330	GLY	D
234	331	GLU	A
235	345	GLU	C
236	346	ALA	C
237	347	THR	D
238	348	TRP	D

Continued on next page

Table S63 – *Continued from previous page*

Homology index	Residue Index	Residue abbreviation	Module
239	352	TYR	D
240	358	LEU	A
241	359	TYR	A
242	360	THR	A
243	361	TRP	A
244	362	ILE	A
245	363	ALA	A
246	364	THR	A
247	365	THR	A
248	366	ASN	A
249	367	ALA	A
250	368	ILE	A
251	373	ILE	A
252	374	ALA	A
253	375	ALA	A
254	376	ASP	A
255	377	SER	A
256	381	THR	A
257	382	TYR	A
258	383	ALA	A
259	384	ASN	A
260	386	ALA	A
261	387	PHE	A
262	388	TYR	A
263	389	THR	A
264	390	ASP	A
265	391	SER	A
266	392	ASN	A
267	393	THR	A
268	394	ILE	A
269	395	ALA	A
270	396	MET	A
271	397	ALA	A
272	398	LYS	A
273	403	SER	A
274	404	GLN	A
275	405	VAL	A
276	406	ILE	A
277	407	THR	A
278	408	VAL	A
279	409	LEU	A
280	410	SER	A
281	416	GLY	A
282	417	SER	A
283	418	SER	A
284	419	TYR	A

Continued on next page

Table S63 – *Continued from previous page*

**Homology index**	**Residue Index**	**Residue abbreviation**	**Module**
285	420	THR	A
286	421	LEU	A
287	422	THR	A
288	433	LYS	A
289	434	LEU	A
290	435	ILE	A
291	436	GLU	A
292	437	ALA	A
293	438	TYR	A
294	456	ALA	A
295	457	SER	A
296	458	GLY	A
297	459	LEU	A
298	460	PRO	A
299	461	ARG	A
300	462	VAL	A
301	463	LEU	A
302	464	LEU	A

Table S64: Residues membership for the *A. oryzae* α-amylase (PDB code 2TAA, chain A)

**Homology index**	**Residue Index**	**Residue abbreviation**	**Module**
0	9	GLN	A
1	10	SER	A
2	11	ILE	B
3	12	TYR	B
4	13	PHE	B
5	14	LEU	C
6	15	LEU	C
7	16	THR	C
8	40	GLY	C
9	41	THR	C
10	42	TRP	C
11	43	GLN	C
12	44	GLY	C
13	45	ILE	C
14	46	ILE	C
15	47	ASP	C
16	48	LYS	C
17	49	LEU	C
18	50	ASP	C
19	51	TYR	C

Continued on next page

Table S64 – *Continued from previous page*

Homology index	Residue Index	Residue abbreviation	Module
20	52	ILE	C
21	53	GLN	C
22	54	GLY	C
23	55	MET	C
24	56	GLY	C
25	57	PHE	C
26	58	THR	C
27	59	ALA	C
28	60	ILE	C
29	61	TRP	C
30	62	ILE	C
31	63	THR	C
32	64	PRO	C
33	65	VAL	C
34	66	THR	C
35	67	ALA	C
36	68	GLN	C
37	69	LEU	C
38	77	ASP	C
39	79	TYR	C
40	80	THR	C
41	81	GLY	C
42	82	TYR	C
43	83	TRP	C
44	84	GLN	C
45	85	THR	C
46	86	ASP	C
47	88	TYR	C
48	89	SER	C
49	90	LEU	C
50	91	ASN	C
51	92	GLU	C
52	93	ASN	C
53	94	TYR	C
54	95	GLY	C
55	96	THR	C
56	97	ALA	C
57	98	ASP	C
58	99	ASP	C
59	100	LEU	C
60	101	LYS	C
61	102	ALA	C
62	103	LEU	C
63	104	SER	C
64	105	SER	C
65	106	ALA	C

Continued on next page

Table S64 – *Continued from previous page*

Homology index	Residue Index	Residue abbreviation	Module
66	107	LEU	C
67	108	HIS	C
68	109	GLU	C
69	110	ARG	C
70	111	GLY	C
71	112	MET	C
72	113	TYR	C
73	114	LEU	C
74	115	MET	C
75	116	VAL	C
76	117	ASP	C
77	118	VAL	C
78	119	VAL	C
79	120	ALA	C
80	121	ASN	C
81	122	HIS	C
82	123	MET	C
83	124	GLY	C
84	125	TYR	C
85	126	ASP	C
86	137	PHE	C
87	139	PRO	C
88	140	PHE	C
89	141	SER	C
90	143	GLN	C
91	144	ASP	C
92	145	TYR	C
93	146	PHE	C
94	147	HIS	B
95	164	CYS	B
96	165	TRP	B
97	166	LEU	B
98	167	GLY	B
99	173	LEU	B
100	174	PRO	B
101	175	ASP	B
102	176	LEU	B
103	177	ASP	B
104	178	THR	B
105	179	THR	B
106	180	LYS	B
107	181	ASP	B
108	182	VAL	B
109	183	VAL	B
110	184	LYS	B
111	185	ASN	B

Continued on next page

Table S64 – *Continued from previous page*

Homology index	Residue Index	Residue abbreviation	Module
112	186	GLU	B
113	187	TRP	B
114	188	TYR	B
115	189	ASP	B
116	190	TRP	B
117	191	VAL	B
118	192	GLY	B
119	193	SER	B
120	194	LEU	B
121	195	VAL	B
122	196	SER	B
123	198	TYR	C
124	199	SER	C
125	200	ILE	C
126	201	ASP	C
127	202	GLY	C
128	203	LEU	B
129	204	ARG	D
130	205	ILE	B
131	206	ASP	B
132	207	THR	B
133	208	VAL	B
134	209	LYS	B
135	210	HIS	B
136	211	VAL	B
137	212	GLN	B
138	213	LYS	B
139	214	ASP	B
140	215	PHE	B
141	216	TRP	B
142	217	PRO	B
143	218	GLY	B
144	219	TYR	B
145	220	ASN	B
146	221	LYS	B
147	222	ALA	B
148	223	ALA	B
149	224	GLY	B
150	225	VAL	B
151	226	TYR	B
152	227	CYS	B
153	228	ILE	C
154	229	GLY	B
155	230	GLU	B
156	231	VAL	B
157	232	LEU	B

Continued on next page

Table S64 – *Continued from previous page*

Homology index	Residue Index	Residue abbreviation	Module
158	233	ASP	B
159	234	GLY	B
160	239	THR	B
161	240	CYS	B
162	241	PRO	B
163	242	TYR	B
164	243	GLN	B
165	244	ASN	B
166	246	MET	B
167	247	ASP	B
168	248	GLY	B
169	249	VAL	B
170	250	LEU	D
171	251	ASN	D
172	252	TYR	D
173	253	PRO	D
174	255	TYR	D
175	256	TYR	D
176	257	PRO	D
177	258	LEU	D
178	259	LEU	D
179	260	ASN	D
180	261	ALA	D
181	262	PHE	D
182	267	GLY	D
183	268	SER	A
184	269	MET	A
185	270	ASP	A
186	275	MET	A
187	276	ILE	D
188	277	ASN	D
189	278	THR	D
190	279	VAL	D
191	280	LYS	B
192	281	SER	B
193	285	ASP	B
194	286	SER	B
195	287	THR	B
196	288	LEU	B
197	289	LEU	B
198	290	GLY	D
199	291	THR	D
200	292	PHE	D
201	293	VAL	A
202	294	GLU	D
203	295	ASN	D

Continued on next page

Table S64 – *Continued from previous page*

Homology index	Residue Index	Residue abbreviation	Module
204	296	HIS	D
205	297	ASP	D
206	298	ASN	D
207	299	PRO	D
208	300	ARG	D
209	301	PHE	D
210	302	ALA	D
211	303	SER	D
212	308	ILE	A
213	310	LEU	A
214	311	ALA	A
215	312	LYS	A
216	313	ASN	A
217	314	VAL	A
218	315	ALA	A
219	316	ALA	A
220	317	PHE	A
221	318	ILE	A
222	319	ILE	A
223	320	LEU	A
224	321	ASN	A
225	322	ASP	D
226	323	GLY	D
227	324	LEU	D
228	325	PRO	A
229	326	ILE	D
230	327	ILE	A
231	328	TYR	D
232	329	ALA	C
233	330	GLY	D
234	331	GLN	A
235	345	GLU	C
236	346	ALA	C
237	347	THR	D
238	348	TRP	D
239	352	TYR	D
240	358	LEU	A
241	359	TYR	A
242	360	LYS	A
243	361	LEU	A
244	362	ILE	A
245	363	ALA	A
246	364	SER	A
247	365	ALA	A
248	366	ASN	A
249	367	ALA	A

Continued on next page

Table S64 – *Continued from previous page*

Homology index	Residue Index	Residue abbreviation	Module
250	368	ILE	A
251	373	ILE	A
252	374	SER	A
253	375	LYS	A
254	376	ASP	A
255	377	THR	A
256	381	THR	A
257	382	TYR	A
258	383	LYS	A
259	384	ASN	A
260	385	PRO	A
261	386	TYR	A
262	387	ILE	A
263	388	LYS	A
264	389	ASP	A
265	390	ASP	A
266	391	THR	A
267	392	THR	A
268	393	ILE	A
269	394	ALA	A
270	395	MET	A
271	396	ARG	A
272	397	LYS	A
273	403	GLN	A
274	404	ILE	A
275	405	VAL	A
276	406	THR	A
277	407	ILE	A
278	408	LEU	A
279	409	SER	A
280	410	ASN	A
281	415	GLY	A
282	416	ASP	A
283	417	SER	A
284	418	TYR	A
285	419	THR	A
286	420	LEU	A
287	421	SER	A
288	431	GLN	A
289	432	GLN	A
290	434	THR	A
291	435	GLU	A
292	436	VAL	A
293	437	ILE	A
294	454	MET	A
295	455	ALA	A

Continued on next page

Table S64 – *Continued from previous page*

Homology index	Residue Index	Residue abbreviation	Module
296	456	GLY	A
297	458	LEU	A
298	459	PRO	A
299	460	ARG	A
300	461	VAL	A
301	462	LEU	A
302	463	TYR	A

Table S65: Residues membership for the *H. sapiens* α-amylase (PDB code 2QV4, chain A)

Homology index	Residue Index	Residue abbreviation	Module
0	11	THR	A
1	12	SER	A
2	13	ILE	B
3	14	VAL	B
4	15	HIS	B
5	16	LEU	C
6	17	PHE	C
7	18	GLU	C
8	19	TRP	C
9	20	ARG	C
10	21	TRP	C
11	22	VAL	C
12	23	ASP	C
13	24	ILE	C
14	25	ALA	C
15	26	LEU	C
16	27	GLU	C
17	28	CYS	C
18	29	GLU	C
19	30	ARG	C
20	32	LEU	C
21	33	ALA	C
22	34	PRO	C
23	35	LYS	C
24	36	GLY	C
25	37	PHE	C
26	38	GLY	C
27	39	GLY	C
28	40	VAL	C
29	41	GLN	C
30	42	VAL	C

Continued on next page

Table S65 – *Continued from previous page*

Homology index	Residue Index	Residue abbreviation	Module
31	43	SER	C
32	44	PRO	C
33	45	PRO	C
34	46	ASN	C
35	47	GLU	C
36	48	ASN	C
37	49	VAL	C
38	51	ILE	C
39	59	TRP	C
40	60	GLU	C
41	61	ARG	C
42	62	TYR	C
43	63	GLN	C
44	64	PRO	C
45	65	VAL	C
46	66	SER	C
47	67	TYR	C
48	68	LYS	C
49	69	LEU	C
50	70	CYS	C
51	71	THR	C
52	72	ARG	C
53	73	SER	C
54	74	GLY	C
55	75	ASN	C
56	76	GLU	C
57	77	ASP	C
58	78	GLU	C
59	79	PHE	C
60	80	ARG	C
61	81	ASN	C
62	82	MET	C
63	83	VAL	C
64	84	THR	C
65	85	ARG	C
66	86	CYS	C
67	87	ASN	C
68	88	ASN	C
69	89	VAL	C
70	90	GLY	C
71	91	VAL	C
72	92	ARG	C
73	93	ILE	C
74	94	TYR	C
75	95	VAL	C
76	96	ASP	C

Continued on next page

Table S65 – *Continued from previous page*

Homology index	Residue Index	Residue abbreviation	Module
77	97	ALA	C
78	98	VAL	C
79	99	ILE	C
80	100	ASN	C
81	101	HIS	C
82	102	MET	C
83	103	CYS	C
84	104	GLY	C
85	105	ASN	C
86	126	PHE	C
87	130	PRO	C
88	131	TYR	C
89	132	SER	C
90	133	GLY	C
91	134	TRP	C
92	135	ASP	C
93	136	PHE	C
94	137	ASN	B
95	160	CYS	B
96	161	ARG	B
97	162	LEU	B
98	163	THR	B
99	165	LEU	B
100	166	LEU	B
101	167	ASP	B
102	168	LEU	B
103	169	ALA	B
104	170	LEU	B
105	171	GLU	B
106	172	LYS	B
107	173	ASP	B
108	174	TYR	B
109	175	VAL	B
110	176	ARG	B
111	177	SER	B
112	178	LYS	B
113	179	ILE	B
114	180	ALA	B
115	181	GLU	B
116	182	TYR	B
117	183	MET	B
118	184	ASN	B
119	185	HIS	B
120	186	LEU	B
121	187	ILE	B
122	188	ASP	B

Continued on next page

Table S65 – *Continued from previous page*

Homology index	Residue Index	Residue abbreviation	Module
123	189	ILE	C
124	190	GLY	C
125	191	VAL	C
126	192	ALA	C
127	193	GLY	C
128	194	PHE	B
129	195	ARG	D
130	196	LEU	B
131	197	ASP	B
132	198	ALA	B
133	199	SER	B
134	200	LYS	B
135	201	HIS	B
136	202	MET	B
137	203	TRP	B
138	204	PRO	B
139	205	GLY	B
140	206	ASP	B
141	207	ILE	B
142	208	LYS	B
143	209	ALA	B
144	210	ILE	B
145	211	LEU	B
146	212	ASP	B
147	213	LYS	B
148	214	LEU	B
149	215	HIS	B
150	228	PRO	B
151	229	PHE	B
152	230	ILE	B
153	231	TYR	C
154	232	GLN	B
155	233	GLU	B
156	234	VAL	B
157	235	ILE	B
158	236	ASP	B
159	237	LEU	B
160	244	SER	B
161	245	SER	B
162	246	ASP	B
163	247	TYR	B
164	248	PHE	B
165	249	GLY	B
166	250	ASN	B
167	251	GLY	B
168	252	ARG	B

Continued on next page

Table S65 – *Continued from previous page*

Homology index	Residue Index	Residue abbreviation	Module
169	253	VAL	B
170	254	THR	D
171	255	GLU	D
172	256	PHE	D
173	257	LYS	D
174	259	GLY	D
175	260	ALA	D
176	261	LYS	D
177	262	LEU	D
178	263	GLY	D
179	264	THR	D
180	265	VAL	D
181	266	ILE	D
182	272	GLU	D
183	273	LYS	A
184	274	MET	A
185	275	SER	A
186	280	TRP	A
187	281	GLY	D
188	282	GLU	D
189	283	GLY	D
190	285	GLY	D
191	286	PHE	B
192	287	VAL	B
193	288	PRO	B
194	289	SER	B
195	290	ASP	B
196	291	ARG	B
197	292	ALA	B
198	293	LEU	D
199	294	VAL	D
200	295	PHE	D
201	296	VAL	A
202	297	ASP	D
203	298	ASN	D
204	299	HIS	D
205	300	ASP	D
206	301	ASN	D
207	302	GLN	D
208	314	THR	D
209	315	PHE	D
210	316	TRP	D
211	317	ASP	D
212	319	ARG	A
213	320	LEU	A
214	321	TYR	A

Continued on next page

Table S65 – *Continued from previous page*

Homology index	Residue Index	Residue abbreviation	Module
215	322	LYS	A
216	323	MET	A
217	324	ALA	A
218	325	VAL	A
219	326	GLY	A
220	327	PHE	A
221	328	MET	A
222	329	LEU	A
223	330	ALA	A
224	331	HIS	A
225	333	TYR	D
226	334	GLY	D
227	335	PHE	D
228	336	THR	A
229	337	ARG	D
230	338	VAL	A
231	339	MET	D
232	340	SER	C
233	341	SER	D
234	342	TYR	A
235	343	ARG	C
236	381	ASP	C
237	382	TRP	D
238	383	VAL	D
239	384	CYS	D
240	387	ARG	A
241	388	TRP	A
242	389	ARG	A
243	390	GLN	A
244	391	ILE	A
245	392	ARG	A
246	393	ASN	A
247	394	MET	A
248	395	VAL	A
249	396	ILE	A
250	397	PHE	A
251	398	ARG	A
252	399	ASN	A
253	400	VAL	A
254	401	VAL	A
255	402	ASP	A
256	403	GLY	A
257	404	GLN	A
258	405	PRO	A
259	406	PHE	A
260	408	ASN	A

Continued on next page

Table S65 – *Continued from previous page*

Homology index	Residue Index	Residue abbreviation	Module
261	409	TRP	A
262	410	TYR	A
263	411	ASP	A
264	413	GLY	A
265	414	SER	A
266	415	ASN	A
267	416	GLN	A
268	417	VAL	A
269	418	ALA	A
270	419	PHE	A
271	420	GLY	A
272	421	ARG	A
273	422	GLY	A
274	423	ASN	A
275	425	GLY	A
276	426	PHE	A
277	427	ILE	A
278	428	VAL	A
279	429	PHE	A
280	430	ASN	A
281	433	ASP	A
282	434	TRP	A
283	435	SER	A
284	436	PHE	A
285	437	SER	A
286	438	LEU	A
287	439	THR	A
288	445	PRO	A
289	446	ALA	A
290	449	TYR	A
291	450	CYS	A
292	451	ASP	A
293	452	VAL	A
294	479	ILE	A
295	480	SER	A
296	481	ASN	A
297	486	PRO	A
298	487	PHE	A
299	488	ILE	A
300	489	ALA	A
301	490	ILE	A
302	491	HIS	A

Table S66: Residues membership for the *H. sapiens* α-amylase (PDB code 3BAJ, chain A)

Homology index	Residue Index	Residue abbreviation	Module
0	11	THR	A
1	12	SER	A
2	13	ILE	B
3	14	VAL	B
4	15	HIS	B
5	16	LEU	C
6	17	PHE	C
7	18	GLU	C
8	19	TRP	C
9	20	ARG	C
10	21	TRP	C
11	22	VAL	C
12	23	ASP	C
13	24	ILE	C
14	25	ALA	C
15	26	LEU	C
16	27	GLU	C
17	28	CYS	C
18	29	GLU	C
19	30	ARG	C
20	32	LEU	C
21	33	ALA	C
22	34	PRO	C
23	35	LYS	C
24	36	GLY	C
25	37	PHE	C
26	38	GLY	C
27	39	GLY	C
28	40	VAL	C
29	41	GLN	C
30	42	VAL	C
31	43	SER	C
32	44	PRO	C
33	45	PRO	C
34	46	ASN	C
35	47	GLU	C
36	48	ASN	C
37	49	VAL	C
38	51	ILE	C
39	59	TRP	C
40	60	GLU	C
41	61	ARG	C
42	62	TYR	C
43	63	GLN	C

Continued on next page

Table S66 – *Continued from previous page*

Homology index	Residue Index	Residue abbreviation	Module
44	64	PRO	C
45	65	VAL	C
46	66	SER	C
47	67	TYR	C
48	68	LYS	C
49	69	LEU	C
50	70	CYS	C
51	71	THR	C
52	72	ARG	C
53	73	SER	C
54	74	GLY	C
55	75	ASN	C
56	76	GLU	C
57	77	ASP	C
58	78	GLU	C
59	79	PHE	C
60	80	ARG	C
61	81	ASN	C
62	82	MET	C
63	83	VAL	C
64	84	THR	C
65	85	ARG	C
66	86	CYS	C
67	87	ASN	C
68	88	ASN	C
69	89	VAL	C
70	90	GLY	C
71	91	VAL	C
72	92	ARG	C
73	93	ILE	C
74	94	TYR	C
75	95	VAL	C
76	96	ASP	C
77	97	ALA	C
78	98	VAL	C
79	99	ILE	C
80	100	ASN	C
81	101	HIS	C
82	102	MET	C
83	103	CYS	C
84	104	GLY	C
85	105	ASN	C
86	126	PHE	C
87	130	PRO	C
88	131	TYR	C
89	132	SER	C

Continued on next page

Table S66 – *Continued from previous page*

Homology index	Residue Index	Residue abbreviation	Module
90	133	GLY	C
91	134	TRP	C
92	135	ASP	C
93	136	PHE	C
94	137	ASN	B
95	160	CYS	B
96	161	ARG	B
97	162	LEU	B
98	163	THR	B
99	165	LEU	B
100	166	LEU	B
101	167	ASP	B
102	168	LEU	B
103	169	ALA	B
104	170	LEU	B
105	171	GLU	B
106	172	LYS	B
107	173	ASP	B
108	174	TYR	B
109	175	VAL	B
110	176	ARG	B
111	177	SER	B
112	178	LYS	B
113	179	ILE	B
114	180	ALA	B
115	181	GLU	B
116	182	TYR	B
117	183	MET	B
118	184	ASN	B
119	185	HIS	B
120	186	LEU	B
121	187	ILE	B
122	188	ASP	B
123	189	ILE	C
124	190	GLY	C
125	191	VAL	C
126	192	ALA	C
127	193	GLY	C
128	194	PHE	B
129	195	ARG	D
130	196	LEU	B
131	197	ASP	B
132	198	ALA	B
133	199	SER	B
134	200	LYS	B
135	201	HIS	B

Continued on next page

Table S66 – *Continued from previous page*

Homology index	Residue Index	Residue abbreviation	Module
136	202	MET	B
137	203	TRP	B
138	204	PRO	B
139	205	GLY	B
140	206	ASP	B
141	207	ILE	B
142	208	LYS	B
143	209	ALA	B
144	210	ILE	B
145	211	LEU	B
146	212	ASP	B
147	213	LYS	B
148	214	LEU	B
149	215	HIS	B
150	228	PRO	B
151	229	PHE	B
152	230	ILE	B
153	231	TYR	C
154	232	GLN	B
155	233	GLU	B
156	234	VAL	B
157	235	ILE	B
158	236	ASP	B
159	237	LEU	B
160	244	SER	B
161	245	SER	B
162	246	ASP	B
163	247	TYR	B
164	248	PHE	B
165	249	GLY	B
166	250	ASN	B
167	251	GLY	B
168	252	ARG	B
169	253	VAL	B
170	254	THR	D
171	255	GLU	D
172	256	PHE	D
173	257	LYS	D
174	259	GLY	D
175	260	ALA	D
176	261	LYS	D
177	262	LEU	D
178	263	GLY	D
179	264	THR	D
180	265	VAL	D
181	266	ILE	D

Continued on next page

Table S66 – *Continued from previous page*

Homology index	Residue Index	Residue abbreviation	Module
182	272	GLU	D
183	273	LYS	A
184	274	MET	A
185	275	SER	A
186	280	TRP	A
187	281	GLY	D
188	282	GLU	D
189	283	GLY	D
190	285	GLY	D
191	286	PHE	B
192	287	VAL	B
193	288	PRO	B
194	289	SER	B
195	290	ASP	B
196	291	ARG	B
197	292	ALA	B
198	293	LEU	D
199	294	VAL	D
200	295	PHE	D
201	296	VAL	A
202	297	ASP	D
203	298	ASN	D
204	299	HIS	D
205	300	ASP	D
206	301	ASN	D
207	302	GLN	D
208	314	THR	D
209	315	PHE	D
210	316	TRP	D
211	317	ASP	D
212	319	ARG	A
213	320	LEU	A
214	321	TYR	A
215	322	LYS	A
216	323	MET	A
217	324	ALA	A
218	325	VAL	A
219	326	GLY	A
220	327	PHE	A
221	328	MET	A
222	329	LEU	A
223	330	ALA	A
224	331	HIS	A
225	333	TYR	D
226	334	GLY	D
227	335	PHE	D

Continued on next page

Table S66 – *Continued from previous page*

Homology index	Residue Index	Residue abbreviation	Module
228	336	THR	A
229	337	ARG	D
230	338	VAL	A
231	339	MET	D
232	340	SER	C
233	341	SER	D
234	342	TYR	A
235	343	ARG	C
236	381	ASP	C
237	382	TRP	D
238	383	VAL	D
239	384	CYS	D
240	387	ARG	A
241	388	TRP	A
242	389	ARG	A
243	390	GLN	A
244	391	ILE	A
245	392	ARG	A
246	393	ASN	A
247	394	MET	A
248	395	VAL	A
249	396	ILE	A
250	397	PHE	A
251	398	ARG	A
252	399	ASN	A
253	400	VAL	A
254	401	VAL	A
255	402	ASP	A
256	403	GLY	A
257	404	GLN	A
258	405	PRO	A
259	406	PHE	A
260	408	ASN	A
261	409	TRP	A
262	410	TYR	A
263	411	ASP	A
264	413	GLY	A
265	414	SER	A
266	415	ASN	A
267	416	GLN	A
268	417	VAL	A
269	418	ALA	A
270	419	PHE	A
271	420	GLY	A
272	421	ARG	A
273	422	GLY	A

Continued on next page

Table S66 – *Continued from previous page*

Homology index	Residue Index	Residue abbreviation	Module
274	423	ASN	A
275	425	GLY	A
276	426	PHE	A
277	427	ILE	A
278	428	VAL	A
279	429	PHE	A
280	430	ASN	A
281	433	ASP	A
282	434	TRP	A
283	435	SER	A
284	436	PHE	A
285	437	SER	A
286	438	LEU	A
287	439	THR	A
288	445	PRO	A
289	446	ALA	A
290	449	TYR	A
291	450	CYS	A
292	451	ASP	A
293	452	VAL	A
294	479	ILE	A
295	480	SER	A
296	481	ASN	A
297	486	PRO	A
298	487	PHE	A
299	488	ILE	A
300	489	ALA	A
301	490	ILE	A
302	491	HIS	A

Table S67: Residues membership for the *H. sapiens* α-amylase (PDB code 3BAI, chain A)

Homology index	Residue Index	Residue abbreviation	Module
0	11	THR	A
1	12	SER	A
2	13	ILE	B
3	14	VAL	B
4	15	HIS	B
5	16	LEU	C
6	17	PHE	C
7	18	GLU	C
8	19	TRP	C

Continued on next page

Table S67 – *Continued from previous page*

Homology index	Residue Index	Residue abbreviation	Module
9	20	ARG	C
10	21	TRP	C
11	22	VAL	C
12	23	ASP	C
13	24	ILE	C
14	25	ALA	C
15	26	LEU	C
16	27	GLU	C
17	28	CYS	C
18	29	GLU	C
19	30	ARG	C
20	32	LEU	C
21	33	ALA	C
22	34	PRO	C
23	35	LYS	C
24	36	GLY	C
25	37	PHE	C
26	38	GLY	C
27	39	GLY	C
28	40	VAL	C
29	41	GLN	C
30	42	VAL	C
31	43	SER	C
32	44	PRO	C
33	45	PRO	C
34	46	ASN	C
35	47	GLU	C
36	48	ASN	C
37	49	VAL	C
38	51	ILE	C
39	59	TRP	C
40	60	GLU	C
41	61	ARG	C
42	62	TYR	C
43	63	GLN	C
44	64	PRO	C
45	65	VAL	C
46	66	SER	C
47	67	TYR	C
48	68	LYS	C
49	69	LEU	C
50	70	CYS	C
51	71	THR	C
52	72	ARG	C
53	73	SER	C
54	74	GLY	C

Continued on next page

Table S67 – *Continued from previous page*

Homology index	Residue Index	Residue abbreviation	Module
55	75	ASN	C
56	76	GLU	C
57	77	ASP	C
58	78	GLU	C
59	79	PHE	C
60	80	ARG	C
61	81	ASN	C
62	82	MET	C
63	83	VAL	C
64	84	THR	C
65	85	ARG	C
66	86	CYS	C
67	87	ASN	C
68	88	ASN	C
69	89	VAL	C
70	90	GLY	C
71	91	VAL	C
72	92	ARG	C
73	93	ILE	C
74	94	TYR	C
75	95	VAL	C
76	96	ASP	C
77	97	ALA	C
78	98	VAL	C
79	99	ILE	C
80	100	ASN	C
81	101	HIS	C
82	102	MET	C
83	103	CYS	C
84	104	GLY	C
85	105	ASN	C
86	126	PHE	C
87	130	PRO	C
88	131	TYR	C
89	132	SER	C
90	133	GLY	C
91	134	TRP	C
92	135	ASP	C
93	136	PHE	C
94	137	ASN	B
95	160	CYS	B
96	161	ARG	B
97	162	LEU	B
98	163	THR	B
99	165	LEU	B
100	166	LEU	B

Continued on next page

Table S67 – *Continued from previous page*

Homology index	Residue Index	Residue abbreviation	Module
101	167	ASP	B
102	168	LEU	B
103	169	ALA	B
104	170	LEU	B
105	171	GLU	B
106	172	LYS	B
107	173	ASP	B
108	174	TYR	B
109	175	VAL	B
110	176	ARG	B
111	177	SER	B
112	178	LYS	B
113	179	ILE	B
114	180	ALA	B
115	181	GLU	B
116	182	TYR	B
117	183	MET	B
118	184	ASN	B
119	185	HIS	B
120	186	LEU	B
121	187	ILE	B
122	188	ASP	B
123	189	ILE	C
124	190	GLY	C
125	191	VAL	C
126	192	ALA	C
127	193	GLY	C
128	194	PHE	B
129	195	ARG	D
130	196	LEU	B
131	197	ASP	B
132	198	ALA	B
133	199	SER	B
134	200	LYS	B
135	201	HIS	B
136	202	MET	B
137	203	TRP	B
138	204	PRO	B
139	205	GLY	B
140	206	ASP	B
141	207	ILE	B
142	208	LYS	B
143	209	ALA	B
144	210	ILE	B
145	211	LEU	B
146	212	ASP	B

Continued on next page

Table S67 – *Continued from previous page*

Homology index	Residue Index	Residue abbreviation	Module
147	213	LYS	B
148	214	LEU	B
149	215	HIS	B
150	228	PRO	B
151	229	PHE	B
152	230	ILE	B
153	231	TYR	C
154	232	GLN	B
155	233	GLU	B
156	234	VAL	B
157	235	ILE	B
158	236	ASP	B
159	237	LEU	B
160	244	SER	B
161	245	SER	B
162	246	ASP	B
163	247	TYR	B
164	248	PHE	B
165	249	GLY	B
166	250	ASN	B
167	251	GLY	B
168	252	ARG	B
169	253	VAL	B
170	254	THR	D
171	255	GLU	D
172	256	PHE	D
173	257	LYS	D
174	259	GLY	D
175	260	ALA	D
176	261	LYS	D
177	262	LEU	D
178	263	GLY	D
179	264	THR	D
180	265	VAL	D
181	266	ILE	D
182	272	GLU	D
183	273	LYS	A
184	274	MET	A
185	275	SER	A
186	280	TRP	A
187	281	GLY	D
188	282	GLU	D
189	283	GLY	D
190	285	GLY	D
191	286	PHE	B
192	287	VAL	B

Continued on next page

Table S67 – *Continued from previous page*

Homology index	Residue Index	Residue abbreviation	Module
193	288	PRO	B
194	289	SER	B
195	290	ASP	B
196	291	ARG	B
197	292	ALA	B
198	293	LEU	D
199	294	VAL	D
200	295	PHE	D
201	296	VAL	A
202	297	ASP	D
203	298	ASN	D
204	299	HIS	D
205	300	ASP	D
206	301	ASN	D
207	302	GLN	D
208	314	THR	D
209	315	PHE	D
210	316	TRP	D
211	317	ASP	D
212	319	ARG	A
213	320	LEU	A
214	321	TYR	A
215	322	LYS	A
216	323	MET	A
217	324	ALA	A
218	325	VAL	A
219	326	GLY	A
220	327	PHE	A
221	328	MET	A
222	329	LEU	A
223	330	ALA	A
224	331	HIS	A
225	333	TYR	D
226	334	GLY	D
227	335	PHE	D
228	336	THR	A
229	337	ARG	D
230	338	VAL	A
231	339	MET	D
232	340	SER	C
233	341	SER	D
234	342	TYR	A
235	343	ARG	C
236	381	ASP	C
237	382	TRP	D
238	383	VAL	D

Continued on next page

Table S67 – *Continued from previous page*

Homology index	Residue Index	Residue abbreviation	Module
239	384	CYS	D
240	387	ARG	A
241	388	TRP	A
242	389	ARG	A
243	390	GLN	A
244	391	ILE	A
245	392	ARG	A
246	393	ASN	A
247	394	MET	A
248	395	VAL	A
249	396	ILE	A
250	397	PHE	A
251	398	ARG	A
252	399	ASN	A
253	400	VAL	A
254	401	VAL	A
255	402	ASP	A
256	403	GLY	A
257	404	GLN	A
258	405	PRO	A
259	406	PHE	A
260	408	ASN	A
261	409	TRP	A
262	410	TYR	A
263	411	ASP	A
264	413	GLY	A
265	414	SER	A
266	415	ASN	A
267	416	GLN	A
268	417	VAL	A
269	418	ALA	A
270	419	PHE	A
271	420	GLY	A
272	421	ARG	A
273	422	GLY	A
274	423	ASN	A
275	425	GLY	A
276	426	PHE	A
277	427	ILE	A
278	428	VAL	A
279	429	PHE	A
280	430	ASN	A
281	433	ASP	A
282	434	TRP	A
283	435	SER	A
284	436	PHE	A

Continued on next page

Table S67 – *Continued from previous page*

Homology index	Residue Index	Residue abbreviation	Module
285	437	SER	A
286	438	LEU	A
287	439	THR	A
288	445	PRO	A
289	446	ALA	A
290	449	TYR	A
291	450	CYS	A
292	451	ASP	A
293	452	VAL	A
294	479	ILE	A
295	480	SER	A
296	481	ASN	A
297	486	PRO	A
298	487	PHE	A
299	488	ILE	A
300	489	ALA	A
301	490	ILE	A
302	491	HIS	A

Table S68: Residues membership for the *T. thermosulfurigenes EM1* α-amylase (PDB code 3BMV, chain A)

Homology index	Residue Index	Residue abbreviation	Module
0	15	ASP	A
1	16	VAL	A
2	17	ILE	B
3	18	TYR	B
4	19	GLN	B
5	20	ILE	C
6	21	VAL	C
7	22	THR	C
8	52	GLY	C
9	53	ASP	C
10	54	TRP	C
11	55	GLN	C
12	56	GLY	C
13	57	ILE	C
14	58	ILE	C
15	59	ASN	C
16	60	LYS	C
17	61	ILE	C
18	62	ASN	C
19	63	ASP	C

Continued on next page

Table S68 – *Continued from previous page*

**Homology index**	**Residue Index**	**Residue abbreviation**	**Module**
20	66	LEU	C
21	67	THR	C
22	68	GLY	C
23	69	MET	C
24	70	GLY	C
25	71	VAL	C
26	72	THR	C
27	73	ALA	C
28	74	ILE	C
29	75	TRP	C
30	76	ILE	C
31	77	PRO	C
32	78	GLN	C
33	79	PRO	C
34	80	VAL	C
35	81	GLU	C
36	82	ASN	C
37	83	ILE	C
38	96	THR	C
39	98	TYR	C
40	99	HIS	C
41	100	GLY	C
42	101	TYR	C
43	102	TRP	C
44	103	ALA	C
45	104	ARG	C
46	105	ASP	C
47	107	LYS	C
48	108	ARG	C
49	109	THR	C
50	110	ASN	C
51	111	PRO	C
52	112	TYR	C
53	113	PHE	C
54	114	GLY	C
55	115	SER	C
56	116	PHE	C
57	117	THR	C
58	118	ASP	C
59	119	PHE	C
60	120	GLN	C
61	121	ASN	C
62	122	LEU	C
63	123	ILE	C
64	124	ASN	C
65	125	THR	C

Continued on next page

Table S68 – *Continued from previous page*

Homology index	Residue Index	Residue abbreviation	Module
66	126	ALA	C
67	127	HIS	C
68	128	ALA	C
69	129	HIS	C
70	130	ASN	C
71	131	ILE	C
72	132	LYS	C
73	133	VAL	C
74	134	ILE	C
75	135	ILE	C
76	136	ASP	C
77	137	PHE	C
78	138	ALA	C
79	139	PRO	C
80	140	ASN	C
81	141	HIS	C
82	142	THR	C
83	143	SER	C
84	144	PRO	C
85	145	ALA	C
86	157	ARG	C
87	164	LEU	C
88	165	LEU	C
89	166	GLY	C
90	173	ASN	C
91	174	GLY	C
92	175	TYR	C
93	176	PHE	C
94	177	HIS	B
95	193	ARG	B
96	194	ASN	B
97	195	LEU	B
98	196	PHE	B
99	198	LEU	B
100	199	ALA	B
101	200	ASP	B
102	201	LEU	B
103	202	ASN	B
104	203	GLN	B
105	204	GLN	B
106	205	ASN	B
107	206	SER	B
108	207	THR	B
109	208	ILE	B
110	209	ASP	B
111	210	SER	B

Continued on next page

Table S68 – *Continued from previous page*

**Homology index**	**Residue Index**	**Residue abbreviation**	**Module**
112	211	TYR	B
113	212	LEU	B
114	213	LYS	B
115	214	SER	B
116	215	ALA	B
117	216	ILE	B
118	217	LYS	B
119	218	VAL	B
120	219	TRP	B
121	220	LEU	B
122	221	ASP	B
123	222	MET	C
124	223	GLY	C
125	224	ILE	C
126	225	ASP	C
127	226	GLY	C
128	227	ILE	B
129	228	ARG	D
130	229	LEU	B
131	230	ASP	B
132	231	ALA	B
133	232	VAL	B
134	233	LYS	B
135	234	HIS	B
136	235	MET	B
137	236	PRO	B
138	237	PHE	B
139	238	GLY	B
140	239	TRP	B
141	240	GLN	B
142	241	LYS	B
143	242	ASN	B
144	243	PHE	B
145	244	MET	B
146	245	ASP	B
147	246	SER	B
148	247	ILE	B
149	248	LEU	B
150	253	VAL	B
151	254	PHE	B
152	255	THR	B
153	256	PHE	C
154	257	GLY	B
155	258	GLU	B
156	259	TRP	B
157	260	PHE	B

Continued on next page

Table S68 – *Continued from previous page*

Homology index	Residue Index	Residue abbreviation	Module
158	261	LEU	B
159	262	GLY	B
160	270	ASN	B
161	271	THR	B
162	272	TYR	B
163	273	PHE	B
164	275	ASN	B
165	276	GLU	B
166	278	GLY	B
167	279	MET	B
168	280	SER	B
169	281	LEU	B
170	282	LEU	D
171	283	ASP	D
172	284	PHE	D
173	285	ARG	D
174	287	SER	D
175	288	GLN	D
176	289	LYS	D
177	290	VAL	D
178	291	ARG	D
179	292	GLN	D
180	293	VAL	D
181	294	PHE	D
182	299	ASP	D
183	300	THR	A
184	301	MET	A
185	302	TYR	A
186	307	MET	A
187	308	ILE	D
188	309	GLN	D
189	310	SER	D
190	311	THR	D
191	312	ALA	B
192	313	SER	B
193	317	PHE	B
194	318	ILE	B
195	319	ASN	B
196	320	ASP	B
197	321	MET	B
198	322	VAL	D
199	323	THR	D
200	324	PHE	D
201	325	ILE	A
202	326	ASP	D
203	327	ASN	D

Continued on next page

Table S68 – *Continued from previous page*

Homology index	Residue Index	Residue abbreviation	Module
204	328	HIS	D
205	329	ASP	D
206	330	MET	D
207	331	ASP	D
208	332	ARG	D
209	333	PHE	D
210	334	TYR	D
211	335	ASN	D
212	339	THR	A
213	341	PRO	A
214	342	VAL	A
215	343	GLU	A
216	344	GLN	A
217	345	ALA	A
218	346	LEU	A
219	347	ALA	A
220	348	PHE	A
221	349	THR	A
222	350	LEU	A
223	351	THR	A
224	352	SER	A
225	353	ARG	D
226	354	GLY	D
227	355	VAL	D
228	356	PRO	A
229	357	ALA	D
230	358	ILE	A
231	359	TYR	D
232	360	TYR	C
233	361	GLY	D
234	362	THR	A
235	376	ALA	C
236	377	MET	C
237	378	MET	D
238	379	THR	D
239	381	PHE	D
240	387	ALA	A
241	388	TYR	A
242	389	ASN	A
243	390	VAL	A
244	391	ILE	A
245	392	LYS	A
246	393	LYS	A
247	394	LEU	A
248	395	ALA	A
249	396	PRO	A

Continued on next page

Table S68 – Continued from previous page

Homology index	Residue Index	Residue abbreviation	Module
250	397	LEU	A
251	398	ARG	A
252	399	LYS	A
253	400	SER	A
254	401	ASN	A
255	402	PRO	A
256	406	TYR	A
257	407	GLY	A
258	408	THR	A
259	409	THR	A
260	411	GLN	A
261	412	ARG	A
262	413	TRP	A
263	414	ILE	A
264	415	ASN	A
265	416	ASN	A
266	417	ASP	A
267	418	VAL	A
268	419	TYR	A
269	420	ILE	A
270	421	TYR	A
271	422	GLU	A
272	423	ARG	A
273	428	ASN	A
274	429	VAL	A
275	430	ALA	A
276	431	LEU	A
277	432	VAL	A
278	433	ALA	A
279	434	ILE	A
280	435	ASN	A
281	439	SER	A
282	440	THR	A
283	441	SER	A
284	442	TYR	A
285	443	ASN	A
286	444	ILE	A
287	445	THR	A
288	454	GLY	A
289	455	THR	A
290	457	THR	A
291	458	ASP	A
292	459	VAL	A
293	460	LEU	A
294	483	SER	A
295	484	ALA	A

Continued on next page

Table S68 – *Continued from previous page*

Homology index	Residue Index	Residue abbreviation	Module
296	485	GLY	A
297	486	GLU	A
298	487	VAL	A
299	488	ALA	A
300	489	VAL	A
301	490	TRP	A
302	491	GLN	A

Table S69: Residues membership for the *H. sapiens* α-amylase (PDB code 3BAW, chain A)

Homology index	Residue Index	Residue abbreviation	Module
0	11	THR	A
1	12	SER	A
2	13	ILE	B
3	14	VAL	B
4	15	HIS	B
5	16	LEU	C
6	17	PHE	C
7	18	GLU	C
8	19	TRP	C
9	20	ARG	C
10	21	TRP	C
11	22	VAL	C
12	23	ASP	C
13	24	ILE	C
14	25	ALA	C
15	26	LEU	C
16	27	GLU	C
17	28	CYS	C
18	29	GLU	C
19	30	ARG	C
20	32	LEU	C
21	33	ALA	C
22	34	PRO	C
23	35	LYS	C
24	36	GLY	C
25	37	PHE	C
26	38	GLY	C
27	39	GLY	C
28	40	VAL	C
29	41	GLN	C
30	42	VAL	C

Continued on next page

Table S69 – *Continued from previous page*

Homology index	Residue Index	Residue abbreviation	Module
31	43	SER	C
32	44	PRO	C
33	45	PRO	C
34	46	ASN	C
35	47	GLU	C
36	48	ASN	C
37	49	VAL	C
38	51	ILE	C
39	59	TRP	C
40	60	GLU	C
41	61	ARG	C
42	62	TYR	C
43	63	GLN	C
44	64	PRO	C
45	65	VAL	C
46	66	SER	C
47	67	TYR	C
48	68	LYS	C
49	69	LEU	C
50	70	CYS	C
51	71	THR	C
52	72	ARG	C
53	73	SER	C
54	74	GLY	C
55	75	ASN	C
56	76	GLU	C
57	77	ASP	C
58	78	GLU	C
59	79	PHE	C
60	80	ARG	C
61	81	ASN	C
62	82	MET	C
63	83	VAL	C
64	84	THR	C
65	85	ARG	C
66	86	CYS	C
67	87	ASN	C
68	88	ASN	C
69	89	VAL	C
70	90	GLY	C
71	91	VAL	C
72	92	ARG	C
73	93	ILE	C
74	94	TYR	C
75	95	VAL	C
76	96	ASP	C

Continued on next page

Table S69 – *Continued from previous page*

**Homology index**	**Residue Index**	**Residue abbreviation**	**Module**
77	97	ALA	C
78	98	VAL	C
79	99	ILE	C
80	100	ASN	C
81	101	HIS	C
82	102	MET	C
83	103	CYS	C
84	104	GLY	C
85	105	ASN	C
86	126	PHE	C
87	130	PRO	C
88	131	TYR	C
89	132	SER	C
90	133	GLY	C
91	134	TRP	C
92	135	ASP	C
93	136	PHE	C
94	137	ASN	B
95	160	CYS	B
96	161	ARG	B
97	162	LEU	B
98	163	THR	B
99	165	LEU	B
100	166	LEU	B
101	167	ASP	B
102	168	LEU	B
103	169	ALA	B
104	170	LEU	B
105	171	GLU	B
106	172	LYS	B
107	173	ASP	B
108	174	TYR	B
109	175	VAL	B
110	176	ARG	B
111	177	SER	B
112	178	LYS	B
113	179	ILE	B
114	180	ALA	B
115	181	GLU	B
116	182	TYR	B
117	183	MET	B
118	184	ASN	B
119	185	HIS	B
120	186	LEU	B
121	187	ILE	B
122	188	ASP	B

Continued on next page

Table S69 – *Continued from previous page*

Homology index	Residue Index	Residue abbreviation	Module
123	189	ILE	C
124	190	GLY	C
125	191	VAL	C
126	192	ALA	C
127	193	GLY	C
128	194	PHE	B
129	195	ARG	D
130	196	LEU	B
131	197	ASP	B
132	198	ALA	B
133	199	SER	B
134	200	LYS	B
135	201	HIS	B
136	202	MET	B
137	203	TRP	B
138	204	PRO	B
139	205	GLY	B
140	206	ASP	B
141	207	ILE	B
142	208	LYS	B
143	209	ALA	B
144	210	ILE	B
145	211	LEU	B
146	212	ASP	B
147	213	LYS	B
148	214	LEU	B
149	215	HIS	B
150	228	PRO	B
151	229	PHE	B
152	230	ILE	B
153	231	TYR	C
154	232	GLN	B
155	233	GLU	B
156	234	VAL	B
157	235	ILE	B
158	236	ASP	B
159	237	LEU	B
160	244	SER	B
161	245	SER	B
162	246	ASP	B
163	247	TYR	B
164	248	PHE	B
165	249	GLY	B
166	250	ASN	B
167	251	GLY	B
168	252	ARG	B

Continued on next page

Table S69 – *Continued from previous page*

Homology index	Residue Index	Residue abbreviation	Module
169	253	VAL	B
170	254	THR	D
171	255	GLU	D
172	256	PHE	D
173	257	LYS	D
174	259	GLY	D
175	260	ALA	D
176	261	LYS	D
177	262	LEU	D
178	263	GLY	D
179	264	THR	D
180	265	VAL	D
181	266	ILE	D
182	272	GLU	D
183	273	LYS	A
184	274	MET	A
185	275	SER	A
186	280	TRP	A
187	281	GLY	D
188	282	GLU	D
189	283	GLY	D
190	285	GLY	D
191	286	PHE	B
192	287	VAL	B
193	288	PRO	B
194	289	SER	B
195	290	ASP	B
196	291	ARG	B
197	292	ALA	B
198	293	LEU	D
199	294	VAL	D
200	295	PHE	D
201	296	VAL	A
202	297	ASP	D
203	298	ASN	D
204	299	HIS	D
205	300	ASP	D
206	301	ASN	D
207	302	GLN	D
208	314	THR	D
209	315	PHE	D
210	316	TRP	D
211	317	ASP	D
212	319	ARG	A
213	320	LEU	A
214	321	TYR	A

Continued on next page

Table S69 – *Continued from previous page*

Homology index	Residue Index	Residue abbreviation	Module
215	322	LYS	A
216	323	MET	A
217	324	ALA	A
218	325	VAL	A
219	326	GLY	A
220	327	PHE	A
221	328	MET	A
222	329	LEU	A
223	330	ALA	A
224	331	HIS	A
225	333	TYR	D
226	334	GLY	D
227	335	PHE	D
228	336	THR	A
229	337	ARG	D
230	338	VAL	A
231	339	MET	D
232	340	SER	C
233	341	SER	D
234	342	TYR	A
235	343	ARG	C
236	381	ASP	C
237	382	TRP	D
238	383	VAL	D
239	384	CYS	D
240	387	ARG	A
241	388	TRP	A
242	389	ARG	A
243	390	GLN	A
244	391	ILE	A
245	392	ARG	A
246	393	ASN	A
247	394	MET	A
248	395	VAL	A
249	396	ILE	A
250	397	PHE	A
251	398	ARG	A
252	399	ASN	A
253	400	VAL	A
254	401	VAL	A
255	402	ASP	A
256	403	GLY	A
257	404	GLN	A
258	405	PRO	A
259	406	PHE	A
260	408	ASN	A

Continued on next page

Table S69 – *Continued from previous page*

Homology index	Residue Index	Residue abbreviation	Module
261	409	TRP	A
262	410	TYR	A
263	411	ASP	A
264	413	GLY	A
265	414	SER	A
266	415	ASN	A
267	416	GLN	A
268	417	VAL	A
269	418	ALA	A
270	419	PHE	A
271	420	GLY	A
272	421	ARG	A
273	422	GLY	A
274	423	ASN	A
275	425	GLY	A
276	426	PHE	A
277	427	ILE	A
278	428	VAL	A
279	429	PHE	A
280	430	ASN	A
281	433	ASP	A
282	434	TRP	A
283	435	SER	A
284	436	PHE	A
285	437	SER	A
286	438	LEU	A
287	439	THR	A
288	445	PRO	A
289	446	ALA	A
290	449	TYR	A
291	450	CYS	A
292	451	ASP	A
293	452	VAL	A
294	479	ILE	A
295	480	SER	A
296	481	ASN	A
297	486	PRO	A
298	487	PHE	A
299	488	ILE	A
300	489	ALA	A
301	490	ILE	A
302	491	HIS	A

Table S70: Residues membership for the *B. circulans,s8* α-amylase (PDB code 3CGT, chain A)

Homology index	Residue Index	Residue abbreviation	Module
0	15	ASP	A
1	16	VAL	A
2	17	ILE	B
3	18	TYR	B
4	19	GLN	B
5	20	VAL	C
6	21	PHE	C
7	22	THR	C
8	52	GLY	C
9	53	ASP	C
10	54	TRP	C
11	55	GLN	C
12	56	GLY	C
13	57	LEU	C
14	58	ILE	C
15	59	ASN	C
16	60	LYS	C
17	61	ILE	C
18	62	ASN	C
19	63	ASP	C
20	66	PHE	C
21	67	SER	C
22	68	ASP	C
23	69	LEU	C
24	70	GLY	C
25	71	VAL	C
26	72	THR	C
27	73	ALA	C
28	74	LEU	C
29	75	TRP	C
30	76	ILE	C
31	77	SER	C
32	78	GLN	C
33	79	PRO	C
34	80	VAL	C
35	81	GLU	C
36	82	ASN	C
37	83	ILE	C
38	95	THR	C
39	97	TYR	C
40	98	HIS	C
41	99	GLY	C
42	100	TYR	C
43	101	TRP	C

Continued on next page

Table S70 – *Continued from previous page*

Homology index	Residue Index	Residue abbreviation	Module
44	102	ALA	C
45	103	ARG	C
46	104	ASP	C
47	106	LYS	C
48	107	LYS	C
49	108	THR	C
50	109	ASN	C
51	110	PRO	C
52	111	TYR	C
53	112	PHE	C
54	113	GLY	C
55	114	THR	C
56	115	MET	C
57	116	ALA	C
58	117	ASP	C
59	118	PHE	C
60	119	GLN	C
61	120	ASN	C
62	121	LEU	C
63	122	ILE	C
64	123	THR	C
65	124	THR	C
66	125	ALA	C
67	126	HIS	C
68	127	ALA	C
69	128	LYS	C
70	129	GLY	C
71	130	ILE	C
72	131	LYS	C
73	132	ILE	C
74	133	VAL	C
75	134	ILE	C
76	135	ASP	C
77	136	PHE	C
78	137	ALA	C
79	138	PRO	C
80	139	ASN	C
81	140	HIS	C
82	141	THR	C
83	142	SER	C
84	143	PRO	C
85	144	ALA	C
86	156	ARG	C
87	163	LEU	C
88	164	VAL	C
89	165	GLY	C

Continued on next page

Table S70 – *Continued from previous page*

Homology index	Residue Index	Residue abbreviation	Module
90	172	ASN	C
91	173	GLY	C
92	174	TYR	C
93	175	PHE	C
94	176	HIS	B
95	192	LYS	B
96	193	ASN	B
97	194	LEU	B
98	195	TYR	B
99	197	LEU	B
100	198	ALA	B
101	199	ASP	B
102	200	PHE	B
103	201	ASN	B
104	202	HIS	B
105	203	ASN	B
106	204	ASN	B
107	205	ALA	B
108	206	THR	B
109	207	ILE	B
110	208	ASP	B
111	209	LYS	B
112	210	TYR	B
113	211	PHE	B
114	212	LYS	B
115	213	ASP	B
116	214	ALA	B
117	215	ILE	B
118	216	LYS	B
119	217	LEU	B
120	218	TRP	B
121	219	LEU	B
122	220	ASP	B
123	221	MET	C
124	222	GLY	C
125	223	VAL	C
126	224	ASP	C
127	225	GLY	C
128	226	ILE	B
129	227	ARG	D
130	228	VAL	B
131	229	ASP	B
132	230	ALA	B
133	231	VAL	B
134	232	LYS	B
135	233	HIS	B

Continued on next page

Table S70 – *Continued from previous page*

Homology index	Residue Index	Residue abbreviation	Module
136	234	MET	B
137	235	PRO	B
138	236	LEU	B
139	237	GLY	B
140	238	TRP	B
141	239	GLN	B
142	240	LYS	B
143	241	SER	B
144	242	TRP	B
145	243	MET	B
146	244	SER	B
147	245	SER	B
148	246	ILE	B
149	247	TYR	B
150	252	VAL	B
151	253	PHE	B
152	254	THR	B
153	255	PHE	C
154	256	GLY	B
155	257	ALA	B
156	258	TRP	B
157	259	PHE	B
158	260	LEU	B
159	261	GLY	B
160	269	ASN	B
161	270	THR	B
162	271	ASP	B
163	272	PHE	B
164	274	ASN	B
165	275	LYS	B
166	277	GLY	B
167	278	MET	B
168	279	SER	B
169	280	LEU	B
170	281	LEU	D
171	282	ASP	D
172	283	PHE	D
173	284	ARG	D
174	286	ASN	D
175	287	SER	D
176	288	ALA	D
177	289	VAL	D
178	290	ARG	D
179	291	ASN	D
180	292	VAL	D
181	293	PHE	D

Continued on next page

Table S70 – *Continued from previous page*

Homology index	Residue Index	Residue abbreviation	Module
182	298	SER	D
183	299	ASN	A
184	300	MET	A
185	301	TYR	A
186	306	MET	A
187	307	ILE	D
188	308	ASN	D
189	309	SER	D
190	310	THR	D
191	311	ALA	B
192	312	THR	B
193	316	GLN	B
194	317	VAL	B
195	318	ASN	B
196	319	ASP	B
197	320	GLN	B
198	321	VAL	D
199	322	THR	D
200	323	PHE	D
201	324	ILE	A
202	325	ASP	D
203	326	ASN	D
204	327	HIS	D
205	328	ASP	D
206	329	MET	D
207	330	ASP	D
208	331	ARG	D
209	332	PHE	D
210	333	LYS	D
211	334	THR	D
212	339	ASN	A
213	341	ARG	A
214	342	LEU	A
215	343	GLU	A
216	344	GLN	A
217	345	ALA	A
218	346	LEU	A
219	347	ALA	A
220	348	PHE	A
221	349	THR	A
222	350	LEU	A
223	351	THR	A
224	352	SER	A
225	353	ARG	D
226	354	GLY	D
227	355	VAL	D

Continued on next page

Table S70 – *Continued from previous page*

Homology index	Residue Index	Residue abbreviation	Module
228	356	PRO	A
229	357	ALA	D
230	358	ILE	A
231	359	TYR	D
232	360	TYR	C
233	361	GLY	D
234	362	THR	A
235	376	ALA	C
236	377	LYS	C
237	378	MET	D
238	379	PRO	D
239	381	PHE	D
240	387	ALA	A
241	388	PHE	A
242	389	ASN	A
243	390	VAL	A
244	391	ILE	A
245	392	SER	A
246	393	LYS	A
247	394	LEU	A
248	395	ALA	A
249	396	PRO	A
250	397	LEU	A
251	398	ARG	A
252	399	LYS	A
253	400	SER	A
254	401	ASN	A
255	402	PRO	A
256	406	TYR	A
257	407	GLY	A
258	408	SER	A
259	409	THR	A
260	411	GLN	A
261	412	ARG	A
262	413	TRP	A
263	414	ILE	A
264	415	ASN	A
265	416	ASN	A
266	417	ASP	A
267	418	VAL	A
268	419	TYR	A
269	420	VAL	A
270	421	TYR	A
271	422	GLU	A
272	423	ARG	A
273	428	SER	A

Continued on next page

Table S70 – *Continued from previous page*

Homology index	Residue Index	Residue abbreviation	Module
274	429	VAL	A
275	430	ALA	A
276	431	VAL	A
277	432	VAL	A
278	433	ALA	A
279	434	VAL	A
280	435	ASN	A
281	439	SER	A
282	440	THR	A
283	441	SER	A
284	442	ALA	A
285	443	SER	A
286	444	ILE	A
287	445	THR	A
288	454	GLY	A
289	455	SER	A
290	457	THR	A
291	458	ASP	A
292	459	VAL	A
293	460	LEU	A
294	482	ALA	A
295	483	ALA	A
296	484	GLY	A
297	485	ALA	A
298	486	THR	A
299	487	ALA	A
300	488	VAL	A
301	489	TRP	A
302	490	GLN	A

Table S71: Residues membership for the *T. thermosulfurigenes EM1* α-amylase (PDB code 3BMW, chain A)

Homology index	Residue Index	Residue abbreviation	Module
0	15	ASP	A
1	16	VAL	A
2	17	ILE	B
3	18	TYR	B
4	19	GLN	B
5	20	ILE	C
6	21	VAL	C
7	22	THR	C
8	52	GLY	C

Continued on next page

Table S71 – *Continued from previous page*

**Homology index**	**Residue Index**	**Residue abbreviation**	**Module**
9	53	ASP	C
10	54	TRP	C
11	55	GLN	C
12	56	GLY	C
13	57	ILE	C
14	58	ILE	C
15	59	ASN	C
16	60	LYS	C
17	61	ILE	C
18	62	ASN	C
19	63	ASP	C
20	66	LEU	C
21	67	THR	C
22	68	GLY	C
23	69	MET	C
24	70	GLY	C
25	71	VAL	C
26	72	THR	C
27	73	ALA	C
28	74	ILE	C
29	75	TRP	C
30	76	ILE	C
31	77	PRO	C
32	78	GLN	C
33	79	PRO	C
34	80	VAL	C
35	81	GLU	C
36	82	ASN	C
37	83	ILE	C
38	96	THR	C
39	98	TYR	C
40	99	HIS	C
41	100	GLY	C
42	101	TYR	C
43	102	TRP	C
44	103	ALA	C
45	104	ARG	C
46	105	ASP	C
47	107	LYS	C
48	108	ARG	C
49	109	THR	C
50	110	ASN	C
51	111	PRO	C
52	112	TYR	C
53	113	PHE	C
54	114	GLY	C

Continued on next page

Table S71 – *Continued from previous page*

Homology index	Residue Index	Residue abbreviation	Module
55	115	SER	C
56	116	PHE	C
57	117	THR	C
58	118	ASP	C
59	119	PHE	C
60	120	GLN	C
61	121	ASN	C
62	122	LEU	C
63	123	ILE	C
64	124	ASN	C
65	125	THR	C
66	126	ALA	C
67	127	HIS	C
68	128	ALA	C
69	129	HIS	C
70	130	ASN	C
71	131	ILE	C
72	132	LYS	C
73	133	VAL	C
74	134	ILE	C
75	135	ILE	C
76	136	ASP	C
77	137	PHE	C
78	138	ALA	C
79	139	PRO	C
80	140	ASN	C
81	141	HIS	C
82	142	THR	C
83	143	SER	C
84	144	PRO	C
85	145	ALA	C
86	157	ARG	C
87	164	LEU	C
88	165	LEU	C
89	166	GLY	C
90	173	ASN	C
91	174	GLY	C
92	175	TYR	C
93	176	PHE	C
94	177	HIS	B
95	193	ARG	B
96	194	ASN	B
97	195	LEU	B
98	196	PHE	B
99	198	LEU	B
100	199	ALA	B

Continued on next page

Table S71 – *Continued from previous page*

Homology index	Residue Index	Residue abbreviation	Module
101	200	ASP	B
102	201	LEU	B
103	202	ASN	B
104	203	GLN	B
105	204	GLN	B
106	205	ASN	B
107	206	SER	B
108	207	THR	B
109	208	ILE	B
110	209	ASP	B
111	210	SER	B
112	211	TYR	B
113	212	LEU	B
114	213	LYS	B
115	214	SER	B
116	215	ALA	B
117	216	ILE	B
118	217	LYS	B
119	218	VAL	B
120	219	TRP	B
121	220	LEU	B
122	221	ASP	B
123	222	MET	C
124	223	GLY	C
125	224	ILE	C
126	225	ASP	C
127	226	GLY	C
128	227	ILE	B
129	228	ARG	D
130	229	LEU	B
131	230	ASP	B
132	231	ALA	B
133	232	VAL	B
134	233	LYS	B
135	234	HIS	B
136	235	MET	B
137	236	PRO	B
138	237	PHE	B
139	238	GLY	B
140	239	TRP	B
141	240	GLN	B
142	241	LYS	B
143	242	ASN	B
144	243	PHE	B
145	244	MET	B
146	245	ASP	B

Continued on next page

Table S71 – *Continued from previous page*

Homology index	Residue Index	Residue abbreviation	Module
147	246	SER	B
148	247	ILE	B
149	248	LEU	B
150	253	VAL	B
151	254	PHE	B
152	255	THR	B
153	256	PHE	C
154	257	GLY	B
155	258	GLU	B
156	259	TRP	B
157	260	PHE	B
158	261	LEU	B
159	262	GLY	B
160	270	ASN	B
161	271	THR	B
162	272	TYR	B
163	273	PHE	B
164	275	ASN	B
165	276	GLU	B
166	278	GLY	B
167	279	MET	B
168	280	SER	B
169	281	LEU	B
170	282	LEU	D
171	283	ASP	D
172	284	PHE	D
173	285	ARG	D
174	287	SER	D
175	288	GLN	D
176	289	LYS	D
177	290	VAL	D
178	291	ARG	D
179	292	GLN	D
180	293	VAL	D
181	294	PHE	D
182	299	ASP	D
183	300	THR	A
184	301	MET	A
185	302	TYR	A
186	307	MET	A
187	308	ILE	D
188	309	GLN	D
189	310	SER	D
190	311	THR	D
191	312	ALA	B
192	313	SER	B

Continued on next page

Table S71 – *Continued from previous page*

Homology index	Residue Index	Residue abbreviation	Module
193	317	PHE	B
194	318	ILE	B
195	319	ASN	B
196	320	ASP	B
197	321	MET	B
198	322	VAL	D
199	323	THR	D
200	324	PHE	D
201	325	ILE	A
202	326	ASP	D
203	327	ASN	D
204	328	HIS	D
205	329	ASP	D
206	330	MET	D
207	331	ASP	D
208	332	ARG	D
209	333	PHE	D
210	334	TYR	D
211	335	ASN	D
212	339	THR	A
213	341	PRO	A
214	342	VAL	A
215	343	GLU	A
216	344	GLN	A
217	345	ALA	A
218	346	LEU	A
219	347	ALA	A
220	348	PHE	A
221	349	THR	A
222	350	LEU	A
223	351	THR	A
224	352	SER	A
225	353	ARG	D
226	354	GLY	D
227	355	VAL	D
228	356	PRO	A
229	357	ALA	D
230	358	ILE	A
231	359	TYR	D
232	360	TYR	C
233	361	GLY	D
234	362	THR	A
235	376	ALA	C
236	377	MET	C
237	378	MET	D
238	379	THR	D

Continued on next page

Table S71 – *Continued from previous page*

Homology index	Residue Index	Residue abbreviation	Module
239	381	PHE	D
240	387	ALA	A
241	388	TYR	A
242	389	ASN	A
243	390	VAL	A
244	391	ILE	A
245	392	LYS	A
246	393	LYS	A
247	394	LEU	A
248	395	ALA	A
249	396	PRO	A
250	397	LEU	A
251	398	ARG	A
252	399	LYS	A
253	400	SER	A
254	401	ASN	A
255	402	PRO	A
256	406	TYR	A
257	407	GLY	A
258	408	THR	A
259	409	THR	A
260	411	GLN	A
261	412	ARG	A
262	413	TRP	A
263	414	ILE	A
264	415	ASN	A
265	416	ASN	A
266	417	ASP	A
267	418	VAL	A
268	419	TYR	A
269	420	ILE	A
270	421	TYR	A
271	422	GLU	A
272	423	ARG	A
273	428	ASN	A
274	429	VAL	A
275	430	ALA	A
276	431	LEU	A
277	432	VAL	A
278	433	ALA	A
279	434	ILE	A
280	435	ASN	A
281	439	SER	A
282	440	THR	A
283	441	SER	A
284	442	TYR	A

Continued on next page

Table S71 – *Continued from previous page*

Homology index	Residue Index	Residue abbreviation	Module
285	443	ASN	A
286	444	ILE	A
287	445	THR	A
288	454	GLY	A
289	455	THR	A
290	457	THR	A
291	458	ASP	A
292	459	VAL	A
293	460	LEU	A
294	483	SER	A
295	484	ALA	A
296	485	GLY	A
297	486	GLU	A
298	487	VAL	A
299	488	ALA	A
300	489	VAL	A
301	490	TRP	A
302	491	GLN	A

Table S72: Residues membership for the *S. scrofa* α-amylase (PDB code 3L2M, chain A)

Homology index	Residue Index	Residue abbreviation	Module
0	11	THR	A
1	12	SER	A
2	13	ILE	B
3	14	VAL	B
4	15	HIS	B
5	16	LEU	C
6	17	PHE	C
7	18	GLU	C
8	19	TRP	C
9	20	ARG	C
10	21	TRP	C
11	22	VAL	C
12	23	ASP	C
13	24	ILE	C
14	25	ALA	C
15	26	LEU	C
16	27	GLU	C
17	28	CYS	C
18	29	GLU	C
19	30	ARG	C

Continued on next page

Table S72 – *Continued from previous page*

Homology index	Residue Index	Residue abbreviation	Module
20	32	LEU	C
21	33	GLY	C
22	34	PRO	C
23	35	LYS	C
24	36	GLY	C
25	37	PHE	C
26	38	GLY	C
27	39	GLY	C
28	40	VAL	C
29	41	GLN	C
30	42	VAL	C
31	43	SER	C
32	44	PRO	C
33	45	PRO	C
34	46	ASN	C
35	47	GLU	C
36	48	ASN	C
37	49	ILE	C
38	51	VAL	C
39	59	TRP	C
40	60	GLU	C
41	61	ARG	C
42	62	TYR	C
43	63	GLN	C
44	64	PRO	C
45	65	VAL	C
46	66	SER	C
47	67	TYR	C
48	68	LYS	C
49	69	LEU	C
50	70	CYS	C
51	71	THR	C
52	72	ARG	C
53	73	SER	C
54	74	GLY	C
55	75	ASN	C
56	76	GLU	C
57	77	ASN	C
58	78	GLU	C
59	79	PHE	C
60	80	ARG	C
61	81	ASP	C
62	82	MET	C
63	83	VAL	C
64	84	THR	C
65	85	ARG	C

Continued on next page

Table S72 – *Continued from previous page*

Homology index	Residue Index	Residue abbreviation	Module
66	86	CYS	C
67	87	ASN	C
68	88	ASN	C
69	89	VAL	C
70	90	GLY	C
71	91	VAL	C
72	92	ARG	C
73	93	ILE	C
74	94	TYR	C
75	95	VAL	C
76	96	ASP	C
77	97	ALA	C
78	98	VAL	C
79	99	ILE	C
80	100	ASN	C
81	101	HIS	C
82	102	MET	C
83	103	CYS	C
84	104	GLY	C
85	105	SER	C
86	126	PHE	C
87	130	PRO	C
88	131	TYR	C
89	132	SER	C
90	133	ALA	C
91	134	TRP	C
92	135	ASP	C
93	136	PHE	C
94	137	ASN	B
95	160	CYS	B
96	161	GLN	B
97	162	LEU	B
98	163	VAL	B
99	165	LEU	B
100	166	LEU	B
101	167	ASP	B
102	168	LEU	B
103	169	ALA	B
104	170	LEU	B
105	171	GLU	B
106	172	LYS	B
107	173	ASP	B
108	174	TYR	B
109	175	VAL	B
110	176	ARG	B
111	177	SER	B

Continued on next page

Table S72 – *Continued from previous page*

Homology index	Residue Index	Residue abbreviation	Module
112	178	MET	B
113	179	ILE	B
114	180	ALA	B
115	181	ASP	B
116	182	TYR	B
117	183	LEU	B
118	184	ASN	B
119	185	LYS	B
120	186	LEU	B
121	187	ILE	B
122	188	ASP	B
123	189	ILE	C
124	190	GLY	C
125	191	VAL	C
126	192	ALA	C
127	193	GLY	C
128	194	PHE	B
129	195	ARG	D
130	196	ILE	B
131	197	ASP	B
132	198	ALA	B
133	199	SER	B
134	200	LYS	B
135	201	HIS	B
136	202	MET	B
137	203	TRP	B
138	204	PRO	B
139	205	GLY	B
140	206	ASP	B
141	207	ILE	B
142	208	LYS	B
143	209	ALA	B
144	210	VAL	B
145	211	LEU	B
146	212	ASP	B
147	213	LYS	B
148	214	LEU	B
149	215	HIS	B
150	228	PRO	B
151	229	PHE	B
152	230	ILE	B
153	231	PHE	C
154	232	GLN	B
155	233	GLU	B
156	234	VAL	B
157	235	ILE	B

Continued on next page

Table S72 – *Continued from previous page*

Homology index	Residue Index	Residue abbreviation	Module
158	236	ASP	B
159	237	LEU	B
160	244	SER	B
161	245	SER	B
162	246	GLU	B
163	247	TYR	B
164	248	PHE	B
165	249	GLY	B
166	250	ASN	B
167	251	GLY	B
168	252	ARG	B
169	253	VAL	B
170	254	THR	D
171	255	GLU	D
172	256	PHE	D
173	257	LYS	D
174	259	GLY	D
175	260	ALA	D
176	261	LYS	D
177	262	LEU	D
178	263	GLY	D
179	264	THR	D
180	265	VAL	D
181	266	VAL	D
182	272	GLU	D
183	273	LYS	A
184	274	MET	A
185	275	SER	A
186	280	TRP	A
187	281	GLY	D
188	282	GLU	D
189	283	GLY	D
190	285	GLY	D
191	286	PHE	B
192	287	MET	B
193	288	PRO	B
194	289	SER	B
195	290	ASP	B
196	291	ARG	B
197	292	ALA	B
198	293	LEU	D
199	294	VAL	D
200	295	PHE	D
201	296	VAL	A
202	297	ASP	D
203	298	ASN	D

Continued on next page

Table S72 – *Continued from previous page*

Homology index	Residue Index	Residue abbreviation	Module
204	299	HIS	D
205	300	ASP	D
206	301	ASN	D
207	302	GLN	D
208	314	THR	D
209	315	PHE	D
210	316	TRP	D
211	317	ASP	D
212	319	ARG	A
213	320	LEU	A
214	321	TYR	A
215	322	LYS	A
216	323	VAL	A
217	324	ALA	A
218	325	VAL	A
219	326	GLY	A
220	327	PHE	A
221	328	MET	A
222	329	LEU	A
223	330	ALA	A
224	331	HIS	A
225	333	TYR	D
226	334	GLY	D
227	335	PHE	D
228	336	THR	A
229	337	ARG	D
230	338	VAL	A
231	339	MET	D
232	340	SER	C
233	341	SER	D
234	342	TYR	A
235	343	ARG	C
236	381	ASP	C
237	382	TRP	D
238	383	VAL	D
239	384	CYS	D
240	387	ARG	A
241	388	TRP	A
242	389	ARG	A
243	390	GLN	A
244	391	ILE	A
245	392	ARG	A
246	393	ASN	A
247	394	MET	A
248	395	VAL	A
249	396	TRP	A

Continued on next page

Table S72 – *Continued from previous page*

Homology index	Residue Index	Residue abbreviation	Module
250	397	PHE	A
251	398	ARG	A
252	399	ASN	A
253	400	VAL	A
254	401	VAL	A
255	402	ASP	A
256	403	GLY	A
257	404	GLN	A
258	405	PRO	A
259	406	PHE	A
260	408	ASN	A
261	409	TRP	A
262	410	TRP	A
263	411	ASP	A
264	413	GLY	A
265	414	SER	A
266	415	ASN	A
267	416	GLN	A
268	417	VAL	A
269	418	ALA	A
270	419	PHE	A
271	420	GLY	A
272	421	ARG	A
273	422	GLY	A
274	423	ASN	A
275	425	GLY	A
276	426	PHE	A
277	427	ILE	A
278	428	VAL	A
279	429	PHE	A
280	430	ASN	A
281	433	ASP	A
282	434	TRP	A
283	435	GLN	A
284	436	LEU	A
285	437	SER	A
286	438	SER	A
287	439	THR	A
288	445	PRO	A
289	446	GLY	A
290	449	TYR	A
291	450	CYS	A
292	451	ASP	A
293	452	VAL	A
294	479	ILE	A
295	480	SER	A

Continued on next page

Table S72 – *Continued from previous page*

Homology index	Residue Index	Residue abbreviation	Module
296	481	ASN	A
297	486	PRO	A
298	487	PHE	A
299	488	ILE	A
300	489	ALA	A
301	490	ILE	A
302	491	HIS	A

Table S73: Residues membership for the *S. scrofa* α-amylase (PDB code 3L2L, chain A)

Homology index	Residue Index	Residue abbreviation	Module
0	11	THR	A
1	12	SER	A
2	13	ILE	B
3	14	VAL	B
4	15	HIS	B
5	16	LEU	C
6	17	PHE	C
7	18	GLU	C
8	19	TRP	C
9	20	ARG	C
10	21	TRP	C
11	22	VAL	C
12	23	ASP	C
13	24	ILE	C
14	25	ALA	C
15	26	LEU	C
16	27	GLU	C
17	28	CYS	C
18	29	GLU	C
19	30	ARG	C
20	32	LEU	C
21	33	GLY	C
22	34	PRO	C
23	35	LYS	C
24	36	GLY	C
25	37	PHE	C
26	38	GLY	C
27	39	GLY	C
28	40	VAL	C
29	41	GLN	C
30	42	VAL	C

Continued on next page

Table S73 – *Continued from previous page*

**Homology index**	**Residue Index**	**Residue abbreviation**	**Module**
31	43	SER	C
32	44	PRO	C
33	45	PRO	C
34	46	ASN	C
35	47	GLU	C
36	48	ASN	C
37	49	ILE	C
38	51	VAL	C
39	59	TRP	C
40	60	GLU	C
41	61	ARG	C
42	62	TYR	C
43	63	GLN	C
44	64	PRO	C
45	65	VAL	C
46	66	SER	C
47	67	TYR	C
48	68	LYS	C
49	69	LEU	C
50	70	CYS	C
51	71	THR	C
52	72	ARG	C
53	73	SER	C
54	74	GLY	C
55	75	ASN	C
56	76	GLU	C
57	77	ASN	C
58	78	GLU	C
59	79	PHE	C
60	80	ARG	C
61	81	ASP	C
62	82	MET	C
63	83	VAL	C
64	84	THR	C
65	85	ARG	C
66	86	CYS	C
67	87	ASN	C
68	88	ASN	C
69	89	VAL	C
70	90	GLY	C
71	91	VAL	C
72	92	ARG	C
73	93	ILE	C
74	94	TYR	C
75	95	VAL	C
76	96	ASP	C

Continued on next page

Table S73 – *Continued from previous page*

Homology index	Residue Index	Residue abbreviation	Module
77	97	ALA	C
78	98	VAL	C
79	99	ILE	C
80	100	ASN	C
81	101	HIS	C
82	102	MET	C
83	103	CYS	C
84	104	GLY	C
85	105	SER	C
86	126	PHE	C
87	130	PRO	C
88	131	TYR	C
89	132	SER	C
90	133	ALA	C
91	134	TRP	C
92	135	ASP	C
93	136	PHE	C
94	137	ASN	B
95	160	CYS	B
96	161	GLN	B
97	162	LEU	B
98	163	VAL	B
99	165	LEU	B
100	166	LEU	B
101	167	ASP	B
102	168	LEU	B
103	169	ALA	B
104	170	LEU	B
105	171	GLU	B
106	172	LYS	B
107	173	ASP	B
108	174	TYR	B
109	175	VAL	B
110	176	ARG	B
111	177	SER	B
112	178	MET	B
113	179	ILE	B
114	180	ALA	B
115	181	ASP	B
116	182	TYR	B
117	183	LEU	B
118	184	ASN	B
119	185	LYS	B
120	186	LEU	B
121	187	ILE	B
122	188	ASP	B

Continued on next page

Table S73 – *Continued from previous page*

Homology index	Residue Index	Residue abbreviation	Module
123	189	ILE	C
124	190	GLY	C
125	191	VAL	C
126	192	ALA	C
127	193	GLY	C
128	194	PHE	B
129	195	ARG	D
130	196	ILE	B
131	197	ASP	B
132	198	ALA	B
133	199	SER	B
134	200	LYS	B
135	201	HIS	B
136	202	MET	B
137	203	TRP	B
138	204	PRO	B
139	205	GLY	B
140	206	ASP	B
141	207	ILE	B
142	208	LYS	B
143	209	ALA	B
144	210	VAL	B
145	211	LEU	B
146	212	ASP	B
147	213	LYS	B
148	214	LEU	B
149	215	HIS	B
150	228	PRO	B
151	229	PHE	B
152	230	ILE	B
153	231	PHE	C
154	232	GLN	B
155	233	GLU	B
156	234	VAL	B
157	235	ILE	B
158	236	ASP	B
159	237	LEU	B
160	244	SER	B
161	245	SER	B
162	246	GLU	B
163	247	TYR	B
164	248	PHE	B
165	249	GLY	B
166	250	ASN	B
167	251	GLY	B
168	252	ARG	B

Continued on next page

Table S73 – *Continued from previous page*

Homology index	Residue Index	Residue abbreviation	Module
169	253	VAL	B
170	254	THR	D
171	255	GLU	D
172	256	PHE	D
173	257	LYS	D
174	259	GLY	D
175	260	ALA	D
176	261	LYS	D
177	262	LEU	D
178	263	GLY	D
179	264	THR	D
180	265	VAL	D
181	266	VAL	D
182	272	GLU	D
183	273	LYS	A
184	274	MET	A
185	275	SER	A
186	280	TRP	A
187	281	GLY	D
188	282	GLU	D
189	283	GLY	D
190	285	GLY	D
191	286	PHE	B
192	287	MET	B
193	288	PRO	B
194	289	SER	B
195	290	ASP	B
196	291	ARG	B
197	292	ALA	B
198	293	LEU	D
199	294	VAL	D
200	295	PHE	D
201	296	VAL	A
202	297	ASP	D
203	298	ASN	D
204	299	HIS	D
205	300	ASP	D
206	301	ASN	D
207	302	GLN	D
208	314	THR	D
209	315	PHE	D
210	316	TRP	D
211	317	ASP	D
212	319	ARG	A
213	320	LEU	A
214	321	TYR	A

Continued on next page

Table S73 – *Continued from previous page*

Homology index	Residue Index	Residue abbreviation	Module
215	322	LYS	A
216	323	VAL	A
217	324	ALA	A
218	325	VAL	A
219	326	GLY	A
220	327	PHE	A
221	328	MET	A
222	329	LEU	A
223	330	ALA	A
224	331	HIS	A
225	333	TYR	D
226	334	GLY	D
227	335	PHE	D
228	336	THR	A
229	337	ARG	D
230	338	VAL	A
231	339	MET	D
232	340	SER	C
233	341	SER	D
234	342	TYR	A
235	343	ARG	C
236	381	ASP	C
237	382	TRP	D
238	383	VAL	D
239	384	CYS	D
240	387	ARG	A
241	388	TRP	A
242	389	ARG	A
243	390	GLN	A
244	391	ILE	A
245	392	ARG	A
246	393	ASN	A
247	394	MET	A
248	395	VAL	A
249	396	TRP	A
250	397	PHE	A
251	398	ARG	A
252	399	ASN	A
253	400	VAL	A
254	401	VAL	A
255	402	ASP	A
256	403	GLY	A
257	404	GLN	A
258	405	PRO	A
259	406	PHE	A
260	408	ASN	A

Continued on next page

Table S73 – *Continued from previous page*

Homology index	Residue Index	Residue abbreviation	Module
261	409	TRP	A
262	410	TRP	A
263	411	ASP	A
264	413	GLY	A
265	414	SER	A
266	415	ASN	A
267	416	GLN	A
268	417	VAL	A
269	418	ALA	A
270	419	PHE	A
271	420	GLY	A
272	421	ARG	A
273	422	GLY	A
274	423	ASN	A
275	425	GLY	A
276	426	PHE	A
277	427	ILE	A
278	428	VAL	A
279	429	PHE	A
280	430	ASN	A
281	433	ASP	A
282	434	TRP	A
283	435	GLN	A
284	436	LEU	A
285	437	SER	A
286	438	SER	A
287	439	THR	A
288	445	PRO	A
289	446	GLY	A
290	449	TYR	A
291	450	CYS	A
292	451	ASP	A
293	452	VAL	A
294	479	ILE	A
295	480	SER	A
296	481	ASN	A
297	486	PRO	A
298	487	PHE	A
299	488	ILE	A
300	489	ALA	A
301	490	ILE	A
302	491	HIS	A

Table S74: Residues membership for the *B. circulans,s8* α-amylase (PDB code 4CGT, chain A)

**Homology index**	**Residue Index**	**Residue abbreviation**	**Module**
0	15	ASP	A
1	16	VAL	A
2	17	ILE	B
3	18	TYR	B
4	19	GLN	B
5	20	VAL	C
6	21	PHE	C
7	22	THR	C
8	52	GLY	C
9	53	ASP	C
10	54	TRP	C
11	55	GLN	C
12	56	GLY	C
13	57	LEU	C
14	58	ILE	C
15	59	ASN	C
16	60	LYS	C
17	61	ILE	C
18	62	ASN	C
19	63	ASP	C
20	66	PHE	C
21	67	SER	C
22	68	ASP	C
23	69	LEU	C
24	70	GLY	C
25	71	VAL	C
26	72	THR	C
27	73	ALA	C
28	74	LEU	C
29	75	TRP	C
30	76	ILE	C
31	77	SER	C
32	78	GLN	C
33	79	PRO	C
34	80	VAL	C
35	81	GLU	C
36	82	ASN	C
37	83	ILE	C
38	95	THR	C
39	97	TYR	C
40	98	HIS	C
41	99	GLY	C
42	100	TYR	C
43	101	TRP	C

Continued on next page

Table S74 – *Continued from previous page*

Homology index	Residue Index	Residue abbreviation	Module
44	102	ALA	C
45	103	ARG	C
46	104	ASP	C
47	106	LYS	C
48	107	LYS	C
49	108	THR	C
50	109	ASN	C
51	110	PRO	C
52	111	TYR	C
53	112	PHE	C
54	113	GLY	C
55	114	THR	C
56	115	MET	C
57	116	ALA	C
58	117	ASP	C
59	118	PHE	C
60	119	GLN	C
61	120	ASN	C
62	121	LEU	C
63	122	ILE	C
64	123	THR	C
65	124	THR	C
66	125	ALA	C
67	126	HIS	C
68	127	ALA	C
69	128	LYS	C
70	129	GLY	C
71	130	ILE	C
72	131	LYS	C
73	132	ILE	C
74	133	VAL	C
75	134	ILE	C
76	135	ASP	C
77	136	PHE	C
78	137	ALA	C
79	138	PRO	C
80	139	ASN	C
81	140	HIS	C
82	141	THR	C
83	142	SER	C
84	143	PRO	C
85	144	ALA	C
86	156	ARG	C
87	163	LEU	C
88	164	VAL	C
89	165	GLY	C

Continued on next page

Table S74 – *Continued from previous page*

Homology index	Residue Index	Residue abbreviation	Module
90	172	ASN	C
91	173	GLY	C
92	174	TYR	C
93	175	PHE	C
94	176	HIS	B
95	192	LYS	B
96	193	ASN	B
97	194	LEU	B
98	195	TYR	B
99	197	LEU	B
100	198	ALA	B
101	199	ASP	B
102	200	PHE	B
103	201	ASN	B
104	202	HIS	B
105	203	ASN	B
106	204	ASN	B
107	205	ALA	B
108	206	THR	B
109	207	ILE	B
110	208	ASP	B
111	209	LYS	B
112	210	TYR	B
113	211	PHE	B
114	212	LYS	B
115	213	ASP	B
116	214	ALA	B
117	215	ILE	B
118	216	LYS	B
119	217	LEU	B
120	218	TRP	B
121	219	LEU	B
122	220	ASP	B
123	221	MET	C
124	222	GLY	C
125	223	VAL	C
126	224	ASP	C
127	225	GLY	C
128	226	ILE	B
129	227	ARG	D
130	228	VAL	B
131	229	ASP	B
132	230	ALA	B
133	231	VAL	B
134	232	LYS	B
135	233	HIS	B

Continued on next page

Table S74 – *Continued from previous page*

Homology index	Residue Index	Residue abbreviation	Module
136	234	MET	B
137	235	PRO	B
138	236	LEU	B
139	237	GLY	B
140	238	TRP	B
141	239	GLN	B
142	240	LYS	B
143	241	SER	B
144	242	TRP	B
145	243	MET	B
146	244	SER	B
147	245	SER	B
148	246	ILE	B
149	247	TYR	B
150	252	VAL	B
151	253	PHE	B
152	254	THR	B
153	255	PHE	C
154	256	GLY	B
155	257	GLU	B
156	258	TRP	B
157	259	PHE	B
158	260	LEU	B
159	261	GLY	B
160	269	ASN	B
161	270	THR	B
162	271	ASP	B
163	272	PHE	B
164	274	ASN	B
165	275	LYS	B
166	277	GLY	B
167	278	MET	B
168	279	SER	B
169	280	LEU	B
170	281	LEU	D
171	282	ASP	D
172	283	PHE	D
173	284	ARG	D
174	286	ASN	D
175	287	SER	D
176	288	ALA	D
177	289	VAL	D
178	290	ARG	D
179	291	ASN	D
180	292	VAL	D
181	293	PHE	D

Continued on next page

Table S74 – *Continued from previous page*

Homology index	Residue Index	Residue abbreviation	Module
182	298	SER	D
183	299	ASN	A
184	300	MET	A
185	301	TYR	A
186	306	MET	A
187	307	ILE	D
188	308	ASN	D
189	309	SER	D
190	310	THR	D
191	311	ALA	B
192	312	THR	B
193	316	GLN	B
194	317	VAL	B
195	318	ASN	B
196	319	ASP	B
197	320	GLN	B
198	321	VAL	D
199	322	THR	D
200	323	PHE	D
201	324	ILE	A
202	325	ASP	D
203	326	ASN	D
204	327	HIS	D
205	328	ASP	D
206	329	MET	D
207	330	ASP	D
208	331	ARG	D
209	332	PHE	D
210	333	LYS	D
211	334	THR	D
212	339	ASN	A
213	341	ARG	A
214	342	LEU	A
215	343	GLU	A
216	344	GLN	A
217	345	ALA	A
218	346	LEU	A
219	347	ALA	A
220	348	PHE	A
221	349	THR	A
222	350	LEU	A
223	351	THR	A
224	352	SER	A
225	353	ARG	D
226	354	GLY	D
227	355	VAL	D

Continued on next page

Table S74 – *Continued from previous page*

Homology index	Residue Index	Residue abbreviation	Module
228	356	PRO	A
229	357	ALA	D
230	358	ILE	A
231	359	TYR	D
232	360	TYR	C
233	361	GLY	D
234	362	THR	A
235	376	ALA	C
236	377	LYS	C
237	378	MET	D
238	379	PRO	D
239	381	PHE	D
240	387	ALA	A
241	388	PHE	A
242	389	ASN	A
243	390	VAL	A
244	391	ILE	A
245	392	SER	A
246	393	LYS	A
247	394	LEU	A
248	395	ALA	A
249	396	PRO	A
250	397	LEU	A
251	398	ARG	A
252	399	LYS	A
253	400	SER	A
254	401	ASN	A
255	402	PRO	A
256	406	TYR	A
257	407	GLY	A
258	408	SER	A
259	409	THR	A
260	411	GLN	A
261	412	ARG	A
262	413	TRP	A
263	414	ILE	A
264	415	ASN	A
265	416	ASN	A
266	417	ASP	A
267	418	VAL	A
268	419	TYR	A
269	420	VAL	A
270	421	TYR	A
271	422	GLU	A
272	423	ARG	A
273	428	SER	A

Continued on next page

Table S74 – *Continued from previous page*

Homology index	Residue Index	Residue abbreviation	Module
274	429	VAL	A
275	430	ALA	A
276	431	VAL	A
277	432	VAL	A
278	433	ALA	A
279	434	VAL	A
280	435	ASN	A
281	439	SER	A
282	440	THR	A
283	441	SER	A
284	442	ALA	A
285	443	SER	A
286	444	ILE	A
287	445	THR	A
288	454	GLY	A
289	455	SER	A
290	457	THR	A
291	458	ASP	A
292	459	VAL	A
293	460	LEU	A
294	482	ALA	A
295	483	ALA	A
296	484	GLY	A
297	485	ALA	A
298	486	THR	A
299	487	ALA	A
300	488	VAL	A
301	489	TRP	A
302	490	GLN	A

Table S75: Residues membership for the *N. polysaccharea* α-amylase (PDB code 3UEQ, chain A)

Homology index	Residue Index	Residue abbreviation	Module
0	98	GLN	A
1	99	VAL	A
2	100	GLY	B
3	101	GLY	B
4	102	VAL	B
5	103	CYS	C
6	104	TYR	C
7	105	VAL	C
8	110	GLY	C

Continued on next page

Table S75 – *Continued from previous page*

Homology index	Residue Index	Residue abbreviation	Module
9	111	ASP	C
10	112	LEU	C
11	113	LYS	C
12	114	GLY	C
13	115	LEU	C
14	116	LYS	C
15	117	ASP	C
16	118	LYS	C
17	119	ILE	C
18	120	PRO	C
19	121	TYR	C
20	122	PHE	C
21	123	GLN	C
22	124	GLU	C
23	125	LEU	C
24	126	GLY	C
25	127	LEU	C
26	128	THR	C
27	129	TYR	C
28	130	LEU	C
29	131	HIS	C
30	132	LEU	C
31	133	MET	C
32	134	PRO	C
33	135	LEU	C
34	136	PHE	C
35	137	LYS	C
36	138	CYS	C
37	139	PRO	C
38	141	GLY	C
39	144	ASP	C
40	145	GLY	C
41	146	GLY	C
42	147	TYR	C
43	148	ALA	C
44	149	VAL	C
45	150	SER	C
46	151	SER	C
47	153	ARG	C
48	154	ASP	C
49	155	VAL	C
50	156	ASN	C
51	157	PRO	C
52	158	ALA	C
53	159	LEU	C
54	160	GLY	C

Continued on next page

Table S75 – *Continued from previous page*

Homology index	Residue Index	Residue abbreviation	Module
55	161	THR	C
56	162	ILE	C
57	163	GLY	C
58	164	ASP	C
59	165	LEU	C
60	166	ARG	C
61	167	GLU	C
62	168	VAL	C
63	169	ILE	C
64	170	ALA	C
65	171	ALA	C
66	172	LEU	C
67	173	HIS	C
68	174	GLU	C
69	175	ALA	C
70	176	GLY	C
71	177	ILE	C
72	178	SER	C
73	179	ALA	C
74	180	VAL	C
75	181	VAL	C
76	182	ASP	C
77	183	PHE	C
78	184	ILE	C
79	185	PHE	C
80	186	ASN	C
81	187	HIS	C
82	188	THR	C
83	189	SER	C
84	190	ASN	C
85	191	GLU	C
86	192	HIS	C
87	193	GLU	C
88	194	TRP	C
89	195	ALA	C
90	206	ASP	C
91	207	ASN	C
92	208	PHE	C
93	209	TYR	C
94	210	TYR	B
95	247	TRP	B
96	248	THR	B
97	249	THR	B
98	250	PHE	B
99	254	GLN	B
100	255	TRP	B

Continued on next page

Table S75 – *Continued from previous page*

Homology index	Residue Index	Residue abbreviation	Module
101	256	ASP	B
102	257	LEU	B
103	258	ASN	B
104	259	TYR	B
105	260	SER	B
106	261	ASN	B
107	262	PRO	B
108	263	TRP	B
109	264	VAL	B
110	265	PHE	B
111	266	ARG	B
112	267	ALA	B
113	268	MET	B
114	269	ALA	B
115	270	GLY	B
116	271	GLU	B
117	272	MET	B
118	273	LEU	B
119	274	PHE	B
120	275	LEU	B
121	276	ALA	B
122	277	ASN	B
123	278	LEU	C
124	279	GLY	C
125	280	VAL	C
126	281	ASP	C
127	282	ILE	C
128	283	LEU	B
129	284	ARG	D
130	285	MET	B
131	286	ASP	B
132	287	ALA	B
133	288	VAL	B
134	289	ALA	B
135	290	PHE	B
136	291	ILE	B
137	292	TRP	B
138	306	HIS	B
139	307	ALA	B
140	308	LEU	B
141	309	ILE	B
142	310	ARG	B
143	311	ALA	B
144	312	PHE	B
145	313	ASN	B
146	314	ALA	B

Continued on next page

Table S75 – *Continued from previous page*

Homology index	Residue Index	Residue abbreviation	Module
147	315	VAL	B
148	316	MET	B
149	317	ARG	B
150	323	VAL	B
151	324	PHE	B
152	325	PHE	B
153	326	LYS	C
154	327	SER	B
155	328	GLU	B
156	329	ALA	B
157	330	ILE	B
158	331	VAL	B
159	332	HIS	B
160	336	VAL	B
161	337	VAL	B
162	338	GLN	B
163	339	TYR	B
164	340	ILE	B
165	341	GLY	B
166	345	CYS	B
167	346	GLN	B
168	347	ILE	B
169	348	GLY	B
170	349	TYR	D
171	350	ASN	D
172	351	PRO	D
173	352	LEU	D
174	354	MET	D
175	355	ALA	D
176	356	LEU	D
177	357	LEU	D
178	358	TRP	D
179	359	ASN	D
180	360	THR	D
181	361	LEU	D
182	364	ARG	D
183	365	GLU	A
184	366	VAL	A
185	367	ASN	A
186	372	ALA	A
187	373	LEU	D
188	374	THR	D
189	375	TYR	D
190	376	ARG	D
191	377	HIS	B
192	378	ASN	B

Continued on next page

Table S75 – *Continued from previous page*

Homology index	Residue Index	Residue abbreviation	Module
193	381	GLU	B
194	382	HIS	B
195	383	THR	B
196	384	ALA	B
197	385	TRP	B
198	386	VAL	D
199	387	ASN	D
200	388	TYR	D
201	389	VAL	A
202	390	ARG	D
203	391	SER	D
204	392	HIS	D
205	393	ASP	D
206	394	ASP	D
207	448	SER	D
208	450	THR	D
209	451	ALA	D
210	452	ALA	D
211	453	ALA	D
212	464	ALA	A
213	467	ARG	A
214	468	ILE	A
215	469	LYS	A
216	470	LEU	A
217	471	LEU	A
218	472	TYR	A
219	473	SER	A
220	474	ILE	A
221	475	ALA	A
222	476	LEU	A
223	477	SER	A
224	478	THR	A
225	479	GLY	D
226	480	GLY	D
227	481	LEU	D
228	482	PRO	A
229	483	LEU	D
230	484	ILE	A
231	485	TYR	D
232	486	LEU	C
233	487	GLY	D
234	488	ASP	A
235	514	PRO	C
236	515	ARG	C
237	516	TYR	D
238	517	ASN	D

Continued on next page

Table S75 – *Continued from previous page*

Homology index	Residue Index	Residue abbreviation	Module
239	521	TYR	D
240	534	ILE	A
241	535	TYR	A
242	536	GLN	A
243	537	ASP	A
244	538	LEU	A
245	539	ARG	A
246	540	HIS	A
247	541	MET	A
248	542	ILE	A
249	543	ALA	A
250	544	VAL	A
251	545	ARG	A
252	546	GLN	A
253	547	SER	A
254	548	ASN	A
255	549	PRO	A
256	553	GLY	A
257	554	GLY	A
258	555	ARG	A
259	556	LEU	A
260	558	THR	A
261	559	PHE	A
262	560	ASN	A
263	561	THR	A
264	563	ASN	A
265	564	LYS	A
266	565	HIS	A
267	566	ILE	A
268	567	ILE	A
269	568	GLY	A
270	569	TYR	A
271	570	ILE	A
272	571	ARG	A
273	573	ASN	A
274	574	ALA	A
275	575	LEU	A
276	576	LEU	A
277	577	ALA	A
278	578	PHE	A
279	579	GLY	A
280	580	ASN	A
281	584	TYR	A
282	585	PRO	A
283	586	GLN	A
284	587	THR	A

Continued on next page

Table S75 – *Continued from previous page*

Homology index	Residue Index	Residue abbreviation	Module
285	588	VAL	A
286	589	THR	A
287	590	ALA	A
288	599	LYS	A
289	600	ALA	A
290	601	HIS	A
291	602	ASP	A
292	603	LEU	A
293	604	ILE	A
294	617	LEU	A
295	618	GLN	A
296	619	PRO	A
297	621	GLN	A
298	622	VAL	A
299	623	MET	A
300	624	TRP	A
301	625	LEU	A
302	626	GLU	A

Table S76: Residues membership for the *B. circulans,s8* α-amylase (PDB code 6CGT, chain A)

Homology index	Residue Index	Residue abbreviation	Module
0	15	ASP	A
1	16	VAL	A
2	17	ILE	B
3	18	TYR	B
4	19	GLN	B
5	20	VAL	C
6	21	PHE	C
7	22	THR	C
8	52	GLY	C
9	53	ASP	C
10	54	TRP	C
11	55	GLN	C
12	56	GLY	C
13	57	LEU	C
14	58	ILE	C
15	59	ASN	C
16	60	LYS	C
17	61	ILE	C
18	62	ASN	C
19	63	ASP	C

Continued on next page

Table S76 – *Continued from previous page*

**Homology index**	**Residue Index**	**Residue abbreviation**	**Module**
20	66	PHE	C
21	67	SER	C
22	68	ASP	C
23	69	LEU	C
24	70	GLY	C
25	71	VAL	C
26	72	THR	C
27	73	ALA	C
28	74	LEU	C
29	75	TRP	C
30	76	ILE	C
31	77	SER	C
32	78	GLN	C
33	79	PRO	C
34	80	VAL	C
35	81	GLU	C
36	82	ASN	C
37	83	ILE	C
38	95	THR	C
39	97	TYR	C
40	98	HIS	C
41	99	GLY	C
42	100	TYR	C
43	101	TRP	C
44	102	ALA	C
45	103	ARG	C
46	104	ASP	C
47	106	LYS	C
48	107	LYS	C
49	108	THR	C
50	109	ASN	C
51	110	PRO	C
52	111	TYR	C
53	112	PHE	C
54	113	GLY	C
55	114	THR	C
56	115	MET	C
57	116	ALA	C
58	117	ASP	C
59	118	PHE	C
60	119	GLN	C
61	120	ASN	C
62	121	LEU	C
63	122	ILE	C
64	123	THR	C
65	124	THR	C

Continued on next page

Table S76 – *Continued from previous page*

Homology index	Residue Index	Residue abbreviation	Module
66	125	ALA	C
67	126	HIS	C
68	127	ALA	C
69	128	LYS	C
70	129	GLY	C
71	130	ILE	C
72	131	LYS	C
73	132	ILE	C
74	133	VAL	C
75	134	ILE	C
76	135	ASP	C
77	136	PHE	C
78	137	ALA	C
79	138	PRO	C
80	139	ASN	C
81	140	HIS	C
82	141	THR	C
83	142	SER	C
84	143	PRO	C
85	144	ALA	C
86	156	ARG	C
87	163	LEU	C
88	164	VAL	C
89	165	GLY	C
90	172	ASN	C
91	173	GLY	C
92	174	TYR	C
93	175	PHE	C
94	176	HIS	B
95	192	LYS	B
96	193	ASN	B
97	194	THR	B
98	195	TYR	B
99	197	LEU	B
100	198	ALA	B
101	199	ASP	B
102	200	PHE	B
103	201	ASN	B
104	202	HIS	B
105	203	ASN	B
106	204	ASN	B
107	205	ALA	B
108	206	THR	B
109	207	ILE	B
110	208	ASP	B
111	209	LYS	B

Continued on next page

Table S76 – *Continued from previous page*

Homology index	Residue Index	Residue abbreviation	Module
112	210	TYR	B
113	211	PHE	B
114	212	LYS	B
115	213	ASP	B
116	214	ALA	B
117	215	ILE	B
118	216	LYS	B
119	217	LEU	B
120	218	TRP	B
121	219	LEU	B
122	220	ASP	B
123	221	MET	C
124	222	GLY	C
125	223	VAL	C
126	224	ASP	C
127	225	GLY	C
128	226	ILE	B
129	227	ARG	D
130	228	VAL	B
131	229	ASP	B
132	230	ALA	B
133	231	VAL	B
134	232	LYS	B
135	233	HIS	B
136	234	MET	B
137	235	PRO	B
138	236	LEU	B
139	237	GLY	B
140	238	TRP	B
141	239	GLN	B
142	240	LYS	B
143	241	SER	B
144	242	TRP	B
145	243	MET	B
146	244	SER	B
147	245	SER	B
148	246	ILE	B
149	247	TYR	B
150	252	VAL	B
151	253	PHE	B
152	254	THR	B
153	255	PHE	C
154	256	GLY	B
155	257	GLU	B
156	258	TRP	B
157	259	PHE	B

Continued on next page

Table S76 – *Continued from previous page*

Homology index	Residue Index	Residue abbreviation	Module
158	260	LEU	B
159	261	GLY	B
160	269	ASN	B
161	270	THR	B
162	271	ASP	B
163	272	PHE	B
164	274	ASN	B
165	275	LYS	B
166	277	GLY	B
167	278	MET	B
168	279	SER	B
169	280	LEU	B
170	281	LEU	D
171	282	ASP	D
172	283	PHE	D
173	284	ARG	D
174	286	ASN	D
175	287	SER	D
176	288	ALA	D
177	289	VAL	D
178	290	ARG	D
179	291	ASN	D
180	292	VAL	D
181	293	PHE	D
182	298	SER	D
183	299	ASN	A
184	300	MET	A
185	301	TYR	A
186	306	MET	A
187	307	ILE	D
188	308	ASN	D
189	309	SER	D
190	310	THR	D
191	311	ALA	B
192	312	THR	B
193	316	GLN	B
194	317	VAL	B
195	318	ASN	B
196	319	ASP	B
197	320	GLN	B
198	321	VAL	D
199	322	THR	D
200	323	PHE	D
201	324	ILE	A
202	325	ASP	D
203	326	ASN	D

Continued on next page

Table S76 – *Continued from previous page*

Homology index	Residue Index	Residue abbreviation	Module
204	327	HIS	D
205	328	ASP	D
206	329	MET	D
207	330	ASP	D
208	331	ARG	D
209	332	PHE	D
210	333	LYS	D
211	334	THR	D
212	339	ASN	A
213	341	ARG	A
214	342	LEU	A
215	343	GLU	A
216	344	GLN	A
217	345	ALA	A
218	346	LEU	A
219	347	ALA	A
220	348	PHE	A
221	349	THR	A
222	350	LEU	A
223	351	THR	A
224	352	SER	A
225	353	ARG	D
226	354	GLY	D
227	355	VAL	D
228	356	PRO	A
229	357	ALA	D
230	358	ILE	A
231	359	TYR	D
232	360	TYR	C
233	361	GLY	D
234	362	THR	A
235	376	ALA	C
236	377	LYS	C
237	378	MET	D
238	379	PRO	D
239	381	PHE	D
240	387	ALA	A
241	388	PHE	A
242	389	ASN	A
243	390	VAL	A
244	391	ILE	A
245	392	SER	A
246	393	LYS	A
247	394	LEU	A
248	395	ALA	A
249	396	PRO	A

Continued on next page

Table S76 – *Continued from previous page*

Homology index	Residue Index	Residue abbreviation	Module
250	397	LEU	A
251	398	ARG	A
252	399	LYS	A
253	400	SER	A
254	401	ASN	A
255	402	PRO	A
256	406	TYR	A
257	407	GLY	A
258	408	SER	A
259	409	THR	A
260	411	GLN	A
261	412	ARG	A
262	413	TRP	A
263	414	ILE	A
264	415	ASN	A
265	416	ASN	A
266	417	ASP	A
267	418	VAL	A
268	419	TYR	A
269	420	VAL	A
270	421	TYR	A
271	422	GLU	A
272	423	ARG	A
273	428	SER	A
274	429	VAL	A
275	430	ALA	A
276	431	VAL	A
277	432	VAL	A
278	433	ALA	A
279	434	VAL	A
280	435	ASN	A
281	439	SER	A
282	440	THR	A
283	441	SER	A
284	442	ALA	A
285	443	SER	A
286	444	ILE	A
287	445	THR	A
288	454	GLY	A
289	455	SER	A
290	457	THR	A
291	458	ASP	A
292	459	VAL	A
293	460	LEU	A
294	482	ALA	A
295	483	ALA	A

Continued on next page

Table S76 – *Continued from previous page*

Homology index	Residue Index	Residue abbreviation	Module
296	484	GLY	A
297	485	ALA	A
298	486	THR	A
299	487	ALA	A
300	488	VAL	A
301	489	TRP	A
302	490	GLN	A

Table S77: Residues membership for the *B. circulans,s8* α-amylase (PDB code 5CGT, chain A)

Homology index	Residue Index	Residue abbreviation	Module
0	15	ASP	A
1	16	VAL	A
2	17	ILE	B
3	18	TYR	B
4	19	GLN	B
5	20	VAL	C
6	21	PHE	C
7	22	THR	C
8	52	GLY	C
9	53	ASP	C
10	54	TRP	C
11	55	GLN	C
12	56	GLY	C
13	57	LEU	C
14	58	ILE	C
15	59	ASN	C
16	60	LYS	C
17	61	ILE	C
18	62	ASN	C
19	63	ASP	C
20	66	PHE	C
21	67	SER	C
22	68	ASP	C
23	69	LEU	C
24	70	GLY	C
25	71	VAL	C
26	72	THR	C
27	73	ALA	C
28	74	LEU	C
29	75	TRP	C
30	76	ILE	C

Continued on next page

Table S77 – *Continued from previous page*

Homology index	Residue Index	Residue abbreviation	Module
31	77	SER	C
32	78	GLN	C
33	79	PRO	C
34	80	VAL	C
35	81	GLU	C
36	82	ASN	C
37	83	ILE	C
38	95	THR	C
39	97	TYR	C
40	98	HIS	C
41	99	GLY	C
42	100	TYR	C
43	101	TRP	C
44	102	ALA	C
45	103	ARG	C
46	104	ASP	C
47	106	LYS	C
48	107	LYS	C
49	108	THR	C
50	109	ASN	C
51	110	PRO	C
52	111	TYR	C
53	112	PHE	C
54	113	GLY	C
55	114	THR	C
56	115	MET	C
57	116	ALA	C
58	117	ASP	C
59	118	PHE	C
60	119	GLN	C
61	120	ASN	C
62	121	LEU	C
63	122	ILE	C
64	123	THR	C
65	124	THR	C
66	125	ALA	C
67	126	HIS	C
68	127	ALA	C
69	128	LYS	C
70	129	GLY	C
71	130	ILE	C
72	131	LYS	C
73	132	ILE	C
74	133	VAL	C
75	134	ILE	C
76	135	ASP	C

Continued on next page

Table S77 – *Continued from previous page*

Homology index	Residue Index	Residue abbreviation	Module
77	136	PHE	C
78	137	ALA	C
79	138	PRO	C
80	139	ASN	C
81	140	HIS	C
82	141	THR	C
83	142	SER	C
84	143	PRO	C
85	144	ALA	C
86	156	ARG	C
87	163	LEU	C
88	164	VAL	C
89	165	GLY	C
90	172	ASN	C
91	173	GLY	C
92	174	TYR	C
93	175	PHE	C
94	176	HIS	B
95	192	LYS	B
96	193	ASN	B
97	194	LEU	B
98	195	TYR	B
99	197	LEU	B
100	198	ALA	B
101	199	ASP	B
102	200	PHE	B
103	201	ASN	B
104	202	HIS	B
105	203	ASN	B
106	204	ASN	B
107	205	ALA	B
108	206	THR	B
109	207	ILE	B
110	208	ASP	B
111	209	LYS	B
112	210	TYR	B
113	211	PHE	B
114	212	LYS	B
115	213	ASP	B
116	214	ALA	B
117	215	ILE	B
118	216	LYS	B
119	217	LEU	B
120	218	TRP	B
121	219	LEU	B
122	220	ASP	B

Continued on next page

Table S77 – *Continued from previous page*

Homology index	Residue Index	Residue abbreviation	Module
123	221	MET	C
124	222	GLY	C
125	223	VAL	C
126	224	ASP	C
127	225	GLY	C
128	226	ILE	B
129	227	ARG	D
130	228	VAL	B
131	229	ALA	B
132	230	ALA	B
133	231	VAL	B
134	232	LYS	B
135	233	HIS	B
136	234	MET	B
137	235	PRO	B
138	236	LEU	B
139	237	GLY	B
140	238	TRP	B
141	239	GLN	B
142	240	LYS	B
143	241	SER	B
144	242	TRP	B
145	243	MET	B
146	244	SER	B
147	245	SER	B
148	246	ILE	B
149	247	TYR	B
150	252	VAL	B
151	253	PHE	B
152	254	THR	B
153	255	PHE	C
154	256	GLY	B
155	257	GLU	B
156	258	TRP	B
157	259	PHE	B
158	260	LEU	B
159	261	GLY	B
160	269	ASN	B
161	270	THR	B
162	271	ASP	B
163	272	PHE	B
164	274	ASN	B
165	275	LYS	B
166	277	GLY	B
167	278	MET	B
168	279	SER	B

Continued on next page

Table S77 – *Continued from previous page*

Homology index	Residue Index	Residue abbreviation	Module
169	280	LEU	B
170	281	LEU	D
171	282	ASP	D
172	283	PHE	D
173	284	ARG	D
174	286	ASN	D
175	287	SER	D
176	288	ALA	D
177	289	VAL	D
178	290	ARG	D
179	291	ASN	D
180	292	VAL	D
181	293	PHE	D
182	298	SER	D
183	299	ASN	A
184	300	MET	A
185	301	TYR	A
186	306	MET	A
187	307	ILE	D
188	308	ASN	D
189	309	SER	D
190	310	THR	D
191	311	ALA	B
192	312	THR	B
193	316	GLN	B
194	317	VAL	B
195	318	ASN	B
196	319	ASP	B
197	320	GLN	B
198	321	VAL	D
199	322	THR	D
200	323	PHE	D
201	324	ILE	A
202	325	ASP	D
203	326	ASN	D
204	327	HIS	D
205	328	ASP	D
206	329	MET	D
207	330	ASP	D
208	331	ARG	D
209	332	PHE	D
210	333	LYS	D
211	334	THR	D
212	339	ASN	A
213	341	ARG	A
214	342	LEU	A

Continued on next page

Table S77 – *Continued from previous page*

Homology index	Residue Index	Residue abbreviation	Module
215	343	GLU	A
216	344	GLN	A
217	345	ALA	A
218	346	LEU	A
219	347	ALA	A
220	348	PHE	A
221	349	THR	A
222	350	LEU	A
223	351	THR	A
224	352	SER	A
225	353	ARG	D
226	354	GLY	D
227	355	VAL	D
228	356	PRO	A
229	357	ALA	D
230	358	ILE	A
231	359	TYR	D
232	360	TYR	C
233	361	GLY	D
234	362	THR	A
235	376	ALA	C
236	377	LYS	C
237	378	MET	D
238	379	PRO	D
239	381	PHE	D
240	387	ALA	A
241	388	PHE	A
242	389	ASN	A
243	390	VAL	A
244	391	ILE	A
245	392	SER	A
246	393	LYS	A
247	394	LEU	A
248	395	ALA	A
249	396	PRO	A
250	397	LEU	A
251	398	ARG	A
252	399	LYS	A
253	400	SER	A
254	401	ASN	A
255	402	PRO	A
256	406	TYR	A
257	407	GLY	A
258	408	SER	A
259	409	THR	A
260	411	GLN	A

Continued on next page

Table S77 – *Continued from previous page*

**Homology index**	**Residue Index**	**Residue abbreviation**	**Module**
261	412	ARG	A
262	413	TRP	A
263	414	ILE	A
264	415	ASN	A
265	416	ASN	A
266	417	ASP	A
267	418	VAL	A
268	419	TYR	A
269	420	VAL	A
270	421	TYR	A
271	422	GLU	A
272	423	ARG	A
273	428	SER	A
274	429	VAL	A
275	430	ALA	A
276	431	VAL	A
277	432	VAL	A
278	433	ALA	A
279	434	VAL	A
280	435	ASN	A
281	439	SER	A
282	440	THR	A
283	441	SER	A
284	442	ALA	A
285	443	SER	A
286	444	ILE	A
287	445	THR	A
288	454	GLY	A
289	455	SER	A
290	457	THR	A
291	458	ASP	A
292	459	VAL	A
293	460	LEU	A
294	482	ALA	A
295	483	ALA	A
296	484	GLY	A
297	485	ALA	A
298	486	THR	A
299	487	ALA	A
300	488	VAL	A
301	489	TRP	A
302	490	GLN	A

Table S78: Residues membership for the *B. circulans,s8* α-amylase (PDB code 7CGT, chain A)

Homology index	Residue Index	Residue abbreviation	Module
0	15	ASP	A
1	16	VAL	A
2	17	ILE	B
3	18	TYR	B
4	19	GLN	B
5	20	VAL	C
6	21	PHE	C
7	22	THR	C
8	52	GLY	C
9	53	ASP	C
10	54	TRP	C
11	55	GLN	C
12	56	GLY	C
13	57	LEU	C
14	58	ILE	C
15	59	ASN	C
16	60	LYS	C
17	61	ILE	C
18	62	ASN	C
19	63	ASP	C
20	66	PHE	C
21	67	SER	C
22	68	ASP	C
23	69	LEU	C
24	70	GLY	C
25	71	VAL	C
26	72	THR	C
27	73	ALA	C
28	74	LEU	C
29	75	TRP	C
30	76	ILE	C
31	77	SER	C
32	78	GLN	C
33	79	PRO	C
34	80	VAL	C
35	81	GLU	C
36	82	ASN	C
37	83	ILE	C
38	95	THR	C
39	97	TYR	C
40	98	HIS	C
41	99	GLY	C
42	100	TYR	C
43	101	TRP	C

Continued on next page

Table S78 – *Continued from previous page*

Homology index	Residue Index	Residue abbreviation	Module
44	102	ALA	C
45	103	ARG	C
46	104	ASP	C
47	106	LYS	C
48	107	LYS	C
49	108	THR	C
50	109	ASN	C
51	110	PRO	C
52	111	TYR	C
53	112	PHE	C
54	113	GLY	C
55	114	THR	C
56	115	MET	C
57	116	ALA	C
58	117	ASP	C
59	118	PHE	C
60	119	GLN	C
61	120	ASN	C
62	121	LEU	C
63	122	ILE	C
64	123	THR	C
65	124	THR	C
66	125	ALA	C
67	126	HIS	C
68	127	ALA	C
69	128	LYS	C
70	129	GLY	C
71	130	ILE	C
72	131	LYS	C
73	132	ILE	C
74	133	VAL	C
75	134	ILE	C
76	135	ASP	C
77	136	PHE	C
78	137	ALA	C
79	138	PRO	C
80	139	ASN	C
81	140	HIS	C
82	141	THR	C
83	142	SER	C
84	143	PRO	C
85	144	ALA	C
86	156	ARG	C
87	163	LEU	C
88	164	VAL	C
89	165	GLY	C

Continued on next page

Table S78 – *Continued from previous page*

Homology index	Residue Index	Residue abbreviation	Module
90	172	ASN	C
91	173	GLY	C
92	174	TYR	C
93	175	PHE	C
94	176	HIS	B
95	192	LYS	B
96	193	ASN	B
97	194	LEU	B
98	195	TYR	B
99	197	LEU	B
100	198	ALA	B
101	199	ASP	B
102	200	PHE	B
103	201	ASN	B
104	202	HIS	B
105	203	ASN	B
106	204	ASN	B
107	205	ALA	B
108	206	THR	B
109	207	ILE	B
110	208	ASP	B
111	209	LYS	B
112	210	TYR	B
113	211	PHE	B
114	212	LYS	B
115	213	ASP	B
116	214	ALA	B
117	215	ILE	B
118	216	LYS	B
119	217	LEU	B
120	218	TRP	B
121	219	LEU	B
122	220	ASP	B
123	221	MET	C
124	222	GLY	C
125	223	VAL	C
126	224	ASP	C
127	225	GLY	C
128	226	ILE	B
129	227	ARG	D
130	228	VAL	B
131	229	ALA	B
132	230	ALA	B
133	231	VAL	B
134	232	LYS	B
135	233	HIS	B

Continued on next page

Table S78 – *Continued from previous page*

Homology index	Residue Index	Residue abbreviation	Module
136	234	MET	B
137	235	PRO	B
138	236	LEU	B
139	237	GLY	B
140	238	TRP	B
141	239	GLN	B
142	240	LYS	B
143	241	SER	B
144	242	TRP	B
145	243	MET	B
146	244	SER	B
147	245	SER	B
148	246	ILE	B
149	247	TYR	B
150	252	VAL	B
151	253	PHE	B
152	254	THR	B
153	255	PHE	C
154	256	GLY	B
155	257	GLU	B
156	258	TRP	B
157	259	PHE	B
158	260	LEU	B
159	261	GLY	B
160	269	ASN	B
161	270	THR	B
162	271	ASP	B
163	272	PHE	B
164	274	ASN	B
165	275	LYS	B
166	277	GLY	B
167	278	MET	B
168	279	SER	B
169	280	LEU	B
170	281	LEU	D
171	282	ASP	D
172	283	PHE	D
173	284	ARG	D
174	286	ASN	D
175	287	SER	D
176	288	ALA	D
177	289	VAL	D
178	290	ARG	D
179	291	ASN	D
180	292	VAL	D
181	293	PHE	D

Continued on next page

Table S78 – *Continued from previous page*

Homology index	Residue Index	Residue abbreviation	Module
182	298	SER	D
183	299	ASN	A
184	300	MET	A
185	301	TYR	A
186	306	MET	A
187	307	ILE	D
188	308	ASN	D
189	309	SER	D
190	310	THR	D
191	311	ALA	B
192	312	THR	B
193	316	GLN	B
194	317	VAL	B
195	318	ASN	B
196	319	ASP	B
197	320	GLN	B
198	321	VAL	D
199	322	THR	D
200	323	PHE	D
201	324	ILE	A
202	325	ASP	D
203	326	ASN	D
204	327	HIS	D
205	328	ASP	D
206	329	MET	D
207	330	ASP	D
208	331	ARG	D
209	332	PHE	D
210	333	LYS	D
211	334	THR	D
212	339	ASN	A
213	341	ARG	A
214	342	LEU	A
215	343	GLU	A
216	344	GLN	A
217	345	ALA	A
218	346	LEU	A
219	347	ALA	A
220	348	PHE	A
221	349	THR	A
222	350	LEU	A
223	351	THR	A
224	352	SER	A
225	353	ARG	D
226	354	GLY	D
227	355	VAL	D

Continued on next page

Table S78 – *Continued from previous page*

Homology index	Residue Index	Residue abbreviation	Module
228	356	PRO	A
229	357	ALA	D
230	358	ILE	A
231	359	TYR	D
232	360	TYR	C
233	361	GLY	D
234	362	THR	A
235	376	ALA	C
236	377	LYS	C
237	378	MET	D
238	379	PRO	D
239	381	PHE	D
240	387	ALA	A
241	388	PHE	A
242	389	ASN	A
243	390	VAL	A
244	391	ILE	A
245	392	SER	A
246	393	LYS	A
247	394	LEU	A
248	395	ALA	A
249	396	PRO	A
250	397	LEU	A
251	398	ARG	A
252	399	LYS	A
253	400	SER	A
254	401	ASN	A
255	402	PRO	A
256	406	TYR	A
257	407	GLY	A
258	408	SER	A
259	409	THR	A
260	411	GLN	A
261	412	ARG	A
262	413	TRP	A
263	414	ILE	A
264	415	ASN	A
265	416	ASN	A
266	417	ASP	A
267	418	VAL	A
268	419	TYR	A
269	420	VAL	A
270	421	TYR	A
271	422	GLU	A
272	423	ARG	A
273	428	SER	A

Continued on next page

Table S78 – *Continued from previous page*

Homology index	Residue Index	Residue abbreviation	Module
274	429	VAL	A
275	430	ALA	A
276	431	VAL	A
277	432	VAL	A
278	433	ALA	A
279	434	VAL	A
280	435	ASN	A
281	439	SER	A
282	440	THR	A
283	441	SER	A
284	442	ALA	A
285	443	SER	A
286	444	ILE	A
287	445	THR	A
288	454	GLY	A
289	455	SER	A
290	457	THR	A
291	458	ASP	A
292	459	VAL	A
293	460	LEU	A
294	482	ALA	A
295	483	ALA	A
296	484	GLY	A
297	485	ALA	A
298	486	THR	A
299	487	ALA	A
300	488	VAL	A
301	489	TRP	A
302	490	GLN	A

Table S79: Residues membership for the *A. oryzae* α-amylase (PDB code 6TAA, chain A)

Homology index	Residue Index	Residue abbreviation	Module
0	9	GLN	A
1	10	SER	A
2	11	ILE	B
3	12	TYR	B
4	13	PHE	B
5	14	LEU	C
6	15	LEU	C
7	16	THR	C
8	40	GLY	C

Continued on next page

Table S79 – *Continued from previous page*

Homology index	Residue Index	Residue abbreviation	Module
9	41	THR	C
10	42	TRP	C
11	43	GLN	C
12	44	GLY	C
13	45	ILE	C
14	46	ILE	C
15	47	ASP	C
16	48	LYS	C
17	49	LEU	C
18	50	ASP	C
19	51	TYR	C
20	52	ILE	C
21	53	GLN	C
22	54	GLY	C
23	55	MET	C
24	56	GLY	C
25	57	PHE	C
26	58	THR	C
27	59	ALA	C
28	60	ILE	C
29	61	TRP	C
30	62	ILE	C
31	63	THR	C
32	64	PRO	C
33	65	VAL	C
34	66	THR	C
35	67	ALA	C
36	68	GLN	C
37	69	LEU	C
38	77	ASP	C
39	79	TYR	C
40	80	HIS	C
41	81	GLY	C
42	82	TYR	C
43	83	TRP	C
44	84	GLN	C
45	85	GLN	C
46	86	ASP	C
47	88	TYR	C
48	89	SER	C
49	90	LEU	C
50	91	ASN	C
51	92	GLU	C
52	93	ASN	C
53	94	TYR	C
54	95	GLY	C

Continued on next page

Table S79 – *Continued from previous page*

Homology index	Residue Index	Residue abbreviation	Module
55	96	THR	C
56	97	ALA	C
57	98	ASP	C
58	99	ASP	C
59	100	LEU	C
60	101	LYS	C
61	102	ALA	C
62	103	LEU	C
63	104	SER	C
64	105	SER	C
65	106	ALA	C
66	107	LEU	C
67	108	HIS	C
68	109	GLU	C
69	110	ARG	C
70	111	GLY	C
71	112	MET	C
72	113	TYR	C
73	114	LEU	C
74	115	MET	C
75	116	VAL	C
76	117	ASP	C
77	118	VAL	C
78	119	VAL	C
79	120	ALA	C
80	121	ASN	C
81	122	HIS	C
82	123	MET	C
83	124	GLY	C
84	125	TYR	C
85	126	ASP	C
86	137	PHE	C
87	139	PRO	C
88	140	PHE	C
89	141	SER	C
90	143	GLN	C
91	144	ASP	C
92	145	TYR	C
93	146	PHE	C
94	147	HIS	B
95	164	CYS	B
96	165	TRP	B
97	166	LEU	B
98	167	GLY	B
99	173	LEU	B
100	174	PRO	B

Continued on next page

Table S79 – *Continued from previous page*

Homology index	Residue Index	Residue abbreviation	Module
101	175	ASP	B
102	176	LEU	B
103	177	ASP	B
104	178	THR	B
105	179	THR	B
106	180	LYS	B
107	181	ASP	B
108	182	VAL	B
109	183	VAL	B
110	184	LYS	B
111	185	ASN	B
112	186	GLU	B
113	187	TRP	B
114	188	TYR	B
115	189	ASP	B
116	190	TRP	B
117	191	VAL	B
118	192	GLY	B
119	193	SER	B
120	194	LEU	B
121	195	VAL	B
122	196	SER	B
123	198	TYR	C
124	199	SER	C
125	200	ILE	C
126	201	ASP	C
127	202	GLY	C
128	203	LEU	B
129	204	ARG	D
130	205	ILE	B
131	206	ASP	B
132	207	THR	B
133	208	VAL	B
134	209	LYS	B
135	210	HIS	B
136	211	VAL	B
137	212	GLN	B
138	213	LYS	B
139	214	ASP	B
140	215	PHE	B
141	216	TRP	B
142	217	PRO	B
143	218	GLY	B
144	219	TYR	B
145	220	ASN	B
146	221	LYS	B

Continued on next page

Table S79 – *Continued from previous page*

Homology index	Residue Index	Residue abbreviation	Module
147	222	ALA	B
148	223	ALA	B
149	224	GLY	B
150	225	VAL	B
151	226	TYR	B
152	227	CYS	B
153	228	ILE	C
154	229	GLY	B
155	230	GLU	B
156	231	VAL	B
157	232	LEU	B
158	233	ASP	B
159	234	GLY	B
160	239	THR	B
161	240	CYS	B
162	241	PRO	B
163	242	TYR	B
164	243	GLN	B
165	244	ASN	B
166	246	MET	B
167	247	ASP	B
168	248	GLY	B
169	249	VAL	B
170	250	LEU	D
171	251	ASN	D
172	252	TYR	D
173	253	PRO	D
174	255	TYR	D
175	256	TYR	D
176	257	PRO	D
177	258	LEU	D
178	259	LEU	D
179	260	ASN	D
180	261	ALA	D
181	262	PHE	D
182	267	GLY	D
183	268	SER	A
184	269	MET	A
185	270	ASP	A
186	275	MET	A
187	276	ILE	D
188	277	ASN	D
189	278	THR	D
190	279	VAL	D
191	280	LYS	B
192	281	SER	B

Continued on next page

Table S79 – *Continued from previous page*

Homology index	Residue Index	Residue abbreviation	Module
193	285	ASP	B
194	286	SER	B
195	287	THR	B
196	288	LEU	B
197	289	LEU	B
198	290	GLY	D
199	291	THR	D
200	292	PHE	D
201	293	VAL	A
202	294	GLU	D
203	295	ASN	D
204	296	HIS	D
205	297	ASP	D
206	298	ASN	D
207	299	PRO	D
208	300	ARG	D
209	301	PHE	D
210	302	ALA	D
211	303	SER	D
212	308	ILE	A
213	310	LEU	A
214	311	ALA	A
215	312	LYS	A
216	313	ASN	A
217	314	VAL	A
218	315	ALA	A
219	316	ALA	A
220	317	PHE	A
221	318	ILE	A
222	319	ILE	A
223	320	LEU	A
224	321	ASN	A
225	322	ASP	D
226	323	GLY	D
227	324	ILE	D
228	325	PRO	A
229	326	ILE	D
230	327	ILE	A
231	328	TYR	D
232	329	ALA	C
233	330	GLY	D
234	331	GLN	A
235	345	GLU	C
236	346	ALA	C
237	347	THR	D
238	348	TRP	D

Continued on next page

Table S79 – *Continued from previous page*

Homology index	Residue Index	Residue abbreviation	Module
239	352	TYR	D
240	358	LEU	A
241	359	TYR	A
242	360	LYS	A
243	361	LEU	A
244	362	ILE	A
245	363	ALA	A
246	364	SER	A
247	365	ALA	A
248	366	ASN	A
249	367	ALA	A
250	368	ILE	A
251	373	ILE	A
252	374	SER	A
253	375	LYS	A
254	376	ASP	A
255	377	THR	A
256	381	THR	A
257	382	TYR	A
258	383	LYS	A
259	384	ASN	A
260	386	PRO	A
261	387	ILE	A
262	388	TYR	A
263	389	LYS	A
264	390	ASP	A
265	391	ASP	A
266	392	THR	A
267	393	THR	A
268	394	ILE	A
269	395	ALA	A
270	396	MET	A
271	397	ARG	A
272	398	LYS	A
273	403	SER	A
274	404	GLN	A
275	405	ILE	A
276	406	VAL	A
277	407	THR	A
278	408	ILE	A
279	409	LEU	A
280	410	SER	A
281	416	GLY	A
282	417	ASP	A
283	418	SER	A
284	419	TYR	A

Continued on next page

Table S79 – *Continued from previous page*

Homology index	Residue Index	Residue abbreviation	Module
285	420	THR	A
286	421	LEU	A
287	422	SER	A
288	432	GLN	A
289	433	GLN	A
290	435	THR	A
291	436	GLU	A
292	437	VAL	A
293	438	ILE	A
294	456	ALA	A
295	457	GLY	A
296	458	GLY	A
297	459	LEU	A
298	460	PRO	A
299	461	ARG	A
300	462	VAL	A
301	463	LEU	A
302	464	TYR	A

Table S80: Residues membership for the *B. circulans,s8* α-amylase (PDB code 8CGT, chain A)

Homology index	Residue Index	Residue abbreviation	Module
0	15	ASP	A
1	16	VAL	A
2	17	ILE	B
3	18	TYR	B
4	19	GLN	B
5	20	VAL	C
6	21	PHE	C
7	22	THR	C
8	52	GLY	C
9	53	ASP	C
10	54	TRP	C
11	55	GLN	C
12	56	GLY	C
13	57	LEU	C
14	58	ILE	C
15	59	ASN	C
16	60	LYS	C
17	61	ILE	C
18	62	ASN	C
19	63	ASP	C

Continued on next page

Table S80 – *Continued from previous page*

Homology index	Residue Index	Residue abbreviation	Module
20	66	PHE	C
21	67	SER	C
22	68	ASP	C
23	69	LEU	C
24	70	GLY	C
25	71	VAL	C
26	72	THR	C
27	73	ALA	C
28	74	LEU	C
29	75	TRP	C
30	76	ILE	C
31	77	SER	C
32	78	GLN	C
33	79	PRO	C
34	80	VAL	C
35	81	GLU	C
36	82	ASN	C
37	83	ILE	C
38	95	THR	C
39	97	TYR	C
40	98	HIS	C
41	99	GLY	C
42	100	TYR	C
43	101	TRP	C
44	102	ALA	C
45	103	ARG	C
46	104	ASP	C
47	106	LYS	C
48	107	LYS	C
49	108	THR	C
50	109	ASN	C
51	110	PRO	C
52	111	TYR	C
53	112	PHE	C
54	113	GLY	C
55	114	THR	C
56	115	MET	C
57	116	ALA	C
58	117	ASP	C
59	118	PHE	C
60	119	GLN	C
61	120	ASN	C
62	121	LEU	C
63	122	ILE	C
64	123	THR	C
65	124	THR	C

Continued on next page

Table S80 – *Continued from previous page*

Homology index	Residue Index	Residue abbreviation	Module
66	125	ALA	C
67	126	HIS	C
68	127	ALA	C
69	128	LYS	C
70	129	GLY	C
71	130	ILE	C
72	131	LYS	C
73	132	ILE	C
74	133	VAL	C
75	134	ILE	C
76	135	ASP	C
77	136	PHE	C
78	137	ALA	C
79	138	PRO	C
80	139	ASN	C
81	140	HIS	C
82	141	THR	C
83	142	SER	C
84	143	PRO	C
85	144	ALA	C
86	156	ARG	C
87	163	LEU	C
88	164	VAL	C
89	165	GLY	C
90	172	ASN	C
91	173	GLY	C
92	174	TYR	C
93	175	PHE	C
94	176	HIS	B
95	192	LYS	B
96	193	ASN	B
97	194	LEU	B
98	195	TYR	B
99	197	LEU	B
100	198	ALA	B
101	199	ASP	B
102	200	PHE	B
103	201	ASN	B
104	202	HIS	B
105	203	ASN	B
106	204	ASN	B
107	205	ALA	B
108	206	THR	B
109	207	ILE	B
110	208	ASP	B
111	209	LYS	B

Continued on next page

Table S80 – *Continued from previous page*

Homology index	Residue Index	Residue abbreviation	Module
112	210	TYR	B
113	211	PHE	B
114	212	LYS	B
115	213	ASP	B
116	214	ALA	B
117	215	ILE	B
118	216	LYS	B
119	217	LEU	B
120	218	TRP	B
121	219	LEU	B
122	220	ASP	B
123	221	MET	C
124	222	GLY	C
125	223	VAL	C
126	224	ASP	C
127	225	GLY	C
128	226	ILE	B
129	227	ARG	D
130	228	VAL	B
131	229	ASP	B
132	230	ALA	B
133	231	VAL	B
134	232	LYS	B
135	233	HIS	B
136	234	MET	B
137	235	PRO	B
138	236	LEU	B
139	237	GLY	B
140	238	TRP	B
141	239	GLN	B
142	240	LYS	B
143	241	SER	B
144	242	TRP	B
145	243	MET	B
146	244	SER	B
147	245	SER	B
148	246	ILE	B
149	247	TYR	B
150	252	VAL	B
151	253	PHE	B
152	254	THR	B
153	255	PHE	C
154	256	GLY	B
155	257	ALA	B
156	258	TRP	B
157	259	PHE	B

Continued on next page

Table S80 – *Continued from previous page*

Homology index	Residue Index	Residue abbreviation	Module
158	260	LEU	B
159	261	GLY	B
160	269	ASN	B
161	270	THR	B
162	271	ASP	B
163	272	PHE	B
164	274	ASN	B
165	275	LYS	B
166	277	GLY	B
167	278	MET	B
168	279	SER	B
169	280	LEU	B
170	281	LEU	D
171	282	ASP	D
172	283	PHE	D
173	284	ARG	D
174	286	ASN	D
175	287	SER	D
176	288	ALA	D
177	289	VAL	D
178	290	ARG	D
179	291	ASN	D
180	292	VAL	D
181	293	PHE	D
182	298	SER	D
183	299	ASN	A
184	300	MET	A
185	301	TYR	A
186	306	MET	A
187	307	ILE	D
188	308	ASN	D
189	309	SER	D
190	310	THR	D
191	311	ALA	B
192	312	THR	B
193	316	GLN	B
194	317	VAL	B
195	318	ASN	B
196	319	ASP	B
197	320	GLN	B
198	321	VAL	D
199	322	THR	D
200	323	PHE	D
201	324	ILE	A
202	325	ASP	D
203	326	ASN	D

Continued on next page

Table S80 – *Continued from previous page*

Homology index	Residue Index	Residue abbreviation	Module
204	327	HIS	D
205	328	ASP	D
206	329	MET	D
207	330	ASP	D
208	331	ARG	D
209	332	PHE	D
210	333	LYS	D
211	334	THR	D
212	339	ASN	A
213	341	ARG	A
214	342	LEU	A
215	343	GLU	A
216	344	GLN	A
217	345	ALA	A
218	346	LEU	A
219	347	ALA	A
220	348	PHE	A
221	349	THR	A
222	350	LEU	A
223	351	THR	A
224	352	SER	A
225	353	ARG	D
226	354	GLY	D
227	355	VAL	D
228	356	PRO	A
229	357	ALA	D
230	358	ILE	A
231	359	TYR	D
232	360	TYR	C
233	361	GLY	D
234	362	THR	A
235	376	ALA	C
236	377	LYS	C
237	378	MET	D
238	379	PRO	D
239	381	PHE	D
240	387	ALA	A
241	388	PHE	A
242	389	ASN	A
243	390	VAL	A
244	391	ILE	A
245	392	SER	A
246	393	LYS	A
247	394	LEU	A
248	395	ALA	A
249	396	PRO	A

Continued on next page

Table S80 – *Continued from previous page*

Homology index	Residue Index	Residue abbreviation	Module
250	397	LEU	A
251	398	ARG	A
252	399	LYS	A
253	400	SER	A
254	401	ASN	A
255	402	PRO	A
256	406	TYR	A
257	407	GLY	A
258	408	SER	A
259	409	THR	A
260	411	GLN	A
261	412	ARG	A
262	413	TRP	A
263	414	ILE	A
264	415	ASN	A
265	416	ASN	A
266	417	ASP	A
267	418	VAL	A
268	419	TYR	A
269	420	VAL	A
270	421	TYR	A
271	422	GLU	A
272	423	ARG	A
273	428	SER	A
274	429	VAL	A
275	430	ALA	A
276	431	VAL	A
277	432	VAL	A
278	433	ALA	A
279	434	VAL	A
280	435	ASN	A
281	439	SER	A
282	440	THR	A
283	441	SER	A
284	442	ALA	A
285	443	SER	A
286	444	ILE	A
287	445	THR	A
288	454	GLY	A
289	455	SER	A
290	457	THR	A
291	458	ASP	A
292	459	VAL	A
293	460	LEU	A
294	482	ALA	A
295	483	ALA	A

Continued on next page

Table S80 – *Continued from previous page*

Homology index	Residue Index	Residue abbreviation	Module
296	484	GLY	A
297	485	ALA	A
298	486	THR	A
299	487	ALA	A
300	488	VAL	A
301	489	TRP	A
302	490	GLN	A

Table S81: Residues membership for the *A. oryzae* α-amylase (PDB code 7TAA, chain A)

Homology index	Residue Index	Residue abbreviation	Module
0	9	GLN	A
1	10	SER	A
2	11	ILE	B
3	12	TYR	B
4	13	PHE	B
5	14	LEU	C
6	15	LEU	C
7	16	THR	C
8	40	GLY	C
9	41	THR	C
10	42	TRP	C
11	43	GLN	C
12	44	GLY	C
13	45	ILE	C
14	46	ILE	C
15	47	ASP	C
16	48	LYS	C
17	49	LEU	C
18	50	ASP	C
19	51	TYR	C
20	52	ILE	C
21	53	GLN	C
22	54	GLY	C
23	55	MET	C
24	56	GLY	C
25	57	PHE	C
26	58	THR	C
27	59	ALA	C
28	60	ILE	C
29	61	TRP	C
30	62	ILE	C

Continued on next page

Table S81 – *Continued from previous page*

Homology index	Residue Index	Residue abbreviation	Module
31	63	THR	C
32	64	PRO	C
33	65	VAL	C
34	66	THR	C
35	67	ALA	C
36	68	GLN	C
37	69	LEU	C
38	77	ASP	C
39	79	TYR	C
40	80	HIS	C
41	81	GLY	C
42	82	TYR	C
43	83	TRP	C
44	84	GLN	C
45	85	GLN	C
46	86	ASP	C
47	88	TYR	C
48	89	SER	C
49	90	LEU	C
50	91	ASN	C
51	92	GLU	C
52	93	ASN	C
53	94	TYR	C
54	95	GLY	C
55	96	THR	C
56	97	ALA	C
57	98	ASP	C
58	99	ASP	C
59	100	LEU	C
60	101	LYS	C
61	102	ALA	C
62	103	LEU	C
63	104	SER	C
64	105	SER	C
65	106	ALA	C
66	107	LEU	C
67	108	HIS	C
68	109	GLU	C
69	110	ARG	C
70	111	GLY	C
71	112	MET	C
72	113	TYR	C
73	114	LEU	C
74	115	MET	C
75	116	VAL	C
76	117	ASP	C

Continued on next page

Table S81 – *Continued from previous page*

Homology index	Residue Index	Residue abbreviation	Module
77	118	VAL	C
78	119	VAL	C
79	120	ALA	C
80	121	ASN	C
81	122	HIS	C
82	123	MET	C
83	124	GLY	C
84	125	TYR	C
85	126	ASP	C
86	137	PHE	C
87	139	PRO	C
88	140	PHE	C
89	141	SER	C
90	143	GLN	C
91	144	ASP	C
92	145	TYR	C
93	146	PHE	C
94	147	HIS	B
95	164	CYS	B
96	165	TRP	B
97	166	LEU	B
98	167	GLY	B
99	173	LEU	B
100	174	PRO	B
101	175	ASP	B
102	176	LEU	B
103	177	ASP	B
104	178	THR	B
105	179	THR	B
106	180	LYS	B
107	181	ASP	B
108	182	VAL	B
109	183	VAL	B
110	184	LYS	B
111	185	ASN	B
112	186	GLU	B
113	187	TRP	B
114	188	TYR	B
115	189	ASP	B
116	190	TRP	B
117	191	VAL	B
118	192	GLY	B
119	193	SER	B
120	194	LEU	B
121	195	VAL	B
122	196	SER	B

Continued on next page

Table S81 – *Continued from previous page*

Homology index	Residue Index	Residue abbreviation	Module
123	198	TYR	C
124	199	SER	C
125	200	ILE	C
126	201	ASP	C
127	202	GLY	C
128	203	LEU	B
129	204	ARG	D
130	205	ILE	B
131	206	ASP	B
132	207	THR	B
133	208	VAL	B
134	209	LYS	B
135	210	HIS	B
136	211	VAL	B
137	212	GLN	B
138	213	LYS	B
139	214	ASP	B
140	215	PHE	B
141	216	TRP	B
142	217	PRO	B
143	218	GLY	B
144	219	TYR	B
145	220	ASN	B
146	221	LYS	B
147	222	ALA	B
148	223	ALA	B
149	224	GLY	B
150	225	VAL	B
151	226	TYR	B
152	227	CYS	B
153	228	ILE	C
154	229	GLY	B
155	230	GLU	B
156	231	VAL	B
157	232	LEU	B
158	233	ASP	B
159	234	GLY	B
160	239	THR	B
161	240	CYS	B
162	241	PRO	B
163	242	TYR	B
164	243	GLN	B
165	244	ASN	B
166	246	MET	B
167	247	ASP	B
168	248	GLY	B

Continued on next page

Table S81 – *Continued from previous page*

Homology index	Residue Index	Residue abbreviation	Module
169	249	VAL	B
170	250	LEU	D
171	251	ASN	D
172	252	TYR	D
173	253	PRO	D
174	255	TYR	D
175	256	TYR	D
176	257	PRO	D
177	258	LEU	D
178	259	LEU	D
179	260	ASN	D
180	261	ALA	D
181	262	PHE	D
182	267	GLY	D
183	268	SER	A
184	269	MET	A
185	270	ASP	A
186	275	MET	A
187	276	ILE	D
188	277	ASN	D
189	278	THR	D
190	279	VAL	D
191	280	LYS	B
192	281	SER	B
193	285	ASP	B
194	286	SER	B
195	287	THR	B
196	288	LEU	B
197	289	LEU	B
198	290	GLY	D
199	291	THR	D
200	292	PHE	D
201	293	VAL	A
202	294	GLU	D
203	295	ASN	D
204	296	HIS	D
205	297	ASP	D
206	298	ASN	D
207	299	PRO	D
208	300	ARG	D
209	301	PHE	D
210	302	ALA	D
211	303	SER	D
212	308	ILE	A
213	310	LEU	A
214	311	ALA	A

Continued on next page

Table S81 – *Continued from previous page*

Homology index	Residue Index	Residue abbreviation	Module
215	312	LYS	A
216	313	ASN	A
217	314	VAL	A
218	315	ALA	A
219	316	ALA	A
220	317	PHE	A
221	318	ILE	A
222	319	ILE	A
223	320	LEU	A
224	321	ASN	A
225	322	ASP	D
226	323	GLY	D
227	324	ILE	D
228	325	PRO	A
229	326	ILE	D
230	327	ILE	A
231	328	TYR	D
232	329	ALA	C
233	330	GLY	D
234	331	GLN	A
235	345	GLU	C
236	346	ALA	C
237	347	THR	D
238	348	TRP	D
239	352	TYR	D
240	358	LEU	A
241	359	TYR	A
242	360	LYS	A
243	361	LEU	A
244	362	ILE	A
245	363	ALA	A
246	364	SER	A
247	365	ALA	A
248	366	ASN	A
249	367	ALA	A
250	368	ILE	A
251	373	ILE	A
252	374	SER	A
253	375	LYS	A
254	376	ASP	A
255	377	THR	A
256	381	THR	A
257	382	TYR	A
258	383	LYS	A
259	384	ASN	A
260	386	PRO	A

Continued on next page

Table S81 – *Continued from previous page*

Homology index	Residue Index	Residue abbreviation	Module
261	387	ILE	A
262	388	TYR	A
263	389	LYS	A
264	390	ASP	A
265	391	ASP	A
266	392	THR	A
267	393	THR	A
268	394	ILE	A
269	395	ALA	A
270	396	MET	A
271	397	ARG	A
272	398	LYS	A
273	403	SER	A
274	404	GLN	A
275	405	ILE	A
276	406	VAL	A
277	407	THR	A
278	408	ILE	A
279	409	LEU	A
280	410	SER	A
281	416	GLY	A
282	417	ASP	A
283	418	SER	A
284	419	TYR	A
285	420	THR	A
286	421	LEU	A
287	422	SER	A
288	432	GLN	A
289	433	GLN	A
290	435	THR	A
291	436	GLU	A
292	437	VAL	A
293	438	ILE	A
294	456	ALA	A
295	457	GLY	A
296	458	GLY	A
297	459	LEU	A
298	460	PRO	A
299	461	ARG	A
300	462	VAL	A
301	463	LEU	A
302	464	TYR	A

Table S82: Residues membership for the *B. circulans,s8* α-amylase (PDB code 9CGT, chain A)

Homology index	Residue Index	Residue abbreviation	Module
0	15	ASP	A
1	16	VAL	A
2	17	ILE	B
3	18	TYR	B
4	19	GLN	B
5	20	VAL	C
6	21	PHE	C
7	22	THR	C
8	52	GLY	C
9	53	ASP	C
10	54	TRP	C
11	55	GLN	C
12	56	GLY	C
13	57	LEU	C
14	58	ILE	C
15	59	ASN	C
16	60	LYS	C
17	61	ILE	C
18	62	ASN	C
19	63	ASP	C
20	66	PHE	C
21	67	SER	C
22	68	ASP	C
23	69	LEU	C
24	70	GLY	C
25	71	VAL	C
26	72	THR	C
27	73	ALA	C
28	74	LEU	C
29	75	TRP	C
30	76	ILE	C
31	77	SER	C
32	78	GLN	C
33	79	PRO	C
34	80	VAL	C
35	81	GLU	C
36	82	ASN	C
37	83	ILE	C
38	95	THR	C
39	97	TYR	C
40	98	HIS	C
41	99	GLY	C
42	100	TYR	C
43	101	TRP	C

Continued on next page

Table S82 – *Continued from previous page*

Homology index	Residue Index	Residue abbreviation	Module
44	102	ALA	C
45	103	ARG	C
46	104	ASP	C
47	106	LYS	C
48	107	LYS	C
49	108	THR	C
50	109	ASN	C
51	110	PRO	C
52	111	TYR	C
53	112	PHE	C
54	113	GLY	C
55	114	THR	C
56	115	MET	C
57	116	ALA	C
58	117	ASP	C
59	118	PHE	C
60	119	GLN	C
61	120	ASN	C
62	121	LEU	C
63	122	ILE	C
64	123	THR	C
65	124	THR	C
66	125	ALA	C
67	126	HIS	C
68	127	ALA	C
69	128	LYS	C
70	129	GLY	C
71	130	ILE	C
72	131	LYS	C
73	132	ILE	C
74	133	VAL	C
75	134	ILE	C
76	135	ASP	C
77	136	PHE	C
78	137	ALA	C
79	138	PRO	C
80	139	ASN	C
81	140	HIS	C
82	141	THR	C
83	142	SER	C
84	143	PRO	C
85	144	ALA	C
86	156	ARG	C
87	163	LEU	C
88	164	VAL	C
89	165	GLY	C

Continued on next page

Table S82 – *Continued from previous page*

Homology index	Residue Index	Residue abbreviation	Module
90	172	ASN	C
91	173	GLY	C
92	174	TYR	C
93	175	PHE	C
94	176	HIS	B
95	192	LYS	B
96	193	ASN	B
97	194	LEU	B
98	195	TYR	B
99	197	LEU	B
100	198	ALA	B
101	199	ASP	B
102	200	PHE	B
103	201	ASN	B
104	202	HIS	B
105	203	ASN	B
106	204	ASN	B
107	205	ALA	B
108	206	THR	B
109	207	ILE	B
110	208	ASP	B
111	209	LYS	B
112	210	TYR	B
113	211	PHE	B
114	212	LYS	B
115	213	ASP	B
116	214	ALA	B
117	215	ILE	B
118	216	LYS	B
119	217	LEU	B
120	218	TRP	B
121	219	LEU	B
122	220	ASP	B
123	221	MET	C
124	222	GLY	C
125	223	VAL	C
126	224	ASP	C
127	225	GLY	C
128	226	ILE	B
129	227	ARG	D
130	228	VAL	B
131	229	ASP	B
132	230	ALA	B
133	231	VAL	B
134	232	LYS	B
135	233	HIS	B

Continued on next page

Table S82 – *Continued from previous page*

Homology index	Residue Index	Residue abbreviation	Module
136	234	MET	B
137	235	PRO	B
138	236	LEU	B
139	237	GLY	B
140	238	TRP	B
141	239	GLN	B
142	240	LYS	B
143	241	SER	B
144	242	TRP	B
145	243	MET	B
146	244	SER	B
147	245	SER	B
148	246	ILE	B
149	247	TYR	B
150	252	VAL	B
151	253	PHE	B
152	254	THR	B
153	255	PHE	C
154	256	GLY	B
155	257	ALA	B
156	258	TRP	B
157	259	PHE	B
158	260	LEU	B
159	261	GLY	B
160	269	ASN	B
161	270	THR	B
162	271	ASP	B
163	272	PHE	B
164	274	ASN	B
165	275	LYS	B
166	277	GLY	B
167	278	MET	B
168	279	SER	B
169	280	LEU	B
170	281	LEU	D
171	282	ASP	D
172	283	PHE	D
173	284	ARG	D
174	286	ASN	D
175	287	SER	D
176	288	ALA	D
177	289	VAL	D
178	290	ARG	D
179	291	ASN	D
180	292	VAL	D
181	293	PHE	D

Continued on next page

Table S82 – *Continued from previous page*

Homology index	Residue Index	Residue abbreviation	Module
182	298	SER	D
183	299	ASN	A
184	300	MET	A
185	301	TYR	A
186	306	MET	A
187	307	ILE	D
188	308	ASN	D
189	309	SER	D
190	310	THR	D
191	311	ALA	B
192	312	THR	B
193	316	GLN	B
194	317	VAL	B
195	318	ASN	B
196	319	ASP	B
197	320	GLN	B
198	321	VAL	D
199	322	THR	D
200	323	PHE	D
201	324	ILE	A
202	325	ASP	D
203	326	ASN	D
204	327	HIS	D
205	328	ASP	D
206	329	MET	D
207	330	ASP	D
208	331	ARG	D
209	332	PHE	D
210	333	LYS	D
211	334	THR	D
212	339	ASN	A
213	341	ARG	A
214	342	LEU	A
215	343	GLU	A
216	344	GLN	A
217	345	ALA	A
218	346	LEU	A
219	347	ALA	A
220	348	PHE	A
221	349	THR	A
222	350	LEU	A
223	351	THR	A
224	352	SER	A
225	353	ARG	D
226	354	GLY	D
227	355	VAL	D

Continued on next page

Table S82 – *Continued from previous page*

Homology index	Residue Index	Residue abbreviation	Module
228	356	PRO	A
229	357	ALA	D
230	358	ILE	A
231	359	TYR	D
232	360	TYR	C
233	361	GLY	D
234	362	THR	A
235	376	ALA	C
236	377	LYS	C
237	378	MET	D
238	379	PRO	D
239	381	PHE	D
240	387	ALA	A
241	388	PHE	A
242	389	ASN	A
243	390	VAL	A
244	391	ILE	A
245	392	SER	A
246	393	LYS	A
247	394	LEU	A
248	395	ALA	A
249	396	PRO	A
250	397	LEU	A
251	398	ARG	A
252	399	LYS	A
253	400	SER	A
254	401	ASN	A
255	402	PRO	A
256	406	TYR	A
257	407	GLY	A
258	408	SER	A
259	409	THR	A
260	411	GLN	A
261	412	ARG	A
262	413	TRP	A
263	414	ILE	A
264	415	ASN	A
265	416	ASN	A
266	417	ASP	A
267	418	VAL	A
268	419	TYR	A
269	420	VAL	A
270	421	TYR	A
271	422	GLU	A
272	423	ARG	A
273	428	SER	A

Continued on next page

Table S82 – *Continued from previous page*

Homology index	Residue Index	Residue abbreviation	Module
274	429	VAL	A
275	430	ALA	A
276	431	VAL	A
277	432	VAL	A
278	433	ALA	A
279	434	VAL	A
280	435	ASN	A
281	439	SER	A
282	440	THR	A
283	441	SER	A
284	442	ALA	A
285	443	SER	A
286	444	ILE	A
287	445	THR	A
288	454	GLY	A
289	455	SER	A
290	457	THR	A
291	458	ASP	A
292	459	VAL	A
293	460	LEU	A
294	482	ALA	A
295	483	ALA	A
296	484	GLY	A
297	485	ALA	A
298	486	THR	A
299	487	ALA	A
300	488	VAL	A
301	489	TRP	A
302	490	GLN	A

Table S83: Residues membership for the *B. licheniformis* α-amylase (PDB code 1VJS, chain A)

Homology index	Residue Index	Residue abbreviation	Module
0	5	GLY	A
1	6	THR	A
2	7	LEU	B
3	8	MET	B
4	9	GLN	B
5	10	TYR	C
6	11	PHE	C
7	15	MET	C
8	20	GLN	C

Continued on next page

Table S83 – *Continued from previous page*

Homology index	Residue Index	Residue abbreviation	Module
9	21	HIS	C
10	22	TRP	C
11	23	LYS	C
12	24	ARG	C
13	25	LEU	C
14	26	GLN	C
15	27	ASN	C
16	28	ASP	C
17	29	SER	C
18	30	ALA	C
19	31	TYR	C
20	32	LEU	C
21	33	ALA	C
22	34	GLU	C
23	35	HIS	C
24	36	GLY	C
25	37	ILE	C
26	38	THR	C
27	39	ALA	C
28	40	VAL	C
29	41	TRP	C
30	42	ILE	C
31	43	PRO	C
32	44	PRO	C
33	45	ALA	C
34	46	TYR	C
35	47	LYS	C
36	48	GLY	C
37	49	THR	C
38	50	SER	C
39	53	ASP	C
40	54	VAL	C
41	55	GLY	C
42	56	TYR	C
43	57	GLY	C
44	58	ALA	C
45	59	TYR	C
46	60	ASP	C
47	62	TYR	C
48	63	ASP	C
49	64	LEU	C
50	65	GLY	C
51	75	THR	C
52	76	LYS	C
53	77	TYR	C
54	78	GLY	C

Continued on next page

Table S83 – *Continued from previous page*

Homology index	Residue Index	Residue abbreviation	Module
55	79	THR	C
56	80	LYS	C
57	81	GLY	C
58	82	GLU	C
59	83	LEU	C
60	84	GLN	C
61	85	SER	C
62	86	ALA	C
63	87	ILE	C
64	88	LYS	C
65	89	SER	C
66	90	LEU	C
67	91	HIS	C
68	92	SER	C
69	93	ARG	C
70	94	ASP	C
71	95	ILE	C
72	96	ASN	C
73	97	VAL	C
74	98	TYR	C
75	99	GLY	C
76	100	ASP	C
77	101	VAL	C
78	102	VAL	C
79	103	ILE	C
80	104	ASN	C
81	105	HIS	C
82	106	LYS	C
83	107	GLY	C
84	108	GLY	C
85	109	ALA	C
86	141	PHE	C
87	154	LYS	C
88	155	TRP	C
89	156	HIS	C
90	157	TRP	C
91	158	TYR	C
92	159	HIS	C
93	160	PHE	C
94	161	ASP	B
95	162	GLY	B
96	163	THR	B
97	164	ASP	B
98	165	TRP	B
99	198	TYR	B
100	199	ALA	B

Continued on next page

Table S83 – *Continued from previous page*

Homology index	Residue Index	Residue abbreviation	Module
101	200	ASP	B
102	201	ILE	B
103	202	ASP	B
104	203	TYR	B
105	204	ASP	B
106	205	HIS	B
107	206	PRO	B
108	207	ASP	B
109	208	VAL	B
110	209	ALA	B
111	210	ALA	B
112	211	GLU	B
113	212	ILE	B
114	213	LYS	B
115	214	ARG	B
116	215	TRP	B
117	216	GLY	B
118	217	THR	B
119	218	TRP	B
120	219	TYR	B
121	220	ALA	B
122	221	ASN	B
123	223	LEU	C
124	224	GLN	C
125	225	LEU	C
126	226	ASP	C
127	227	GLY	C
128	228	PHE	B
129	229	ARG	D
130	230	LEU	B
131	231	ASP	B
132	232	ALA	B
133	233	VAL	B
134	234	LYS	B
135	235	HIS	B
136	236	ILE	B
137	237	LYS	B
138	238	PHE	B
139	239	SER	B
140	240	PHE	B
141	241	LEU	B
142	242	ARG	B
143	243	ASP	B
144	244	TRP	B
145	245	VAL	B
146	246	ASN	B

Continued on next page

Table S83 – *Continued from previous page*

Homology index	Residue Index	Residue abbreviation	Module
147	247	HIS	B
148	248	VAL	B
149	249	ARG	B
150	256	MET	B
151	257	PHE	B
152	258	THR	B
153	259	VAL	C
154	260	ALA	B
155	261	GLU	B
156	262	TYR	B
157	263	TRP	B
158	264	GLN	B
159	265	ASN	B
160	270	LEU	B
161	271	GLU	B
162	272	ASN	B
163	273	TYR	B
164	274	LEU	B
165	275	ASN	B
166	280	ASN	B
167	281	HIS	B
168	282	SER	B
169	283	VAL	B
170	284	PHE	D
171	285	ASP	D
172	286	VAL	D
173	287	PRO	D
174	289	HIS	D
175	290	TYR	D
176	291	GLN	D
177	292	PHE	D
178	293	HIS	D
179	294	ALA	D
180	295	ALA	D
181	296	SER	D
182	302	TYR	D
183	303	ASP	A
184	304	MET	A
185	305	ARG	A
186	307	LEU	A
187	308	LEU	D
188	309	ASN	D
189	310	SER	D
190	313	VAL	D
191	314	SER	B
192	315	LYS	B

Continued on next page

Table S83 – *Continued from previous page*

Homology index	Residue Index	Residue abbreviation	Module
193	316	HIS	B
194	317	PRO	B
195	318	LEU	B
196	319	LYS	B
197	320	ALA	B
198	321	VAL	D
199	322	THR	D
200	323	PHE	D
201	324	VAL	A
202	325	ASP	D
203	326	ASN	D
204	327	HIS	D
205	328	ASP	D
206	329	THR	D
207	330	GLN	D
208	338	THR	D
209	339	VAL	D
210	340	GLN	D
211	341	THR	D
212	342	TRP	A
213	343	PHE	A
214	344	LYS	A
215	345	PRO	A
216	346	LEU	A
217	347	ALA	A
218	348	TYR	A
219	349	ALA	A
220	350	PHE	A
221	351	ILE	A
222	352	LEU	A
223	353	THR	A
224	354	ARG	A
225	356	SER	D
226	357	GLY	D
227	358	TYR	D
228	359	PRO	A
229	360	GLN	D
230	361	VAL	A
231	362	PHE	D
232	363	TYR	C
233	364	GLY	D
234	365	ASP	A
235	366	MET	C
236	367	TYR	C
237	368	GLY	D
238	369	THR	D

Continued on next page

Table S83 – *Continued from previous page*

Homology index	Residue Index	Residue abbreviation	Module
239	379	ALA	D
240	380	LEU	A
241	381	LYS	A
242	382	HIS	A
243	383	LYS	A
244	384	ILE	A
245	385	GLU	A
246	386	PRO	A
247	387	ILE	A
248	388	LEU	A
249	389	LYS	A
250	390	ALA	A
251	391	ARG	A
252	392	LYS	A
253	393	GLN	A
254	394	TYR	A
255	395	ALA	A
256	397	GLY	A
257	398	ALA	A
258	399	GLN	A
259	400	HIS	A
260	401	ASP	A
261	402	TYR	A
262	403	PHE	A
263	404	ASP	A
264	405	HIS	A
265	406	HIS	A
266	407	ASP	A
267	408	ILE	A
268	409	VAL	A
269	410	GLY	A
270	411	TRP	A
271	412	THR	A
272	413	ARG	A
273	414	GLU	A
274	415	GLY	A
275	424	LEU	A
276	425	ALA	A
277	426	ALA	A
278	427	LEU	A
279	428	ILE	A
280	429	THR	A
281	431	GLY	A
282	432	PRO	A
283	433	GLY	A
284	434	GLY	A

Continued on next page

Table S83 – *Continued from previous page*

Homology index	Residue Index	Residue abbreviation	Module
285	435	ALA	A
286	436	LYS	A
287	437	ARG	A
288	446	GLY	A
289	447	GLU	A
290	449	TRP	A
291	450	HIS	A
292	451	ASP	A
293	452	ILE	A
294	472	VAL	A
295	473	ASN	A
296	474	GLY	A
297	476	SER	A
298	477	VAL	A
299	478	SER	A
300	479	ILE	A
301	480	TYR	A
302	481	VAL	A

Table S84: Residues membership for the *T. vulgaris R47* α-amylase (PDB code 1WZK, chain A)

Homology index	Residue Index	Residue abbreviation	Module
0	131	ALA	A
1	132	VAL	A
2	133	ILE	B
3	134	TYR	B
4	135	GLN	B
5	136	ILE	C
6	137	PHE	C
7	138	PRO	C
8	170	GLY	C
9	171	ASP	C
10	172	LEU	C
11	173	LYS	C
12	174	GLY	C
13	175	VAL	C
14	176	ILE	C
15	177	ASP	C
16	178	ARG	C
17	179	LEU	C
18	180	PRO	C
19	181	TYR	C

Continued on next page

Table S84 – *Continued from previous page*

**Homology index**	**Residue Index**	**Residue abbreviation**	**Module**
20	182	LEU	C
21	183	GLU	C
22	184	GLU	C
23	185	LEU	C
24	186	GLY	C
25	187	VAL	C
26	188	THR	C
27	189	ALA	C
28	190	LEU	C
29	191	TYR	C
30	192	PHE	C
31	193	THR	C
32	194	PRO	C
33	195	ILE	C
34	196	PHE	C
35	197	ALA	C
36	198	SER	C
37	199	PRO	C
38	200	SER	C
39	201	HIS	C
40	202	HIS	C
41	203	LYS	C
42	204	TYR	C
43	205	ASP	C
44	206	THR	C
45	207	ALA	C
46	208	ASP	C
47	210	LEU	C
48	211	ALA	C
49	212	ILE	C
50	213	ASP	C
51	214	PRO	C
52	215	GLN	C
53	216	PHE	C
54	217	GLY	C
55	218	ASP	C
56	219	LEU	C
57	220	PRO	C
58	221	THR	C
59	222	PHE	C
60	223	ARG	C
61	224	ARG	C
62	225	LEU	C
63	226	VAL	C
64	227	ASP	C
65	228	GLU	C

Continued on next page

Table S84 – *Continued from previous page*

Homology index	Residue Index	Residue abbreviation	Module
66	229	ALA	C
67	230	HIS	C
68	231	ARG	C
69	232	ARG	C
70	233	GLY	C
71	234	ILE	C
72	235	LYS	C
73	236	ILE	C
74	237	ILE	C
75	238	LEU	C
76	239	ASP	C
77	240	ALA	C
78	241	VAL	C
79	242	PHE	C
80	243	ASN	C
81	244	HIS	C
82	245	ALA	C
83	246	GLY	C
84	247	ASP	C
85	248	GLN	C
86	249	PHE	C
87	250	PHE	C
88	251	ALA	C
89	252	PHE	C
90	265	LYS	C
91	266	ASP	C
92	267	TRP	C
93	268	PHE	C
94	269	PHE	B
95	284	GLU	B
96	285	THR	B
97	286	PHE	B
98	287	ALA	B
99	293	MET	B
100	294	PRO	B
101	295	LYS	B
102	296	LEU	B
103	297	ARG	B
104	298	THR	B
105	299	GLU	B
106	300	ASN	B
107	301	PRO	B
108	302	GLU	B
109	303	VAL	B
110	304	LYS	B
111	305	GLU	B

Continued on next page

Table S84 – *Continued from previous page*

Homology index	Residue Index	Residue abbreviation	Module
112	306	TYR	B
113	307	LEU	B
114	308	PHE	B
115	309	ASP	B
116	310	VAL	B
117	311	ALA	B
118	312	ARG	B
119	313	PHE	B
120	314	TRP	B
121	315	MET	B
122	316	GLU	B
123	317	GLN	C
124	318	GLY	C
125	319	ILE	C
126	320	ASP	C
127	321	GLY	C
128	322	TRP	B
129	323	ARG	D
130	324	LEU	B
131	325	ASP	B
132	326	VAL	B
133	327	ALA	B
134	328	ASN	B
135	329	GLU	B
136	330	VAL	B
137	331	ASP	B
138	332	HIS	B
139	333	ALA	B
140	334	PHE	B
141	335	TRP	B
142	336	ARG	B
143	337	GLU	B
144	338	PHE	B
145	339	ARG	B
146	340	ARG	B
147	341	LEU	B
148	342	VAL	B
149	343	LYS	B
150	349	ALA	B
151	350	LEU	B
152	351	ILE	B
153	352	VAL	C
154	353	GLY	B
155	354	GLU	B
156	355	ILE	B
157	356	TRP	B

Continued on next page

Table S84 – *Continued from previous page*

Homology index	Residue Index	Residue abbreviation	Module
158	357	HIS	B
159	358	ASP	B
160	359	ALA	B
161	360	SER	B
162	361	GLY	B
163	362	TRP	B
164	363	LEU	B
165	364	MET	B
166	368	PHE	B
167	369	ASP	B
168	370	SER	B
169	371	VAL	B
170	372	MET	D
171	373	ASN	D
172	374	TYR	D
173	375	LEU	D
174	377	ARG	D
175	378	GLU	D
176	379	SER	D
177	380	VAL	D
178	381	ILE	D
179	382	ARG	D
180	383	PHE	D
181	384	PHE	D
182	389	ILE	D
183	390	HIS	A
184	391	ALA	A
185	392	GLU	A
186	397	GLU	A
187	398	LEU	D
188	399	THR	D
189	400	ARG	D
190	401	ALA	D
191	402	ARG	B
192	403	MET	B
193	409	ALA	B
194	410	ALA	B
195	411	GLN	B
196	412	GLY	B
197	413	LEU	B
198	414	TRP	D
199	415	ASN	D
200	416	LEU	D
201	417	LEU	A
202	418	ASP	D
203	419	SER	D

Continued on next page

Table S84 – *Continued from previous page*

Homology index	Residue Index	Residue abbreviation	Module
204	420	HIS	D
205	421	ASP	D
206	422	THR	D
207	423	GLU	D
208	424	ARG	D
209	425	PHE	D
210	426	LEU	D
211	427	THR	D
212	433	GLU	A
213	435	LYS	A
214	436	PHE	A
215	437	ARG	A
216	438	LEU	A
217	439	ALA	A
218	440	VAL	A
219	441	LEU	A
220	442	PHE	A
221	443	GLN	A
222	444	MET	A
223	445	THR	A
224	446	TYR	A
225	447	LEU	D
226	448	GLY	D
227	449	THR	D
228	450	PRO	A
229	451	LEU	D
230	452	ILE	A
231	453	TYR	D
232	454	TYR	C
233	455	GLY	D
234	456	ASP	A
235	470	ARG	C
236	471	PRO	C
237	472	MET	D
238	473	ILE	D
239	474	TRP	D
240	483	LEU	A
241	484	PHE	A
242	485	GLU	A
243	486	PHE	A
244	487	TYR	A
245	488	LYS	A
246	489	GLU	A
247	490	LEU	A
248	491	ILE	A
249	492	ARG	A

Continued on next page

Table S84 – *Continued from previous page*

Homology index	Residue Index	Residue abbreviation	Module
250	493	LEU	A
251	494	ARG	A
252	495	HIS	A
253	496	ARG	A
254	497	LEU	A
255	498	ALA	A
256	502	ARG	A
257	503	GLY	A
258	504	ASN	A
259	505	VAL	A
260	507	SER	A
261	508	TRP	A
262	509	HIS	A
263	510	ALA	A
264	513	GLN	A
265	514	ALA	A
266	515	ASN	A
267	516	LEU	A
268	517	TYR	A
269	518	ALA	A
270	519	PHE	A
271	520	VAL	A
272	521	ARG	A
273	526	GLN	A
274	527	HIS	A
275	528	VAL	A
276	529	GLY	A
277	530	VAL	A
278	531	VAL	A
279	532	LEU	A
280	533	ASN	A
281	536	GLY	A
282	537	GLU	A
283	538	LYS	A
284	539	GLN	A
285	540	THR	A
286	541	VAL	A
287	542	LEU	A
288	552	THR	A
289	553	TRP	A
290	554	LEU	A
291	555	ASP	A
292	556	CYS	A
293	557	LEU	A
294	574	ARG	A
295	575	PRO	A

Continued on next page

Table S84 – *Continued from previous page*

Homology index	Residue Index	Residue abbreviation	Module
296	576	TYR	A
297	577	GLN	A
298	578	GLY	A
299	579	MET	A
300	580	ILE	A
301	581	LEU	A
302	582	TRP	A

Table S85: Residues membership for the *N. polysaccharea* α-amylase (PDB code 1JGI, chain A)

Homology index	Residue Index	Residue abbreviation	Module
0	98	GLN	A
1	99	VAL	A
2	100	GLY	B
3	101	GLY	B
4	102	VAL	B
5	103	CYS	C
6	104	TYR	C
7	105	VAL	C
8	110	GLY	C
9	111	ASP	C
10	112	LEU	C
11	113	LYS	C
12	114	GLY	C
13	115	LEU	C
14	116	LYS	C
15	117	ASP	C
16	118	LYS	C
17	119	ILE	C
18	120	PRO	C
19	121	TYR	C
20	122	PHE	C
21	123	GLN	C
22	124	GLU	C
23	125	LEU	C
24	126	GLY	C
25	127	LEU	C
26	128	THR	C
27	129	TYR	C
28	130	LEU	C
29	131	TYR	C
30	132	LEU	C

Continued on next page

Table S85 – *Continued from previous page*

Homology index	Residue Index	Residue abbreviation	Module
31	133	MET	C
32	134	PRO	C
33	135	LEU	C
34	136	PHE	C
35	137	LYS	C
36	138	CYS	C
37	139	PRO	C
38	141	GLY	C
39	144	ASP	C
40	145	GLY	C
41	146	GLY	C
42	147	TYR	C
43	148	ALA	C
44	149	VAL	C
45	150	SER	C
46	151	SER	C
47	153	ARG	C
48	154	ASP	C
49	155	VAL	C
50	156	ASN	C
51	157	PRO	C
52	158	ALA	C
53	159	LEU	C
54	160	GLY	C
55	161	THR	C
56	162	ILE	C
57	163	GLY	C
58	164	ASP	C
59	165	LEU	C
60	166	ARG	C
61	167	GLU	C
62	168	VAL	C
63	169	ILE	C
64	170	ALA	C
65	171	ALA	C
66	172	LEU	C
67	173	HIS	C
68	174	GLU	C
69	175	ALA	C
70	176	GLY	C
71	177	ILE	C
72	178	SER	C
73	179	ALA	C
74	180	VAL	C
75	181	VAL	C
76	182	ASP	C

Continued on next page

Table S85 – *Continued from previous page*

Homology index	Residue Index	Residue abbreviation	Module
77	183	PHE	C
78	184	ILE	C
79	185	PHE	C
80	186	ASN	C
81	187	HIS	C
82	188	THR	C
83	189	SER	C
84	190	ASN	C
85	191	GLU	C
86	192	HIS	C
87	193	GLU	C
88	194	TRP	C
89	195	ALA	C
90	206	ASP	C
91	207	ASN	C
92	208	PHE	C
93	209	TYR	C
94	210	TYR	B
95	247	TRP	B
96	248	THR	B
97	249	THR	B
98	250	PHE	B
99	254	GLN	B
100	255	TRP	B
101	256	ASP	B
102	257	LEU	B
103	258	ASN	B
104	259	TYR	B
105	260	SER	B
106	261	ASN	B
107	262	PRO	B
108	263	TRP	B
109	264	VAL	B
110	265	PHE	B
111	266	ARG	B
112	267	ALA	B
113	268	MET	B
114	269	ALA	B
115	270	GLY	B
116	271	GLU	B
117	272	MET	B
118	273	LEU	B
119	274	PHE	B
120	275	LEU	B
121	276	ALA	B
122	277	ASN	B

Continued on next page

Table S85 – *Continued from previous page*

Homology index	Residue Index	Residue abbreviation	Module
123	278	LEU	C
124	279	GLY	C
125	280	VAL	C
126	281	ASP	C
127	282	ILE	C
128	283	LEU	B
129	284	ARG	D
130	285	MET	B
131	286	ASP	B
132	287	ALA	B
133	288	VAL	B
134	289	ALA	B
135	290	PHE	B
136	291	ILE	B
137	292	TRP	B
138	306	HIS	B
139	307	ALA	B
140	308	LEU	B
141	309	ILE	B
142	310	ARG	B
143	311	ALA	B
144	312	PHE	B
145	313	ASN	B
146	314	ALA	B
147	315	VAL	B
148	316	MET	B
149	317	ARG	B
150	323	VAL	B
151	324	PHE	B
152	325	PHE	B
153	326	LYS	C
154	327	SER	B
155	328	GLN	B
156	329	ALA	B
157	330	ILE	B
158	331	VAL	B
159	332	HIS	B
160	336	VAL	B
161	337	VAL	B
162	338	GLN	B
163	339	TYR	B
164	340	ILE	B
165	341	GLY	B
166	345	CYS	B
167	346	GLN	B
168	347	ILE	B

Continued on next page

Table S85 – *Continued from previous page*

Homology index	Residue Index	Residue abbreviation	Module
169	348	GLY	B
170	349	TYR	D
171	350	ASN	D
172	351	PRO	D
173	352	LEU	D
174	354	MET	D
175	355	ALA	D
176	356	LEU	D
177	357	LEU	D
178	358	TRP	D
179	359	ASN	D
180	360	THR	D
181	361	LEU	D
182	364	ARG	D
183	365	GLU	A
184	366	VAL	A
185	367	ASN	A
186	372	ALA	A
187	373	LEU	D
188	374	THR	D
189	375	TYR	D
190	376	ARG	D
191	377	HIS	B
192	378	ASN	B
193	381	GLU	B
194	382	HIS	B
195	383	THR	B
196	384	ALA	B
197	385	TRP	B
198	386	VAL	D
199	387	ASN	D
200	388	TYR	D
201	389	VAL	A
202	390	ARG	D
203	391	SER	D
204	392	HIS	D
205	393	ASP	D
206	394	ASP	D
207	448	SER	D
208	450	THR	D
209	451	ALA	D
210	452	ALA	D
211	453	ALA	D
212	464	ALA	A
213	467	ARG	A
214	468	ILE	A

Continued on next page

Table S85 – *Continued from previous page*

Homology index	Residue Index	Residue abbreviation	Module
215	469	LYS	A
216	470	LEU	A
217	471	LEU	A
218	472	TYR	A
219	473	SER	A
220	474	ILE	A
221	475	ALA	A
222	476	LEU	A
223	477	SER	A
224	478	THR	A
225	479	GLY	D
226	480	GLY	D
227	481	LEU	D
228	482	PRO	A
229	483	LEU	D
230	484	ILE	A
231	485	TYR	D
232	486	LEU	C
233	487	GLY	D
234	488	ASP	A
235	514	PRO	C
236	515	ARG	C
237	516	TYR	D
238	517	ASN	D
239	521	TYR	D
240	534	ILE	A
241	535	TYR	A
242	536	GLN	A
243	537	ASP	A
244	538	LEU	A
245	539	ARG	A
246	540	HIS	A
247	541	MET	A
248	542	ILE	A
249	543	ALA	A
250	544	VAL	A
251	545	ARG	A
252	546	GLN	A
253	547	SER	A
254	548	ASN	A
255	549	PRO	A
256	553	GLY	A
257	554	GLY	A
258	555	ARG	A
259	556	LEU	A
260	558	THR	A

Continued on next page

Table S85 – *Continued from previous page*

Homology index	Residue Index	Residue abbreviation	Module
261	559	PHE	A
262	560	ASN	A
263	561	THR	A
264	563	ASN	A
265	564	LYS	A
266	565	HIS	A
267	566	ILE	A
268	567	ILE	A
269	568	GLY	A
270	569	TYR	A
271	570	ILE	A
272	571	ARG	A
273	573	ASN	A
274	574	ALA	A
275	575	LEU	A
276	576	LEU	A
277	577	ALA	A
278	578	PHE	A
279	579	GLY	A
280	580	ASN	A
281	584	TYR	A
282	585	PRO	A
283	586	GLN	A
284	587	THR	A
285	588	VAL	A
286	589	THR	A
287	590	ALA	A
288	599	LYS	A
289	600	ALA	A
290	601	HIS	A
291	602	ASP	A
292	603	LEU	A
293	604	ILE	A
294	617	LEU	A
295	618	GLN	A
296	619	PRO	A
297	621	GLN	A
298	622	VAL	A
299	623	MET	A
300	624	TRP	A
301	625	LEU	A
302	626	GLU	A

Table S86: Residues membership for the *H. sapiens* NPC1 (PDB code 3GKH, chain A)protein

**Homology index**	**Residue Index**	**Residue abbreviation**	**Module**
0	23	GLN	A
1	24	SER	A
2	25	CYS	A
3	26	VAL	A
4	27	TRP	A
5	28	TYR	A
6	29	GLY	A
7	30	GLU	A
8	31	CYS	A
9	32	GLY	B
10	33	ILE	B
11	34	ALA	B
12	35	TYR	B
13	36	GLY	B
14	37	ASP	B
15	38	LYS	B
16	39	ARG	B
17	40	TYR	B
18	41	ASN	A
19	42	CYS	A
20	43	GLU	B
21	44	TYR	A
22	45	SER	A
23	46	GLY	A
24	47	PRO	A
25	48	PRO	A
26	49	LYS	A
27	50	PRO	A
28	51	LEU	A
29	52	PRO	A
30	53	LYS	A
31	54	ASP	A
32	55	GLY	A
33	56	TYR	A
34	57	ASP	A
35	58	LEU	A
36	59	VAL	A
37	60	GLN	A
38	61	GLU	A
39	62	LEU	A
40	63	CYS	A
41	64	PRO	A
42	65	GLY	A
43	66	PHE	A

Continued on next page

Table S86 – *Continued from previous page*

Homology index	Residue Index	Residue abbreviation	Module
44	67	PHE	A
45	68	PHE	A
46	69	GLY	A
47	70	GLN	A
48	71	VAL	A
49	72	SER	A
50	73	LEU	A
51	74	CYS	A
52	75	CYS	A
53	76	ASP	A
54	77	VAL	A
55	78	ARG	A
56	79	GLN	A
57	80	LEU	A
58	81	GLN	A
59	82	THR	A
60	83	LEU	A
61	84	LYS	A
62	85	ASP	A
63	86	ASN	B
64	87	LEU	B
65	88	GLN	B
66	89	LEU	B
67	90	PRO	B
68	91	LEU	B
69	92	GLN	B
70	93	PHE	E
71	94	LEU	E
72	95	SER	E
73	96	ARG	E
74	97	CYS	E
75	98	PRO	E
76	99	SER	E
77	100	CYS	E
78	101	PHE	E
79	102	TYR	E
80	103	ASN	E
81	104	LEU	E
82	105	LEU	E
83	106	ASN	E
84	107	LEU	F
85	108	PHE	F
86	109	CYS	A
87	110	GLU	F
88	111	LEU	F
89	112	THR	A

Continued on next page

Table S86 – *Continued from previous page*

Homology index	Residue Index	Residue abbreviation	Module
90	113	CYS	A
91	114	SER	A
92	115	PRO	A
93	116	ARG	A
94	117	GLN	A
95	118	SER	A
96	119	GLN	A
97	120	PHE	F
98	121	LEU	F
99	122	GLN	F
100	123	VAL	F
101	124	THR	F
102	125	ALA	B
103	126	THR	B
104	127	GLU	B
105	128	ASP	B
106	129	TYR	B
107	130	VAL	B
108	131	ASP	B
109	132	PRO	B
110	133	VAL	B
111	134	THR	B
112	135	ASN	B
113	136	GLN	B
114	137	THR	B
115	138	LYS	A
116	139	THR	B
117	140	ASN	B
118	141	VAL	B
119	142	LYS	B
120	143	GLU	F
121	144	LEU	F
122	145	GLN	F
123	146	TYR	F
124	147	TYR	F
125	148	VAL	F
126	149	GLY	F
127	150	GLN	F
128	151	SER	F
129	152	PHE	F
130	153	ALA	F
131	154	ASN	F
132	155	ALA	E
133	156	MET	E
134	157	TYR	E
135	158	ASN	E

Continued on next page

Table S86 – *Continued from previous page*

Homology index	Residue Index	Residue abbreviation	Module
136	159	ALA	E
137	160	CYS	E
138	161	ARG	E
139	162	ASP	E
140	163	VAL	E
141	164	GLU	E
142	165	ALA	E
143	166	PRO	E
144	167	SER	E
145	168	SER	E
146	169	ASN	E
147	170	ASP	E
148	171	LYS	E
149	172	ALA	E
150	173	LEU	E
151	174	GLY	E
152	175	LEU	E
153	176	LEU	E
154	177	CYS	E
155	178	GLY	E
156	179	LYS	E
157	180	ASP	E
158	181	ALA	E
159	182	ASP	E
160	183	ALA	E
161	184	CYS	E
162	185	GLN	E
163	186	ALA	E
164	187	THR	E
165	188	ASN	E
166	189	TRP	D
167	190	ILE	F
168	191	GLU	E
169	192	TYR	E
170	193	MET	D
171	194	PHE	F
172	195	ASN	E
173	196	LYS	B
174	197	ASP	C
175	198	ASN	C
176	199	GLY	C
177	200	GLN	C
178	201	ALA	C
179	202	PRO	C
180	203	PHE	F
181	204	THR	F

Continued on next page

Table S86 – *Continued from previous page*

Homology index	Residue Index	Residue abbreviation	Module
182	205	ILE	F
183	206	THR	F
184	207	PRO	F
185	208	VAL	F
186	209	PHE	F
187	210	SER	F
188	211	ASP	F
189	212	PHE	F
190	213	PRO	F
191	214	VAL	F
192	215	HIS	F
193	216	GLY	F
194	217	MET	F
195	218	GLU	F
196	219	PRO	F
197	220	MET	F
198	221	ASN	F
199	222	ASN	F
200	223	ALA	F
201	224	THR	D
202	225	LYS	D
203	226	GLY	D
204	227	CYS	D
205	228	ASP	D
206	229	GLU	D
207	230	SER	D
208	231	VAL	D
209	232	ASP	D
210	233	GLU	D
211	234	VAL	D
212	235	THR	D
213	236	ALA	D
214	237	PRO	D
215	238	CYS	D
216	239	SER	D
217	240	CYS	D
218	241	GLN	D
219	242	ASP	D
220	243	CYS	D
221	244	SER	D
222	245	ILE	D
223	246	VAL	D
